# Practical and Selective sp^3^ C−H Bond Chlorination via Aminium Radicals

**DOI:** 10.1002/anie.202100030

**Published:** 2021-02-25

**Authors:** Alastair J. McMillan, Martyna Sieńkowska, Piero Di Lorenzo, Gemma K. Gransbury, Nicholas F. Chilton, Michela Salamone, Alessandro Ruffoni, Massimo Bietti, Daniele Leonori

**Affiliations:** ^1^ Department of Chemistry University of Manchester Oxford Road Manchester M13 9PL UK; ^2^ Dipartimento di Scienze e Tecnologie Chimiche Università “Tor Vergata” Via della Ricerca Scientifica 00133 Rome Italy

**Keywords:** aminium radical, C−H functionalization, chlorination, H-atom transfer, late-stage functionalization

## Abstract

The introduction of chlorine atoms into organic molecules is fundamental to the manufacture of industrial chemicals, the elaboration of advanced synthetic intermediates and also the fine‐tuning of physicochemical and biological properties of drugs, agrochemicals and polymers. We report here a general and practical photochemical strategy enabling the site‐selective chlorination of sp^3^ C−H bonds. This process exploits the ability of protonated *N*‐chloroamines to serve as aminium radical precursors and also radical chlorinating agents. Upon photochemical initiation, an efficient radical‐chain propagation is established allowing the functionalization of a broad range of substrates due to the large number of compatible functionalities. The ability to synergistically maximize both polar and steric effects in the H‐atom transfer transition state through appropriate selection of the aminium radical has provided the highest known selectivity in radical sp^3^ C−H chlorination.

## Graphical abstract

Direct and selective sp^3^ C−H chlorination has been achieved using a photoinduced strategy. This approach is based on a radical‐chain propagation that exploits the ability of aminium radicals to undergo site‐selective H‐atom transfer (HAT).

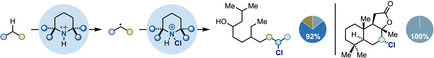

## Introduction

**Scheme 1:**
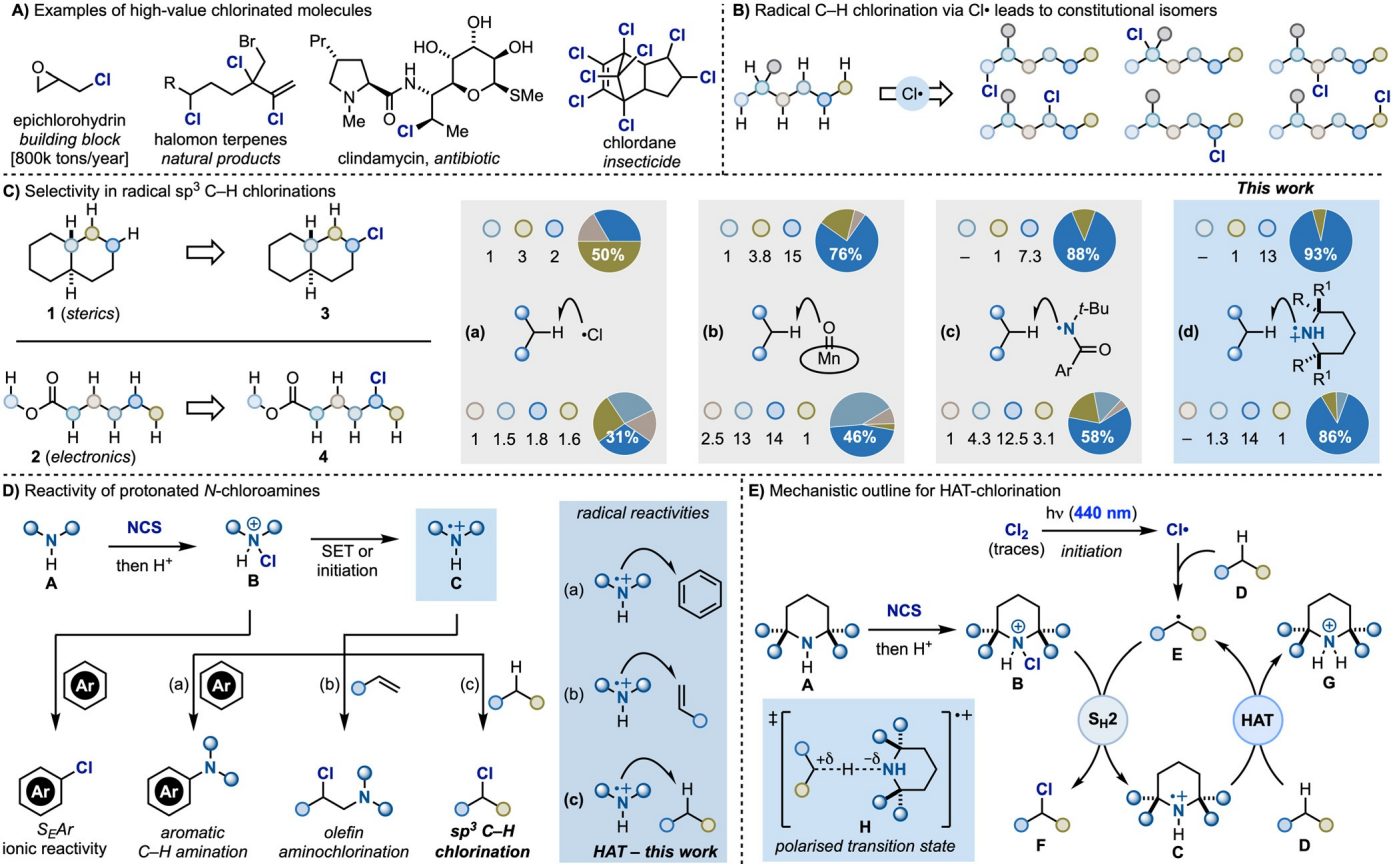
A) Examples of high‐value chlorinated molecules. B) Classical approaches to sp^3^ C−H chlorination via Cl^.^ lead to isomeric mixtures. C) Achieving selectivity in sp^3^ C−H chlorination. D) Reactivity patterns of protonated *N*‐chloroamines: ionic vs. radical processes. E) Proposed mechanistic analysis for HAT chlorination with aminium radicals. SET=single‐electron transfer; S_H_2=bimolecular homolytic substitution; S_E_Ar=electrophilic aromatic substitution.

Chlorine‐containing molecules are integral to all chemical disciplines (Scheme 1 A).^[1]^ It has been estimated that the manufacture of >50 % of industrial chemicals and >20 % of pharmaceutical products requires at some point the introduction of a chlorine atom into a feedstock or an advanced building block, respectively.^[2]^ Methodologies able to target the selective chlorination of organic compounds are therefore of high importance to enable further elaboration of key synthetic intermediates^[3]^ and also to aid the modulation of physical and biological properties of high‐value final products.^[4]^

Radical sp^3^ C−H chlorination via hydrogen‐atom transfer (HAT) would be an ideal approach to access these building blocks and indeed it is widely used in the industrial valorisation of light alkanes.^[2]^ However, as these processes are based on the radical chain reactivity of chlorine radicals (Cl^.^), they require harsh conditions, are difficult to control and deliver complex mixtures of all possible constitutional isomers (Scheme 1 B).^[5]^ Overall, achieving high selectivity in radical sp^3^ C−H chlorination is still challenging because it requires fine control of the interplay between multiple factors (e.g. enthalpic, polar, steric and stereoelectronic, conjugative and hyperconjugative, torsional and strain) that determine the reactivity of each individual sp^3^ C−H bond.^[6]^

The state‐of‐the‐art in radical sp^3^ C−H chlorination is summarised in Scheme 1 C using *trans*‐decalin **1** and methylhexanoate **2**, two substrates whose HAT‐chlorination is mainly affected by steric and polar factors, respectively. While methodologies based on Cl^.^ radicals are unselective and also lead to poly‐chlorination (Scheme 1 C, box a), systems displaying strong bias for specific positions and leading selectively to **3** ^[7]^ and **4** ^[8]^ have been reported. Groves developed a bioinspired Mn^III^‐porphyrin catalyst able, upon oxidation to Mn^V^=O, to sterically differentiate between sp^3^ C−H bonds. This biphasic system uses NaOCl as both the oxidant and the Cl‐source and often requires an excess of the hydrocarbon substrate (Scheme 1 C, box b).^[9]^ Following the pioneering work of Greene on C−H chlorination using *N*‐*t*‐Bu,*N*‐chloroamide,^[10]^ Alexanian has identified two bench‐stable *N*‐*t*‐Bu,*N*‐chlorobenzamide reagents that, depending on the aromatic substitution pattern, target specific sp^3^ C−H bonds on the basis of either electronic or steric factors (Scheme 1 C, box c).^[11]^ In both cases, photochemical or thermal conditions generate an amidyl radical that participates in a HAT‐based radical‐chain chlorination.

Despite these powerful advances, a methodology able to selectively discriminate between very similar sp^3^ C−H bonds in organic molecules is still a challenge, especially when broad functional group compatibility is required. Here, we describe a practical process for site‐selective sp^3^ C−H chlorination via aminium radicals (Scheme 1 C, box d). This strategy does not require the preparation of bespoke catalysts/reagents and uses simple cyclic amines in combination with *N*‐chlorosuccinimide (NCS). The large structural modularity of commercially available amines facilitates easy tuning of the steric properties of the aminium radicals which, combined with their intrinsic high electrophilicity, has provided the highest known selectivity in radical sp^3^ C−H chlorination.

### Design Plan

We have recently developed photoinduced protocols for aromatic C−H amination and olefin diamination exploiting the generation of aminium radicals from both 1° and 2° alkylamines (Scheme 1 D).^[12]^ These processes rely on in situ amine activation by chlorination followed by protonation (**A** → **B**). The corresponding *N*‐Cl‐ammonium salts **B** are powerful electrophiles in classical ionic chemistry,^[13]^ but upon judicious choice of the reaction conditions their reactivity can be diverted into radical manifolds based on aminium radicals **C**. These species are isoelectronic with alkyl radicals but carry a formal positive charge which dramatically enhances their reactivity towards electron rich π‐systems [see Scheme 1 D, paths (a) and (b)].^[14]^ Pioneering work from Minisci^[15]^ and Deno^[16]^ has also demonstrated that these open‐shell intermediates can be used in radical HAT‐chlorination but required preformed *N*‐chloroamines, H_2_SO_4_ as solvent and either high‐energy UV‐irradiation (*λ*=254 nm) or an excess of FeSO_4_. Despite the potential of this reactivity mode for sp^3^ C−H functionalization, the harsh reaction conditions combined with the known difficulties in preparing and isolating *N*‐chloroamines, have thwarted its adoption and exploitation by the synthetic community.

We recently questioned if our mild activation protocol for aminium radical generation could be adapted for the realization of a synthetic HAT‐functionalization platform enabling site‐selective sp^3^ C−H chlorination. As shown in Scheme 1 E, our strategy is based on amine activation (chlorination and protonation, **A** → **B**), followed by photoinduced initiation to start a radical‐chain propagation centred on aminium radical **C**. We were hopeful that **C** would enable site‐selective HAT from the aliphatic C−H bond of an organic molecule **D** and give the corresponding radical **E** and the protonated amine **G**. Chlorine‐transfer (S_H_2) between **B** and **E** would provide the chlorinated product **F**, and, crucially, would regenerate the chain‐carrying aminium radical **C**. There are four intrinsic features that should synergistically maximise “reactivity & selectivity” aspects in this manifold. (1) The N–H BDE in **G** is ≈103 kcal mol^−1^,^[17]^ which is larger than the BDEs of most sp^3^ C−H bonds (e.g. BDE for C−H in cyclohexane=99 kcal mol^−1^)^[18]^ and should provide the enthalpic driving force for the HAT step. (2) The strong electrophilic character of aminium radicals [calculated local electrophilicity index (ω^+^_rc_)^[19]^ for piperidinium radical ≈4.2 eV]^[20]^ is expected to provide extensive charge‐transfer character to the HAT transition state **H**. This kinetic polar effect should impart significant stabilisation and critically amplify the innate electronic differentiation of the sp^3^ C−H bonds.^[21]^ (3) The ability to prepare the HAT‐chlorination reagents **B** in situ from simple amines ought to enable the fine tuning of the chemical environment around the aminium radical thus maximising C−H discrimination in the HAT step based mostly on sterics. (4) A common drawback in radical sp^3^ C−H chlorination is the formation of polychlorinated materials. In our case, the great sensitivity of aminium radicals to polar inductive effects means that the Cl substituent in product **F** acts as a sp^3^ C−H deactivating group thus insulating the product from further and unwanted functionalization.^[22]^

Regarding the initiation process, we initially considered the use of a photoredox catalyst [for example, Ru(bpy)_3_Cl_2_] but we quickly realised that simple blue light irradiation was sufficient to sustain chain propagation and achieve high chemical yields. Our current working hypothesis for this step involves the adventitious photochemical generation of Cl^.^ that would perform the very first HAT (low selectivity) and then enable the generation of the key aminium radical **C**.

### Reaction Optimization

**Scheme 2:**
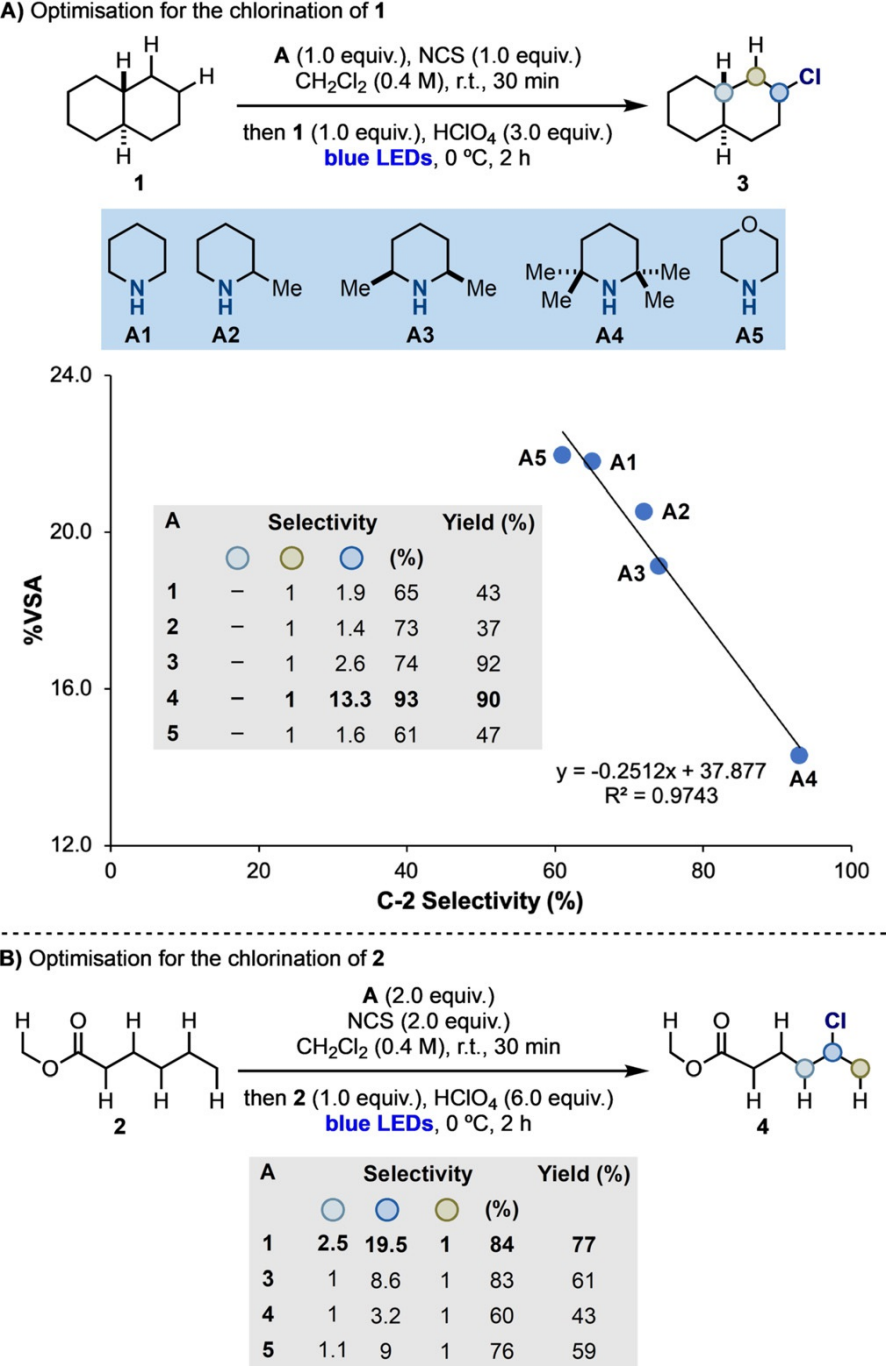
A) Optimization of the sp^3^ C−H chlorination on **1**. B) Optimization of the sp^3^ C−H chlorination on **2**.

We started the optimization of the sp^3^ C−H chlorination process using *trans*‐decalin **1** in order to focus on HAT discrimination based on steric effects. Following the procedure reported in Scheme 2 A, 21 secondary amines **A** (cyclic and acyclic) were screened and all provided preferentially the C‐2 chlorinated product **3** in moderate to high yields.^[23]^ The selectivity of the process depended on the amine structure and in no case abstraction of the weaker tertiary sp^3^ C−H bond (C−H BDE=91 kcal mol^−1^)^[24]^ was observed. This demonstrates the difference in HAT site‐selectivity of this approach vs. other aminium radicals like quinuclidinium or triethylenediammonium bis(tetrafluoroborate)^.+^ (TEDA^.+^) which have been shown to functionalize both 2° and 3° sites.^[25]^

To evaluate the steric hinderance at the aminium radical centre, we employed a simple line‐of‐sight methodology in our *AtomAccess* code^[26]^ and determined the percentage visible solid angle (%VSA) of the *N*‐atom.^[20]^ Pleasingly, a trend between the C‐2 selectivity and the %VSA for all amines was observed despite their large structural difference,^[20]^ and a clear correlation was identified in the case of the piperidine‐based derivatives **A1**–**5** (Scheme 2 A). In this case, the sequential introduction of substituents at C‐2 and C‐6 of the piperidine core resulted in increased steric hinderance that translated into higher selectivity for chlorination at C‐2. In particular, the use of 2,2,6,6‐tetramethylpiperidine (TMP, **A4**) enabled to reach a ratio C‐2:C‐1>13:1, which represent an overall 93 % selectivity. To the best of our knowledge, this is the highest reported selectivity for the chlorination of this substrate.^[27]^

We then used related conditions to evaluate the sp^3^ C−H chlorination of methylhexanoate **2**, which should mostly respond to polar factors as steric discrimination between the methylene positions is less pronounced. In general, the electron withdrawing ester group progressively deactivates the methylene units, making the ω–1 position the least deactivated towards HAT by electrophilic radicals. In this case, as all aminium radicals tested have similar electrophilicity [based on their calculated local electrophilicity indices (ω^+^_rc_)], a less dramatic variation in the reaction selectivity was observed. However, the amines **A1** and **A3** that lead to some of the slightly more electrophilic radicals, gave **4** in 84 % and 83 % ω−1 selectivity, respectively.

It is worth pointing out that during the optimization process (as well as the substrate scope) we encountered a significant challenge in the accurate determination of the reaction selectivity, especially in the case of volatile or unfunctionalized hydrocarbons. Analysis of crude mixtures by ^1^H NMR spectroscopy or gas chromatography, as done by others, proved not accurate in our hands. We therefore used a different approach and employed quantitative ^13^C NMR spectroscopy. The sensitivity of this analytical tool enabled the identification and full assignment of all reaction components even when present in very small amounts.^[28]^

### Radical Initiation

As direct photolysis of *N*‐chloroammoniums is not possible under blue light irradiation,^[29]^ we believe this radical reactivity is initiated by the adventitious generation of Cl_2_ during the *N*‐chloroamine formation step (Scheme 1 E). As Cl_2_ displays a slight absorption in the visible region,^[30]^ under our conditions Cl−Cl bond homolysis^[31]^ can take place thus starting the aminium radical‐based chain propagation.

**Scheme 3:**
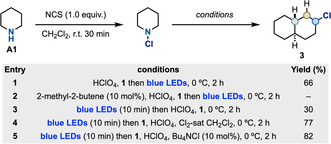
Supporting the involvement of Cl^.^.

Due to difficulties to spectroscopically detect small amounts of Cl_2_ in our reactions, we performed indirect experiments using amine **A1** and hydrocarbon **1** to support this mechanistic hypothesis (Scheme 3). (1) When the reaction was performed in the presence of the Cl_2_‐scavenger 2‐methyl‐2‐butene^[32]^ (10 mol %), functionalization of **1** was suppressed (entry 2). (2) Blue light irradiation of the *N*‐chloroamine prior to the addition of HClO_4_ and **1** also thwarted reactivity most likely because the adventitious Cl_2_ was immediately consumed in unproductive pathways (entry 3). These two experiments are in agreement with the key role of Cl^.^ to initiate the radical chain process. (3) However, chlorination was restored by simply adding Cl_2_‐saturated CH_2_Cl_2_,^[33]^ which lead to formation of **3** in similar yield and selectivity (entry 4). (4) Finally, Bu_4_NCl can generate Cl_2_ by reaction with *N*‐chloroamines^[34]^ and this is also an effective way of re‐initiating our reactions. Indeed, blue light irradiation of the *N*‐chloroamine (to destroy any adventitious Cl_2_), followed by the addition of HClO_4_, **1** and Bu_4_NCl (10 mol %) resulted in sp^3^ C−H chlorination again in similar yield and selectivity (entry 5). During the evaluation of the substrate scope, we have found the addition of Bu_4_NCl to be beneficial to improve the outcome of low yielding examples. We believe this additive aids the radical chlorination in the case of challenging substrates where chain initiation and/or propagation is not efficient. Overall, these results support the role of Cl_2_ as chain initiator and also highlight the importance in following the reaction set‐up procedure to obtain reproducible results.

### Scope of the Process

**Scheme 4:**
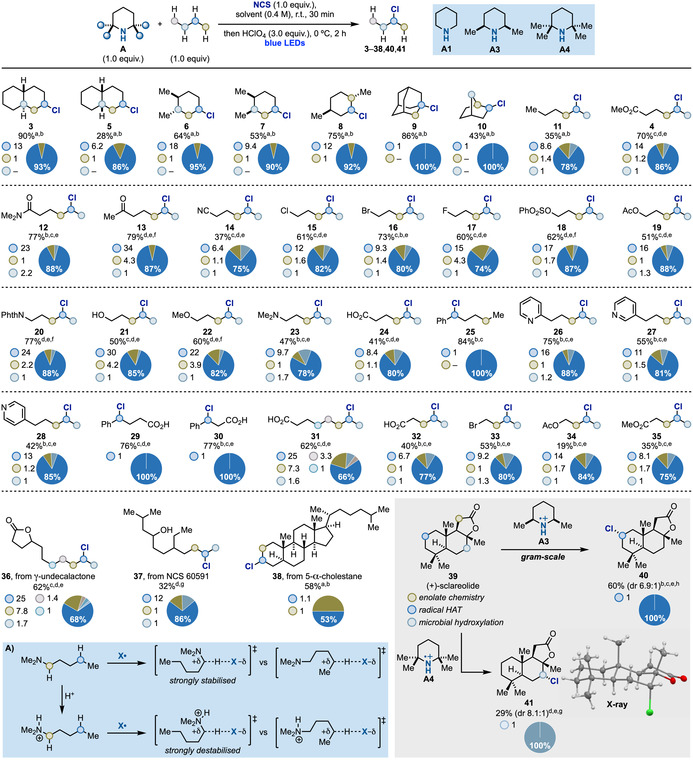
Substrate scope for the photoinduced sp^3^ C−H chlorination strategy. a) amine=**A4**; b) solvent=CH_2_Cl_2_; c amine=**A3**; d) solvent=HFIP; e) the amount of amine, NCS and acid was doubled; f) amine=**A1**; g) the reaction was performed using preformed *N*‐chlorotetramethylpiperidine;^[20]^ h) reaction run adding Bu_4_NCl (10 mol %). Phth=phthalimide.

With a set of three optimum amines, **A1**, **A3** and **A4**, we moved to evaluate the scope of the process (Scheme 4). The chlorination of unfunctionalized hydrocarbons was evaluated using bulky **A4** with the intention of maximising steric discrimination. In analogy to *trans*‐decalin **1**, *cis*‐decalin was also preferentially chlorinated at C‐2 (**5**). The decrease in yield (**5**: 28 % vs. **3**: 90 %) is probably due to the *cis*‐decalin concave shape that can sterically hamper the HAT process without affecting too much the innate selectivity (**5**: 87 % vs. **3**: 93 %). Also in this case, the tertiary position remained untouched. A similar trend of reactivity for programmable chlorination at the more accessible methylene site was observed for *trans*‐ and *cis*‐1,2‐dimethylcyclohexane (**6** and **7**). The lack of chlorination of tertiary sp^3^ C−H bonds in these examples is mechanistically relevant as it confirms that the overall selectivity is controlled by sterics. Indeed, these substrates are known mechanistic probes in HAT‐based C−H functionalization due to the operation of torsional effects. Interestingly, our approach does not target the activated tertiary equatorial C−H bond of **7** and this is in contrast with previous studies based on the reactivity of nitrogen radicals,^[11a]^ as well as dioxiranes^[35]^ and Fe/Mn‐oxo species.^[36]^ A minor functionalization of the tertiary position was observed in *trans*‐1,4‐dimethylcyclohexane (**8**) potentially owing to the more sterically encumbered nature of its methylene sites which required the use of the less hindered **A3** to improve the chemical yield. In the case of adamantane,^[37]^ the tertiary C−H bond has low steric hindrance which lead to its quantitative chlorination (**9**). This selectivity is interesting as other electrophilic radicals like *t*‐BuO^.^ and Cl^.^ usually give ≈1:1 mixture of 3° vs. 2°.^[38]^ High selectivity could also be obtained in the case of bicyclic norbornane that led to the selective formation of **10**.

Next, we explored the second set of reactions conditions identified during the optimization of **2** (see Scheme 2 B). Using a linear 5‐carbon framework, we have been able to evaluate the electronic effect of a broad range of commonly used functionalities. In all cases, high selectivity for the more reactive ω−1 position was observed even in the case of *n*‐hexane (**11**) where no electronic bias is present.

A *N*,*N*‐dimethylamide provided **12** with slightly higher selectivity compared to ester **2**. Ketone (**13**) and nitrile (**14**) functionalities were tolerated but a small decrease in the selectivity was observed. 1‐Cl‐, Br‐ and F‐pentane afforded **15**–**17** which are interesting building blocks for further functionalization^[39]^ but still represent a remarkable synthetic challenge to other methodologies.

We then evaluated other heteroatom‐based functionalities starting with various O‐ and N‐groups of different Lewis basicity and inductive power. Electron withdrawing OSO_2_Ph, OAc and NPhth (NPhth=phthalimide) groups provided the desired products (**18**–**20**) in good yield and high ω−1 selectivity [comparable (**18**) or higher (**19**, **20**) than what previously reported using other systems].^[11a]^

Substrates containing free alcohol, ether and alkylamine substituents are a known challenge in methodologies that employ electrophilic HAT reagents. Bar the free acid, these functionalities enhance reactivity at the α‐position due to hyperconjugation between the heteroatom lone‐pair and the C−H σ* orbital (Scheme 4, box A). This increases the hydridic nature of the α‐C hydrogens (BDE ≈91–95 kcal mol^−1^) and leads to preferential functionalization at this position resulting in α‐chloro‐alcohols and ‐amines that are unstable. As our reactions are run under acidic conditions, protonation (or H‐bonding) switches via polarity reversal the innate reactivity of the *sp* ^3^ C−H bonds thus deactivating the α‐methylene units towards HAT.^[40]^ This strong through‐bond effect enabled selective targeting of the ω−1 site providing **21**–**24** which cannot be achieved by previous strategies. Another manifestation of the acid‐mediated switch in the HAT site‐selectivity was observed for the functionalization of 1‐arylpentanes. In the case of 1‐phenylpentane, benzylic chlorination took place (**25**) owing to its weaker C−H bond (BDE=89 kcal mol^−1^) and the stabilization of the incipient radical. However, in the case of the corresponding 2‐, 3‐ and 4‐pyridyl derivatives, protonation converts the aromatic group into a strong deactivating substituent thus enabling selective targeting of the distal ω−1 site (**26**–**28**).

A Ph substituent is a strong activating group also in the presence of other deactivating functionalities like a free carboxylic acid as demonstrated by the successful formation of **29** and **30**.

It is important to note that in none of these examples we observed radical *sp* ^2^ C−H amination as previously reported by us and others.[^12a^, ^41^] The reaction of aminium radicals with aromatics displays strong solvent dependence and can be achieved using polar media like CH_3_CN and/or HFIP.[^12a^, ^42^] In this case, the use of CH_2_Cl_2_ as the solvent and, crucially, the absence of a photocatalyst, enabled to divert the aminium radical reactivity from π‐addition to HAT. We believe this control over chemoselectivity to be noteworthy, since pioneering laser‐flash photolysis studies by Chow,^[43]^ demonstrated how HAT reactivity is several orders of magnitudes slower than addition to π‐systems.

We were also interested in evaluating longer and shorter alkyl chain derivatives. In the case of octanoic acid, selective ω−1 chlorination was achieved (**31**), however, the remote position of the carboxylic acid leads to attenuation of the polar discrimination between the remote methylene units. Nevertheless, **31** was obtained in overall 66 % ω−1 selectivity. Shorter chain derivatives might suffer from the opposite effect as the deactivating functional group is now closer to the terminal methylene group. Despite this potential negative kinetic polar effect, chlorination of four derivatives bearing a butyl chain (**32**–**35**) was achieved in ≥75 % ω−1 selectivity and moderate to good chemical yield.

We then decided to evaluate the methodology in the late stage modification of more complex and high‐value materials. γ‐Undecalactone is an aroma compound with an intense peach flavour and its seven‐C chain was chlorinated (**36**) in overall 68 % ω−1 selectivity and good yield.

NCS 60591 is a surface‐active agent so the introduction of a Cl‐atom might infer interesting physicochemical properties.^[44]^ Pleasingly, when exposed to our reaction conditions, selective chlorination was achieved in 86 % selectivity (**37**). We believe this high ω−1 selectivity is the result of two synergistic effects, an inductive contribution from the free C‐4‐OH group and a steric one from the branching at C‐7, that progressively deactivate most of the methylene units towards HAT. A manifestation of steric deactivation of CH_2_ groups was observed in the chlorination of 5α‐cholestane, a benchmark substrate for C−H functionalization methodologies, containing 48 different *sp* ^3^ C−H bonds and no heteroatom.[^11a^, ^45^] Our chlorination process enabled selective targeting of C‐2 and C‐3 methylene units over the other 11 possible CH_2_ groups with an overall 58 % yield (**38**) which is similar to what reported by Mn^III^‐porphyrin systems.^[45a]^

The cytotoxic sesquiterpene (+)‐sclareolide (**39**) is another benchmark substrate commonly evaluated in C−H functionalization methodologies.[^11a^, ^45b^] In general, this substrate can be modified at C‐11 using enolate chemistry or at C‐2 by HAT‐based methodologies.^[36a]^ Under our optimized condition using **A3**, we obtained site‐selective chlorination at C‐2 to give 2‐chlorosclareolide (**40**) with good diastereoselectivity (dr 6.9:1) and good chemical yield on gram‐scale. Surprisingly when the bulkier **A4** was tested the unexpected and fully site‐selective chlorination at C‐7 took place (**41**), which was confirmed by X‐Ray analysis.^[46]^ To the best of our knowledge no chemical transformation has allowed the direct targeting of this position and only de novo multistep synthesis^[47]^ or microbial hydroxylation using *Cunninghamella eschinulata* ^[48]^ have been reported for the preparation of C‐7‐functionalised (+)‐sclareolide. In general, C‐2 functionalization is favored due to the planarization of an incipient radical in the HAT transition state that releases the unfavorable 1,3‐diaxial interactions with the angular Me‐groups.^[6b]^ We propose that increasing the steric hinderance of the HAT reagent diverts the site‐selectivity of the process to the less activated but more accessible C‐7 methylene unit.^[49]^ While unexpected this result represent an outstanding example of reagent‐dictated site‐selectivity and suggests that by screening structurally different aminium radicals, the complex topology of natural products might open up for novel and selective functionalization paradigms.

## Conclusion

Achieving selectivity in radical sp^3^ C−H chlorination is still a synthetic challenge especially when broad functional group compatibility is required. In this article we have reported the development of a photochemical protocol for the efficient and site‐selective assembly of chlorinated building blocks. This strategy harnesses the conversion of secondary amines into aminium radicals that are powerful intermediates in HAT processes. The high‐electrophilicity of these species, combined with the ease of modulating the steric hinderance around their N‐centre has allowed to synergistically maximise polar and steric factors in the HAT‐chlorination manifold.

Overall, this reactivity has enabled the direct introduction of chlorine atoms in place of sp^3^ C−H bonds with often the highest known site‐selectivity. The process tolerates a broad range of functionalities that are frequently elusive in other radical approaches thus providing access to high‐value building blocks for further chemical diversification. The possibility to override the innate selectivity for C−H functionalization by changing the steric environment around the aminium radical might enable the exploration of currently elusive chemical space.

## Conflict of interest

The authors declare no conflict of interest.

## Supporting information

As a service to our authors and readers, this journal provides supporting information supplied by the authors. Such materials are peer reviewed and may be re‐organized for online delivery, but are not copy‐edited or typeset. Technical support issues arising from supporting information (other than missing files) should be addressed to the authors.

Supplementary
