# Supplementary material for: Practical and Selective sp3 C−H Bond Chlorination via Aminium Radicals
Source: Angew Chem Int Ed Engl. 2021 Feb 25;60(13):7132–9. doi: 10.1002/anie.202100030 (PMC8048631; doi:10.1002/anie.202100030)
Supplement: Supplementary file 1 — Supplementary [file ANIE-60-7132-s001.pdf]

Angewandte Chemie
Eine Zeitschrift der Gesellschaft Deutscher Chemiker

Supporting Information

Practical and Selective sp^3 C–H Bond Chlorination via Aminium Radicals

Alastair J. McMillan, Martyna Sieńkowska, Piero Di Lorenzo, Gemma K. Gransbury, Nicholas F. Chilton, Michela Salamone, Alessandro Ruffoni, Massimo Bietti,* and Daniele Leonori**

anie_202100030_sm_miscellaneous_information.pdf

Table of contents

1 General Experimental Details 4

2 Starting Material Syntheses 5

4 Reaction Set-Up 8

5 ^{13}C Relaxation Experiments 9

6 Assignment of ^{13}C NMR Spectra 10

7 Reaction Optimisation 11

7.1 General Procedure for the Reaction Optimisation Using *trans*-Decalin – GP1 11

7.2 General Procedure for the Reaction Optimisation Using Methyl Hexanoate – GP2 13

7.3 Control Experiments 15

8 Quantum Yield Determination 16

9 Radical Initiation Experiments 17

10 N-Chlorination of Amines 19

11 General Procedures for the Reaction Scope 20

12 Substrate Scope 22

12.1 Chlorination of *trans*-decalin to give 3 22

12.2 Chlorination of *cis*-Decalin to Give 5 25

12.3 Chlorination of 1,2-*anti*-dimethylcyclohexane to give 6 28

12.4 Chlorination of 1,2-*syn*-dimethylcyclohexane to give 7 31

12.5 Chlorination of 1,4-*anti*-dimethylcyclohexane to give 8 33

12.6 Chlorination of Adamantane to Give 9 36

12.7 Chlorination of Norbornane to Give 10 38

12.8 Chlorination of *n*-Hexane to Give 11 41

12.9 Chlorination of Methyl Hexanoate to Give 4 43

12.10 Chlorination of *N*,*N*-Dimethylhexanamide to Give 12 45

12.11 Chlorination of Heptan-2-one to Give 13 47

12.12 Chlorination of Hexanenitrile to Give 14 49

12.13 Chlorination of 1-Chloropentane to Give 15 51

12.14 Chlorination of 1-Bromopentane to Give 16 53

12.15 Chlorination of 1-Fluoropentane to Give 17 55

12.16 Chlorination of Pentyl Benzenesulfonate to Give 18 57

12.17 Chlorination of Pentyl Acetate to Give 19 59

12.18 Chlorination of 2-Pentylisoindoline-1,3-dione to Give 20 61

12.19 Chlorination of Pentan-1-ol to give 21 63

12.20 Chlorination of 1-Methoxypentane to Give 22 65

12.21 Chlorination of *N*,*N*-Dimethylpentylamine to Give 23 67
12.22 Chlorination of Hexanoic Acid to Give 24 69
12.23 Chlorination of Pentylbenzene to Give 25 71
12.24 Chlorination of 2-Pentylpyridine to Give 26 73
12.25 Chlorination of 3-Pentylpyridine to Give 27 75
12.26 Chlorination of 4-Pentylpyridine to Give 28 77
12.27 Chlorination of 4-Phenylbutanoic Acid to Give 29 79
12.28 Chlorination of 3-Phenylpropanoic Acid to Give 30 81
12.29 Chlorination of Octanoic Acid to Give 31 83
12.30 Chlorination of Pentanoic Acid to Give 32 85
12.31 Chlorination of 1-Bromobutane to Give 33 87
12.32 Chlorination of Butyl Acetate to Give 34 89
12.33 Chlorination of Methyl Pentanoate to Give 35 91
12.34 Chlorination of γ-Undelactone to Give 36 93
12.35 Chlorination of NCS60591 to give 37 96
12.36 Chlorination of 5α-Cholestane to Give 38 99
12.37 Chlorination of (+)-Sclareolide to Give 40 102
12.38 Chlorination of (+)-Sclareolide to Give 41 105
13 Gram-scale Reactions 109
14 X-Ray Analysis of Compound 41 110
15 Computational Studies 112
15.1 General Details 112
15.2 %VSA Values 113
15.3 Correlation Between %VSA and Site-Selectivity for the Chlorination of *trans*-Decalin 115
15.4 Optimised geometries for aminium radicals 116
15.5 Determination of Electrophilicity Indices 126
16 NMR spectra 127
17 References 134

1 General Experimental Details

All required chemicals were used directly without purification unless stated otherwise. All air or moisture sensitive reactions were carried out under nitrogen atmosphere using standard Schlenk manifold technique. All dry solvents were bought from Acros as 99.8% purity. ^{1}H and ^{13}C Nuclear Magnetic Resonance (NMR) spectra were acquired at various field strengths as indicated and were referenced to $CHCl_3$ (7.27 and 77.0 ppm for ^{1}H and ^{13}C respectively) unless otherwise specified. Quantitative ^{13}C NMRs were obtained using a Bruker Avarice III instrument with an Oxford AS600 magnet equipped with liquid helium cryroprobe [5mm CPDCH ^{13}C-^{1}H/D]. Each scan was allotted an acquisition time of 2 minutes to ensure full relaxation of the sample. C-Cl peaks in carbon spectra were identified via the distinctive isotopic effect of ^{35}Cl/^{37}Cl on the adjacent carbon with confirmation from literature or HSQC where necessary.[1] These peaks were then assigned based on literature or HSQC data or via comparison with the spectra of other, closely related, molecules which have literature or HSQC data. ^{1}H NMR coupling constants are reported in Hertz and refer to apparent multiplicities and not true coupling constants. Data is reported as follows: chemical shift, integration, multiplicity (s = singlet, br s = broad singlet, d = doublet, t = triplet, q = quartet, p = pentet, sx = sextet, sp = septet, m = multiplet, dd = doublet of doublets, etc.), proton assignment (determined by 2D NMR experiments: COSY, HSQC and HMBC) where possible. GC-MS analysis was carried out using an AGILENT 7820A-GC and 5975-MS. Spectra were obtained using electron impact ionization (EI) or, if this was not possible, by APCI. Analytical TLC: aluminum backed plates pre-coated (0.25 mm) with Merck Silica Gel 60 F254. Compounds were visualized by exposure to UV-light, by exposure to iodine or by dipping the plates in permanganate (KMnO4), phosphomolybdic acid (PMA), Ceric ammonium molybdate (CAM) or p-Anisaldehyde stain followed by heating (whichever worked best). Flash column chromatography was performed using Merck Silica Gel 60 (40–63 µm). All mixed solvent eluents are reported as v/v solutions. The LEDs used are Kessil PR160 440 nm. All the reactions were conducted in CEM 10 mL glass microwave tubes.

2 Starting Material Syntheses

2-Pentylisoindoline-1,3-dione (S1)

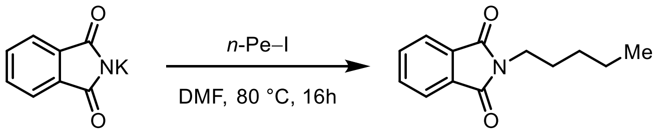

A solution of iodopentane (1.3 mL, 10 mmol, 1.0 equiv.) in DMF (100 mL) was charged with potassium phthalamide (3.7 g, 20 mmol, 2.0 equiv.). The mixture was warmed to 80 °C, stirred overnight and then cooled to r.t. The mixture was diluted with Et_2O (40 mL) and washed with water (8 x 40 mL). The organic layer was dried ($MgSO_4$) and evaporated. Purification by flash column chromatography on silica gel eluting *n*-hexane-EtOAc (4:1) gave **S1** as an oil (1.84 g, 85 %). ^{1}H NMR: (300 MHz, $CDCl_3$) δ 7.87–7.80 (2H, m), 7.73–7.68 (2H, m), 3.67 (2H, t, *J* = 7.2 Hz), 1.74–1.60 (2H, m), 1.42–1.23 (4H, m), 0.88 (3H, t, *J* = 6.8 Hz); ^{13}C NMR (126 MHz, $CDCl_3$) δ 168.6, 134.0, 132.3, 123.3, 38.2, 29.1, 28.4, 22.4, 14.1. Data in accordance with the literature.[2]

Pentyl Benzenesulfonate (S2)

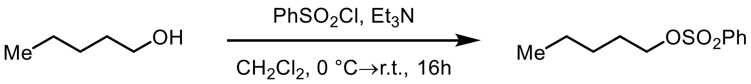

A solution of pentanol (2.6 mL, 24 mmol, 1.1 equiv.) and Et_3N (4.0 mL, 29 mmol, 1.3 equiv.) in CH_2Cl_2 (25 mL) was cooled to 0 ºC and treated with benzene sulfonyl chloride (2.8 mL, 22 mmol, 1 equiv.). The reaction was warmed to r.t. overnight, diluted with Et_2O (40 mL) and washed with 1M HCl (2 x 40 mL) and brine (40 mL). The organic layer was dried ($MgSO_4$), filtered then evaporated. Purification by flash column chromatography on silica gel eluting with *n*-exane–EtOAc (95:5→9:1) gave **S2** as an oil (3.4 g, 68 %). ^{1}H NMR (400 MHz, $CDCl_3$) δ 7.91 (2H, d, *J* = 7.6 Hz), 7.65 (1H, t, *J* = 7.4 Hz), 7.56 (2H, t, *J* = 7.6 Hz), 4.04 (2H, t, *J* = 6.5 Hz), 1.64 (2H, p, *J* = 6.8 Hz), 1.33–1.19 (4H, m), 0.84 (3H, t, *J* = 6.5 Hz); ^{13}C NMR (126 MHz, $CDCl_3$) δ 136.4, 133.8, 129.3, 128.0, 71.1, 28.7, 27.6, 22.2, 14.0. Data in accordance with the literature.[2]

N,N-Dimethylpentan-1-amine (S3)

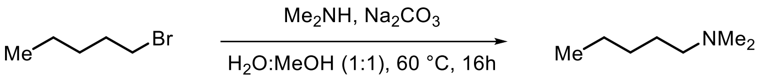

A suspension of Na_2CO_3 (4.2 g, 40 mmol, 2.0 equiv.) in MeOH (20 mL) was treated with Et_2NH (5.1 mL, 40 mmol, 2.0 equiv., 40 % (w/w) solution in H_2O) and 1-bromopentane (2.5

mL, 20 mmol, 1.0 equiv.). The mixture was heated to 60 °C, stirred overnight and cooled to r.t. The mixture was diluted with pentane (20 mL) and washed with water (5 x 20 mL). The organic layer was dried ($MgSO_4$) and **S3** was purified by fractional distillation (0.52 g, 23%). ^{1}H NMR (500 MHz, $CDCl_3$) δ 2.25–2.21 (2H, m), 2.21 (6H, s), 1.45 (2H, p, *J* = 7.5 Hz), 1.29 (4H, m), 0.89 (3H, t, *J* = 7.1 Hz); ^{13}C NMR (126 MHz, $CDCl_3$) δ 60.1, 45.7, 29.9, 27.6, 22.8, 14.2. Data in accordance with the literature.[3]

3-Pentylpyridine (S4)

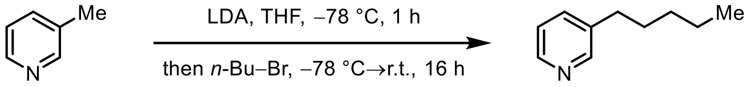

A solution of *i*-Pr_2NH (2.2 mL, 15.5 mmol, 1.55 equiv.) in anhydrous THF (45 mL) was cooled to –78 ºC and treated with *n*-BuLi (9.4 mL, 15 mmol, 1.5 equiv., 1.6 M in hexanes). A solution of 3-picoline (0.97 mL, 10 mmol, 1 equiv.) in anhydrous THF (5 mL) was then added by dropwise the mixture was stirred for 1 h at –78 °C. A solution of 1-bromobutane (1.1 mL, 10 mmol, 1.0 equiv.) in anhydrous THF (5 mL) was added by dropwise and the mixture was warmed to r.t. overnight. The mixture was diluted with NH_4Cl sat. (40 mL) and extracted with EtOAc (3 x 40 mL). The combined organic layers were dried ($MgSO_4$), filtered and evaporated. Purification by flash column chromatography on silica gel eluting *n*-hexane–EtOAc (4:1)gave **S4** as an oil (0.75 g, 50%). ^{1}H NMR (500 MHz, $CDCl_3$) δ 8.44 (1H, d, *J* = 2.3), 8.42 (1H, dd, *J* = 4.8, 1.6), 7.48 (1H, dt, *J* = 7.8, 2.0), 7.19 (1H, dd, *J* = 7.8, 4.8), 2.59 (2H, t, *J* = 7.8 Hz), 1.62 (2H, p, *J* = 7.4), 1.39–1.27 (4H, m), 0.89 (3H, t, *J* = 6.9); ^{13}C NMR ($CDCl_3$, 126 MHz) δ 150.1, 147.3, 138.1, 135.9, 123.3, 33.1, 31.5, 31.0, 22.3, 14.1. Data in accordance with the literature.[4]

4-Pentylpyridine (S5)

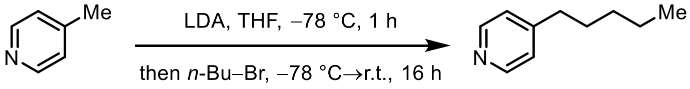

A solution of *i*-Pr_2NH (2.2 mL, 15.5 mmol, 1.55 equiv.) in anhydrous THF (45 mL) was cooled to –78 ºC and treated with *n*-BuLi (9.4 mL, 15 mmol, 1.5 equiv., 1.6 M in hexanes). A solution of 4-picoline (1.0 mL, 10 mmol, 1 equiv.) in anhydrous THF (5 mL) was then added by dropwise the mixture was stirred for 1 h at –78 °C. A solution of 1-bromobutane (1.1 mL, 10 mmol, 1.0 equiv.) in anhydrous THF (5 mL) was added by dropwise and the mixture was warmed to r.t. overnight. The mixture was diluted with NH_4Cl sat. (40 mL) and extracted with EtOAc (3 x 40 mL). The combined organic layers were dried ($MgSO_4$), filtered and evaporated.

Purification by flash column chromatography on silica gel eluting EtOAc-Hexane (1:4) gave **S5** as an oil (0.3 g, 2 mmol, 20 %). ^{1}H NMR ($CDCl_3$, 500 MHz) δ 8.49–8.46 (2H, m), 7.11–7.08 (2H, m), 2.62–2.56 (2H, m), 1.63 (2H, p, J = 7.5 Hz), 1.33 (4H, m), 0.89 (3H, t, J = 6.9 Hz); ^{13}C NMR ($CDCl_3$, 126 MHz) δ 151.9, 149.8, 124.1, 35.4, 31.48, 30.1, 22.6, 14.1. Data in accordance with the literature.[5]

1-Methoxypentane (S6)

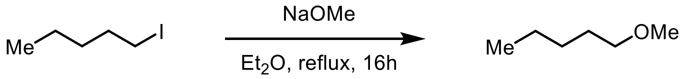

A solution of iodopentane (2.6 mL, 20 mmol, 1.0 equiv.) in Et_2O (10 mL) was treated with NaOMe (9.1 mL, 40 mmol, 2.0 equiv., 25 % in MeOH), heated under reflux and stirred overnight. The mixture was cooled to r.t., diluted with pentane (10 mL) and extracted with H_2O (5 x 20 mL) then dried ($MgSO_4$) and filtered. **S6** was purified by fractional distillation (662 mg, 6.5 mmol, 32 %). ^{1}H NMR ($CDCl_3$, 500 MHz) δ 3.36 (2H, t, J = 6.7 Hz), 3.33 (3H, s), 1.61–1.53 (2H, m), 1.37–1.27 (4H, m), 0.94–0.86 (3H, m); ^{13}C NMR ($CDCl_3$, 126 MHz) δ 73.1, 58.7, 29.5, 28.5, 22.7, 14.2. Data in accordance with the literature.[6]

1-Chloro-2,2,6,6-tetramethylpiperidine (S7)

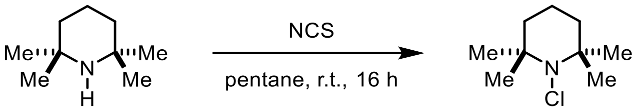

A suspension of NCS (2.9 g, 21 mmol, 1.2 equiv.) in pentane (40 mL) was treated with TMP (3 mL, 18 mmol, 1.0 equiv.) and the mixture was stirred overnight. The solid residues were filtered and the mixture was evaporated. **S7** was purified by fractioned distillation under reduced pressure (2.1 g, 66%). ^{1}H NMR ($CDCl_3$, 400 MHz) δ 1.65–1.59 (4H, m), 1.58–1.50 (2H, m), 1.23 (12H, s); ^{13}C NMR ($CDCl_3$, 126 MHz) δ 62.7, 40.8, 27.5, 17.4. Data in accordance with the literature.[7]

4 Reaction Set-Up

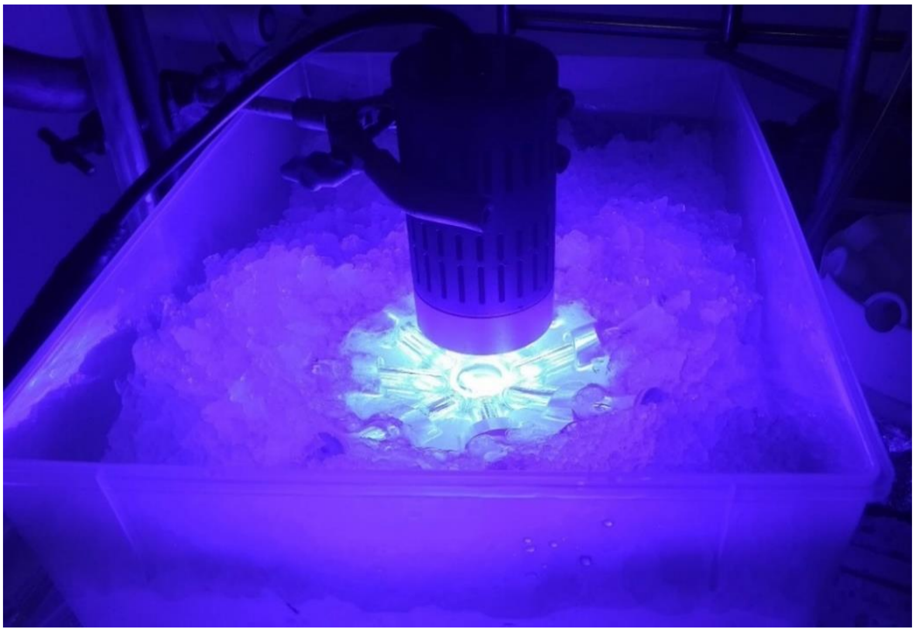

Figure S1.

The reactions were run by irradiating the tubes whilst maintaining them at 0 °C using an ice bath. The water level was maintained so that it covered the tubes at all times. The area between the light source and the tubes was kept clear of ice and a stirrer bar was used to maintain an even temperature. The stirring rate for the ice bath and the reactions was 500 rpm. The light source (Kessil PR160 440 nm) was placed approximately 5 cm away from the tubes. The tubes were held by a custom-made aluminium parallel reaction holder.

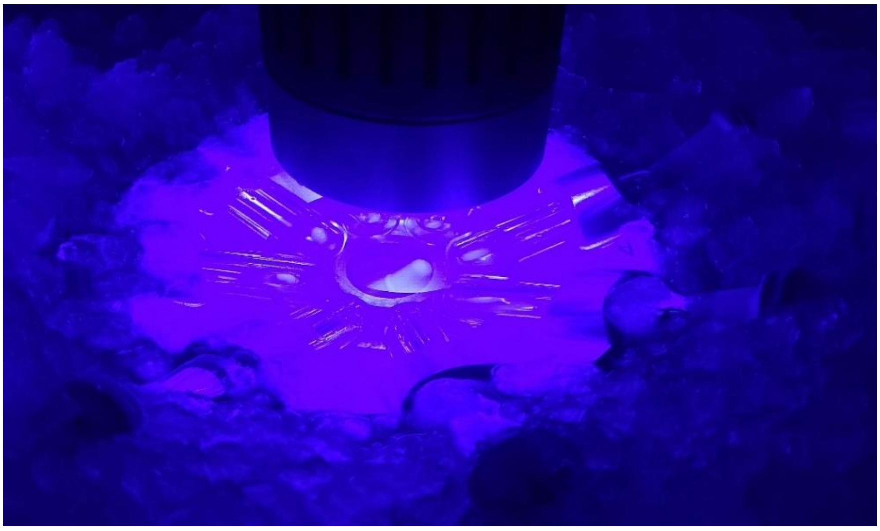

Figure S2.

5 ^{13}C Relaxation Experiments

Approximate values for the ^{13}C NMR T1 constants of the nuclear spins in molecules were acquired via inversion recovery experiments. Recovery delays between 375 ms to 48 s were used and the T1 values calculated from the peak pitting of intensities. The samples were run in non-degassed $CDCl_3$ at room temperature.

1,3-Dinitrobenzene

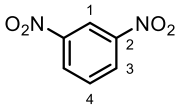

Carbon environment	5T1 Value (s)
1	24
2	168
3	19
4	25

These results demonstrate that the carbons corresponding to environments 1, 3 and 4 should easily be relaxed within the quantitative ^{13}C NMR timescale used (117 s) and therefore that their integrals are suitable for use to determine the reaction yield.

1-chlorocyclohexane

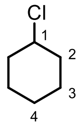

Carbon environment	5T1 Value (s)
1	76
2	46
3	47
4	39

6 Assignment of ^{13}C NMR Spectra

^{1}H NMR spectroscopy could sometimes not be used to determine the reactions selectivity due to peak overlaps. In these cases, the ^{1}H NMR spectra were used as a rough indication only and they are included for completeness. The reaction yields and selectivity have been determined by quantitative ^{13}C NMR spectroscopy.[8] Carbons adjacent to Cl-atoms can be identified in ^{13}C NMR spectra by a distinctive shoulder corresponding to the 3:1 ratio of ^{35}Cl:^{37}Cl isotopes (see Figure S3 for an example from a real reaction crude).[1] When necessary, comparison with literature value and HSQC techniques have been used for full assignment of different isomers.

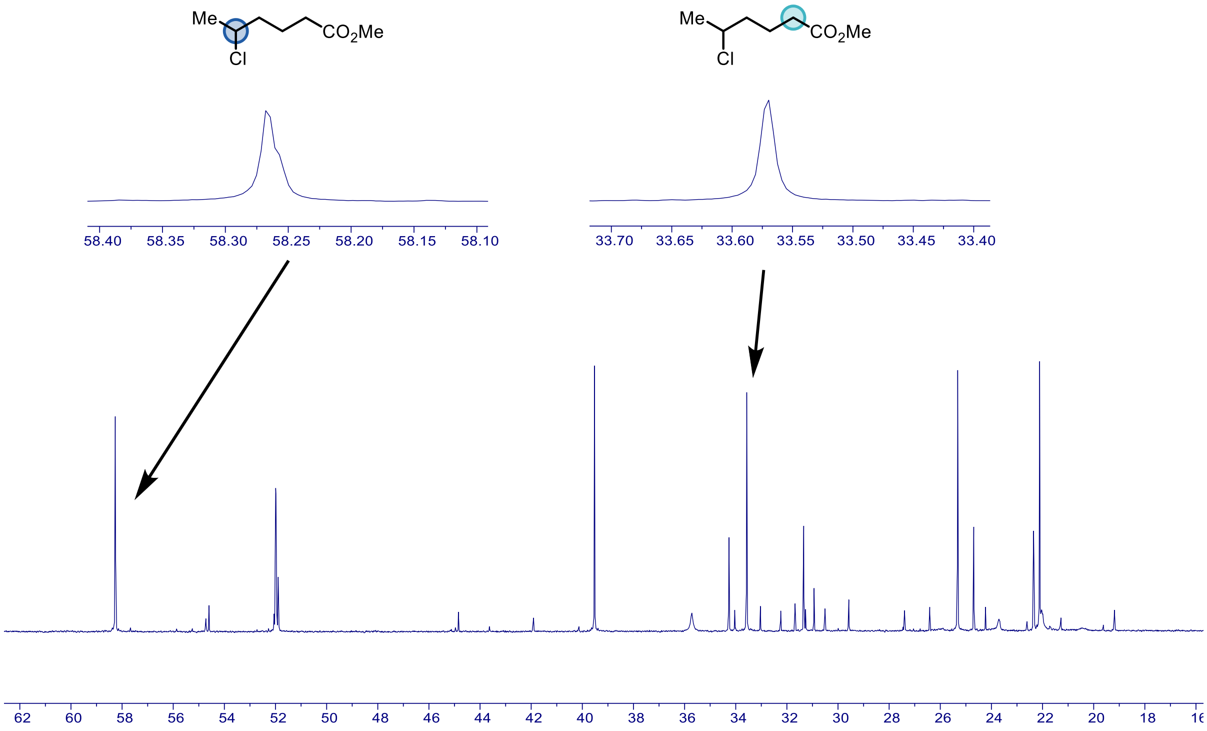

Figure S3.

7 Reaction Optimisation

7.1 General Procedure for the Reaction Optimisation Using *trans*-Decalin – GP1

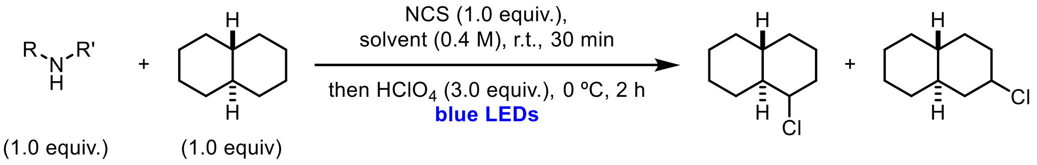

A tube equipped with a stirring bar was charged with NCS (27 mg, 0.2 mmol, 1.0 equiv.) and the amine (0.2 mmol, 1.0 equiv.) if solid. The tube was capped with a Supelco aluminium crimp seal with septum (PTFE/butyl), evacuated and refilled with N_2 (x 3). CH_2Cl_2 (0.5 mL, 0.4 M) (degassed by bubbling through with N_2 for 20 min.) and the amine (0.2 mmol, 1.0 equiv.) if liquid were added and the mixture was stirred for 30 min at r.t. (*it is important to minimise direct exposure to visible light at this stage*). The mixture was cooled to 0 °C and stirred at this temperature for 10 minutes. The mixture was treated with *trans*-decalin (32 μL, 0.2 mmol, 1.0 equiv.) and $HClO_4$ (52 μL, 0.6 mmol, 3.0 equiv., 70 % in H_2O). The blue LEDs were immediately switched on and the mixture was stirred under irradiation for 2 h at 0 °C. The mixture was warmed to r.t. and 1,3-dinitrobenzene (0.25 equiv., 0.1 M solution in $CDCl_3$) and H_2O (4 mL) were added. The layers were separated and the organic phase was passed through a short pad of $MgSO_4$ (anhydrous) directly into an NMR tube for analysis by ^1H NMR and ^{13}C NMR spectroscopy.

Table 1

Entry	Amine	Yield (%)	C-2:C-1 Selectivity	C-2 Selectivity (%)
**1**	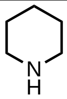	68	1.9:1	66
**2**	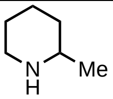	80	3.1:1	76
**3**	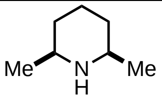	92	2.6:1	72
**4**	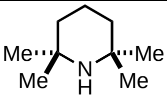	90	13.3:1	93
**5**	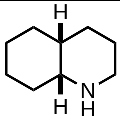	85	2.7:1	73
**6**	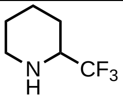	51	2.0:1	67
**7**	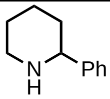	37	1.4:1	58
**8**	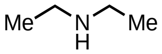	67	2.8:1	74
**9**	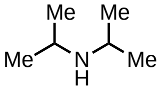	80	3.1:1	76
**11**	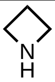	42	1.8:1	64
**12**	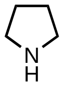	47	2.0:1	67
**13**	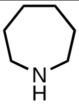	46	2.0:1	67
**14**	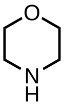	47	1.6:1	62

7.2 General Procedure for the Reaction Optimisation Using Methyl Hexanoate – GP2

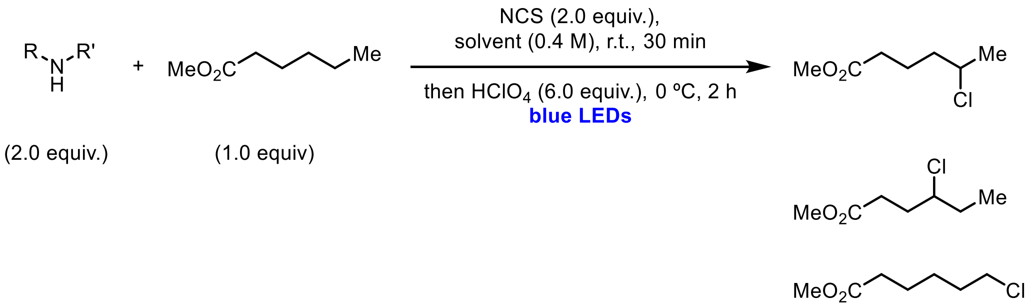

A tube equipped with a stirring bar was charged with NCS (53.4 mg, 0.4 mmol, 2.0 equiv.) and the amine (0.2 mmol, 1.0 equiv.) if solid. The tube was capped with a Supelco aluminium crimp seal with septum (PTFE/butyl), evacuated and refilled with N_2 (x 3). CH_2Cl_2 (0.5 mL, 0.4 M) (degassed by bubbling through with N_2 for 20 min.) and the amine (0.2 mmol, 1.0 equiv.) if liquid were added and the mixture was stirred for 30 min at r.t. (*it is important to minimise direct exposure to visible light at this stage*). The mixture was cooled to 0 °C and stirred at this temperature for 10 minutes. The mixture was treated with methyl hexanoate (29 μL, 0.2 mmol, 1.0 equiv.) and $HClO_4$ (104 μL, 1.2 mmol, 6.0 equiv., 70 % in H_2O). The blue LEDs were immediately switched on and the mixture was stirred under irradiation for 2 h at 0 °C. The mixture was warmed to r.t. and 1,3-dinitrobenzene (0.25 equiv., 0.1 M solution in $CDCl_3$) and H_2O (4 mL) were added. The layers were separated and the organic phase was passed through a short pad of $MgSO_4$ (anhydrous) directly into an NMR tube for analysis by ^1H NMR and ^{13}C NMR spectroscopy.

Table 2

Entry	Amine	Yield (%)	ω–1:ω–2:ω	ω–1 Selectivity (%)
**1**	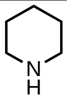	43	18:2.5:1.0	84
**2**	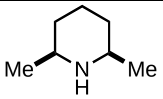	52	11:1.3:1.0	83
**3**	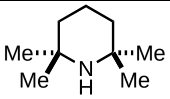	43	2.9:0.9:1.0	60
**4**	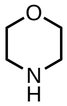	59	9.0:1.8:1.0	76
**5**	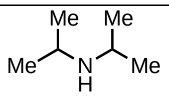	59	6.6:0.9:1.0	78

7.3 Control Experiments

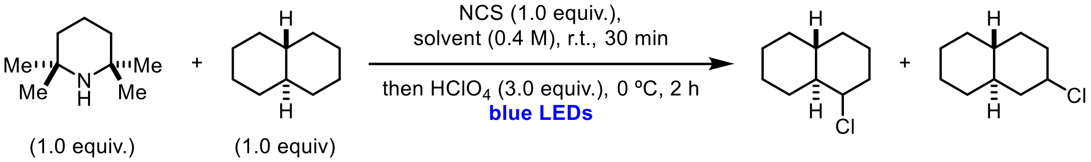

Control experiments were performed according to **GP1** using TMP as amine and CH_2Cl_2 as solvent.

Table 3

Entry	Deviation from Standard Conditions	Yield (%)	C-2:C-1 Selectivity
**1**	–	90	13.3:1
**2**	no light	6	6.1:1
**3**	no acid	0	–
**4**	no amine	12	1.2:1

8 Quantum Yield Determination

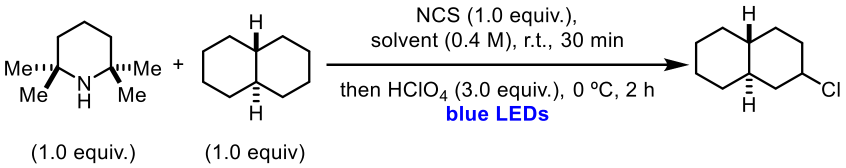

The quantum yield of the photochemical reactions was determined twice at 298 K following procedures described in literature.[9] The degassed reaction tubes were irradiated using blue LED (λ_{max} = 444 nm) as the light source for 5 and 10 min. The yield of products was determined by ^{1}H NMR spectroscopy using 1,3-dinitrobenzene as the internal standard. The photon flux of the blue LEDs used was determined by standard ferrioxalate actinometry.[10] The results are consistent with a process based on a radical-chain propagation.

Table 4.

Entry	Reaction time (min)	Yield (%)	Φ
**1**	5	16	4.8
**2**	10	46	6.9

9 Radical Initiation Experiments

During the formation of the *N*-chloroamines, we suspect a small amount of Cl_2 is formed. Following blue light irradiation latent Cl_2 might lead to the formation of Cl• that can initiate the reactivity.[11,12]

To prove the involvement of Cl• in the initiation step we performed the following experiments.

1. Standard chlorination.

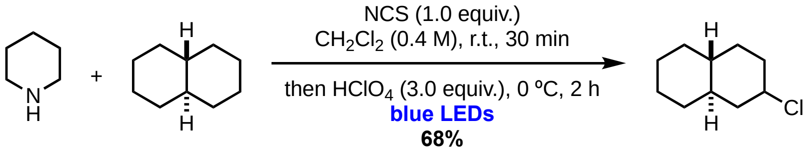

2. When the reaction was run in the presence of 2-methyl-2-butene (5 mol%), which is a known Cl• scavenger[13], no product formation was obtained.

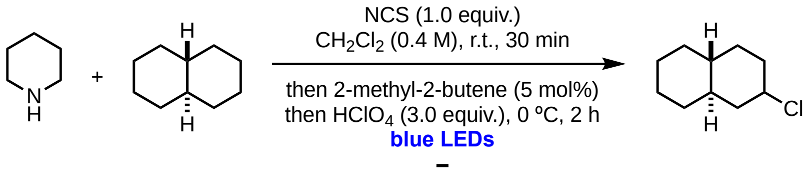

The presence of chlorination of 2-methyl-2-butene was confirmed by the identification of the following products by GC-MS analysis of the reaction mixtures.

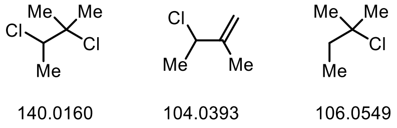

3. When the reaction was irradiated immediately after the formation of the *N*-chloropiperidine, followed by addition of *trans*-decalin and $HClO_4$, low product was obtained. This is consistent with the photochemical decomposition of Cl_2 prior to substrate addition, which should thwart reaction initiation.

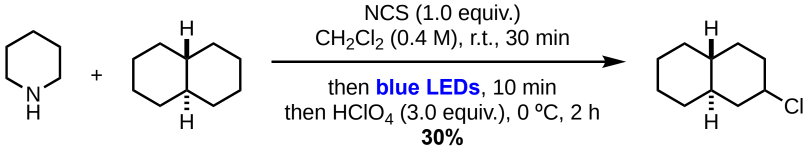

4. To further demonstrate that Cl_2 photolysis hampers the reactions productivity we have added exogenous Cl_2 as a saturated solution in CH_2Cl_2[14] to the previous reaction. This provided a marginal increase in yield.

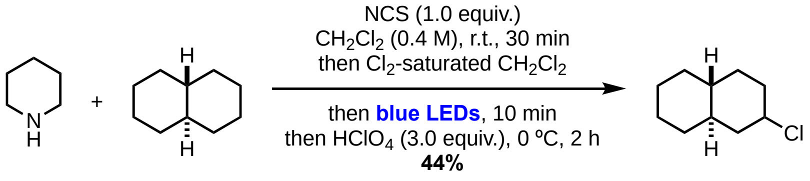

5. When the reaction was irradiated immediately after the formation of the *N*-chloropiperidine, followed by addition of exogenous Cl_2 as a saturated solution in CH_2Cl_2 and then *trans*-decalin and $HClO_4$, high product conversion was obtained. This is consistent with the initiation of the reaction by Cl_2.

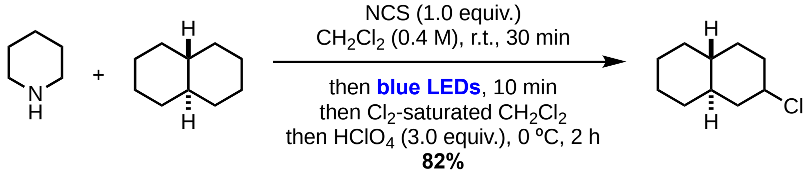

6. A more practical way of ensuring the presence of Cl_2 to initiate the reaction was the addition of *tetra*-butylammonium chloride after light irradiation, followed by *trans*-decalin and $HClO_4$. Also in this case, high product conversion was obtained.

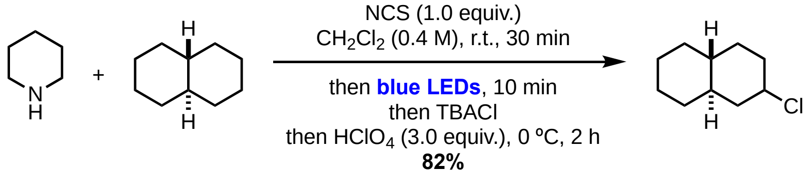

Preparation of Cl_2-Saturated Solution in CH_2Cl_2

Chlorine gas was generated by slowly adding concentrated HCl (100 mL, 37% in H_2O) to NaOCl (300 mL, 8% active chlorine) under N_2 pressure, the resulting gas was bubbled through CH_2Cl_2 (100 mL) using a cannula for 20 minutes until the solution turned a bright yellow colour.

10 N-Chlorination of Amines

The *N*-chlorination of amines is achieved by treatment with NCS in CH_2Cl_2 at room temperature. This reaction is generally quantitative and therefore NCS can be used in equimolar amounts. This avoids the presence of unwanted reactivity based on succinimidyl radical.

These reactions can be easily monitored by ^{1}H NMR spectroscopy as shown below for the N-chlorination of piperidine.

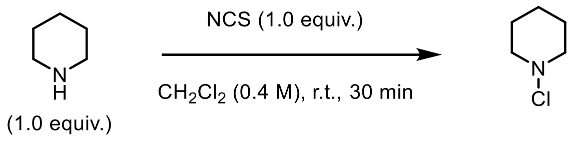

Spectra of the starting amine and the reaction mixture 30 minutes after addition of 1.0 equiv. of NCS demonstrate the complete disappearance of the amine peaks and the corresponding appearance of those of the *N*-chloropiperidine (Figure S4).

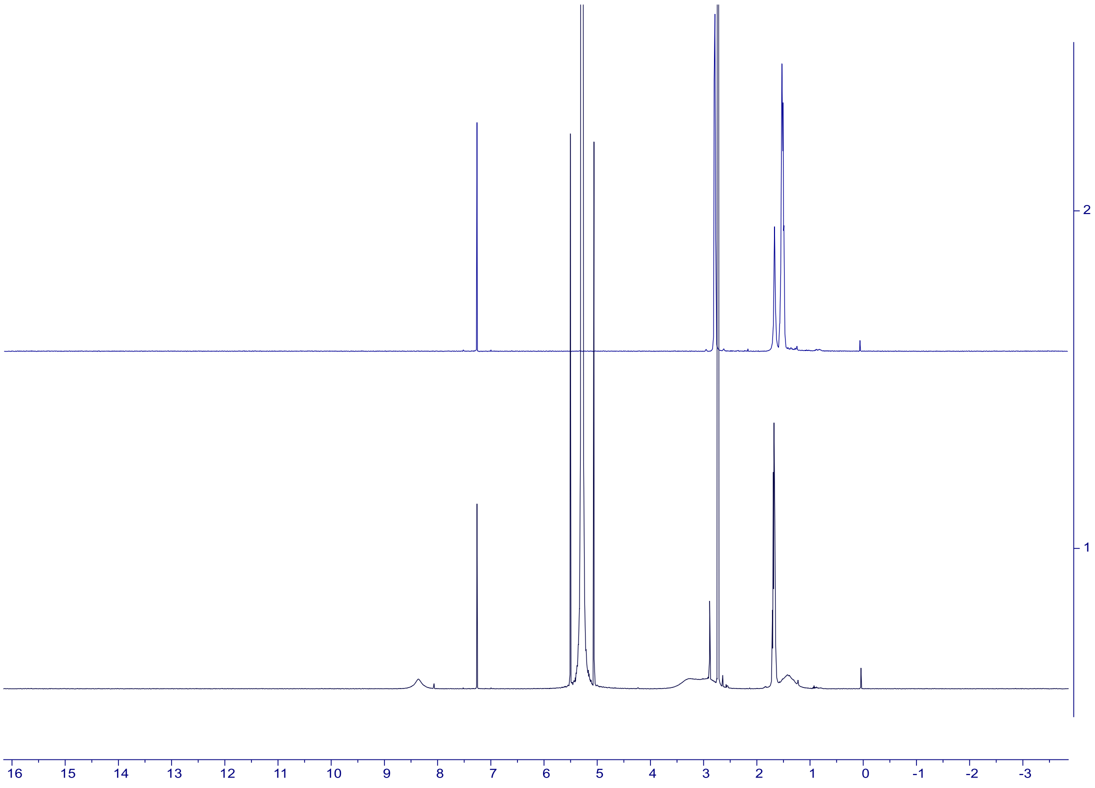

Figure S4.

11 General Procedures for the Reaction Scope

General Procedure for the Chlorination of Functionalised Alkanes ─ GP3

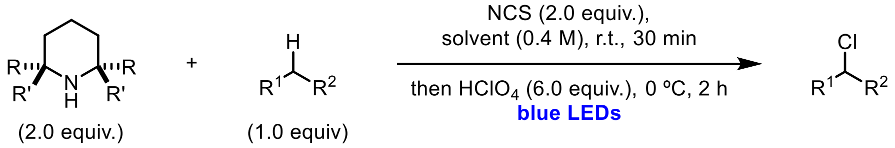

A tube equipped with a stirring bar was charged with NCS (53.4 mg, 0.4 mmol, 2.0 equiv.), capped with a Supelco aluminium crimp seal with septum (PTFE/butyl), evacuated and refilled with N_2 (x 3). The solvent (0.5 mL, 0.4 M) (degassed by bubbling through with N_2 for 20 min.) and the amine (0.4 mmol, 2.0 equiv.) were added and the mixture was stirred for 30 min at room temperature (*it is important to minimise direct exposure to visible light at this stage*). The reaction mixture was cooled to 0 °C and stirred at this temperature for 10 minutes. The mixture was treated with the substrate (0.1 mmol, 1.0 equiv., in the case of solid substrates, they were added as a 2 M solution in the same reaction solvent) and $HClO_4$ (105 µL, 1.2 mmol, 6.0 equiv., 70 % in H_2O). The blue LEDs were immediately switched on and the mixture was stirred under irradiation for 2 h at 0 °C. The mixture was warmed to r.t. and 1,3-dinitrobenzene (8.4 mg, 0.25 equiv., 0.1 M solution in $CDCl_3$) and H_2O (4 mL) were added. The layers were separated and the organic phase was passed through a short pad of $MgSO_4$ (anhydrous) directly into an NMR tube for analysis by ^{1}H and ^{13}C NMR spectroscopy.

General Procedure for the Chlorination of Unfunctionalised Alkanes ─ GP4

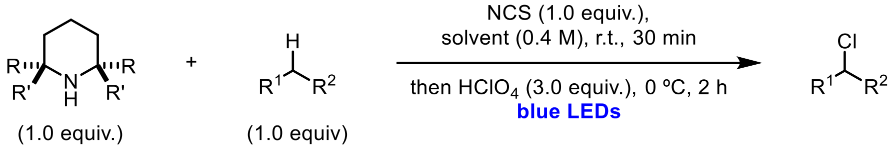

A tube equipped with a stirring bar was charged with NCS (0.2 mmol, 1.0 equiv.), capped with a Supelco aluminium crimp seal with septum (PTFE/butyl), evacuated and refilled with N_2 (x 3). The solvent (0.5 mL, 0.4 M) (degassed by bubbling through with N_2 for 20 min.) and the amine (0.2 mmol, 1.0 equiv.) were added and the mixture was stirred for 30 min at room temperature (*it is important to minimise direct exposure to visible light at this stage*). The reaction mixture was cooled to 0 °C and stirred at this temperature for 10 minutes. The mixture was treated with the substrate (0.1 mmol, 1.0 equiv., in the case of solid substrates, they were added as a 2 M solution in the same reaction solvent) and $HClO_4$ (52 µL, 0.6 mmol, 3.0 equiv., 70 % in H_2O). The blue LEDs were immediately switched on and the mixture was stirred under

irradiation for 2 h at 0 °C. The mixture was warmed to r.t. and 1,3-dinitrobenzene (0.25 equiv., 0.1 M solution in $CDCl_3$) and H_2O (4 mL) were added. The layers were separated and the organic phase was passed through a short pad of $MgSO_4$ (anhydrous) directly into an NMR tube for analysis by ^1H and ^{13}C NMR spectroscopy.

Amines used:

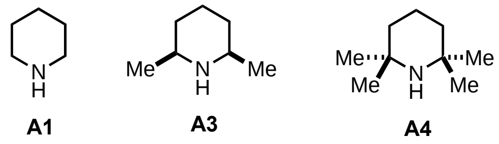

12 Substrate Scope

12.1 Chlorination of *trans*-decalin to give 3

2-Chloro-*trans*-decahydronaphthalene (3)

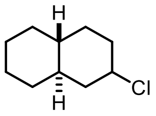

Starting Material	Observed Reaction Products
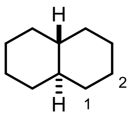	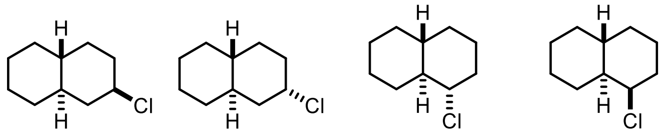 3a 3b 3'a 3'b
**C-2:C-1 (3a:3b:3'a:3'b)**	**C-2 HAT Selectivity (%)**
13:1.0 (18:13:1.5:1.0)	93

Following **GP4** using **A4** as the amine and CH_2Cl_2 as the solvent, *trans*-decalin (32 µL, 0.2 mmol) gave **3** and **3'** in 67% overall yield. GC-MS m/z (EI): 172.1 (M+).

3a: Data in accordance with the literature.[15]

3b: Data in accordance with the literature.[15]

3'a: Data in accordance with the literature.[15]

3'b: Data in accordance with the literature.[15]

^{1}H NMR (600 MHz, $CDCl_3$)

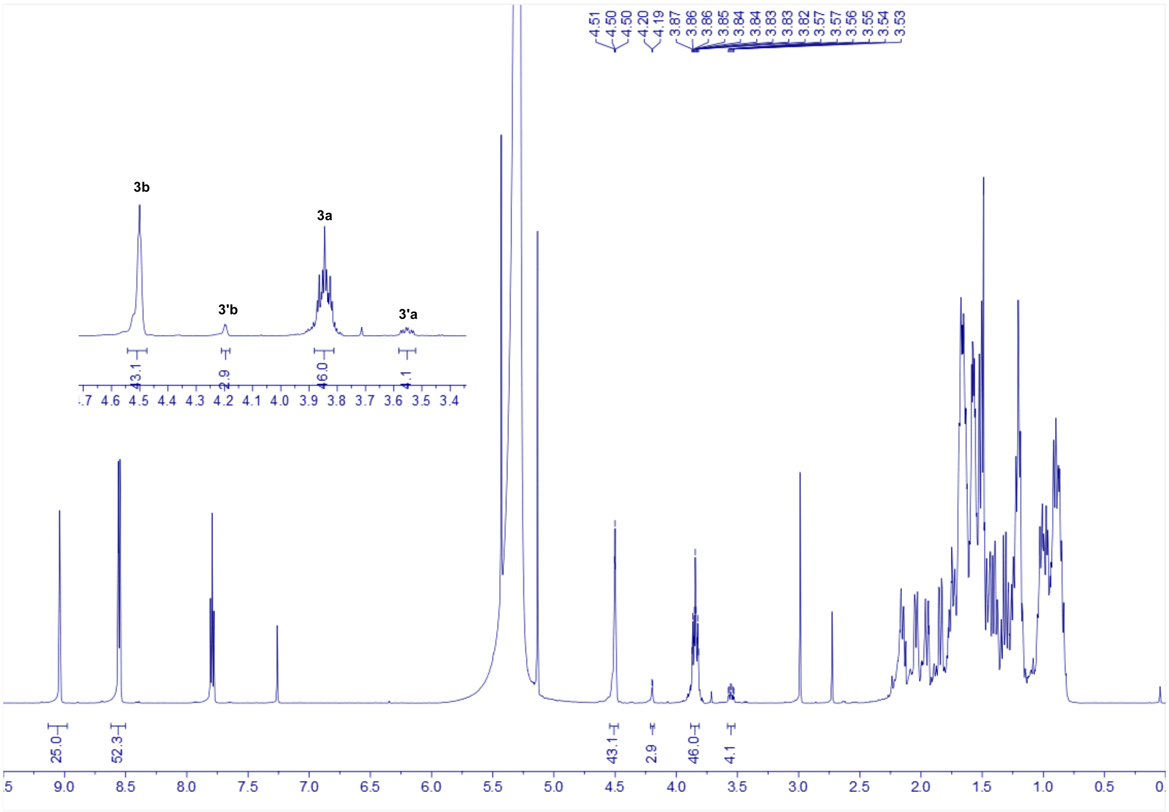

^{13}C Quantitative NMR (151 MHz, $CDCl_3$)

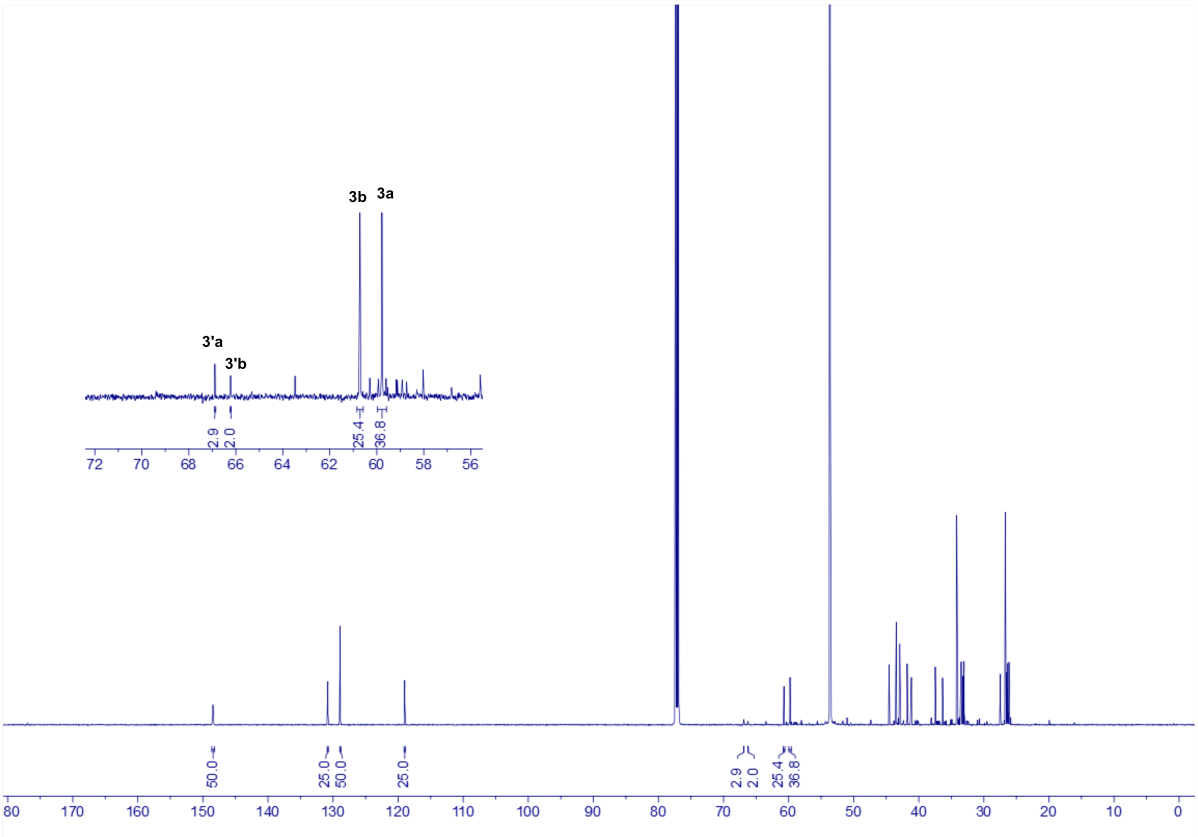

HSQC (400 MHz, $CDCl_3$)

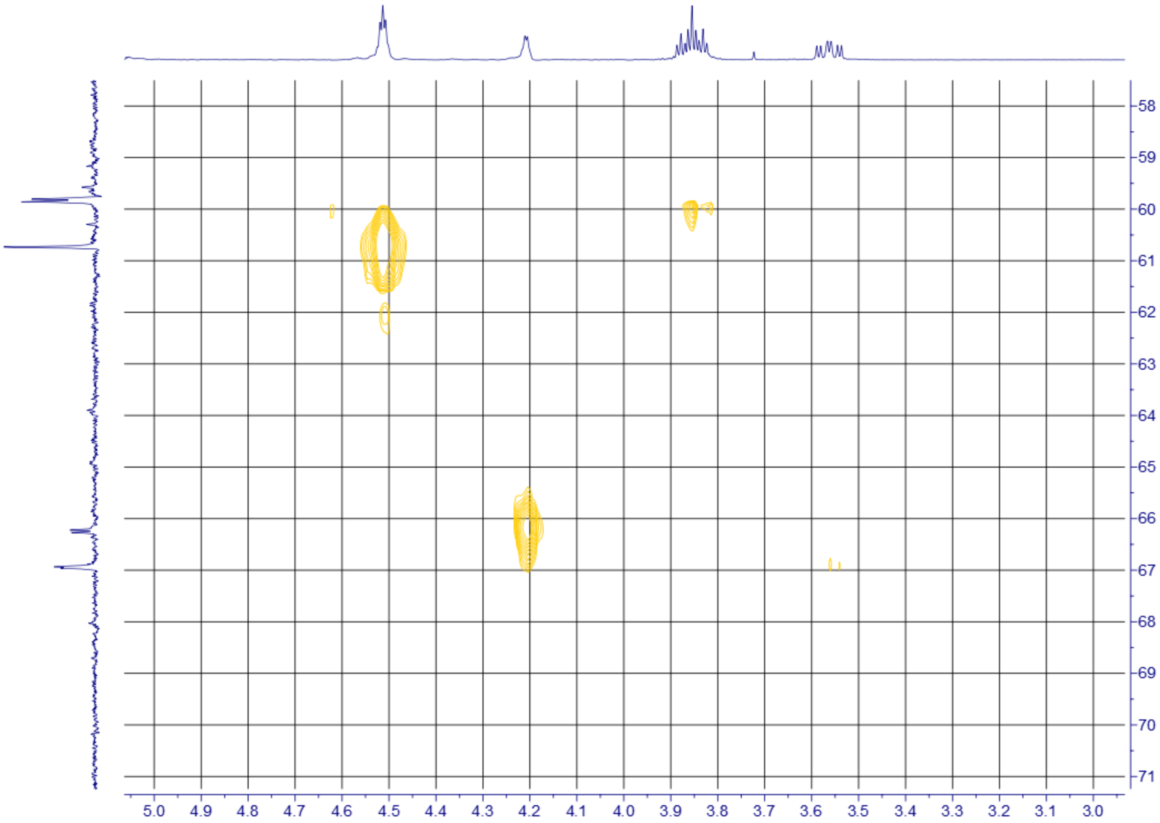

12.2 Chlorination of *cis*-Decalin to Give 5

2-Chloro-*cis*-decahydronaphthalene (5)

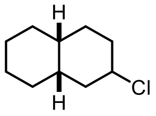

Starting Material	Observed Reaction Products
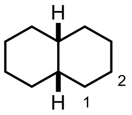	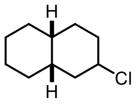 **5** 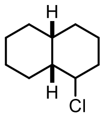 **5'**
**C-2:C-1**	**C-2 HAT Selectivity (%)**
6.2:1.0	86

Following **GP4** using **A4** as the amine and CH_2Cl_2 as the solvent, *cis*-decalin (31 μL, 0.2 mmol) gave **5** and **5'** in 28% yield. GC-MS m/z (EI): 172.1 (M+).

5: Data in accordance with the literature.[16]

5': Data in accordance with the literature.[16]

^{1}H NMR (600 MHz, $CDCl_3$)

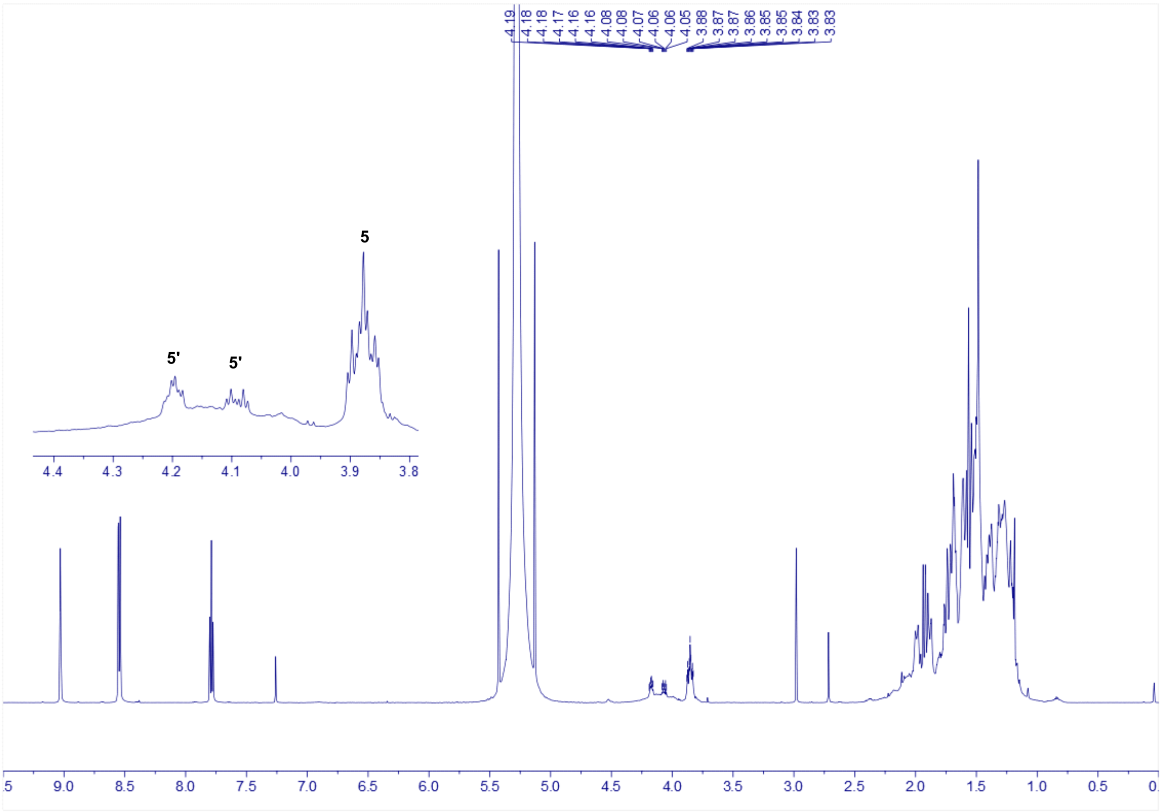

^{13}C Quantitative NMR (151 MHz, $CDCl_3$)

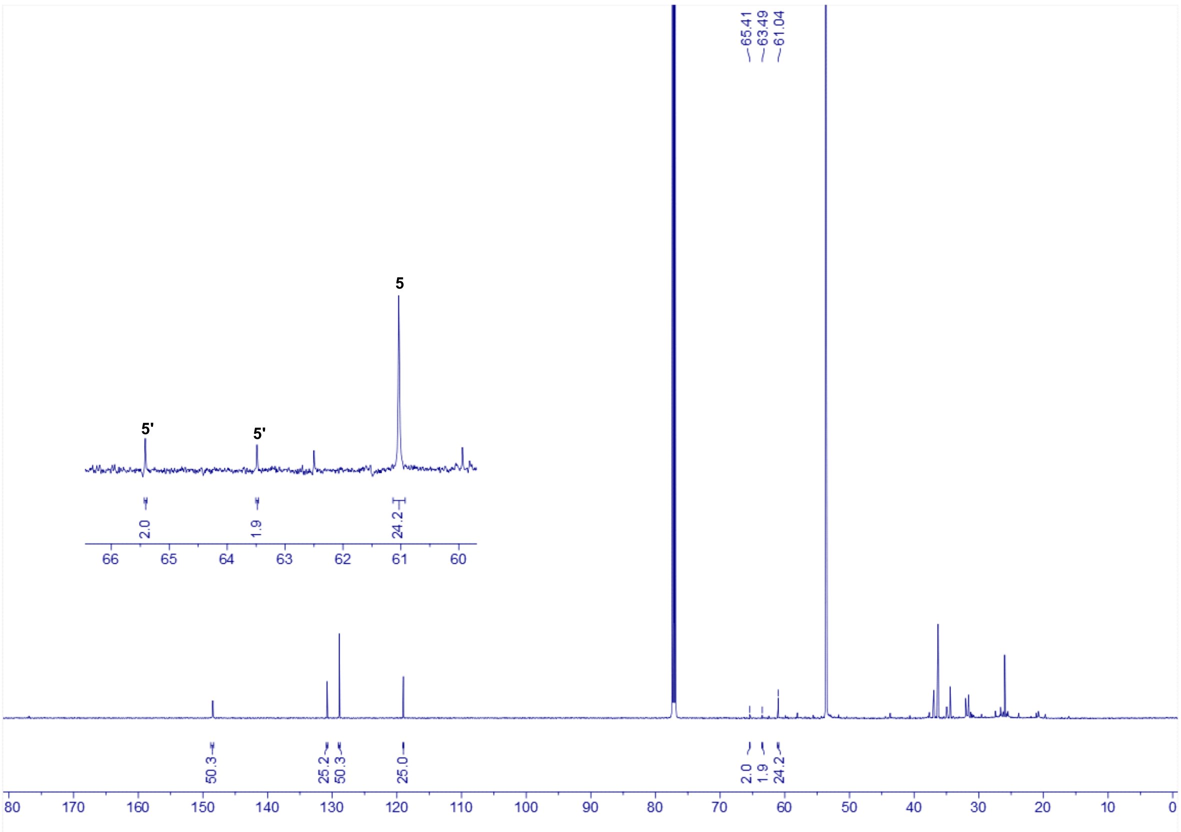

HSQC (400 MHz, $CDCl_3$)

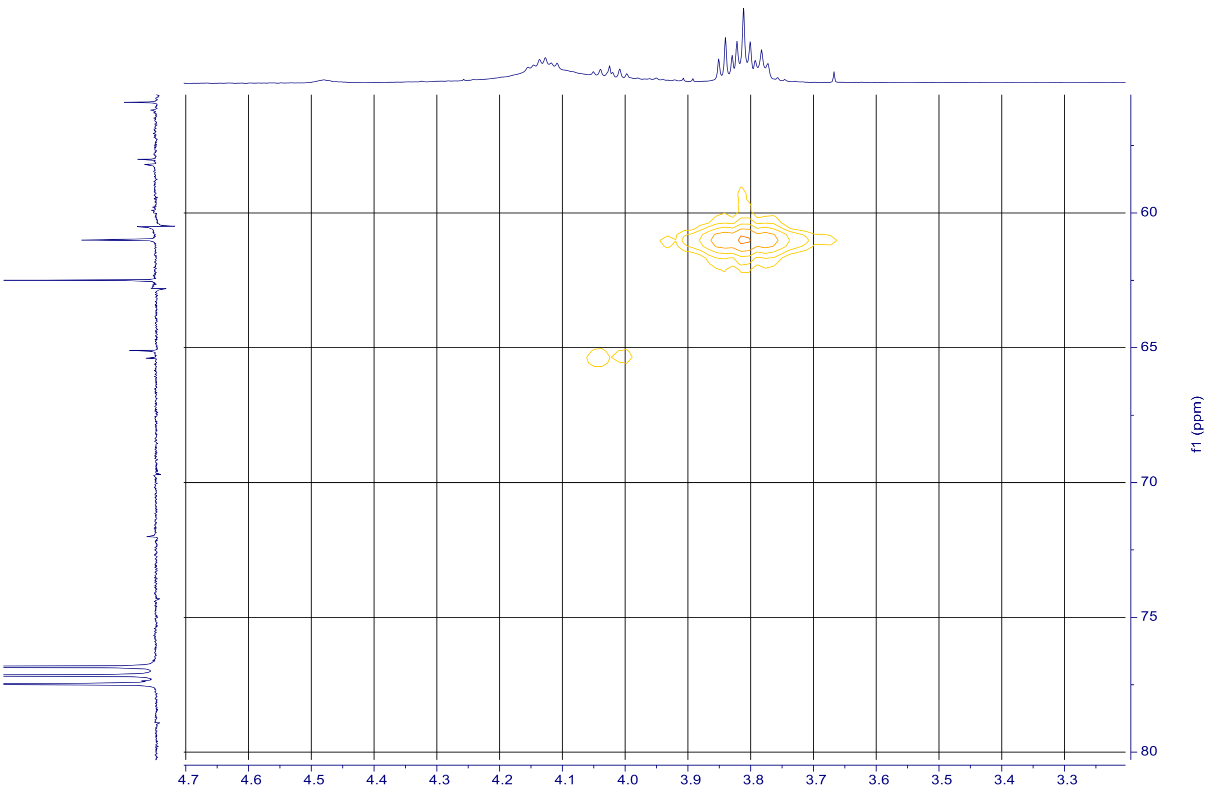

12.3 Chlorination of 1,2-*anti*-dimethylcyclohexane to give 6

4-Chloro-1,2-*anti*-dimethylcyclohexane (6)

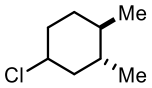

Starting Material	Observed Reaction Products			
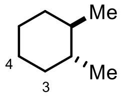	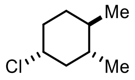 **6a**	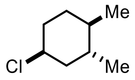 **6b**	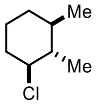 **6'a**	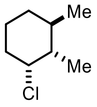 **6'b**
**C-4:C-3 (6a:6b:6'a:6'b)**	**C-4 HAT Selectivity (%)**			
18:1 (27:24:1.8:1.0)	95			

Following **GP4** using **A4** as the and CH_2Cl_2 as the solvent, *trans*-1,2-dimethylcyclohexane (29 μL, 0.2 mmol) gave **6** and **6'** in 64% yield. GC-MS m/z (EI): 146.1 (M+).

6a, **6b**, **6'a**, **6'b**: ^{1}H NMR structural assignments based on *J* constants; ^{13}C NMR structural assignments based on HSQC. HSQC data acquired also from an experiment with **A3** as the amine in order to increase the proportion of the minor isomers and enable better signal monitoring.

^{1}H NMR (600 MHz, $CDCl_3$)

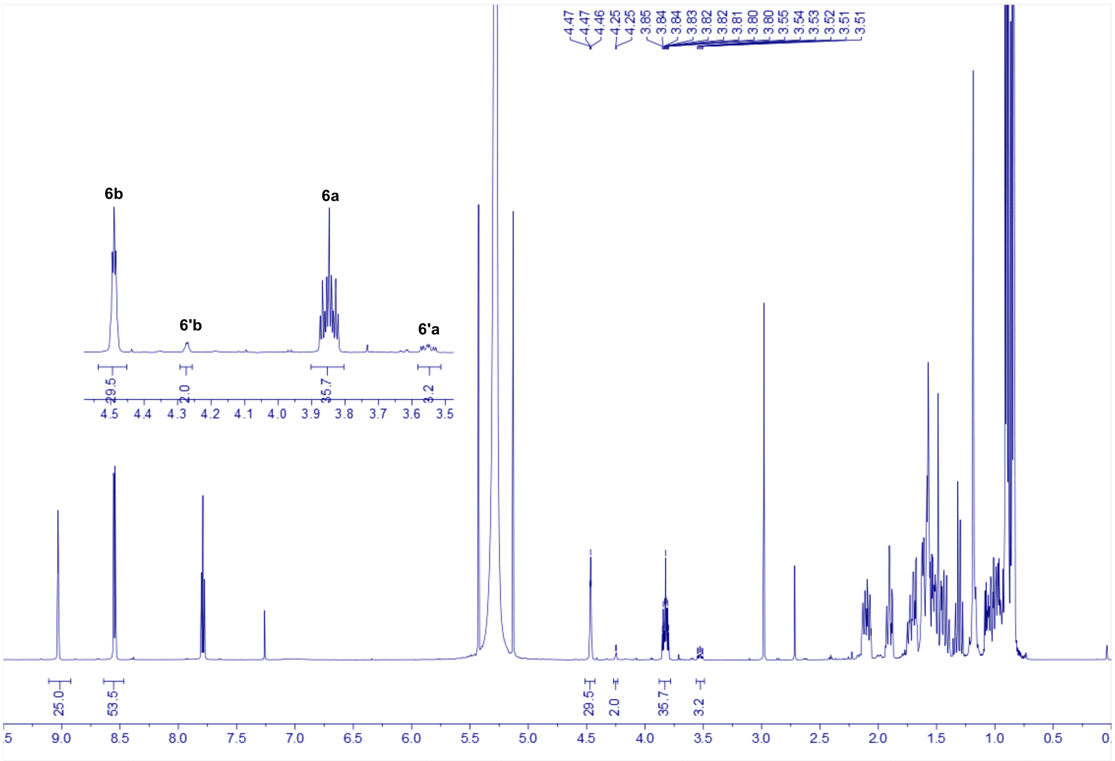

^{13}C Quantitative NMR (151 MHz, $CDCl_3$)

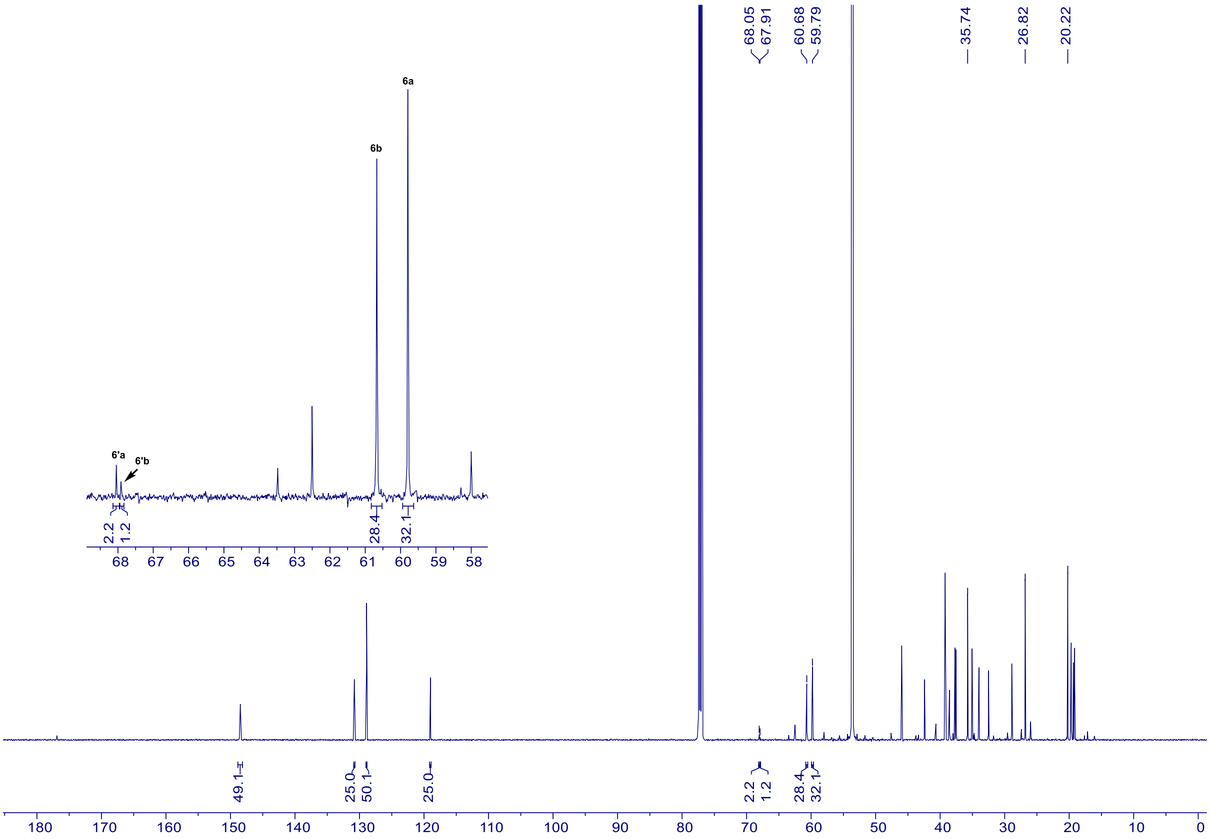

HSQC (400 MHz, $CDCl_3$)

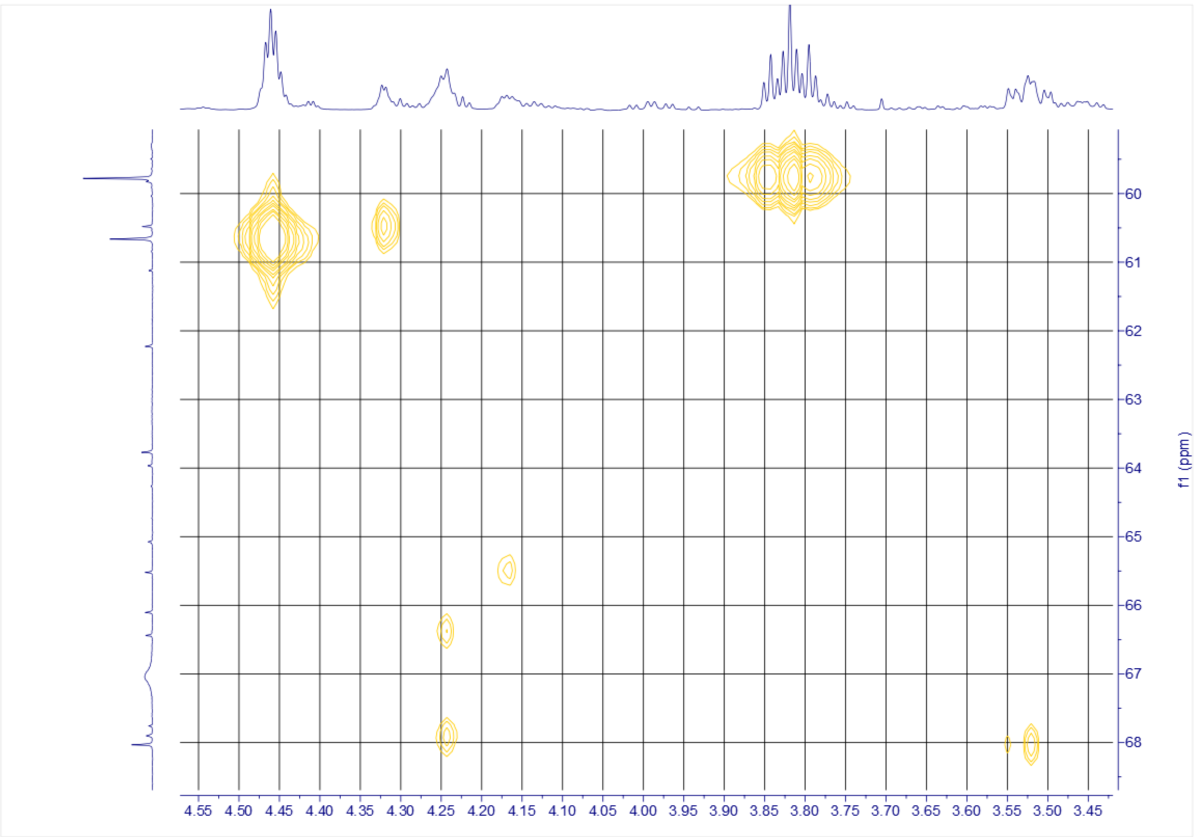

12.4 Chlorination of 1,2-*syn*-dimethylcyclohexane to give 7

4-Chloro-1,2-*syn*-dimethylcyclohexane (7)

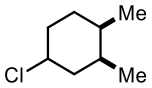

**Starting Material**	**Observed Reaction Products**
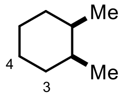	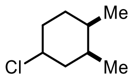 **7** 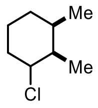 **7'**
**C-4:C-3**	**C-4 HAT Selectivity (%)**
9.4:1	90

Following **GP4** using **A4** as the amine and CH_2Cl_2 as the solvent, *cis*-1,2-dimethylcyclohexane (28 μL, 0.2 mmol) gave **7** and **7'** in 53% yield. GC-MS m/z (EI): 146.1 (M+).

7, 7': ^{1}H NMR structural assignments based on *J* constants.

^{1}H NMR (400 MHz, $CDCl_3$)

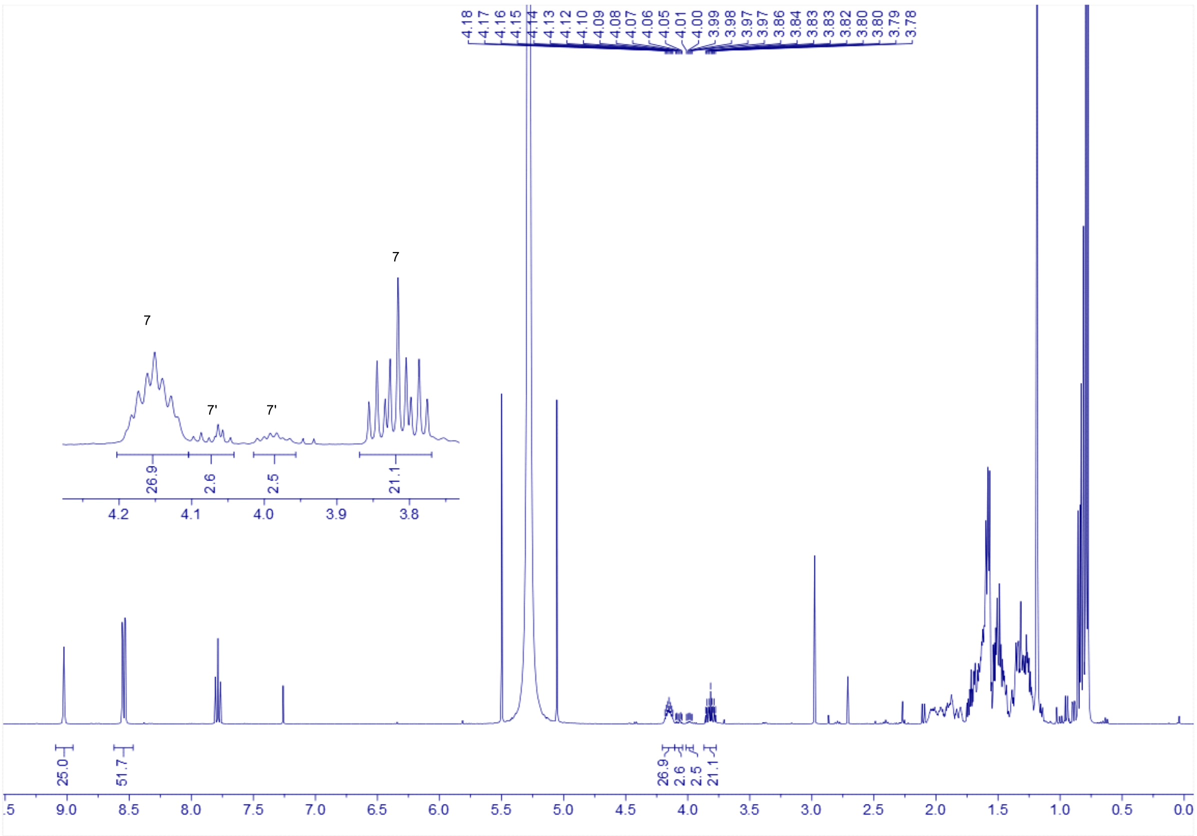

12.5 Chlorination of 1,4-*anti*-dimethylcyclohexane to give 8

2-Chloro-1,4-*anti*-dimethylcyclohexane (8)

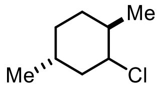

Starting Material	Observed Reaction Products
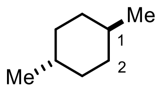	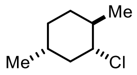 **8a** 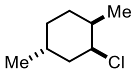 **8b** 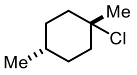 **8'**
**C-1:C-2 (8a:8b:8')**	**C-2 HAT Selectivity (%)**
12:1 (7.2:5.0:1.0)	92

Following **GP4** using **A3** as the amine and CH_2Cl_2 as the solvent, *trans*-1,4-dimethylcyclohexane (29 µL, 0.2 mmol) gave **8** and **8'** in 75% yield. GC-MS m/z (EI): 146.1 (M+).

8a, **8b**: ^{1}H NMR structural assignments based on J constants; ^{13}C NMR structural assignments based on HSQC.

8': ^{13}C NMR structural assigned based on literature data for 1-bromo-1,4-*anti*-dimethylcyclohexane and distinctive $^{35/37}$Cl carbon peak shoulder.[17]

^{1}H NMR (600 MHz, $CDCl_3$)

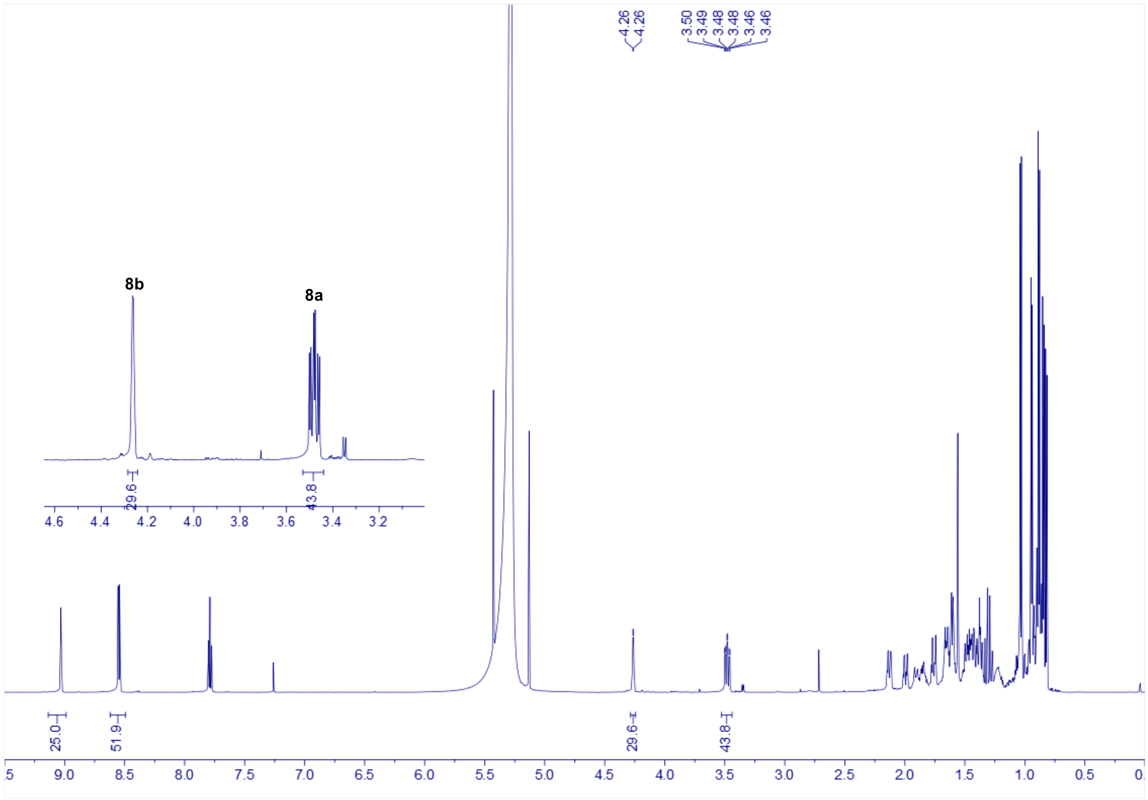

^{13}C Quantitative NMR (151 MHz, $CDCl_3$)

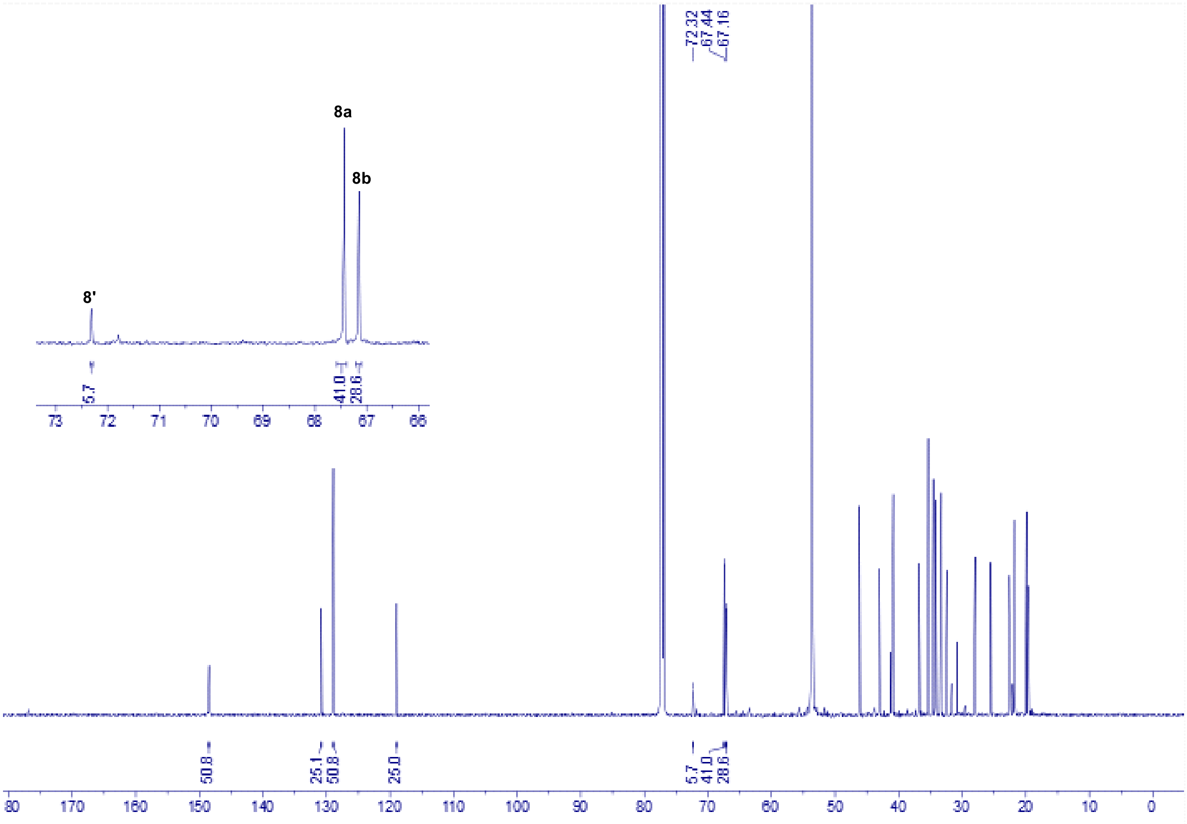

HSQC (400 MHz, $CDCl_3$)

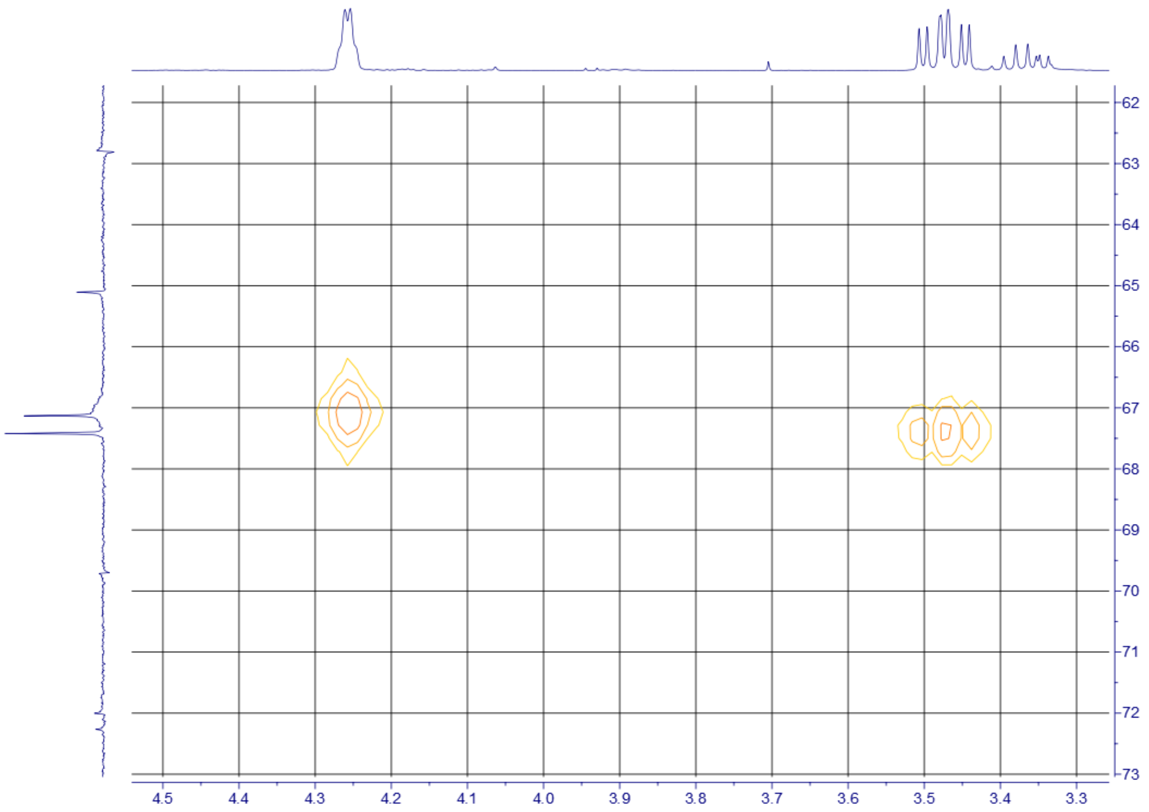

12.6 Chlorination of Adamantane to Give 9

1-Chloroadamantane (9)

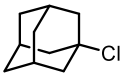

Starting Material	Observed Reaction Products	C-1 HAT Selectivity (%)
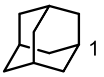	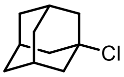 **9**	100

Following **GP4** using **A4** as the amine and CH_2Cl_2 as the solvent, adamantane (27 mg, 0.2 mmol) gave **9** in 86% yield. GC-MS m/z (EI): 170.1 (M+).

9: Data in accordance with the literature.[18]

^{1}H NMR (600 MHz, $CDCl_3$)

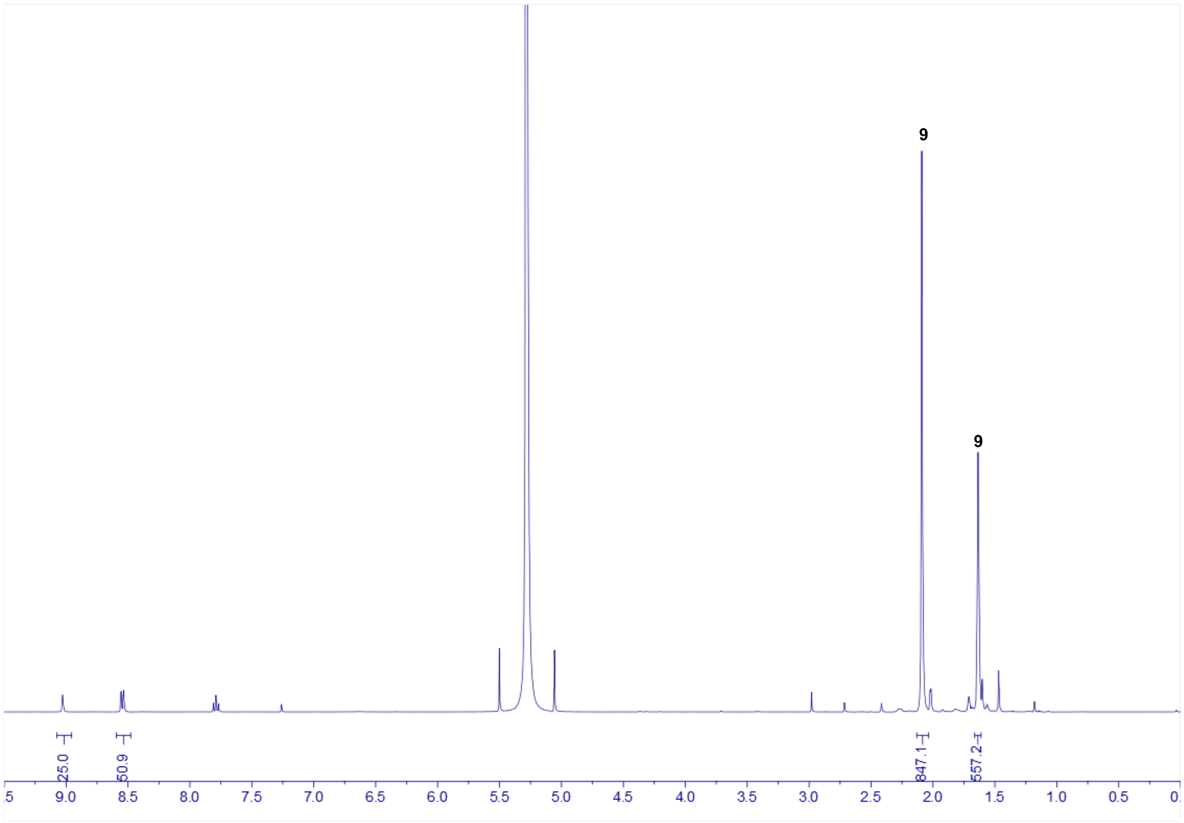

^{13}C Quantitative NMR (151 MHz, $CDCl_3$)

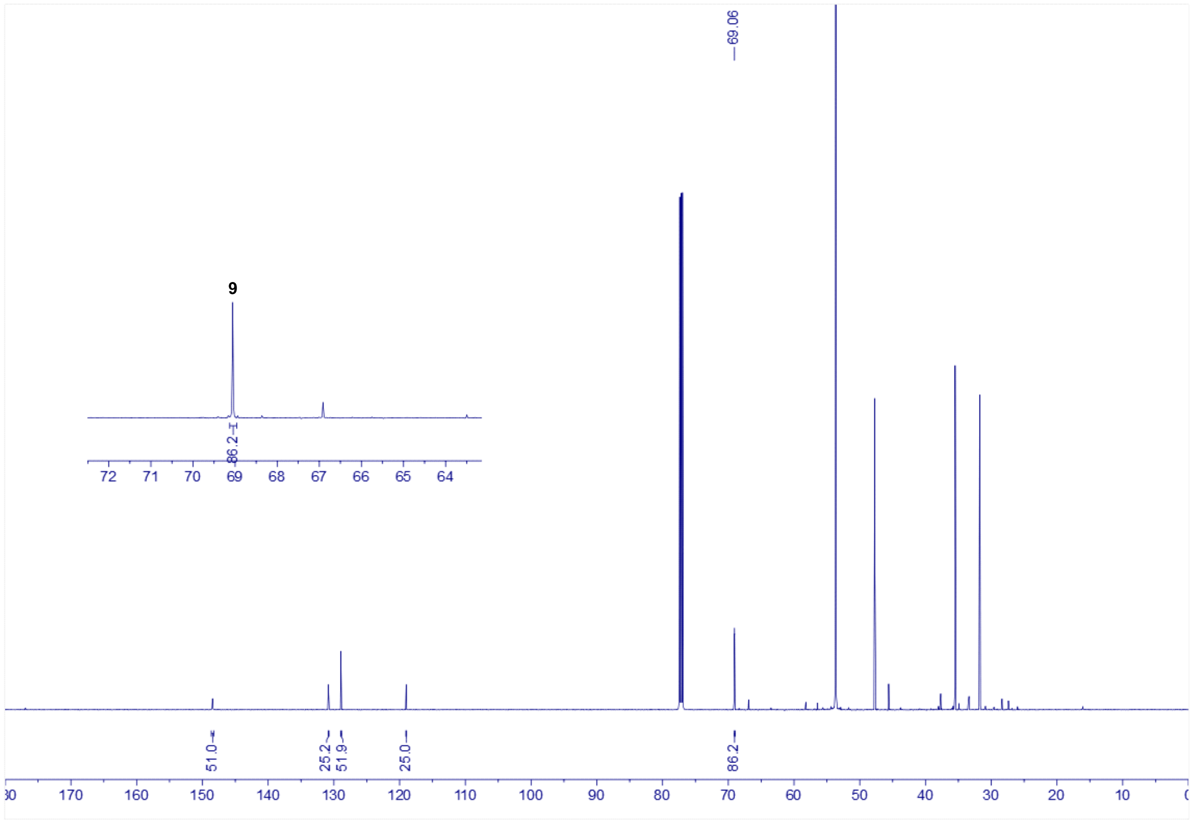

12.7 Chlorination of Norbornane to Give 10

2-Chlorobicyclo[2.2.1]heptane (10)

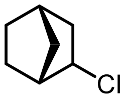

Starting Material	Observed Reaction Products	C-2 HAT Selectivity (%)
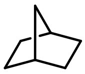 2	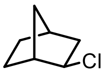 **10a** 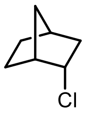 **10b**	100

Following **GP4** using **A4** as the amine and CH_2Cl_2 as the solvent, norbornane (19 mg, 0.2 mmol) gave **10** in 43% yield. GC-MS m/z (EI): 130.0 (M+).

10a, **10b**: Data in accordance with the literature.[19]

^{1}H NMR (400 MHz, $CDCl_3$)

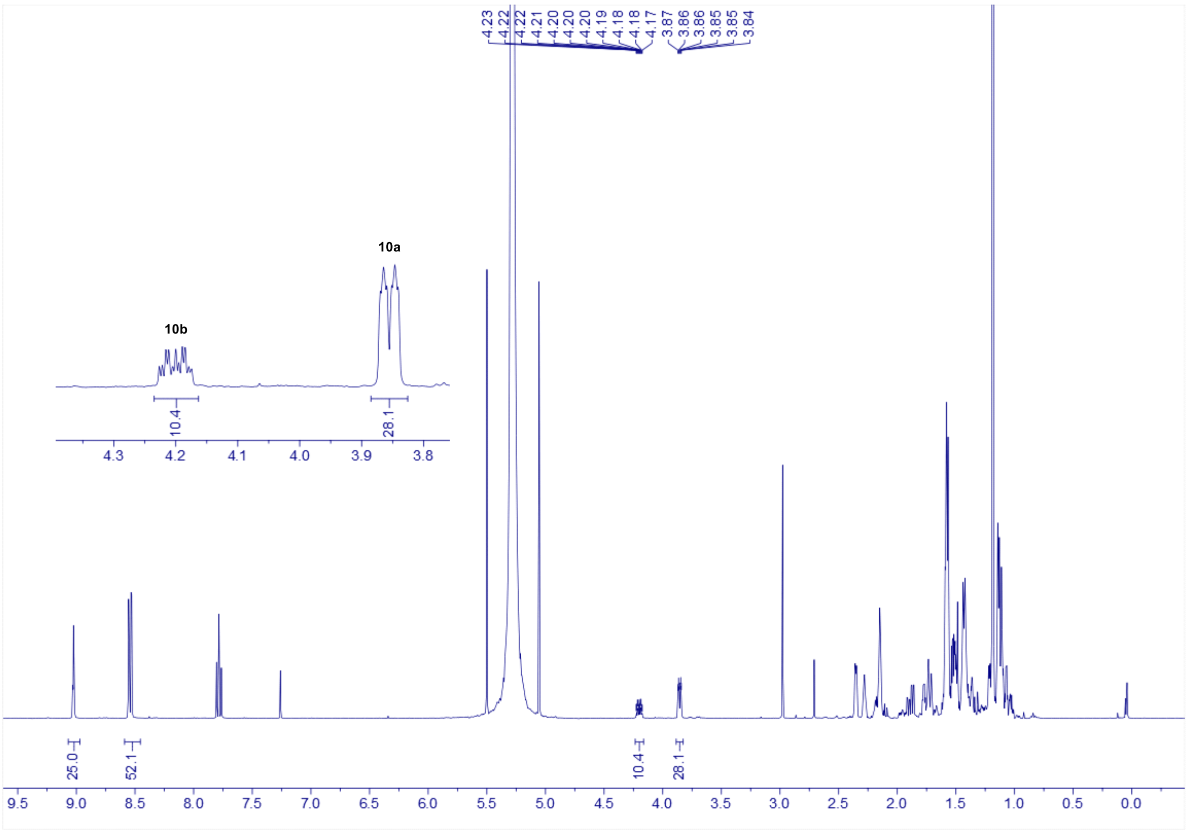

^{13}C Quantitative NMR (151 MHz, $CDCl_3$)

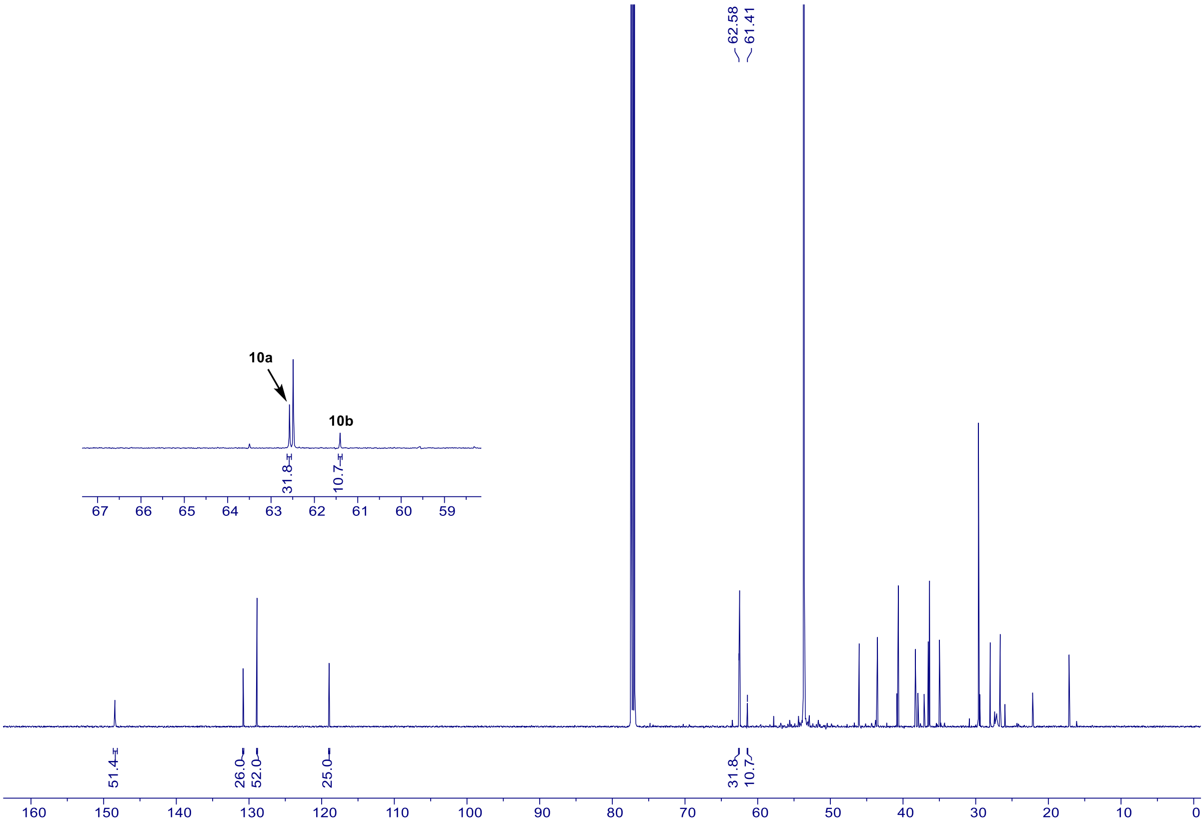

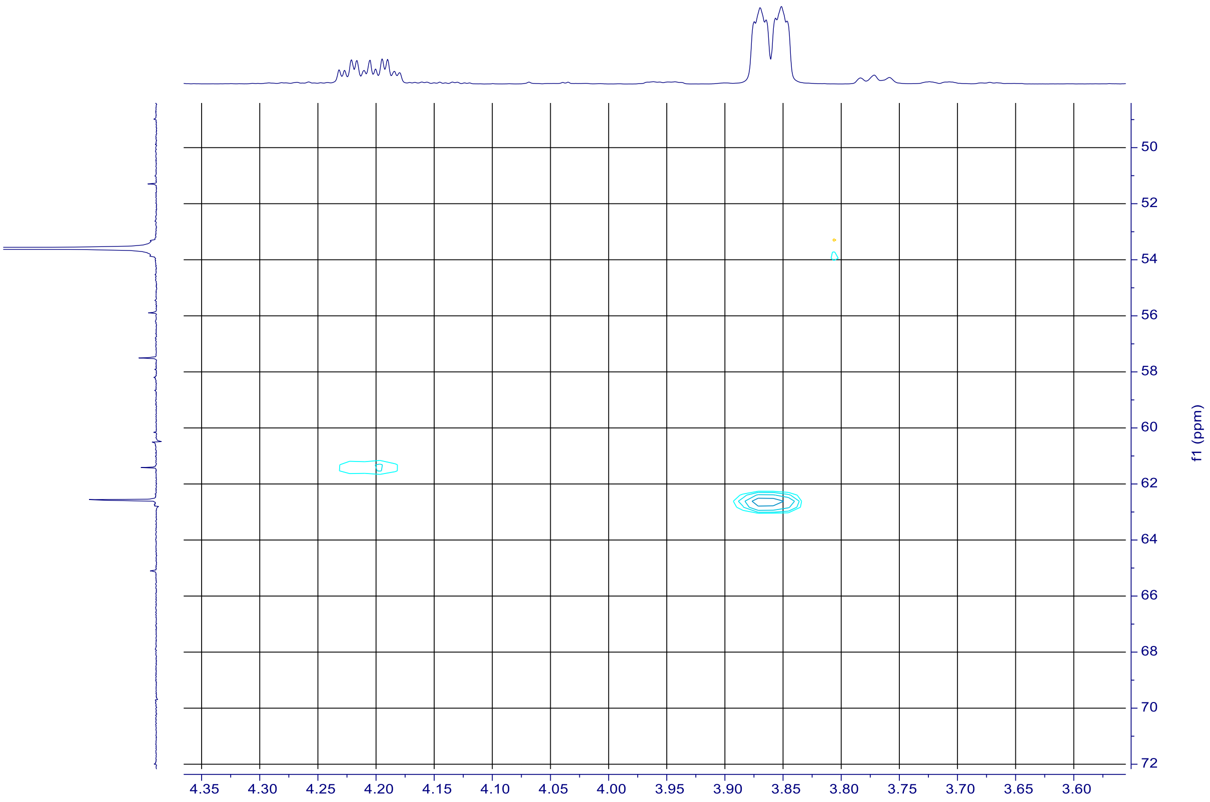
4.35
4.30
4.25
4.20
4.15
4.10
4.05
4.00
3.95
3.90
3.85
3.80
3.75
3.70
3.65
3.60
50
52
54
56
58
60
62
64
66
68
70
72
f1 (ppm)

12.8 Chlorination of *n*-Hexane to Give 11

2-Chlorohexane (11)

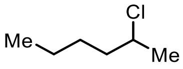

**Starting Material**	**Observed Reaction Products**		
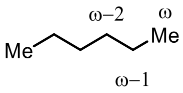	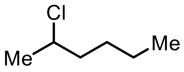 **11**	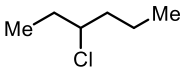 **11'**	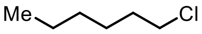 **11''**
**ω–1:ω–2:ω**	**ω–1 HAT Selectivity (%)**		
8.6:1.4:1.0	78		

Following **GP3** using **A4** as the amine and CH_2Cl_2 as the solvent, hexane (27 μL, 0.2 mmol) gave **11**, **11'** and **11''** in 35% yield. GC-MS m/z (EI): 120.1 (M+).

11, **11'**, **11''**: Data in accordance with the literature.[20]

^{1}H NMR (600 MHz, $CDCl_3$)

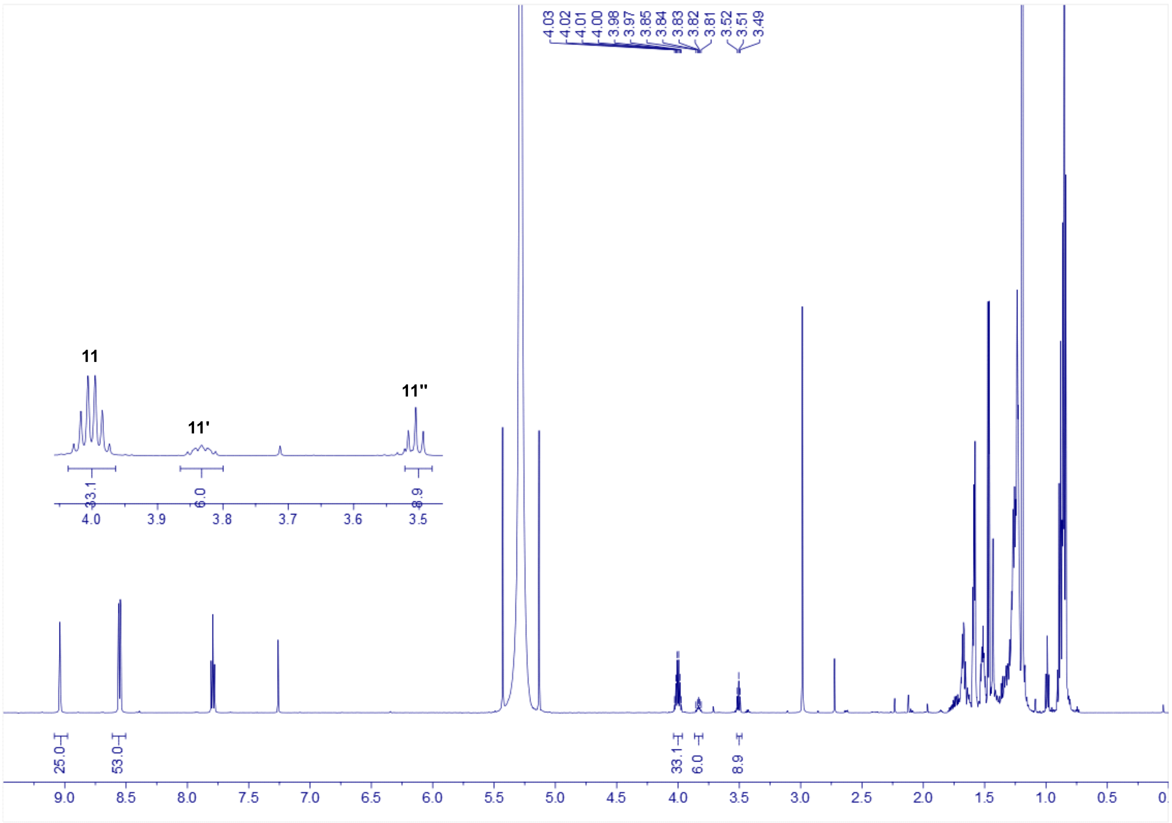

^{13}C NMR (151 MHz, $CDCl_3$)

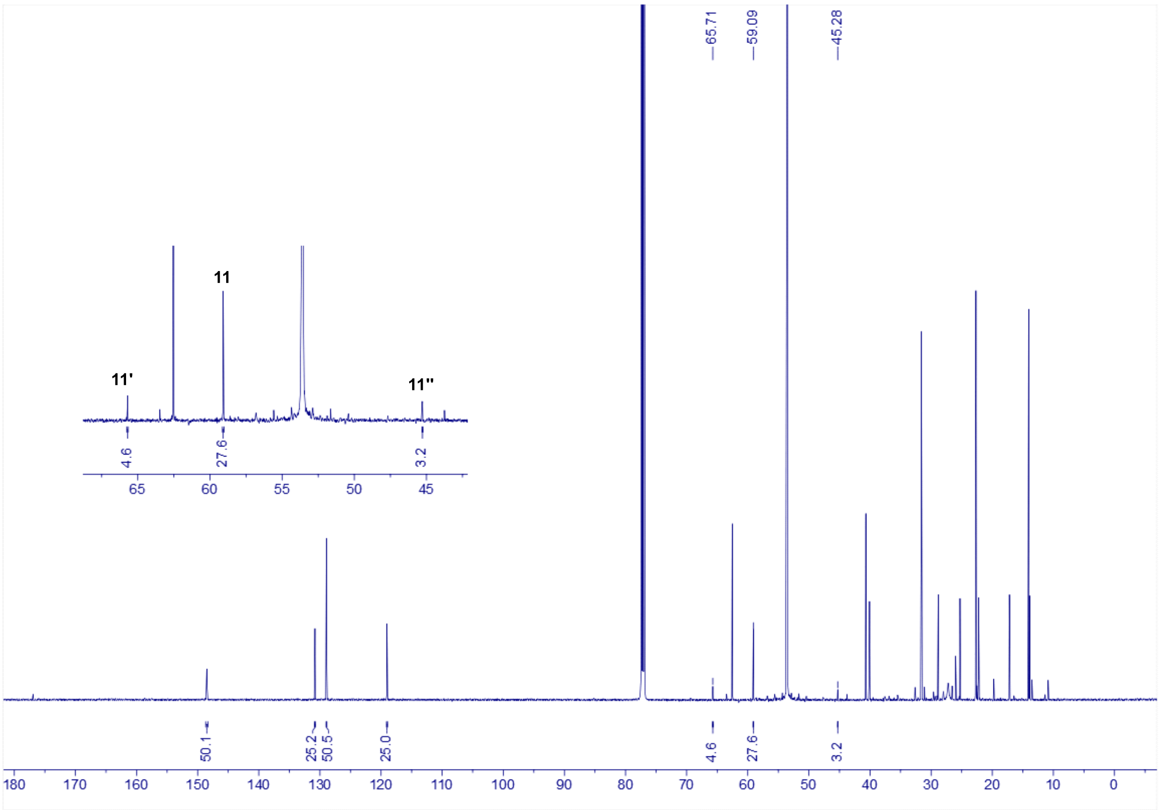

12.9 Chlorination of Methyl Hexanoate to Give 4

Methyl 5-chlorohexanoate (4)

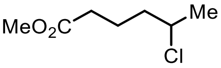

**Starting Material**	**Observed Reaction Products**		
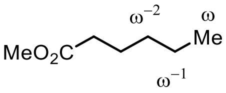	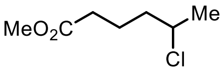 **4**	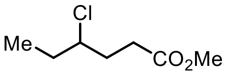 **4'**	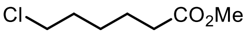 **4"**
**ω–1:ω–2:ω**	**ω–1 HAT Selectivity (%)**		
14:1.2:1.0	86		

Following **GP3** using **A3** as the amine and HFIP as the solvent, methyl hexanoate (30 μL, 0.2 mmol) gave **4**, **4’** and **4’’** in 70% yield. GC-MS m/z (EI): 164.1 (M+).

4, **4’**, **4’’**: Data in accordance with the literature.[21]

^{1}H NMR (600 MHz, $CDCl_3$)

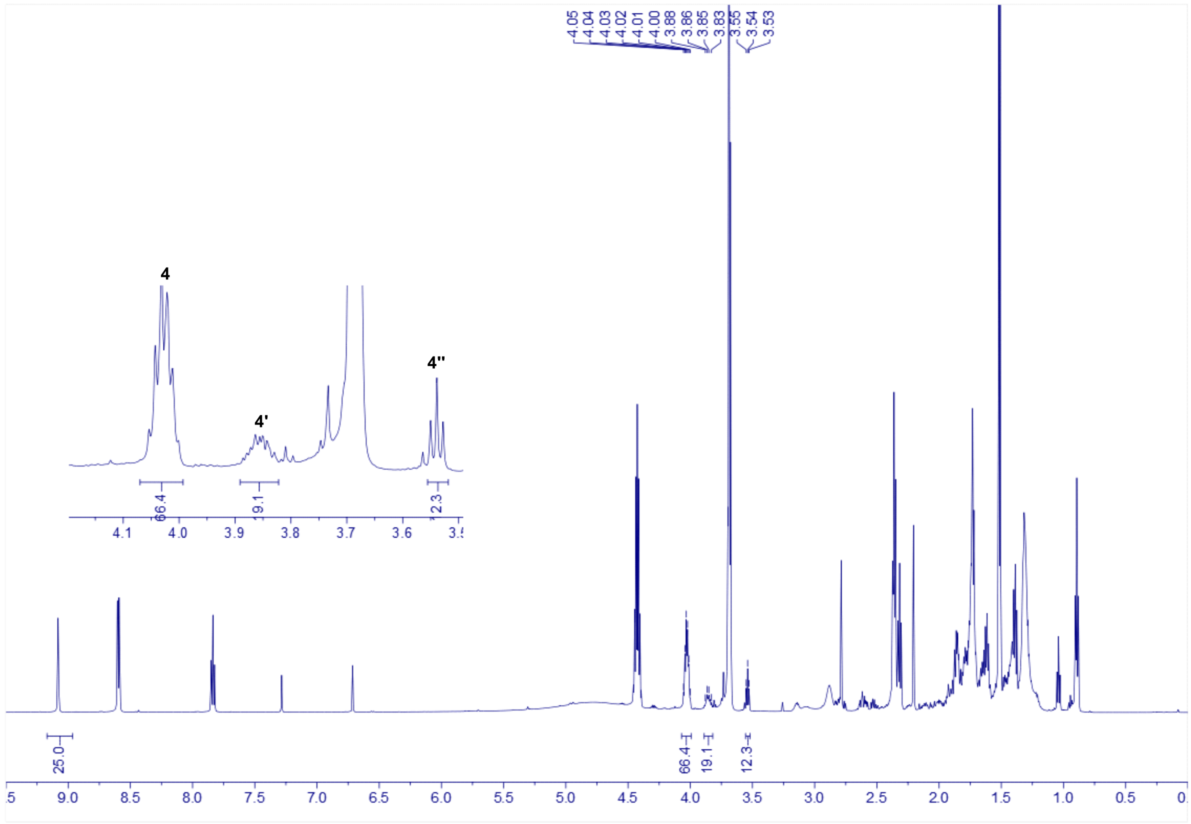

^{13}C Quantitative NMR (151 MHz, $CDCl_3$)

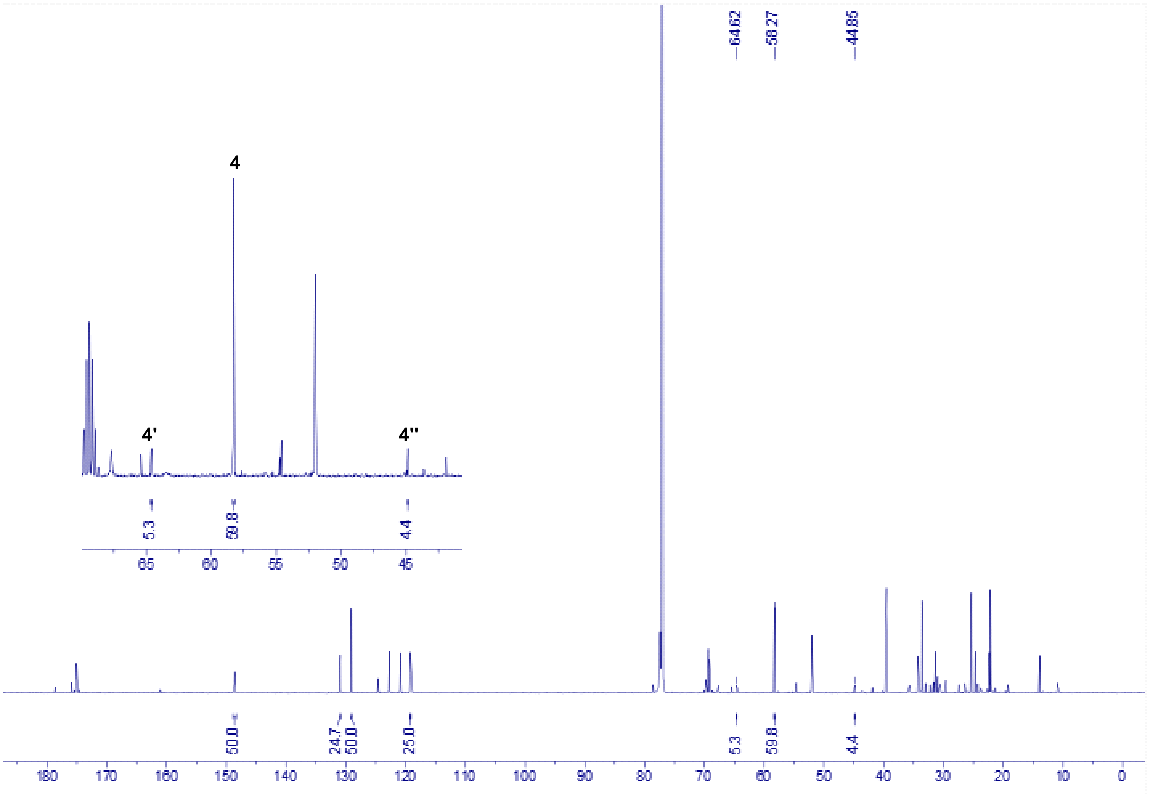

12.10 Chlorination of *N*,*N*-Dimethylhexanamide to Give 12

5-Chloro-*N*,*N*-dimethylhexanamide (12)

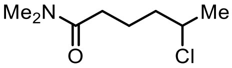

**Starting Material**	**Observed Reaction Products**
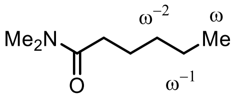	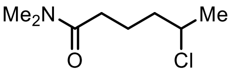 **12** 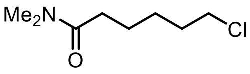 **12'** 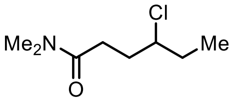 **12"**
**ω–1:ω :ω–2**	**ω–1 HAT Selectivity (%)**
23:2.2:1.0	88

Following **GP3** using **A3** as the amine and HFIP as the solvent, *N*,*N*-dimethylhexanamide (32 μL, 0.2 mmol) gave **12**, **12'** and **12''** in 77% yield. MS (APCI POS): 178.1 (MH+).

12, **12'**, **12''**: structural assignment done by analogy with similar compounds and distinctive $^{35/37}$Cl carbon peak shoulder.

^{1}H NMR (600 MHz, $CDCl_3$)

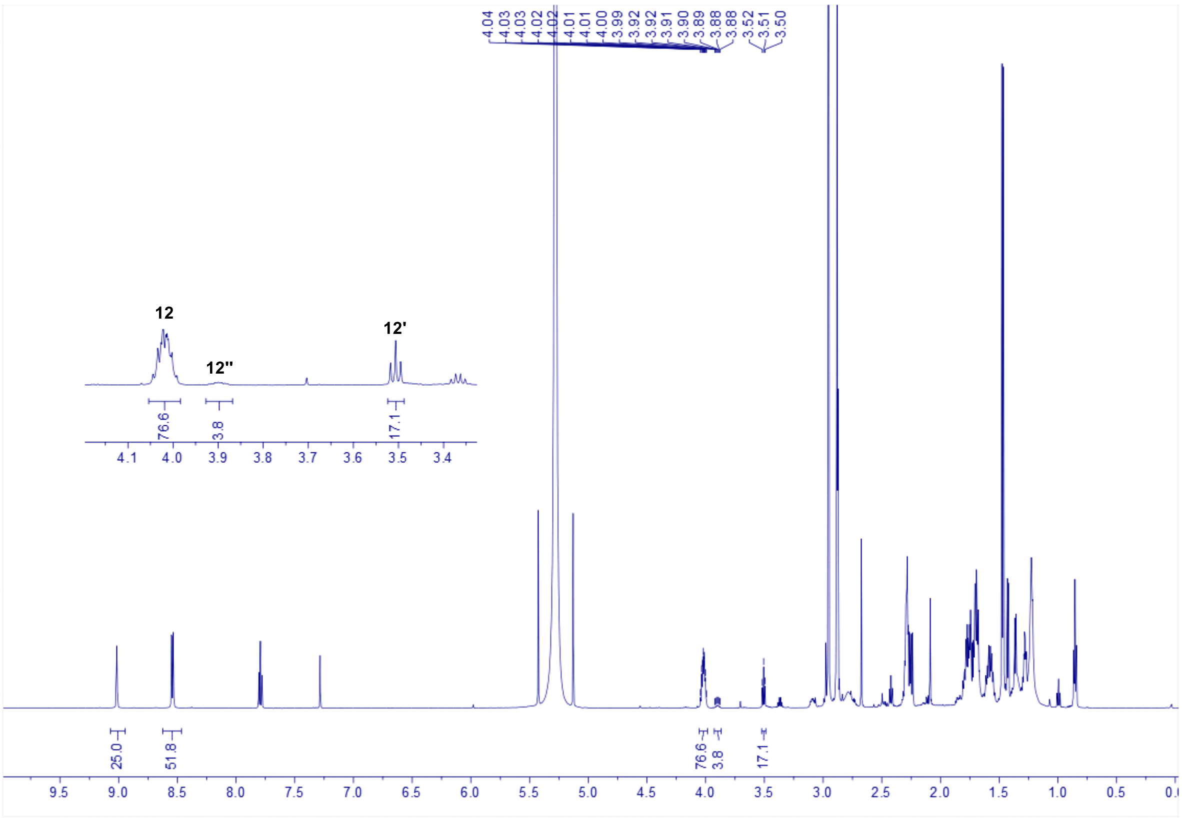

^{13}C Quantitative NMR (151 MHz, $CDCl_3$)

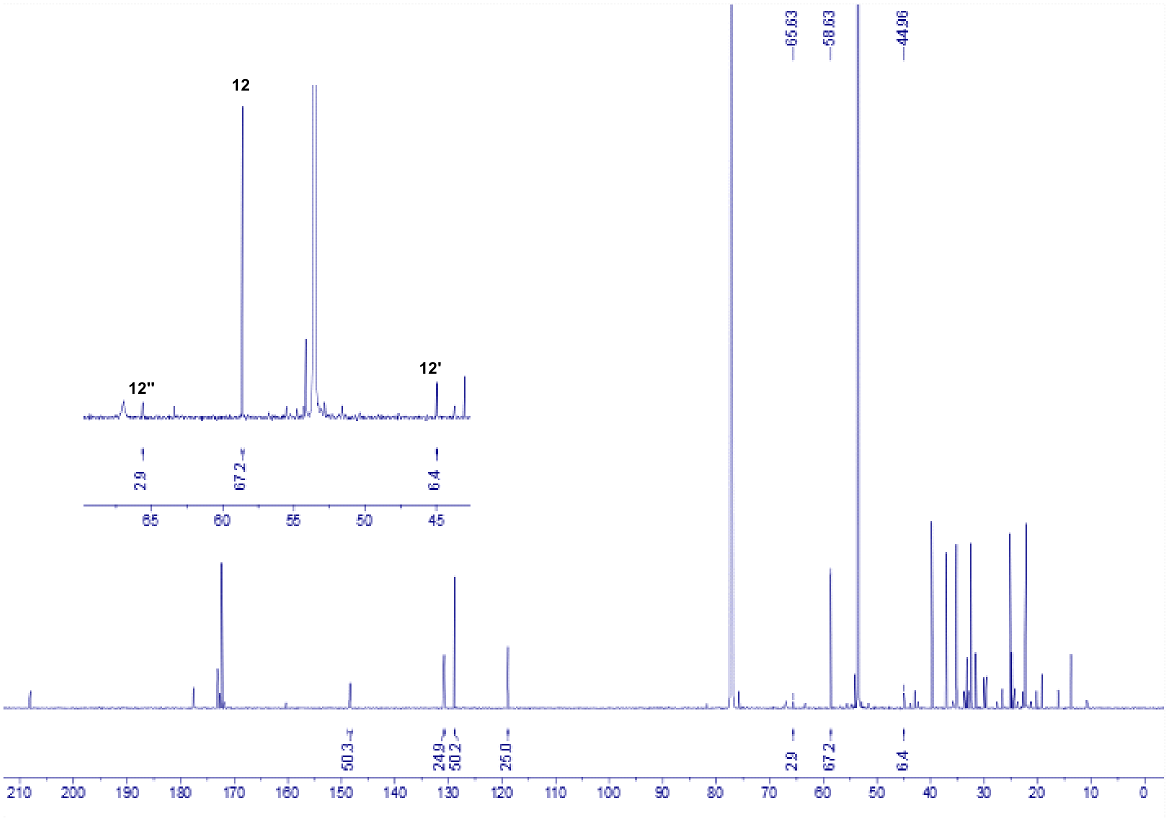

12.11 Chlorination of Heptan-2-one to Give 13

6-Chloroheptan-2-one (13)

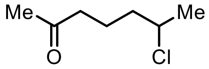

Starting Material	Observed Reaction Products		
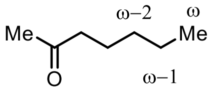	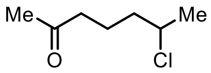 **13**	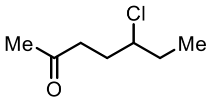 **13'**	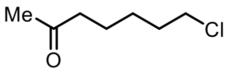 **13"**
**ω–1:ω–2:ω**	**ω–1 HAT Selectivity (%)**		
34:4.3:1.0	87		

Following **GP3** using **A3** as the amine and HFIP as the solvent, heptan-2-one (28 µL, 0.2 mmol) gave **13**, **13’** and **13’’** in 79% yield. MS (APCI POS): 148.9 (MH+).

13, **13’**: Structural assignment done by analogy with similar compounds and distinctive $^{35/37}$Cl carbon peak shoulder.
13’’: Data in accordance with the literature.[22]

^{1}H NMR (400 MHz, $CDCl_3$)

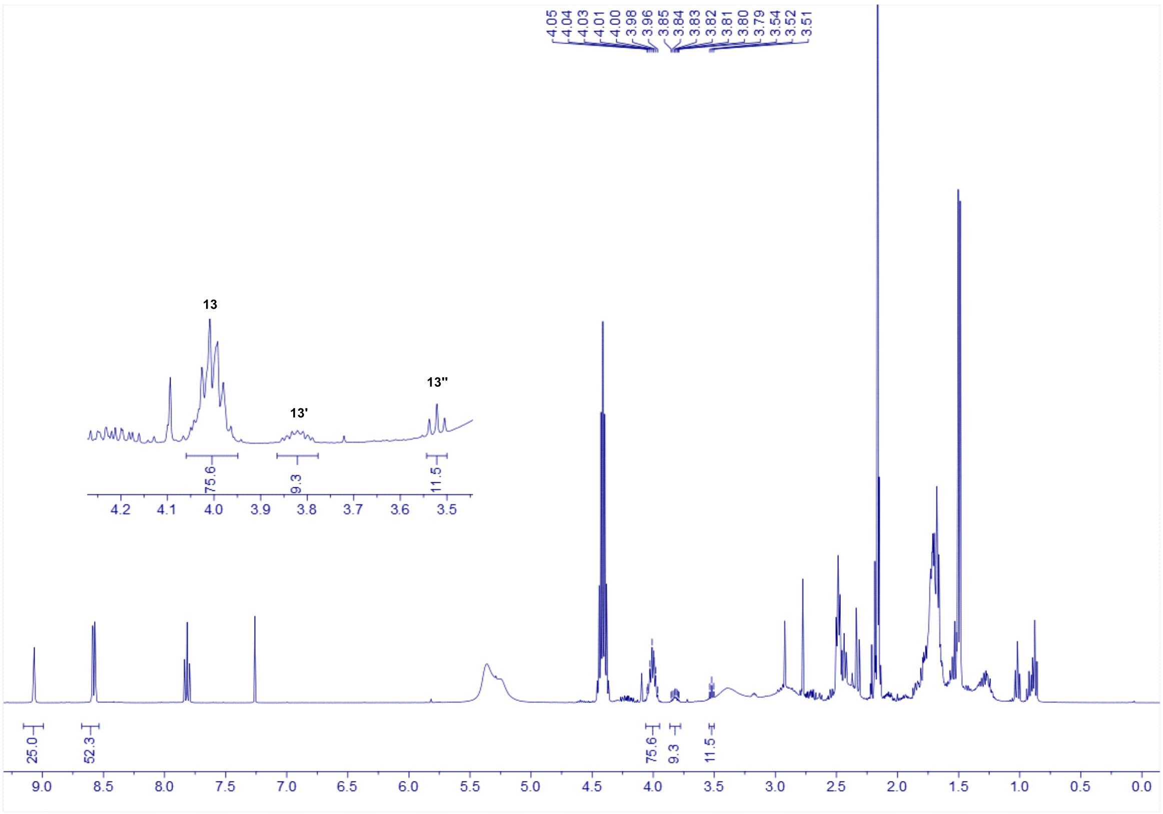

^{13}C Quantitative NMR (151 MHz, $CDCl_3$)

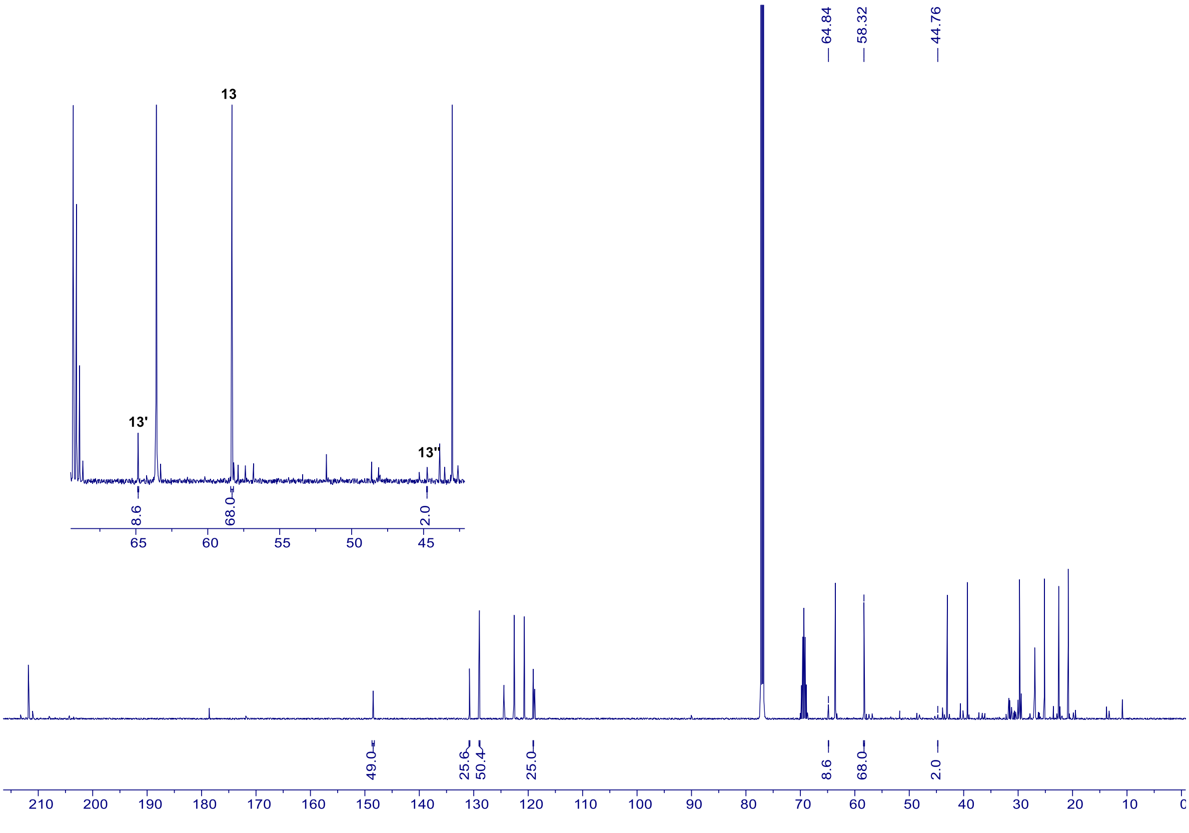

12.12 Chlorination of Hexanenitrile to Give 14

5-Chlorohexanenitrile (14)

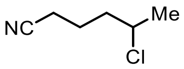

Starting Material	Observed Reaction Products
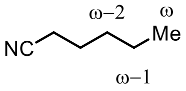	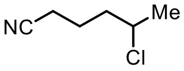 **14** 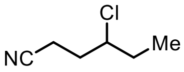 **14'** 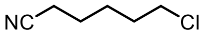 **14"**
**ω–1:ω–2:ω**	**ω–1 HAT Selectivity (%)**
6.4:1.1:1.0	75

Following **GP3** using **A3** as the amine and HFIP as the solvent, hexanenitrile (24 μL, 0.2 mmol) gave **14**, **14’** and **14’’** in 37% yield. GC-MS m/z (EI): 131.1 (M+).

14: Data in accordance with the literature.[2]

14’: Structural assignment done by analogy with similar compounds and distinctive $^{35/37}$Cl carbon peak shoulder.

14’’: Data in accordance with the literature.[23]

^{1}H NMR (600 MHz, $CDCl_3$)

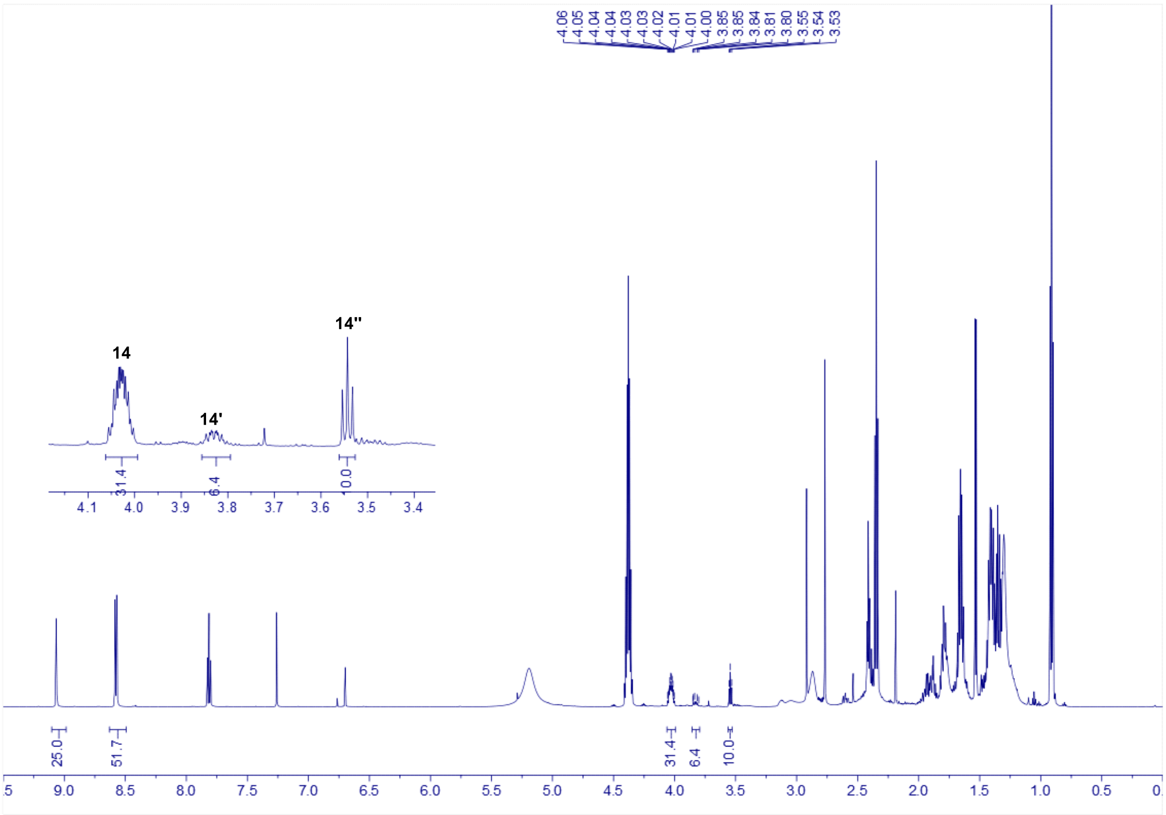

^{13}C Quantitative NMR (151 MHz, $CDCl_3$)

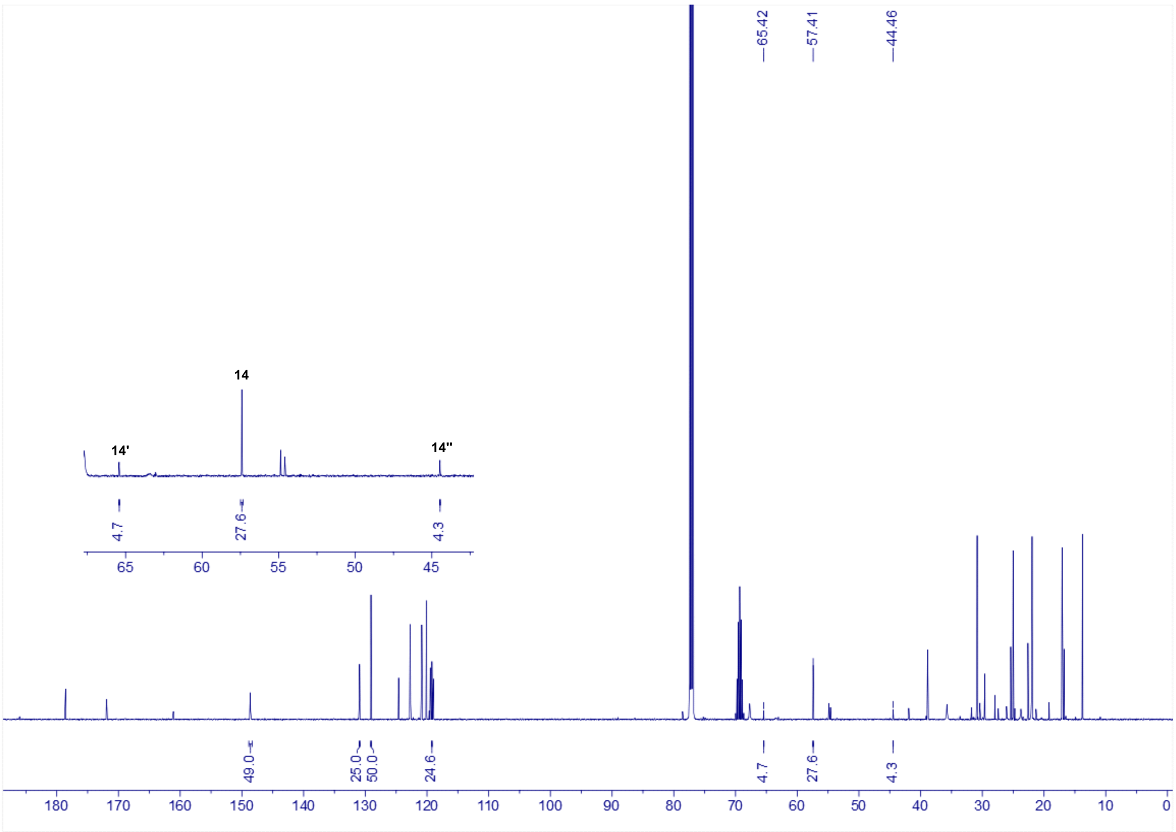

12.13 Chlorination of 1-Chloropentane to Give 15

1,4-Dichloropentane (15)

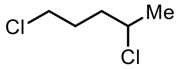

Starting Material	Observed Reaction Products
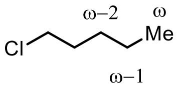	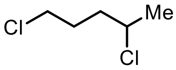 **15** 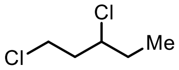 **15'** 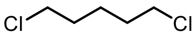 **15''**
**ω–1:ω–2:ω**	**ω–1 HAT Selectivity (%)**
12:1.6:1.0	82

Following **GP3** using **A3** as the amine and HFIP as the solvent, methyl 1-chloropentane (24 μL, 0.2 mmol) gave **15**, **15’** and **15’’** in 61% yield. GC-MS m/z (EI): 140.0 (M+).

15: Data in accordance with the literature.[2]

15’: Structural assignment done by analogy with similar compounds and distinctive $^{35/37}$Cl carbon peak shoulder.

15’’: Data in accordance with the literature. [24]

^1H NMR (600 MHz, $CDCl_3$)

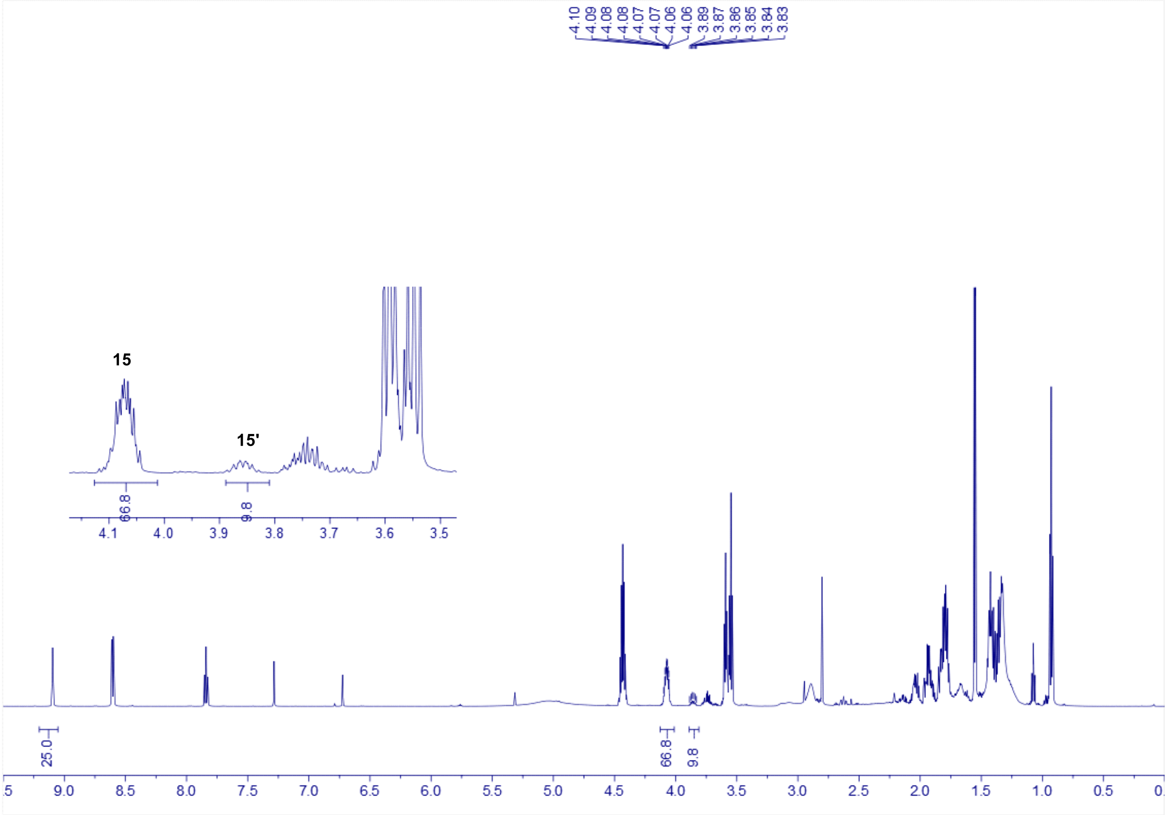

^{13}C Quantitative NMR (151 MHz, $CDCl_3$)

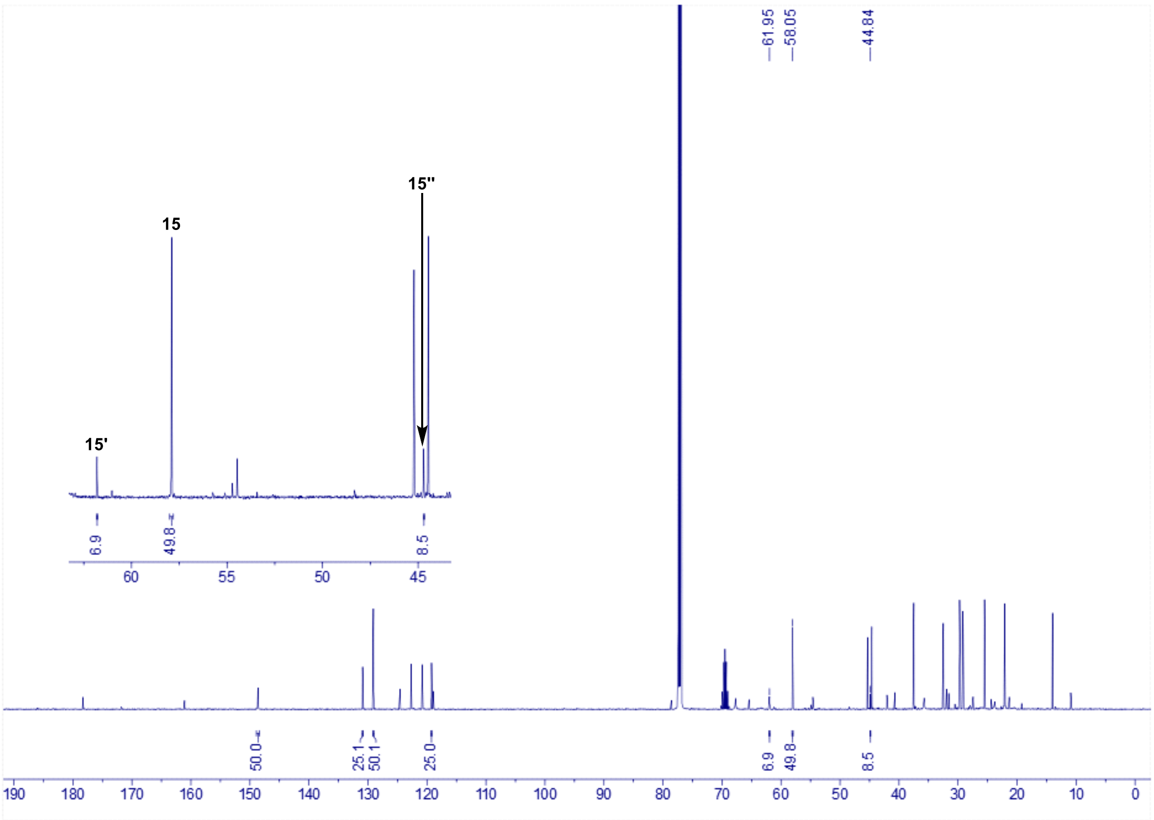

12.14 Chlorination of 1-Bromopentane to Give 16

1-Bromo-4-chloropentane (16)

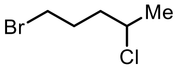

**Starting Material**	**Observed Reaction Products**		
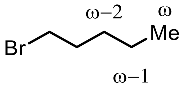	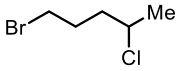 **16**	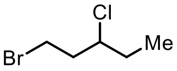 **16'**	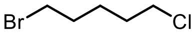 **16"**
**ω–1:ω–2:ω**	**ω–1 HAT Selectivity (%)**		
9.3:1.4:1.0	80		

Following **GP3** using **A3** as the amine and CH_2Cl_2 as the solvent, 1-bromopentane (25 μL, 0.2 mmol) gave **16**, **16'**, **16''** and **16'''** in 73% yield. GC-MS m/z (EI): 184.0 (M+).

16, **16'**, **16''**: Structural assignment done by analogy with similar compounds and distinctive $^{35/37}$Cl carbon peak shoulder.

^{1}H NMR (600 MHz, $CDCl_3$)

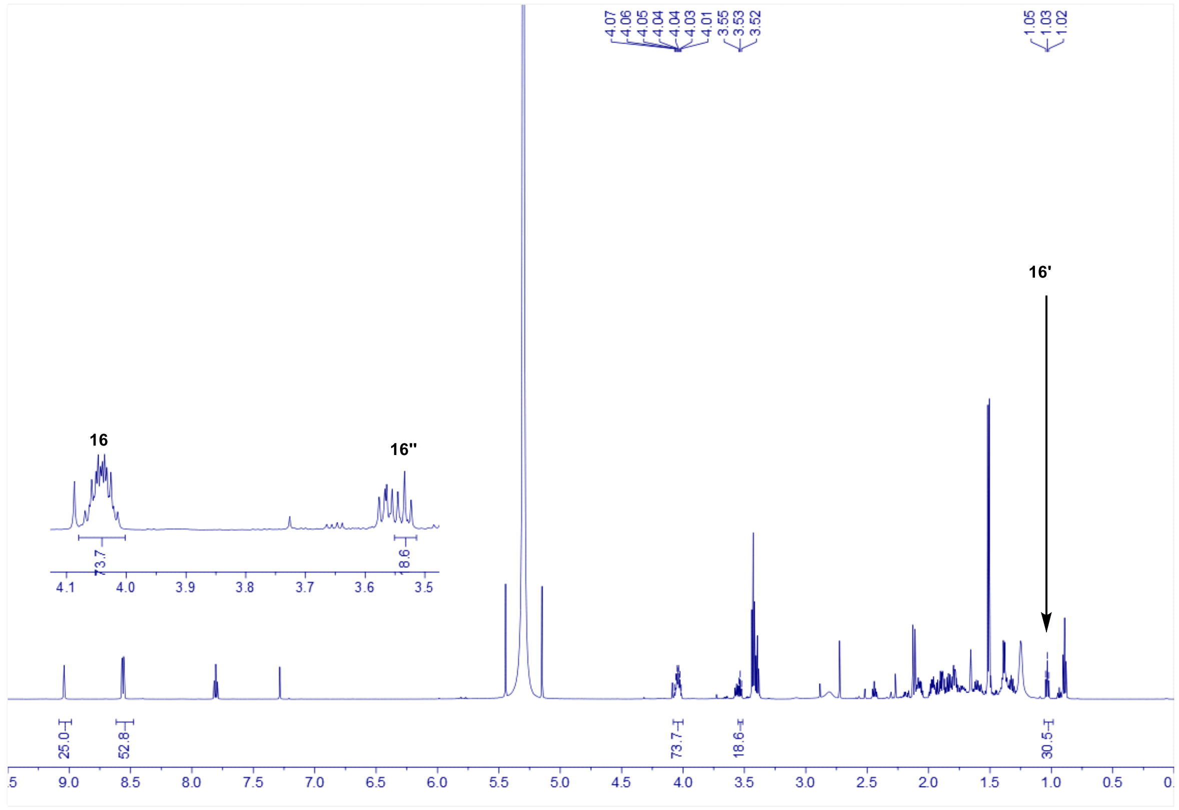

^{13}C NMR (151 MHz, $CDCl_3$)

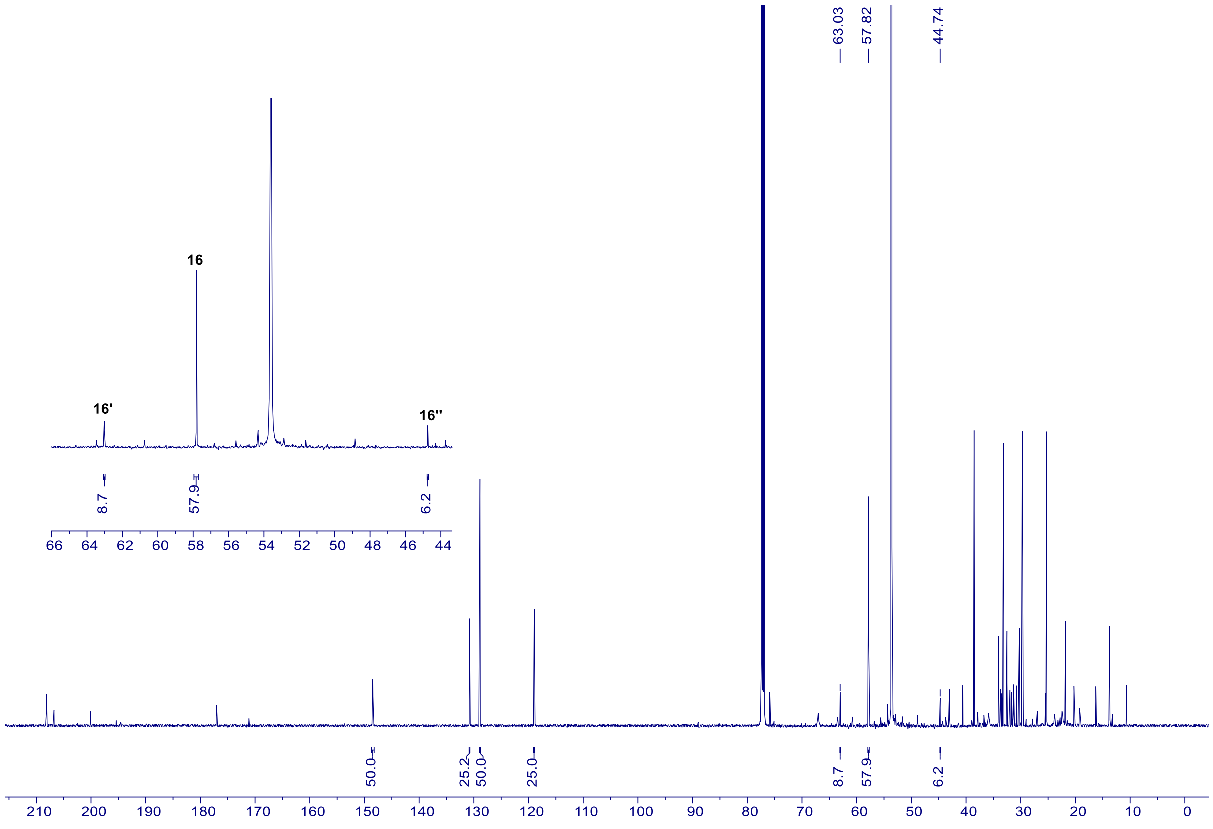

12.15 Chlorination of 1-Fluoropentane to Give 17

4-Chloro-1-fluoropentane (17)

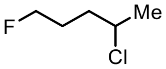

Starting Material	Observed Reaction Products		
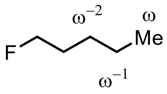	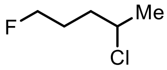 **17**	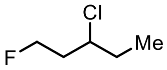 **17'**	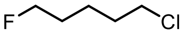 **17"**
**ω–1:ω–2:ω**	**ω–1 HAT Selectivity (%)**		
15:4.3:1.0	74		

Following **GP3** using **A3** as the amine and HFIP as the solvent, 1-fluoropentane (23 μL, 0.2 mmol) gave **17**, **17’** and **17’’** in 60% yield. GC-MS m/z (EI): 124.1 (M+).

17, **17’**: Structural assignment done by analogy with similar compounds and distinctive $^{35/37}$Cl carbon peak shoulder. **17’** assignment is also supported by $J_{C–F}$ = 4.5 Hz.
17’’: Data in accordance with the literature.[25]

^{1}H NMR (600 MHz, $CDCl_3$)

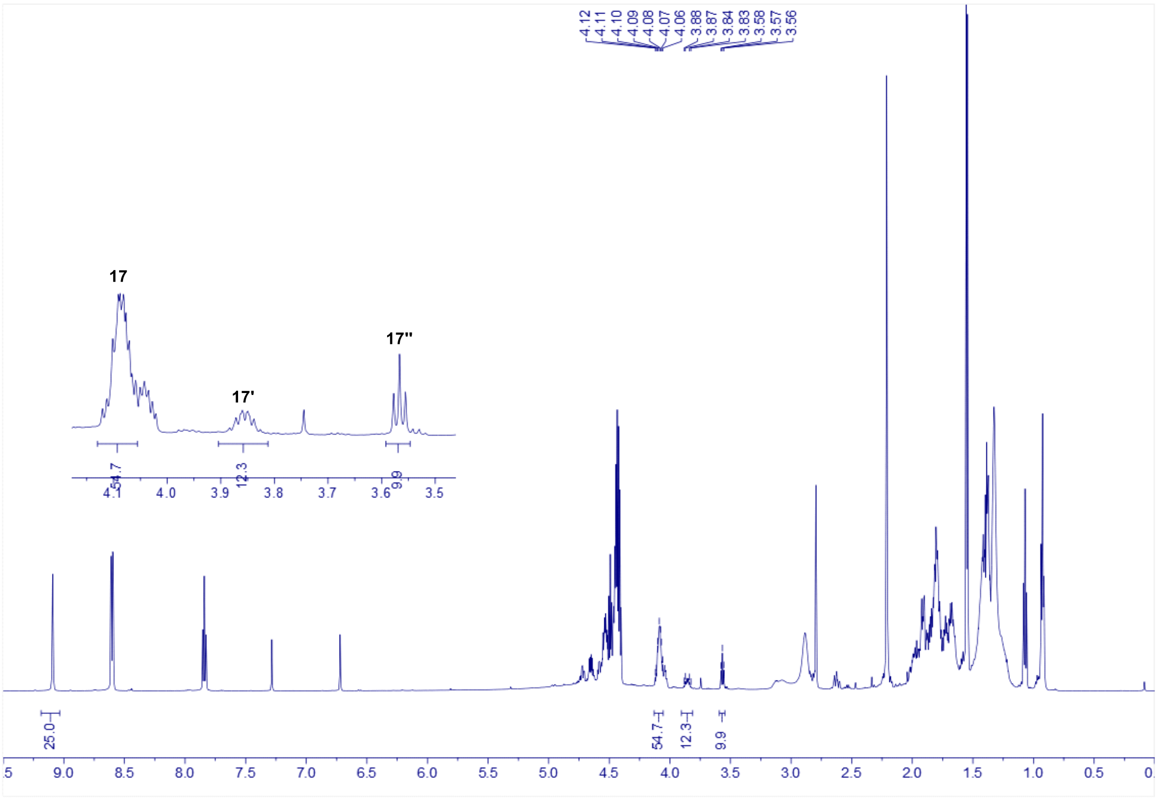

^{13}C Quantitative NMR (151 MHz, $CDCl_3$)

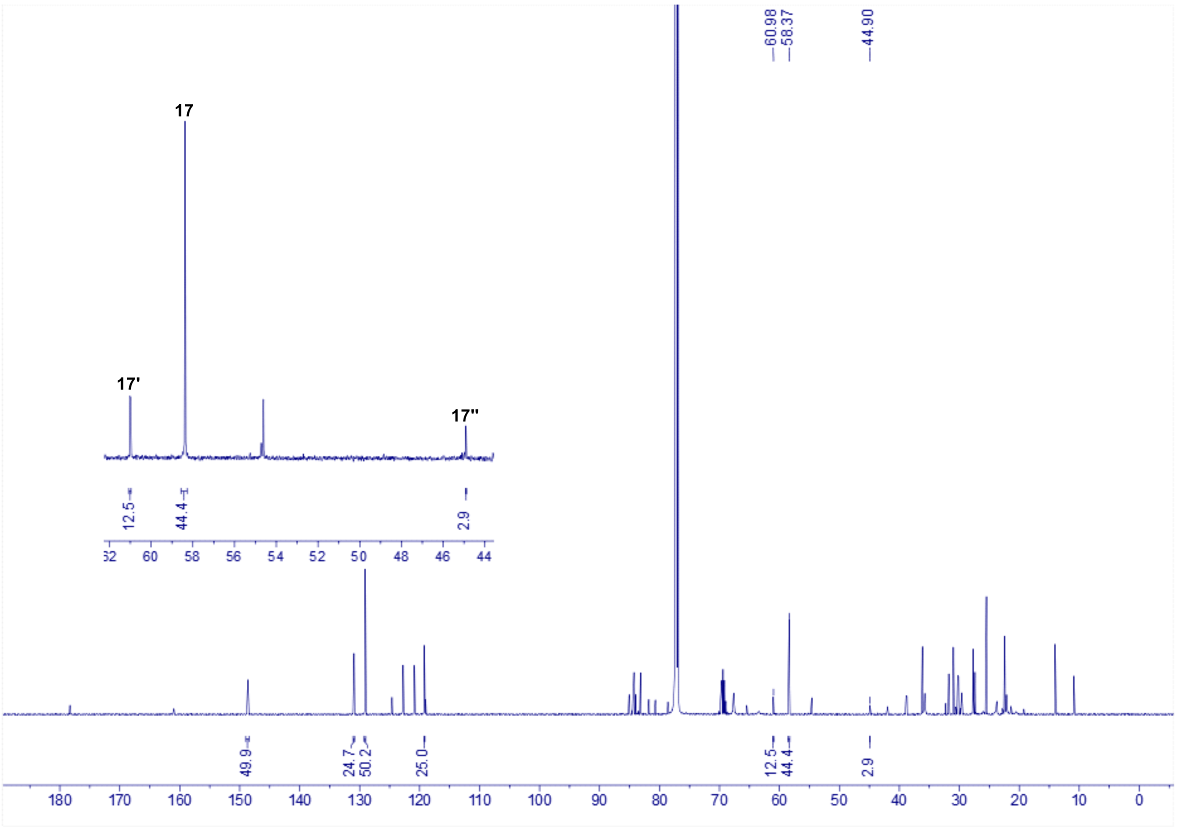

12.16 Chlorination of Pentyl Benzenesulfonate to Give 18

4-Chloropentyl Benzenesulfonate (18)

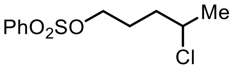

**Starting Material**	**Observed Reaction Products**		
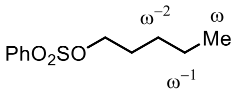	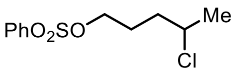 18	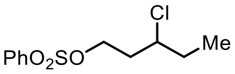 18'	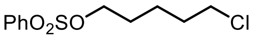 18''
**ω–1:ω–2:ω**	**ω–1 HAT Selectivity (%)**		
17:1.7:1.0	87		

Following **GP3** using **A1** as the amine and HFIP as the solvent, pentyl benzenesulfonate (41 μL, 0.2 mmol) gave **18**, **18’** and **18’’** in 62% yield. GC-MS m/z (EI): 262.1 (M+).

18, **18’**, **18’’**: Data in accordance with the literature.[2]

^1H NMR (600 MHz, $CDCl_3$)

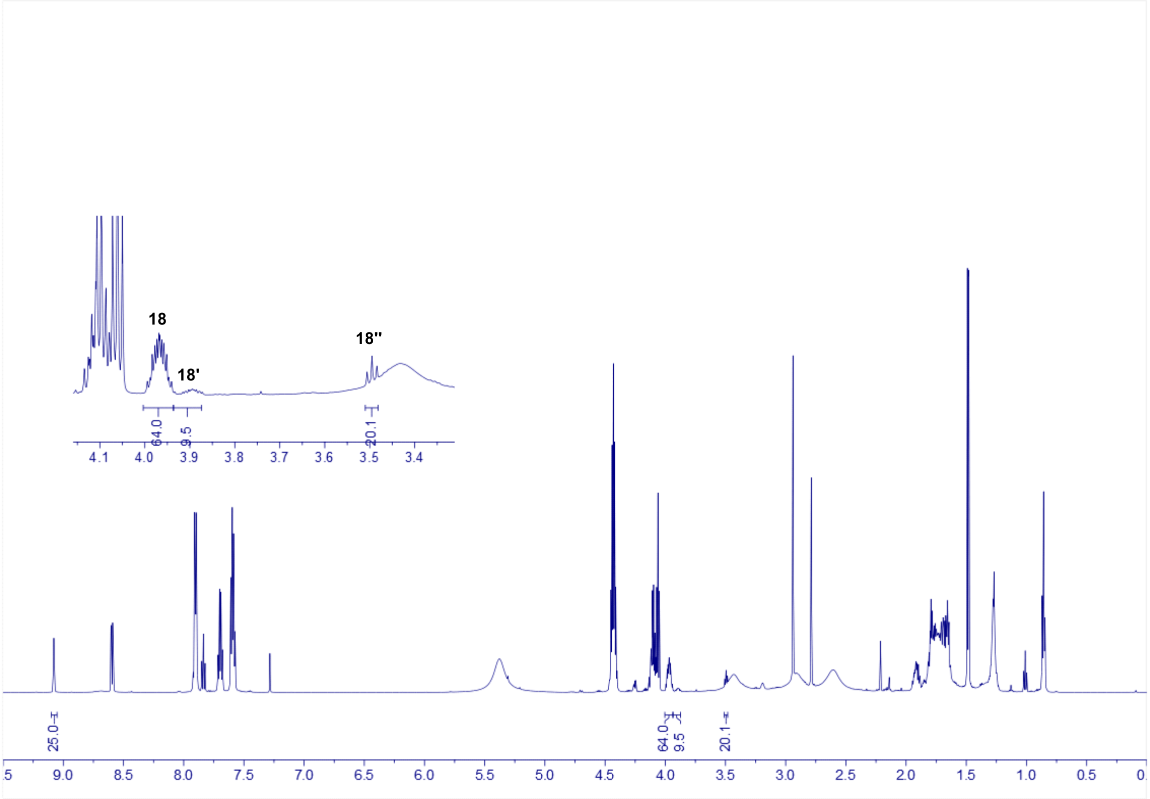

^{13}C Quantitative NMR (151 MHz, $CDCl_3$)

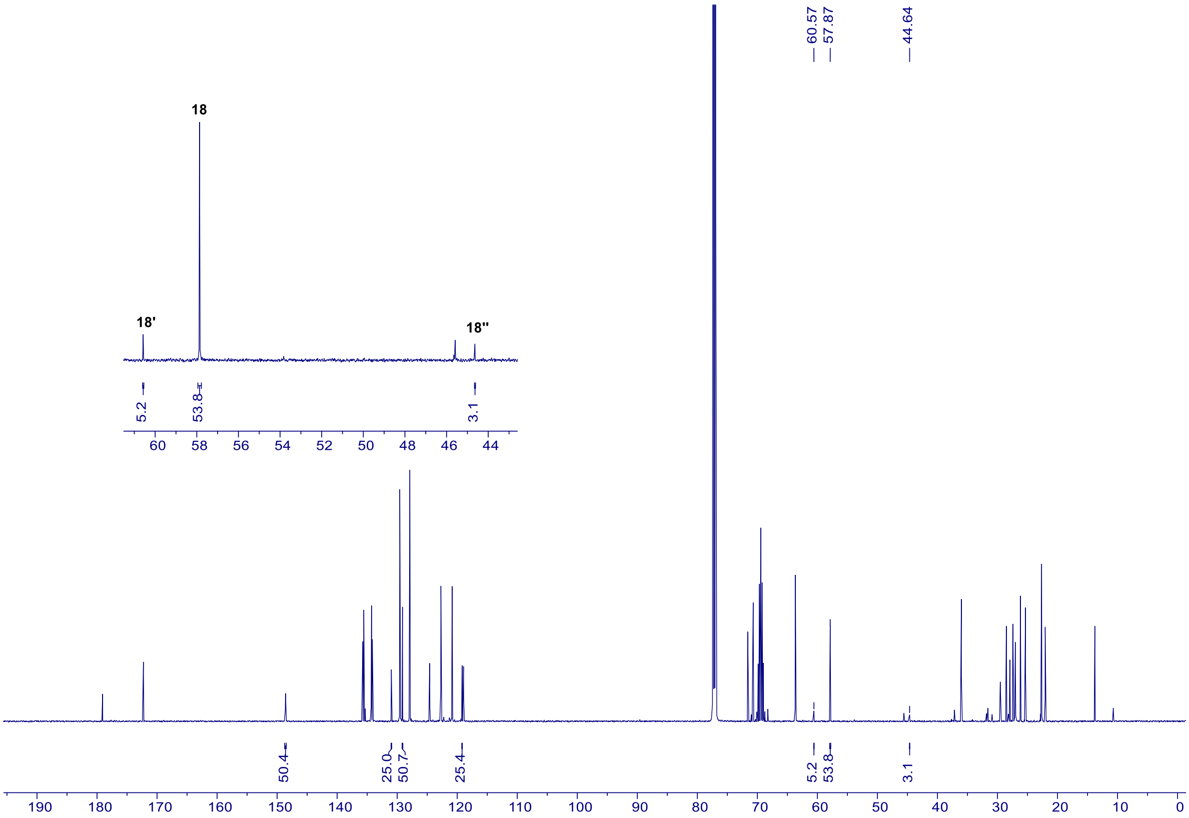

12.17 Chlorination of Pentyl Acetate to Give 19

4-Chloropentyl Acetate (19)

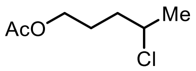

Starting Material	Observed Reaction Products		
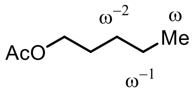	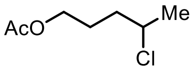 **19**	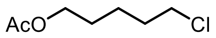 **19'**	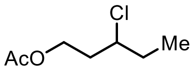 **19''**
**ω–1:ω :ω–2**	**ω–1 HAT Selectivity (%)**		
16:1.3:1.0	88		

Following **GP3** using **A3** as the amine and HFIP as the solvent, pentyl acetate (30 μL, 0.2 mmol) gave **19**, **19’** and **19’’** in 51% yield. GC-MS m/z (EI): 164.1 (M+).

19: Data in accordance with the literature.[2]

19’: Data in accordance with the literature.[26]

19’’: Structural assignment done by analogy with similar compounds and distinctive $^{35/37}$Cl carbon peak shoulder.

^{1}H NMR (600 MHz, $CDCl_3$)

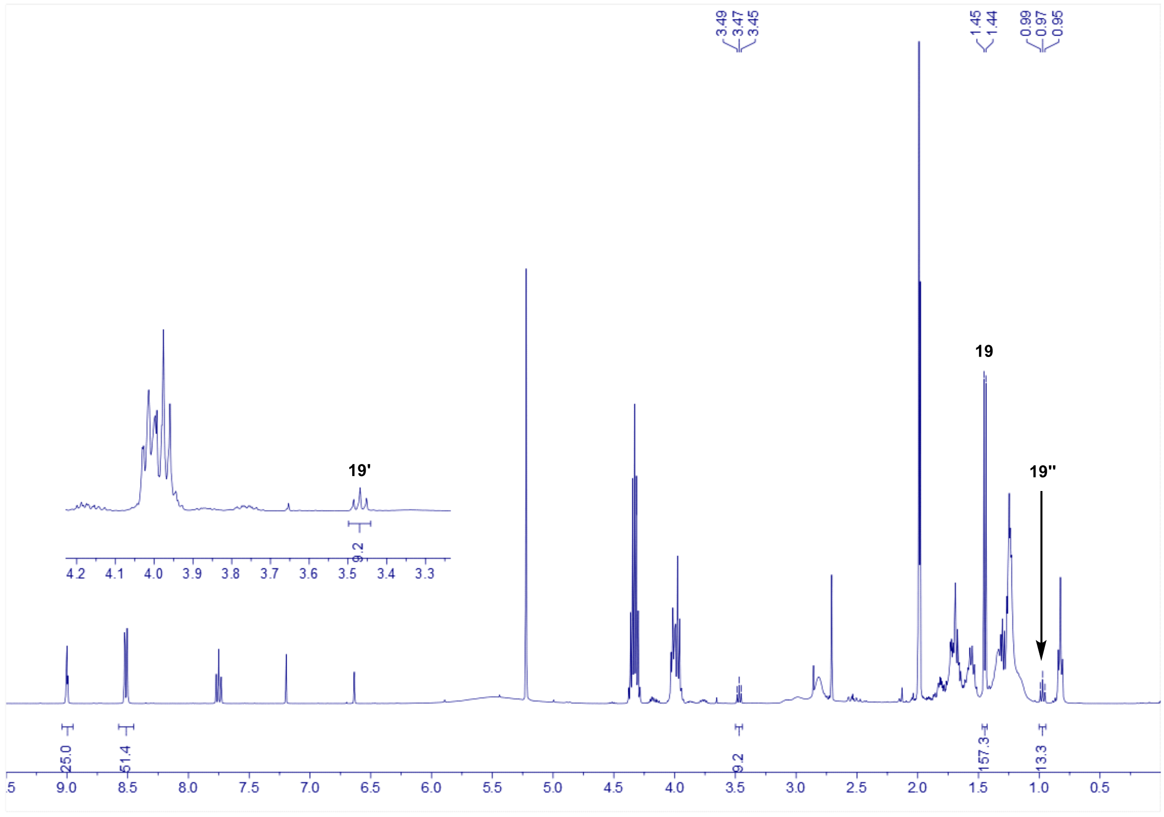

^{13}C Quantitative NMR (151 MHz, $CDCl_3$)

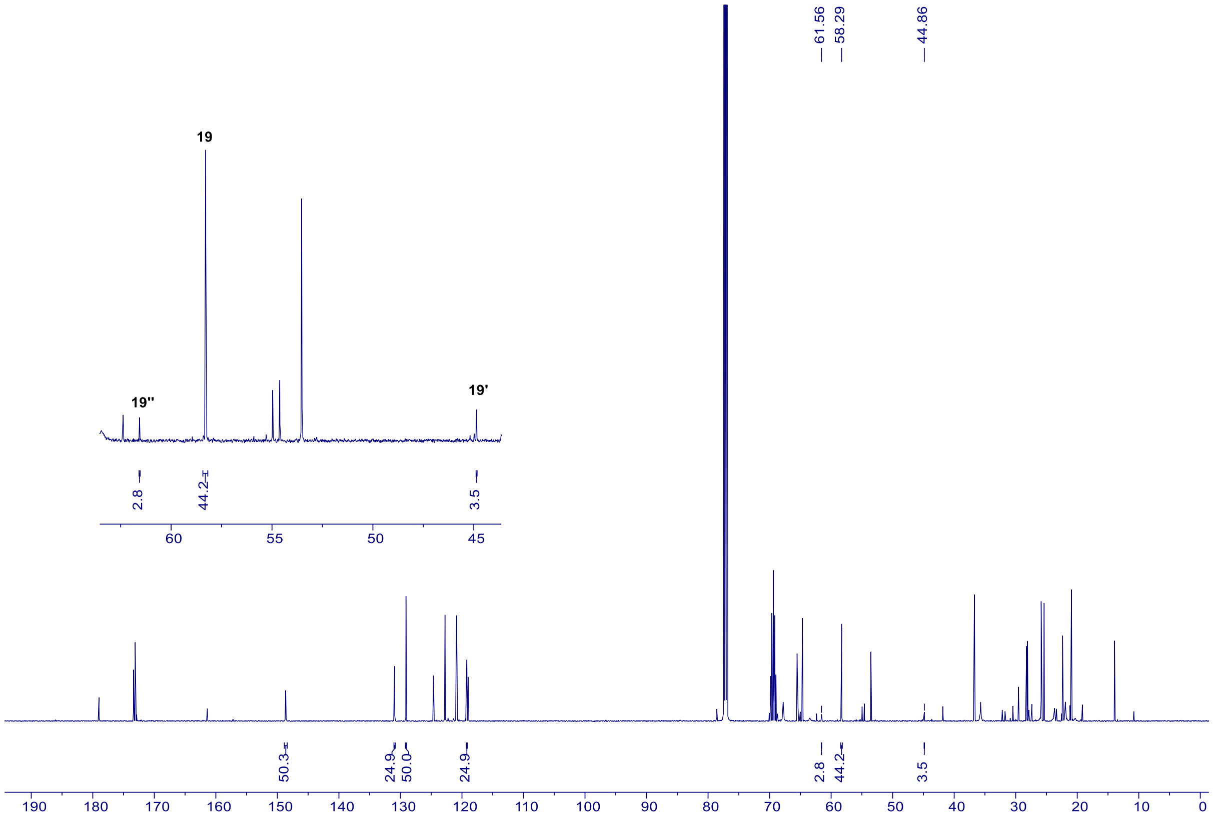

12.18 Chlorination of 2-Pentylisoindoline-1,3-dione to Give 20

2-(4-Chloropentyl)isoindoline-1,3-dione (20)

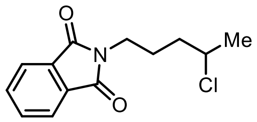

**Starting Material**	**Observed Reaction Products**
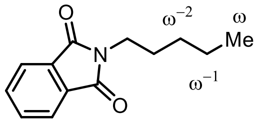	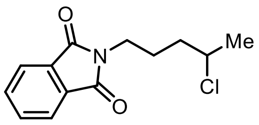 20 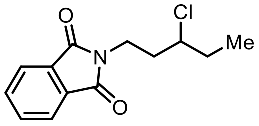 20' 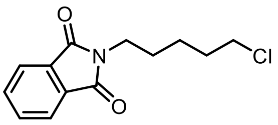 20"
**ω–1:ω–2:ω**	**ω–1 HAT Selectivity (%)**
24:2.2:1.0	88

Following **GP3** using **A1** as the amine and HFIP as the solvent, 2-Pentylisoindoline-1,3-dione (43.5 mg, 0.2 mmol) gave **20**, **20'** and **20''** in 77% yield. GC-MS m/z (EI): 251.1 (M+).

20: Data in accordance with the literature.[2]

20': Structural assignment done by analogy with similar compounds and distinctive $^{35/37}$Cl carbon peak shoulder.

20'': Data in accordance with the literature.[27]

^{1}H NMR (600 MHz, $CDCl_3$)

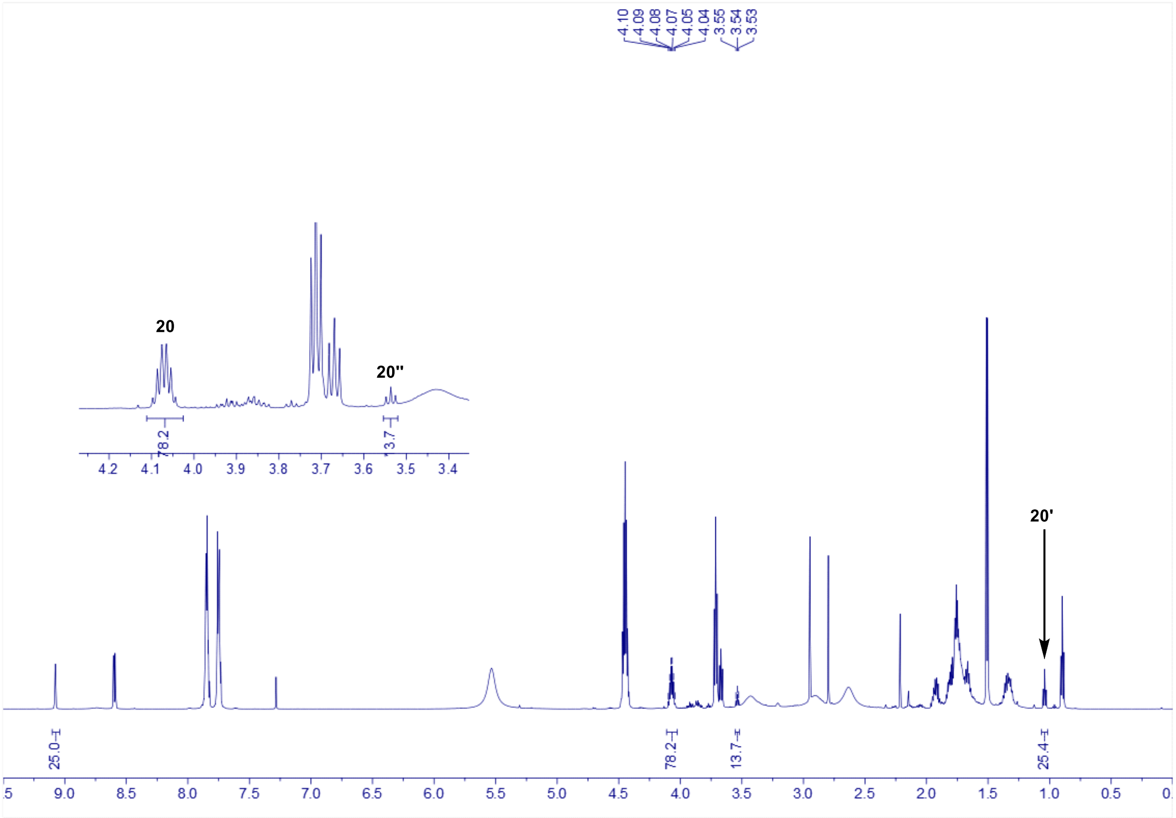

^{13}C NMR (151 MHz, $CDCl_3$)

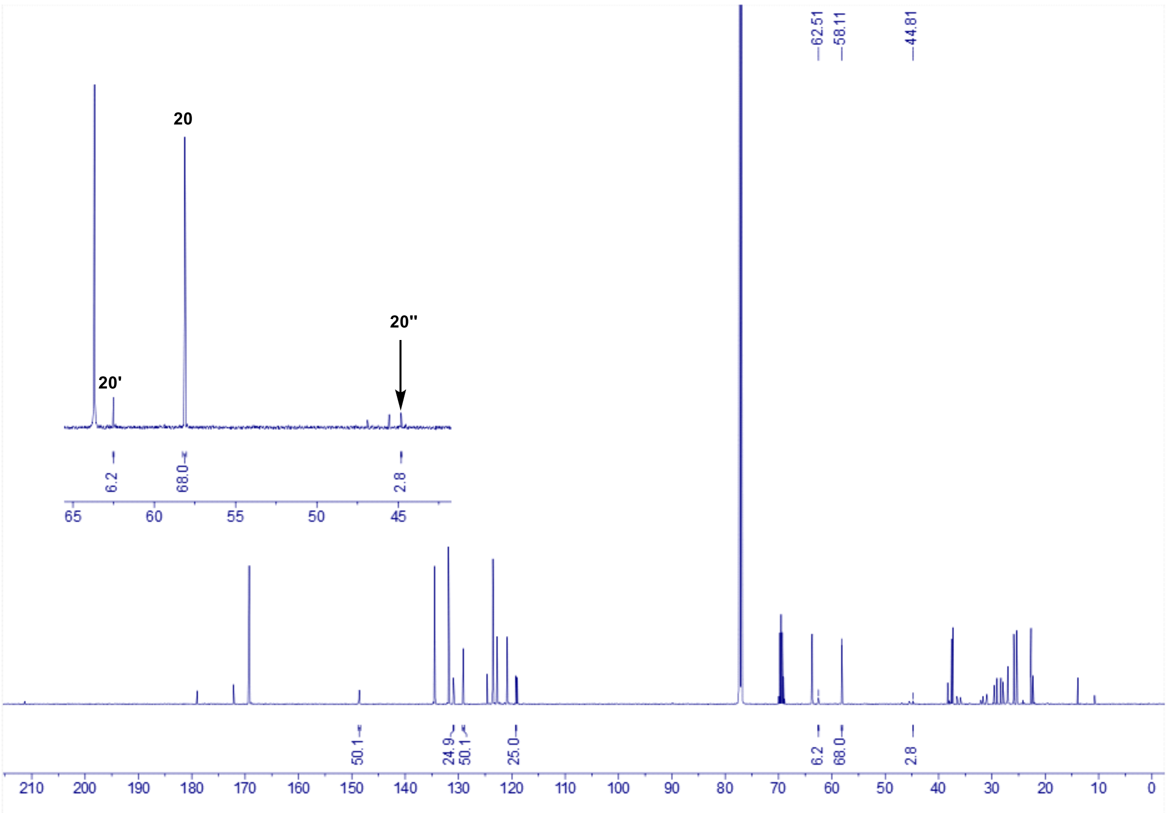

12.19 Chlorination of Pentan-1-ol to give 21

4-Chloropentan-1-ol (21)

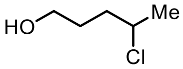

Starting Material	Observed Reaction Products		
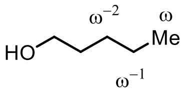	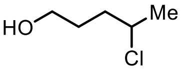 **21**	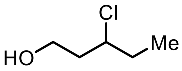 **21'**	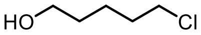 **21"**
**ω–1:ω–2:ω**	**ω–1 HAT Selectivity (%)**		
30:4.2:1.0	85		

Following **GP3** using **A3** as the amine and HFIP as the solvent, pentan-1-ol (22 µL, 0.2 mmol) gave **21**, **21’** and **21’’** in 50% yield. MS (APCI POS): 123.1 (MH+).

21: Data in accordance with the literature.[28]

21’: Structural assignment done by analogy with similar compounds and distinctive $^{35/37}Cl$ carbon peak shoulder.

21’’: Data in accordance with the literature.[29]

^{1}H NMR (600 MHz, $CDCl_3$)

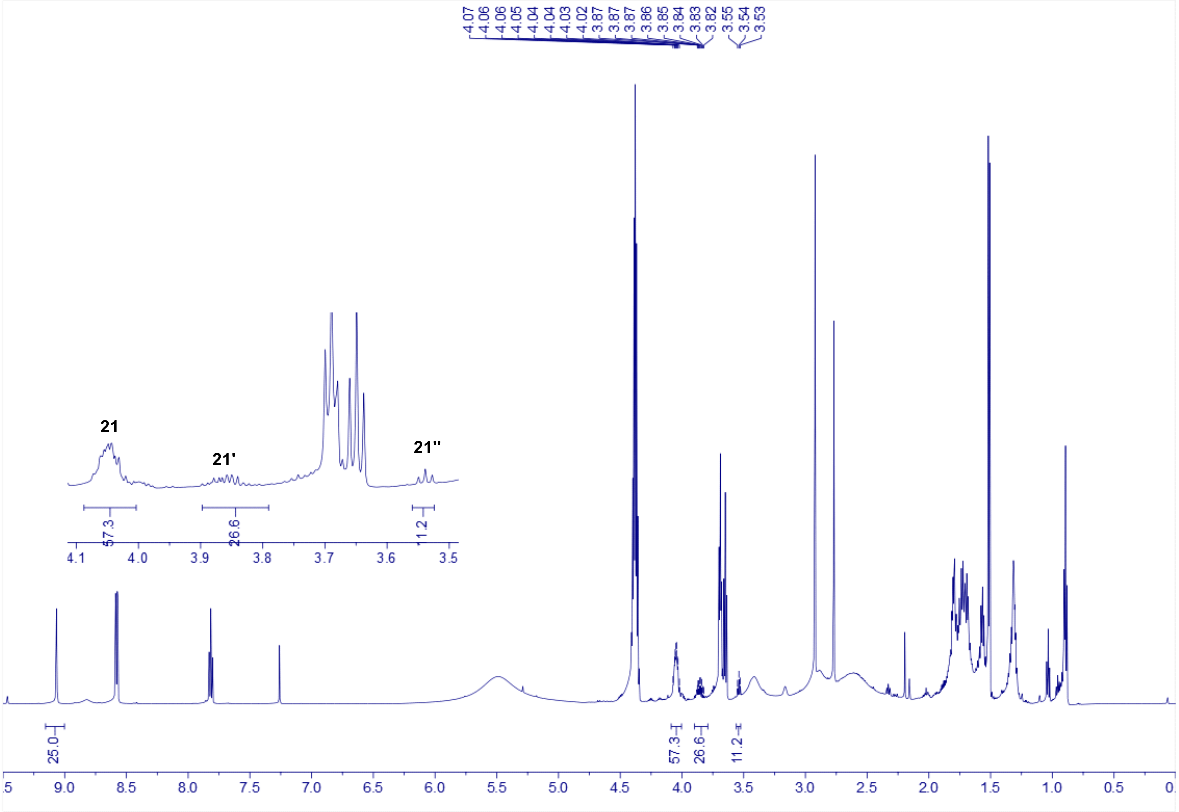

^{13}C Quantitative NMR (151 MHz, $CDCl_3$)

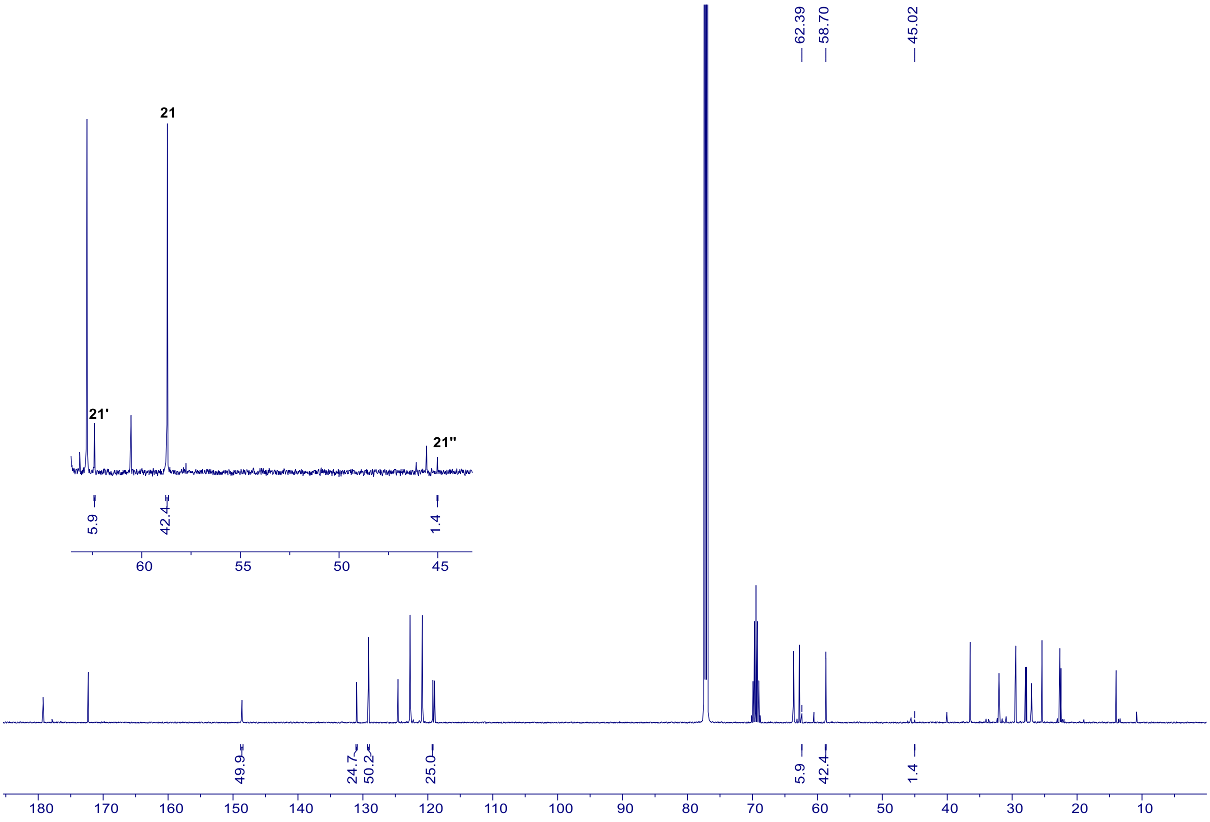

12.20 Chlorination of 1-Methoxypentane to Give 22

4-Chloro-1-methoxypentane (22)

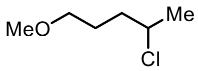

Starting Material	Observed Reaction Products
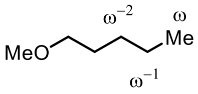	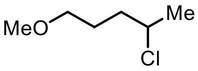 **22** 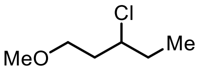 **22'** 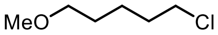 **22"**
**ω–1:ω–2:ω**	**ω–1 HAT Selectivity (%)**
22:3.9:1.0	82

Following **GP3** using **A1** as the amine and HFIP as the solvent, 1-methoxypentane (27 μL, 0.2 mmol) gave **22**, **22'** and **22''** in 60% yield. GC-MS m/z (EI): 136.1 (M+).

22, **22'**: Structural assignment done by analogy with similar compounds and distinctive $^{35/37}$Cl carbon peak shoulder.

22'': Data in accordance with the literature.[30]

^{1}H NMR (600 MHz, $CDCl_3$)

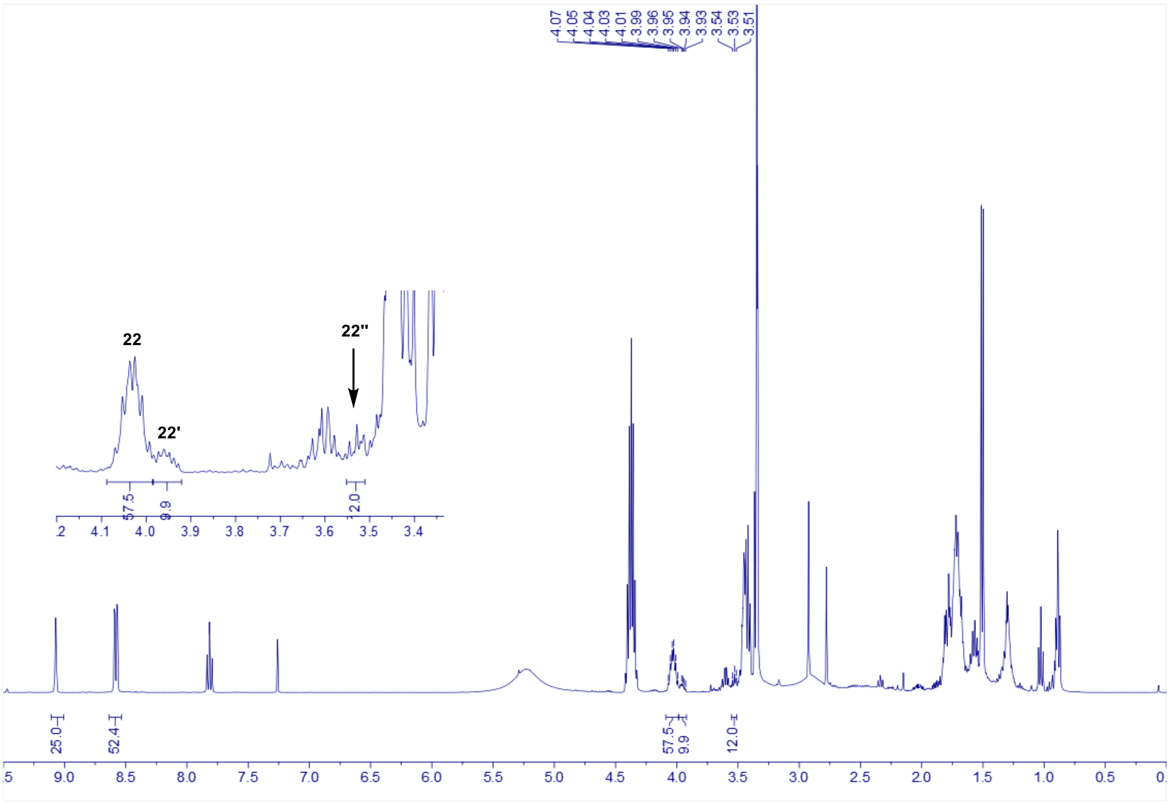

^{13}C Quantitative NMR (151 MHz, $CDCl_3$)

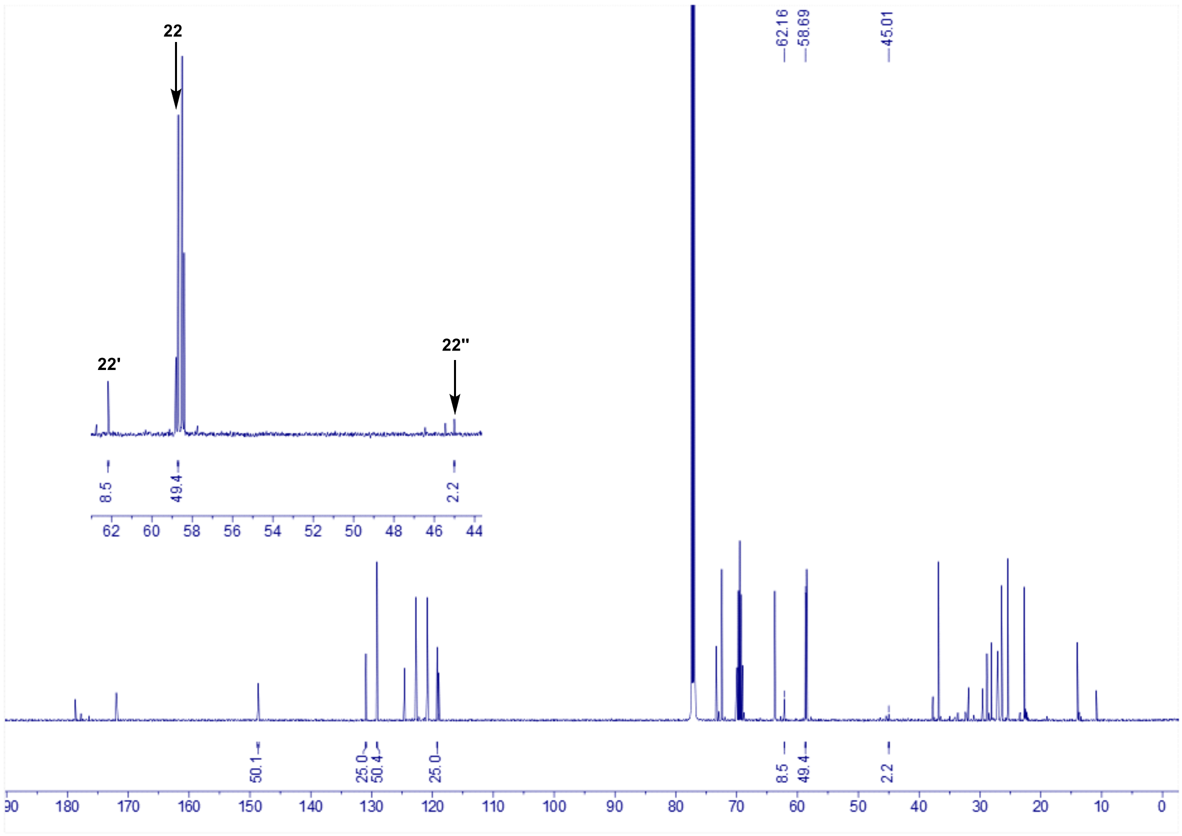

12.21 Chlorination of *N*,*N*-Dimethylpentylamine to Give 23

4-Chloro-*N*,*N*-dimethylpentan-1-amine (23)

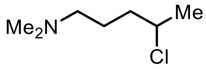

Starting Material	Observed Reaction Products
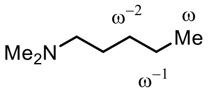	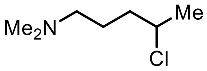 **23** 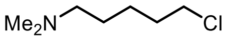 **23'** 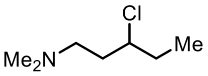 **23"**
**ω–1:ω :ω–2**	**ω–1 HAT Selectivity (%)**
9.7:1.7:1.0	78

Following **GP3** using **A3** as the amine and CH_2Cl_2 as the solvent, *N*,*N*,*N*-dimethylpentylamine (31 μL, 0.2 mmol) gave **23**, **23'** and **23''** in 47% yield. In this case a modified work-up was required: 1,3-dinitrobenzene (8.4 mg, 0.25 equiv., 0.1 M solution in $CDCl_3$) was charged and the mixture was passed through an anhydrous $MgSO_4$ plug directly into an NMR tube for ^1H and ^{13}C NMR spectroscopy analysis. GC-MS m/z (EI): 149.1 (M+).

23, **23'**, **23''**: Structural assignment done by analogy with similar compounds and distinctive $^{35/37}Cl$ carbon peak shoulder.

^{1}H NMR (600 MHz, $CDCl_3$)

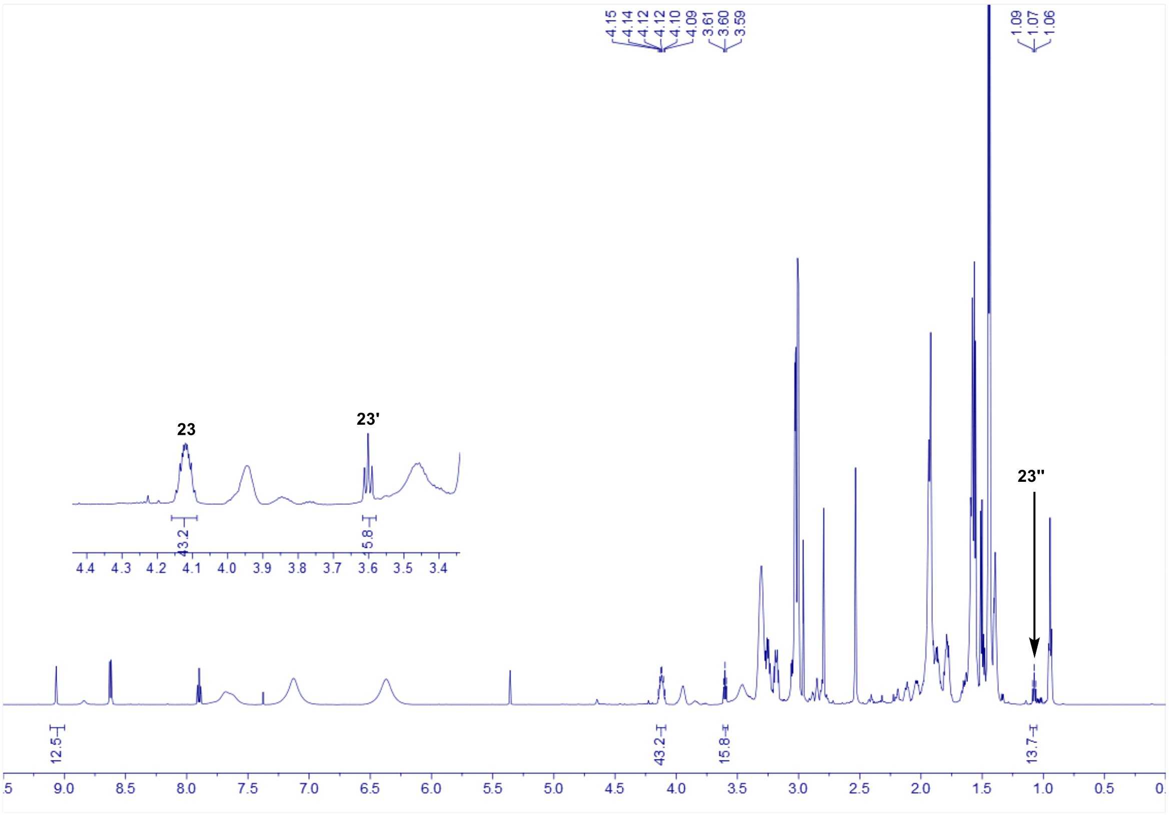

^{13}C Quantitative NMR (151 MHz, $CDCl_3$)

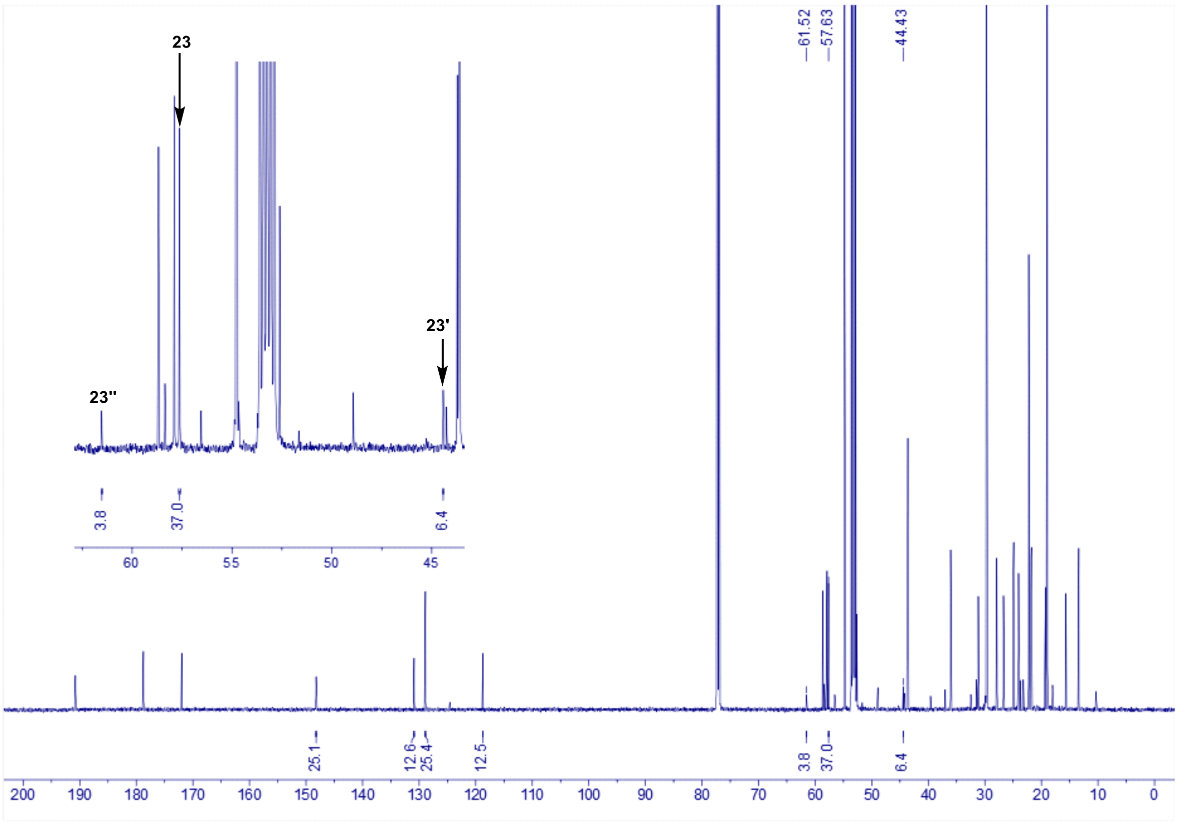

12.22 Chlorination of Hexanoic Acid to Give 24

5-Chlorohexanoic Acid (24)

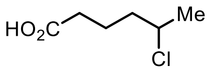

Starting Material	Observed Reaction Products
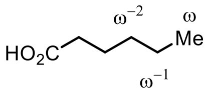	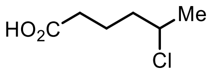 **24** 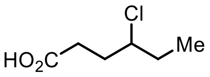 **24'** 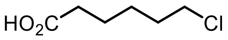 **24"**
**ω–1:ω–2:ω**	**ω–1 HAT Selectivity (%)**
8.4:1.1:1.0	80

Following **GP3** using **A3** as the amine and HFIP as the solvent, hexanoic acid (25 μL, 0.2 mmol) gave **24**, **24'** and **24''** in 41% yield. MS (APCI POS): 151.1 (MH^+).

24, **24'**: Structural assignment done by analogy with similar compounds and distinctive $^{35/37}Cl$ carbon peak shoulder.
24'': Data in accordance with the literature.[31]

^{1}H NMR (600 MHz, $CDCl_3$)

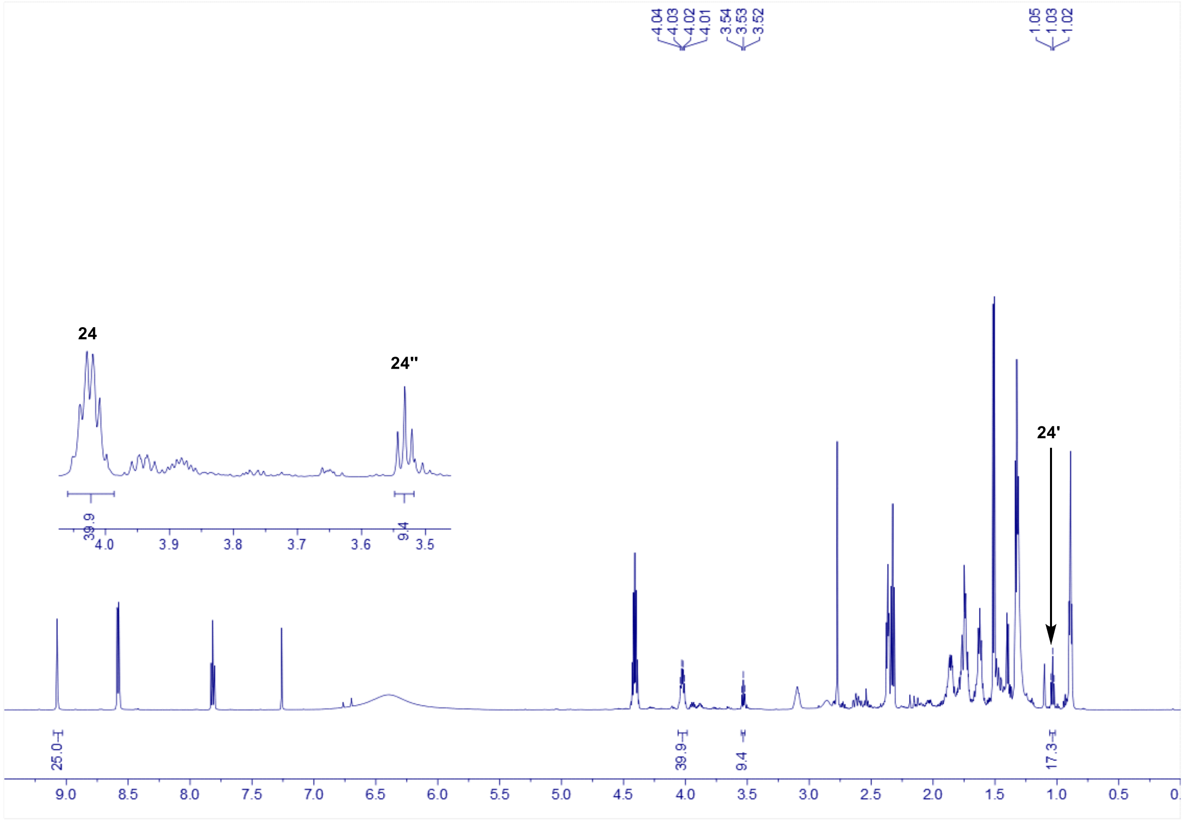

^{13}C Quantitative NMR (151 MHz, $CDCl_3$)

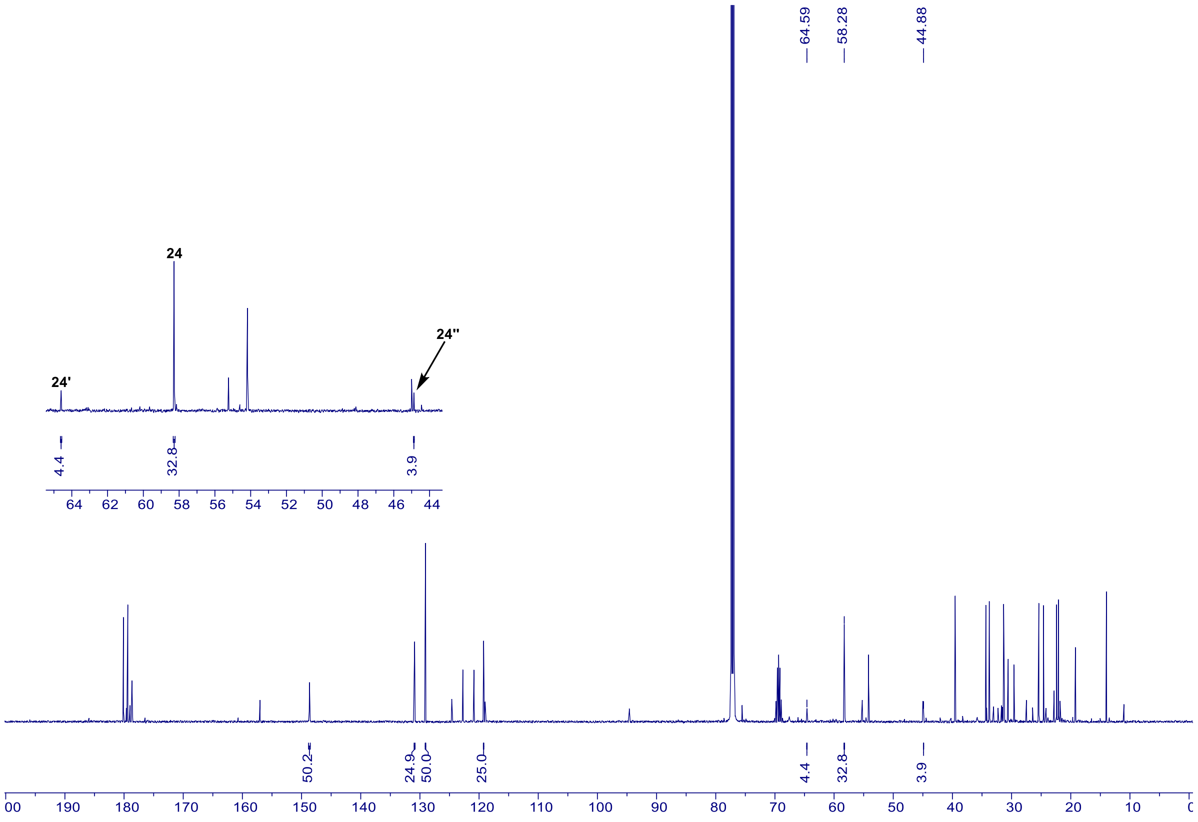

12.23 Chlorination of Pentylbenzene to Give 25

(1-Chloropentyl)benzene (25)

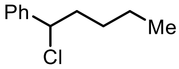

Starting Material	Observed Reaction Products	HAT Selectivity (%)
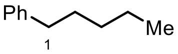	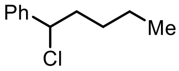 **25**	100

Following **GP4** using **A3** as the amine and CH_2Cl_2 as the solvent, 1-phenylpentane (34 µL, 0.2 mmol) gave **25** in 84% yield. GC-MS m/z (EI): 182.1 (M+).

25: Data in accordance with the literature.[32]

^{1}H NMR (600 MHz, $CDCl_3$)

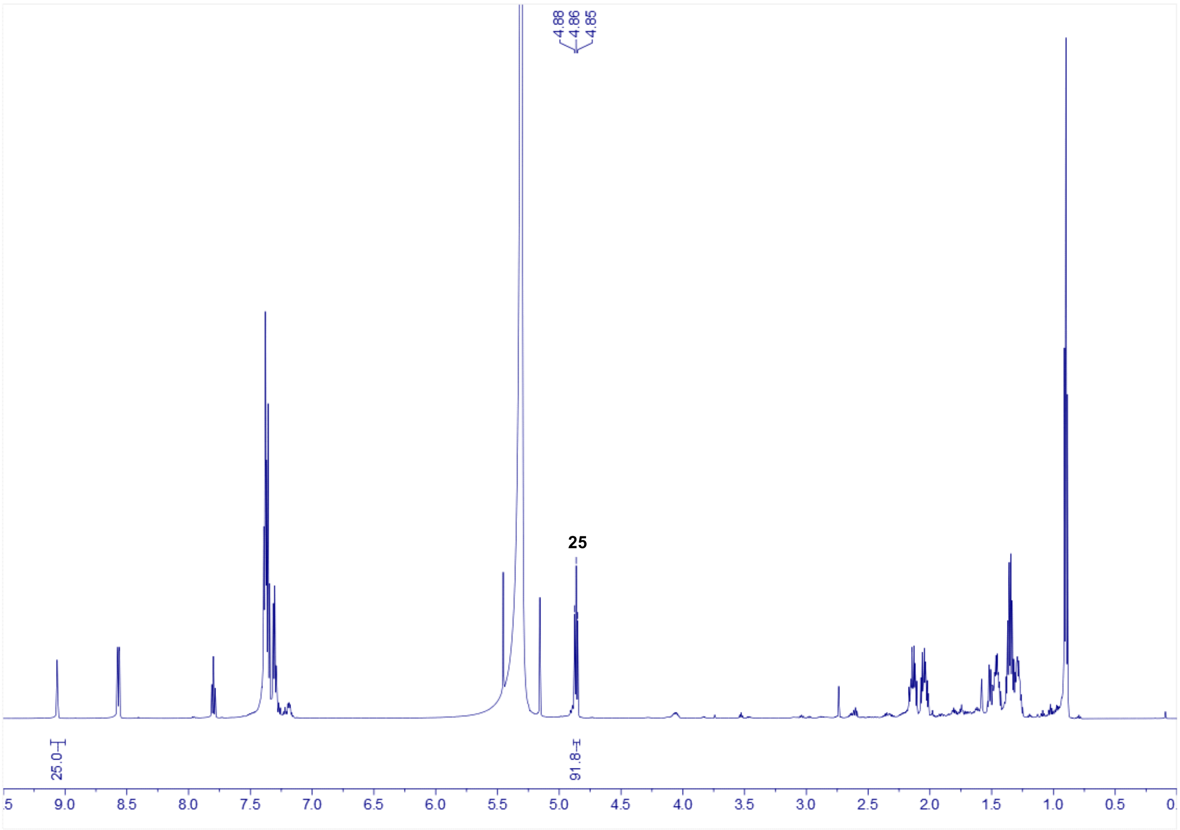

^{13}C Quantitative NMR (151 MHz, $CDCl_3$)

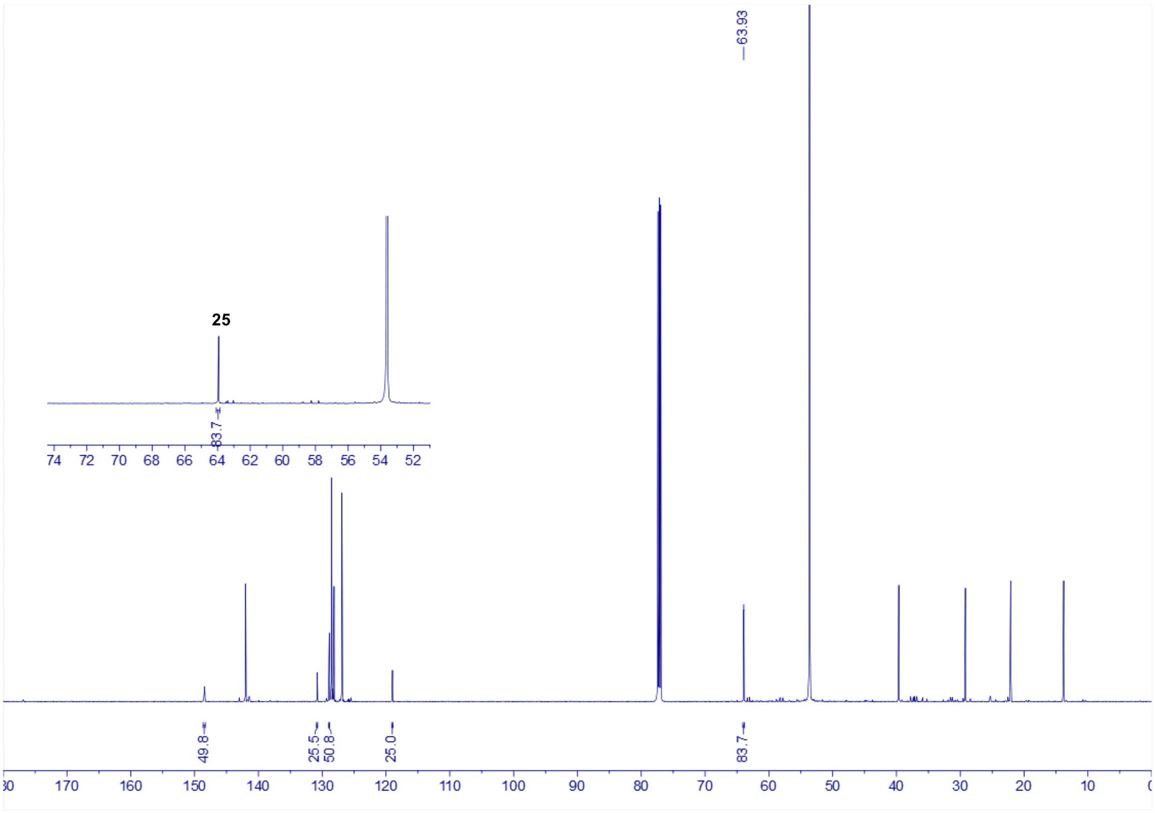

12.24 Chlorination of 2-Pentylpyridine to Give 26

2-(4-Chloropentyl)pyridine (26)

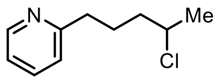

Starting Material	Observed Reaction Products
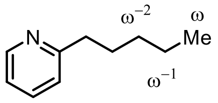	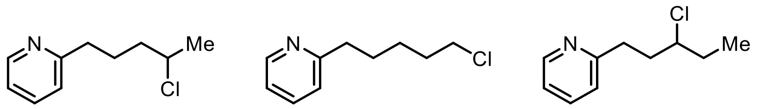 **26** **26'** **26''**
**ω–1:ω :ω–2:ω–3**	**ω–1 HAT Selectivity (%)**
16:1.2: 1.0	88

Following **GP3** using **A3** as the amine and CH_2Cl_2 as the solvent, 2-pentyl pyridine (34 μL, 0.2 mmol) gave **26**, **26'**, **26''** and **26'''** in 75% yield. In this case a modified work-up was required: after irradiation H_2O (2 mL) and 1 M KOH (2 mL) were added followed by 1,3-dinitrobenzene (8.4 mg, 0.25 equiv., 0.1 M solution in $CDCl_3$). The layers were separated and the organic phase was passed through an anhydrous $MgSO_4$ plug directly into an NMR tube for ^{1}H and ^{13}C NMR spectroscopy analysis. GC-MS m/z (EI): 183.1 (M+).

26, **26''**, **26'''**: Structural assignment done by analogy with similar compounds and distinctive $^{35/37}$Cl carbon peak shoulder.

26': Data in accordance with the literature.[33]

^{1}H NMR (600 MHz, $CDCl_3$)

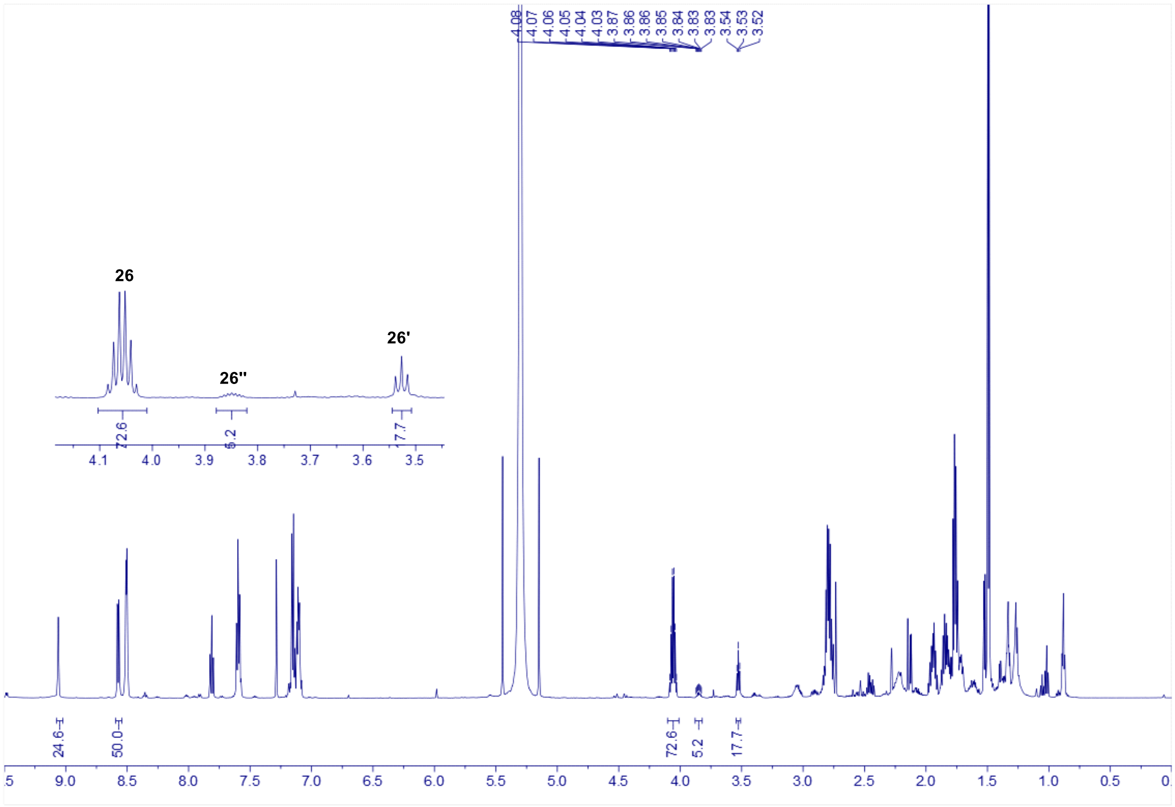

^{13}C Quantitative NMR (151 MHz, $CDCl_3$)

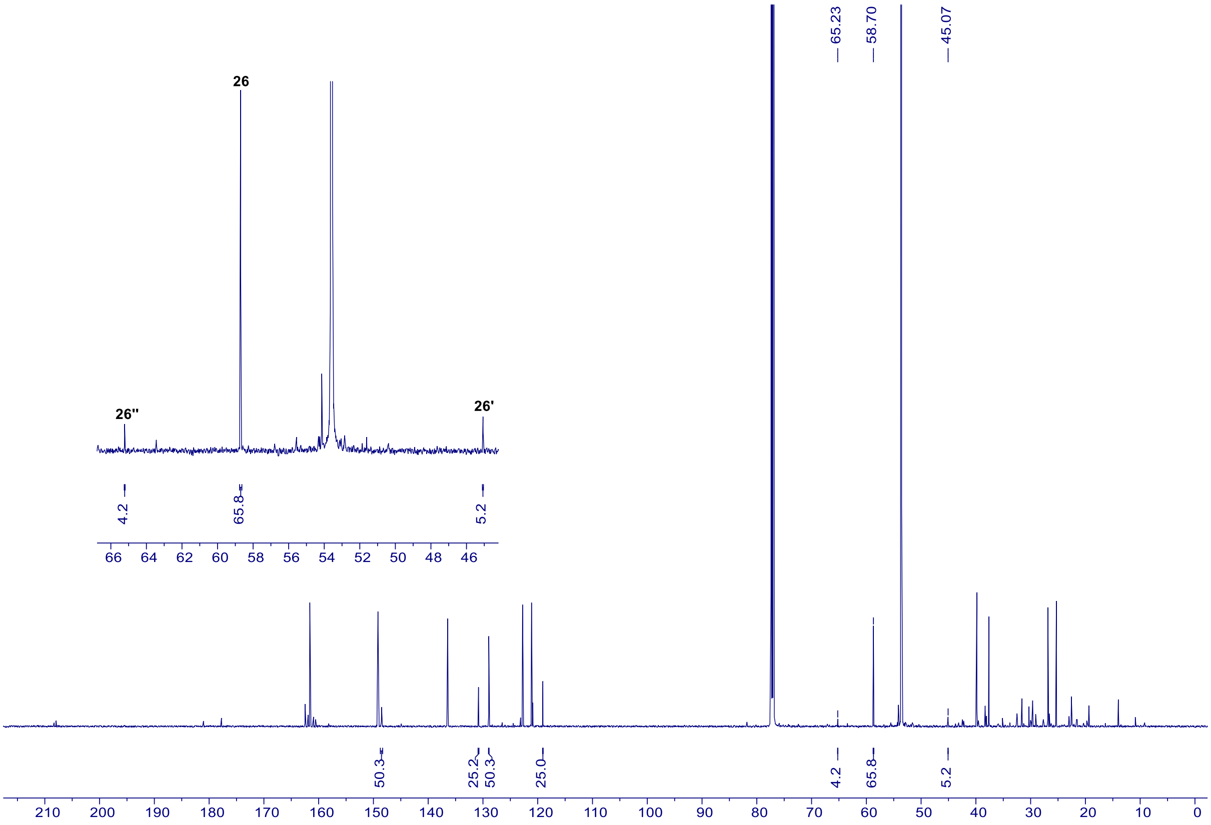

12.25 Chlorination of 3-Pentylpyridine to Give 27

3-(4-Chloropentyl)pyridine (27)

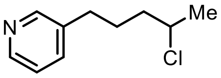

**Starting Material**	**Observed Reaction Products**
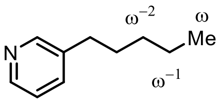	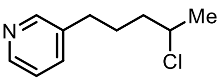 **27** 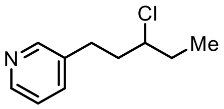 **27'** 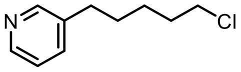 **27''**
**ω–1:ω–2:ω**	**ω–1 HAT Selectivity (%)**
11:1.5:1.0	81

Following **GP3** using **A3** as the amine and CH_2Cl_2 as the solvent, 3-pentyl pyridine (34 μL, 0.2 mmol) gave **27**, **27’** and **27’’** in 55% yield. In this case a modified work-up was required: after irradiation H_2O (2 mL) and 1 M KOH (2 mL) were added followed by 1,3-dinitrobenzene (8.4 mg, 0.25 equiv., 0.1 M solution in $CDCl_3$). The layers were separated and the organic phase was passed through an anhydrous $MgSO_4$ plug directly into an NMR tube for ^{1}H and ^{13}C NMR spectroscopy analysis. GC-MS m/z (EI): 183.1 (M+).

27, **27’**, **27’’**: Structural assignment done by analogy with similar compounds and distinctive $^{35/37}Cl$ carbon peak shoulder.

^{1}H NMR (600 MHz, $CDCl_3$)

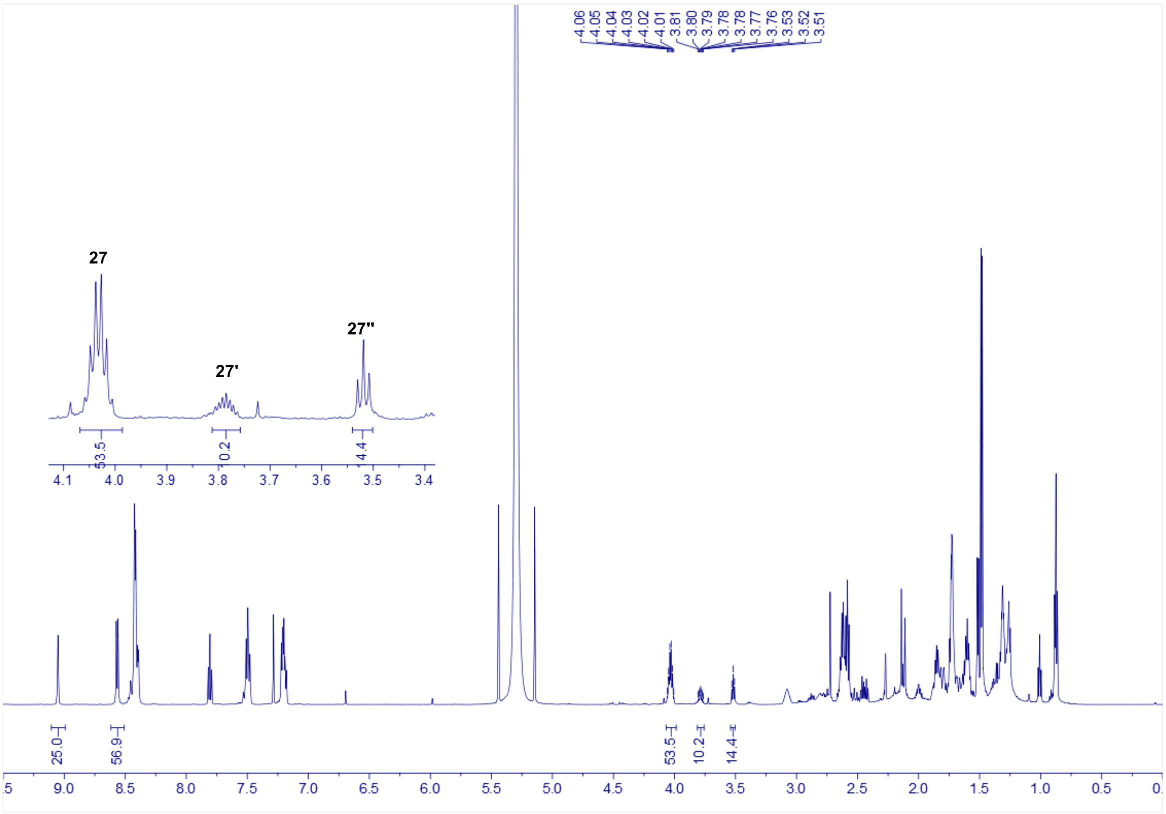

^{13}C NMR (151 MHz, $CDCl_3$)

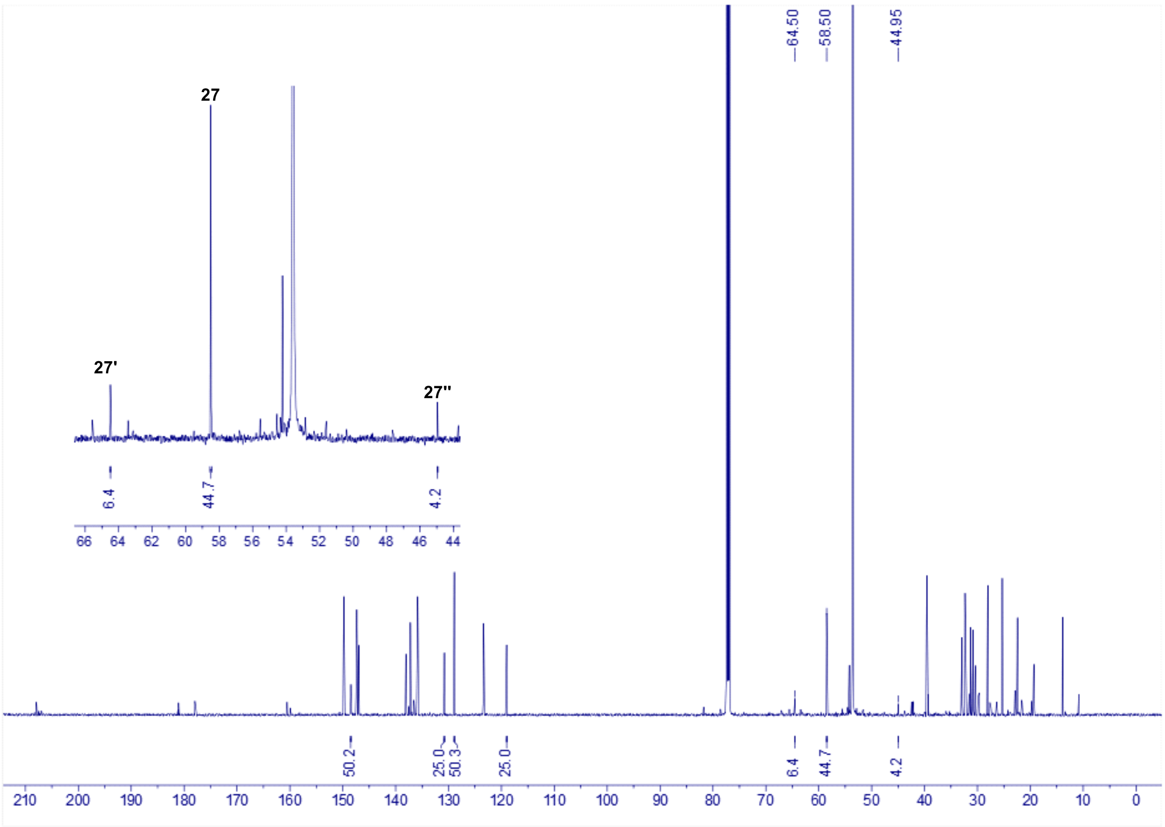

12.26 Chlorination of 4-Pentylpyridine to Give 28

4-(4-Chloropentyl)pyridine (28)

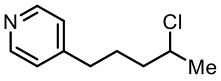

**Starting Material**	**Observed Reaction Products**
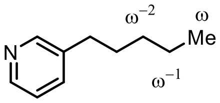	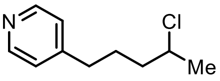 **28** 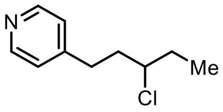 **28'** 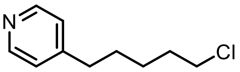 **28"**
**ω–1:ω–2:ω**	**ω–1 HAT Selectivity (%)**
13:1.2:1.0	85

Following **GP3** using **A3** as the amine and CH_2Cl_2 as the solvent, 3-pentyl pyridine (33 μL, 0.2 mmol) gave **28**, **28’** and **28’’** in 42% yield. In this case a modified work-up was required: after irradiation H2O (2 mL) and 1 M KOH (2 mL) were added followed by 1,3-dinitrobenzene (8.4 mg, 0.25 equiv., 0.1 M solution in $CDCl_3$). The layers were separated and the organic phase was passed through an anhydrous $MgSO_4$ plug directly into an NMR tube for ^{1}H and ^{13}C NMR spectroscopy analysis. GC-MS m/z (EI): 183.1 (M+).

28, **28’**, **28’’**: Structural assignment done by analogy with similar compounds and distinctive $^{35/37}$Cl carbon peak shoulder.

^{1}H NMR (600 MHz, $CDCl_3$)

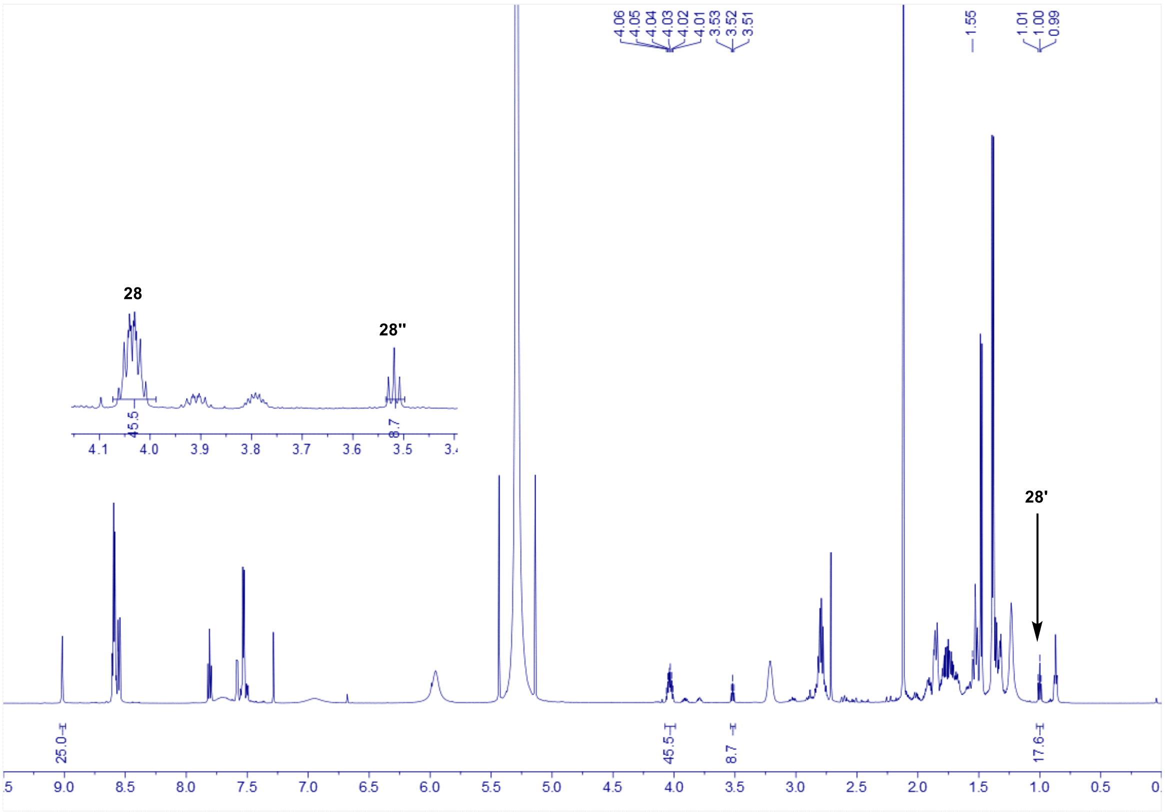

^{13}C NMR (151 MHz, $CDCl_3$)

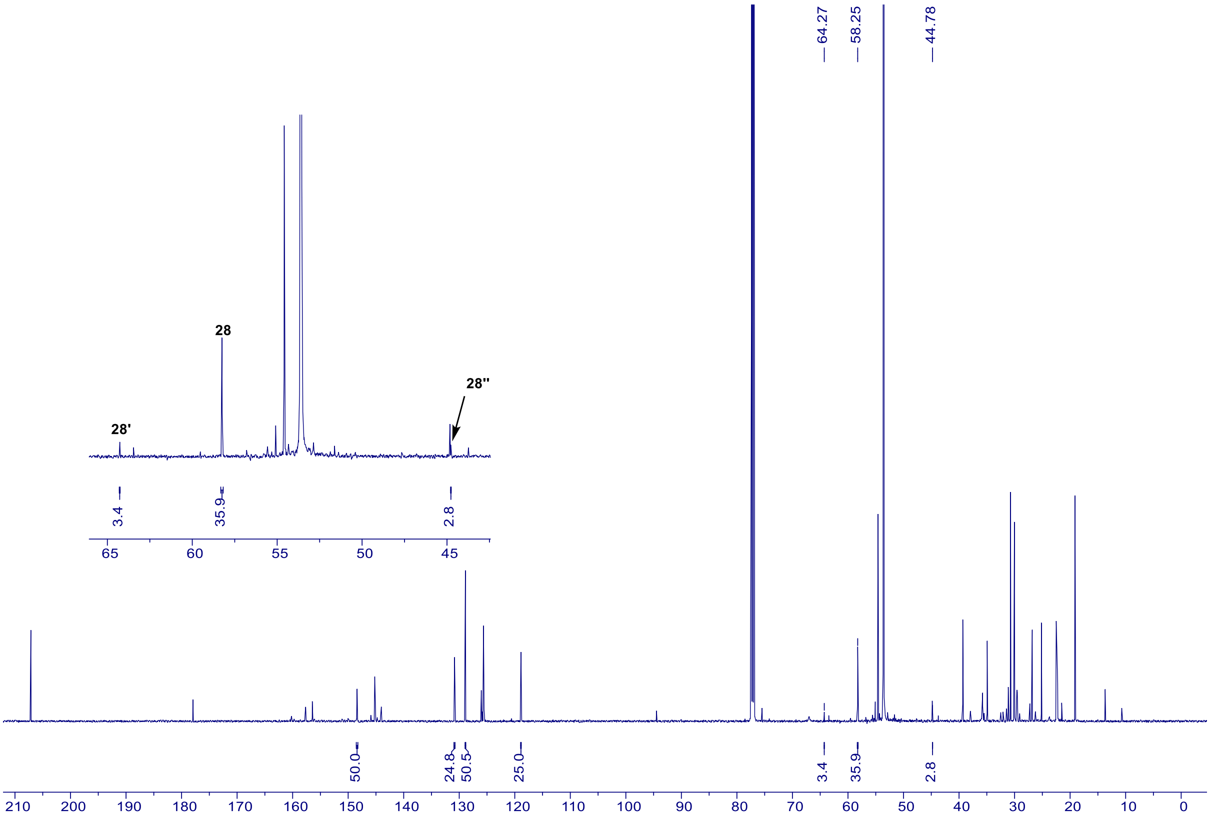

12.27 Chlorination of 4-Phenylbutanoic Acid to Give 29

4-Chloro-4-phenylbutanoic Acid (29)

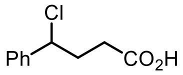

Starting Material	Observed Reaction Products	HAT Selectivity (%)
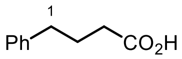	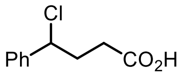 **29**	100

Following **GP3** using **A3** as the amine and CH_2Cl_2 as the solvent, 4-phenylbutanoic acid (33 mg, 0.2 mmol) gave **29** in 76% yield. GC-MS m/z (EI): 198.0 (M+).

29: Data in accordance with the literature.[34]

^{1}H NMR (600 MHz, $CDCl_3$)

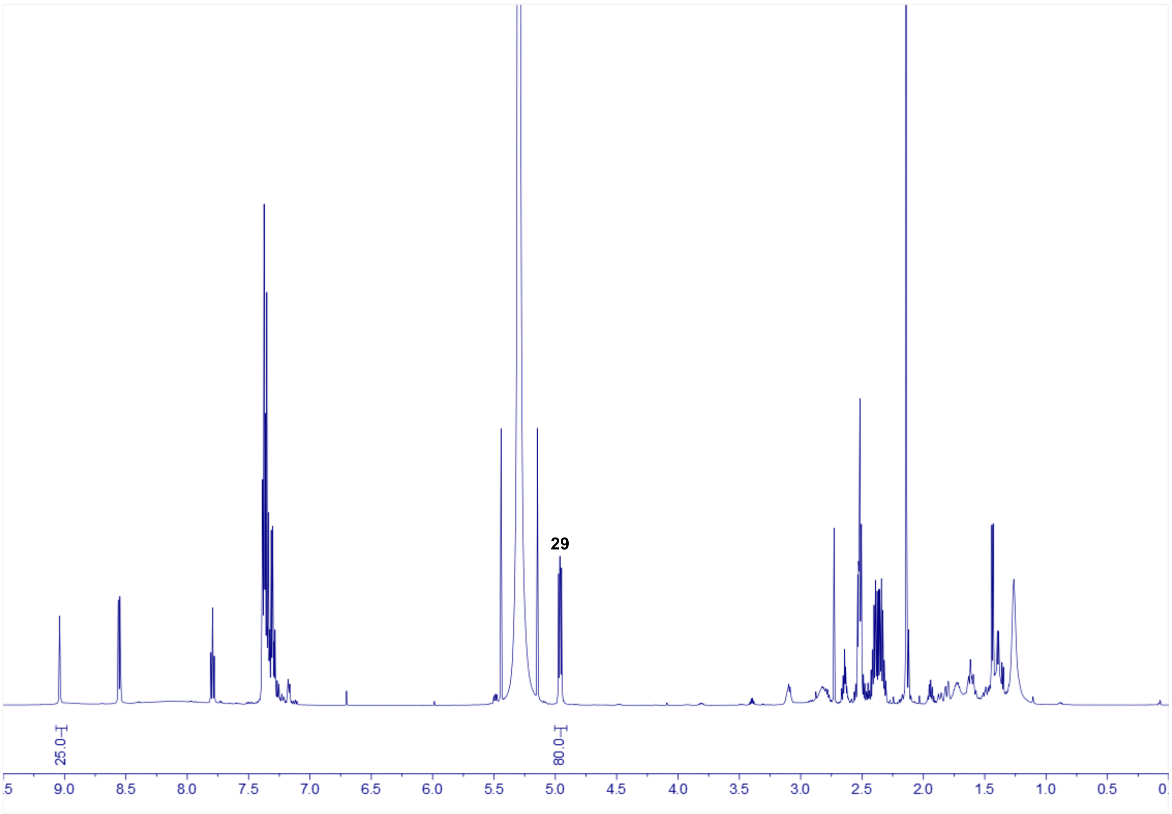

^{13}C Quantitative NMR (151 MHz, $CDCl_3$)

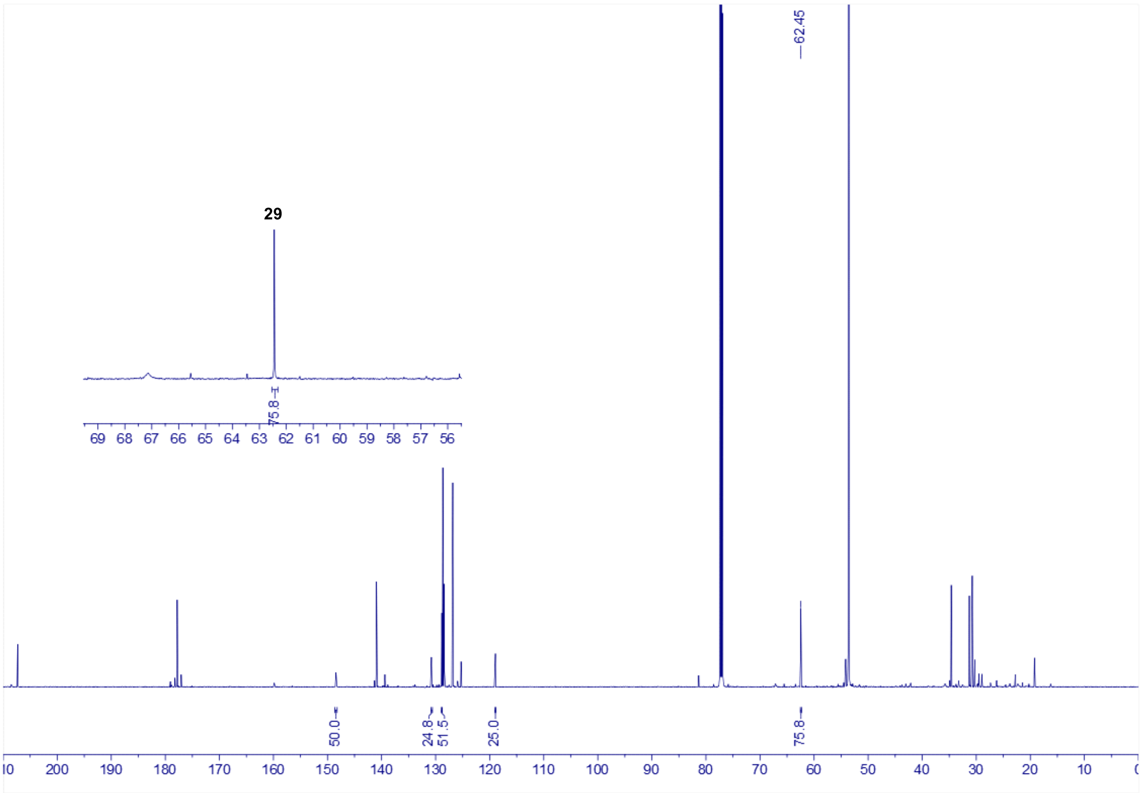

12.28 Chlorination of 3-Phenylpropanoic Acid to Give 30

3-Chloro-3-phenylpropanoic Acid (30)

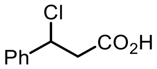

Starting Material	Observed Reaction Products	HAT Selectivity (%)
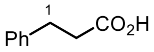	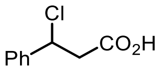 **30**	100

Following **GP3** using **A3** as the amine and CH_2Cl_2 as the solvent, 3-phenylpropanoic acid (30 mg, 0.2 mmol) gave **30** in 77% yield. GC-MS m/z (EI): 184.0 (M+).

30: Data in accordance with the literature.[35]

^{1}H NMR (600 MHz, $CDCl_3$)

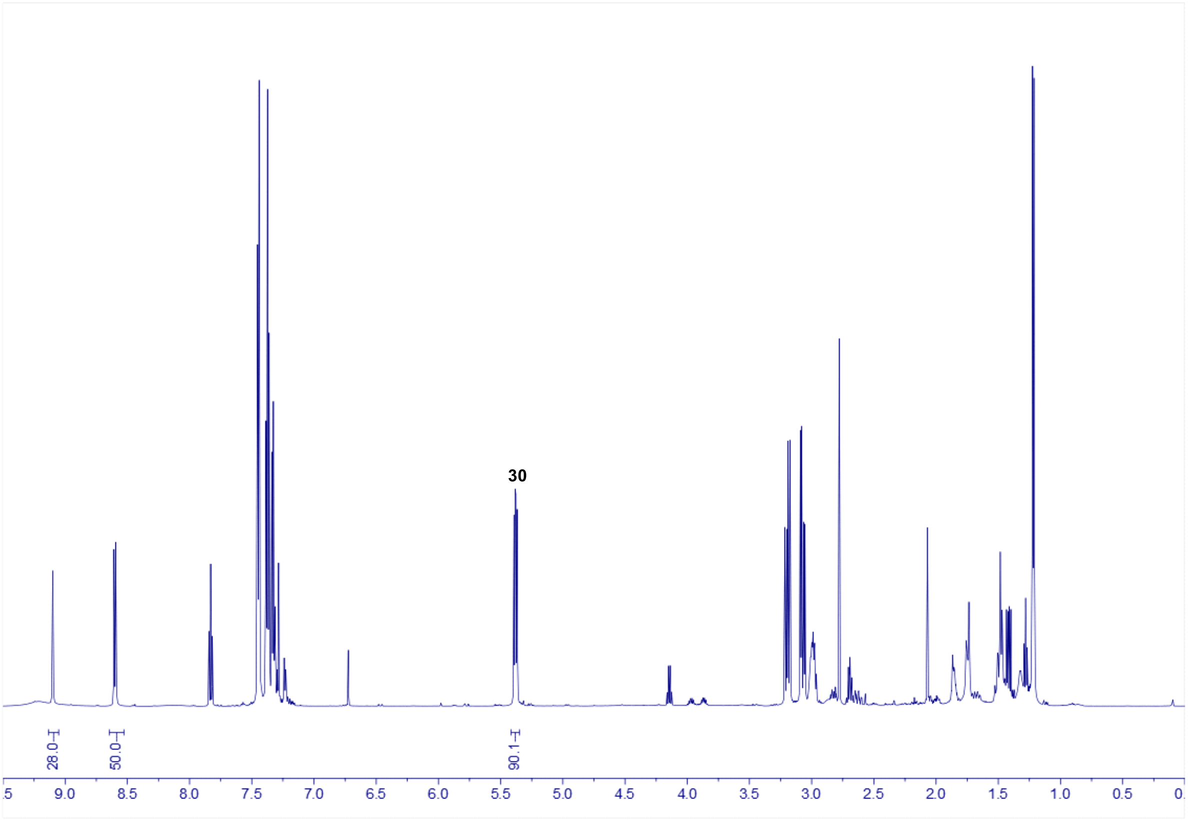

^{13}C NMR (151 MHz, $CDCl_3$)

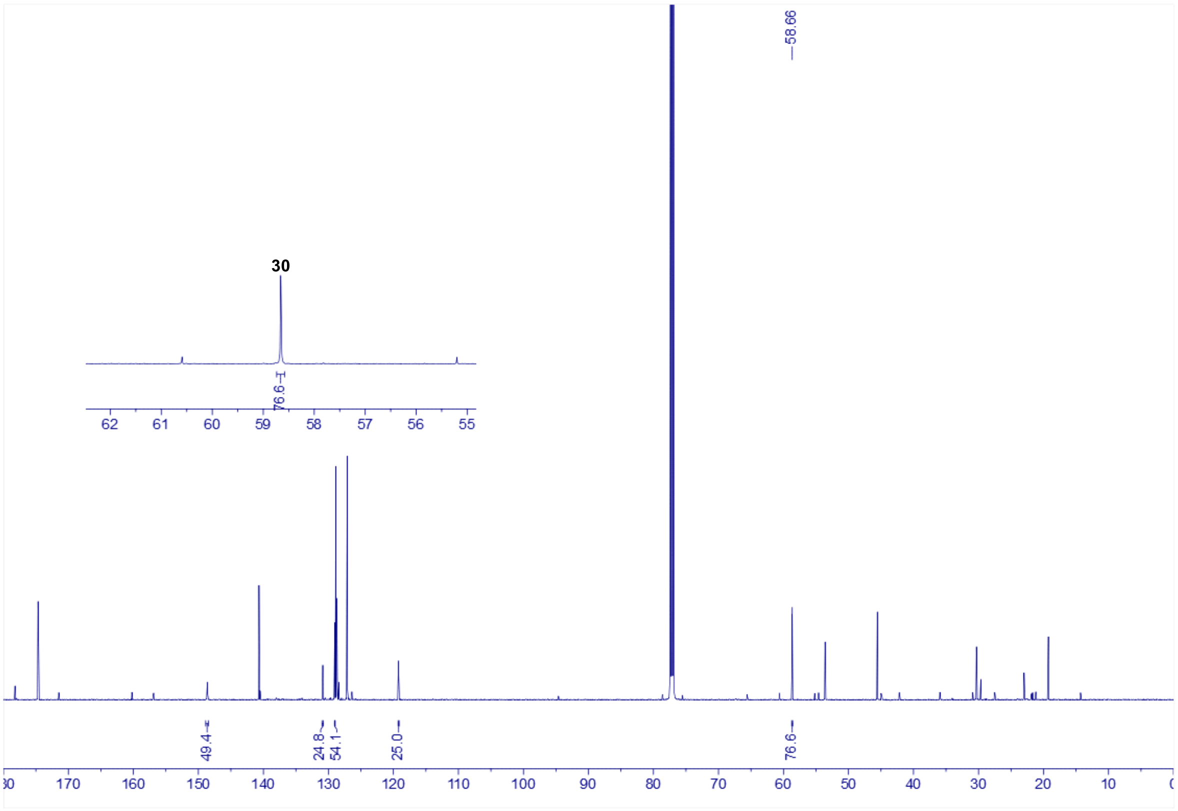

12.29 Chlorination of Octanoic Acid to Give 31

7-Chlorooctanoic acid (31)

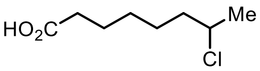

Starting Material	Observed Reaction Products
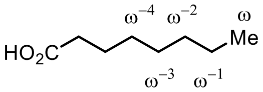	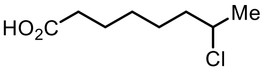 **31** 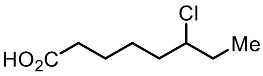 **31'** 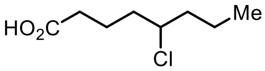 **31''** 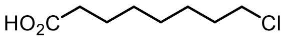 **31'''** 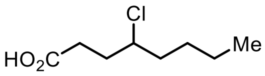 **31''''**
**ω–1:ω–2:ω–3:ω:ω–4**	**ω–1 HAT Selectivity (%)**
25:7.3:3.3:1.6:1.0	66

Following **GP3** using **A3** as the amine and HFIP as solvent, octanoic acid (32 μL, 0.2 mmol) gave **31**, **31'**, **31''**, **31'''** and **31''''** in 62% yield. MS (APCI POS): 179.1 (MH+).

31, **31'**, **31''', 31''''**: Structural assignment done by analogy with similar compounds and distinctive $^{35/37}$Cl carbon peak shoulder.
31'': Data in accordance with the literature.[36]

^{1}H NMR (600 MHz, $CDCl_3$)

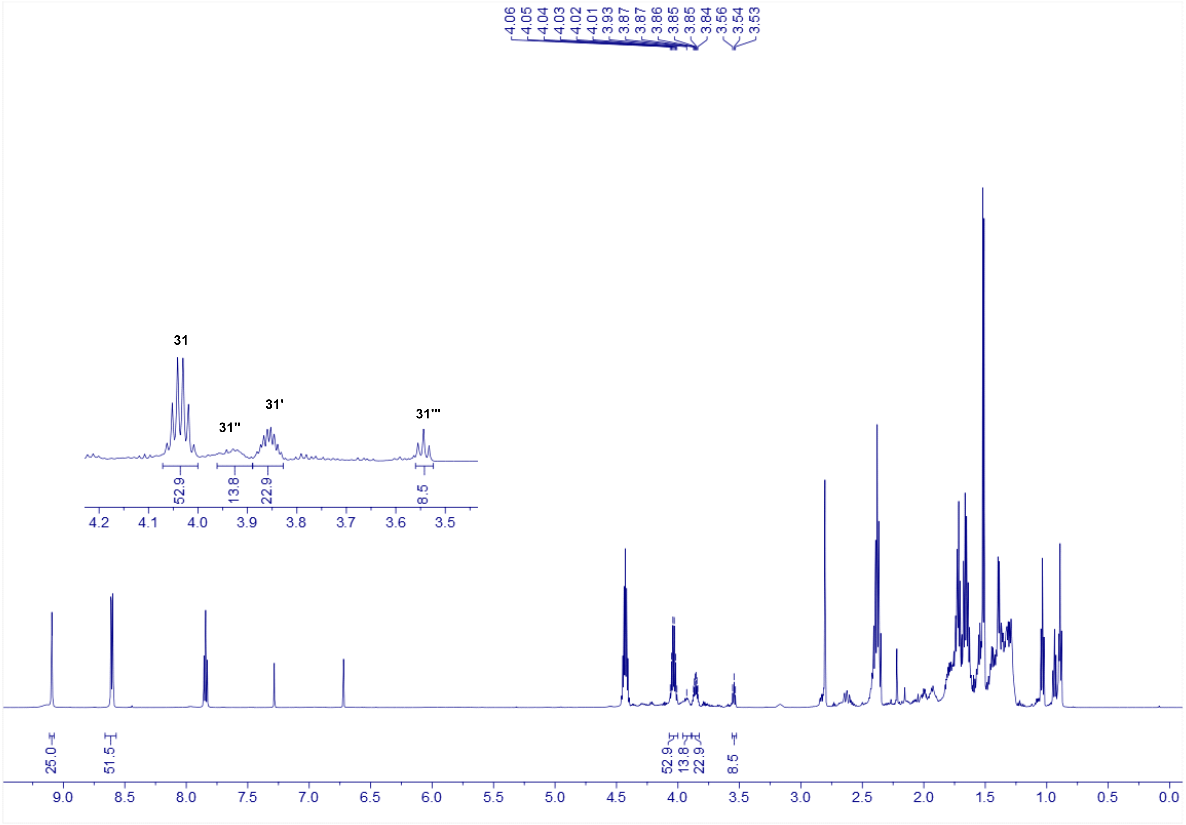

^{13}C NMR (151 MHz, $CDCl_3$)

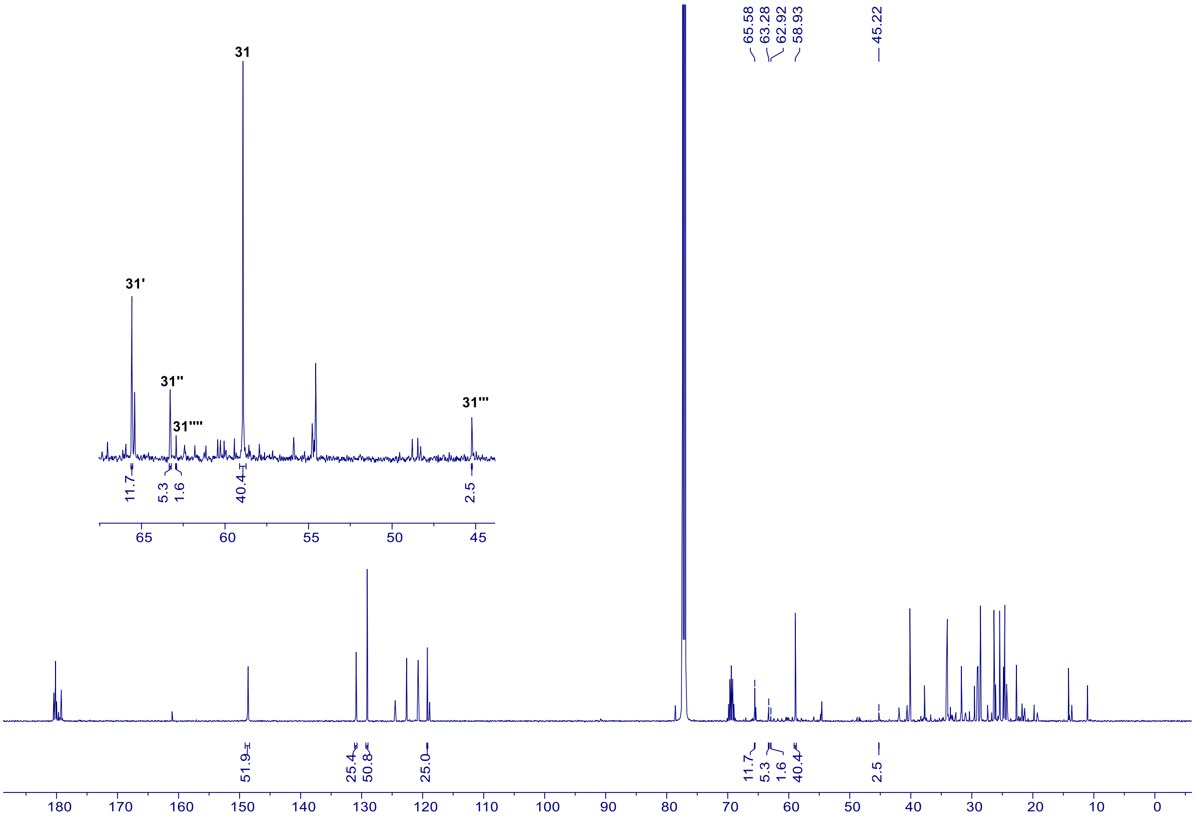

12.30 Chlorination of Pentanoic Acid to Give 32

4-Chloropentanoic acid (32)

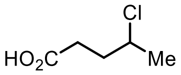

Starting Material	Observed Reaction Products		
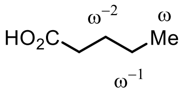	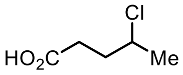 **32**	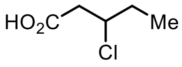 **32'**	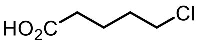 **32"**
**ω–1:ω–2:ω**	**ω–1 HAT Selectivity (%)**		
6.7:1.0:1.0	77		

Following **GP3** using **A3** as the amine and CH_2Cl_2 as the solvent, pentanoic acid (22 μL, 0.2 mmol) gave **32**, **32’** and **32’’** in 40% yield. GC-MS m/z (EI): 136.0 (M+).

32, **32’**: Structural assignment done by analogy with similar compounds and distinctive $^{35/37}$Cl carbon peak shoulder.
32’’: Data in accordance with the literature.[37]

^{1}H NMR (600 MHz, $CDCl_3$)

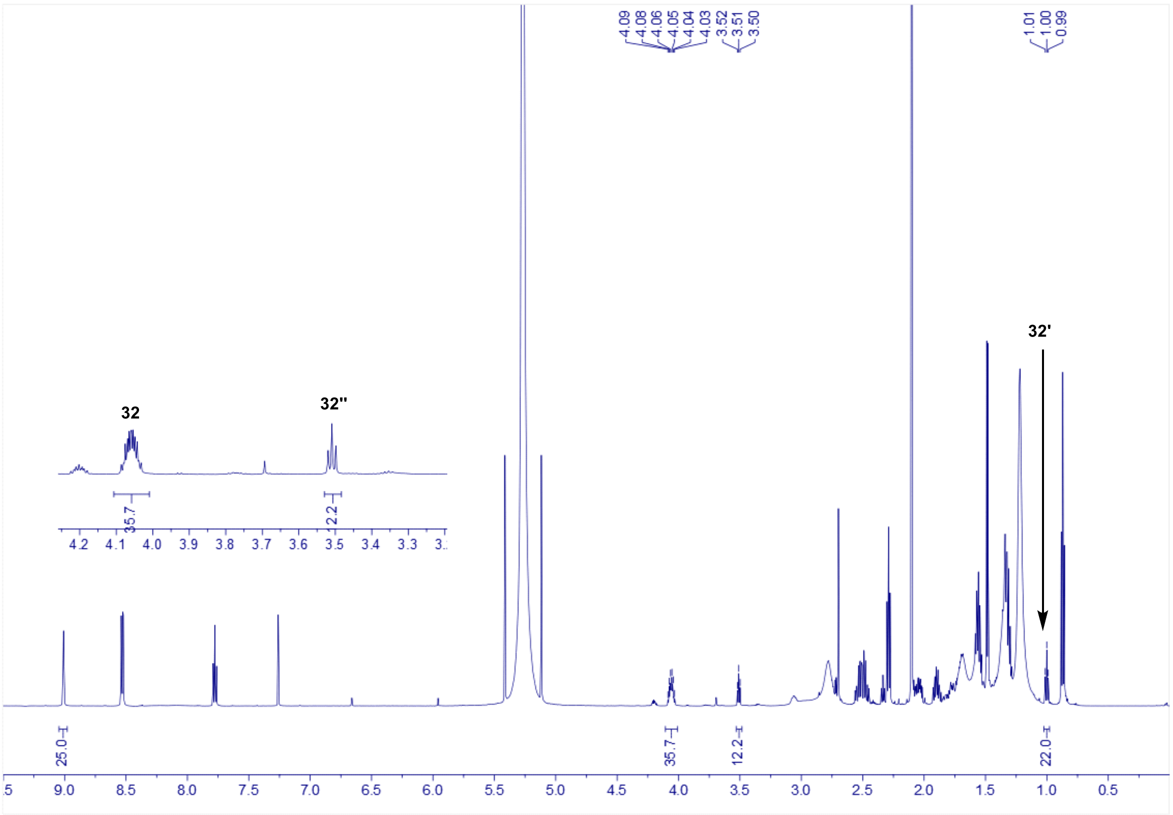

^{13}C Quantitative NMR (151 MHz, $CDCl_3$)

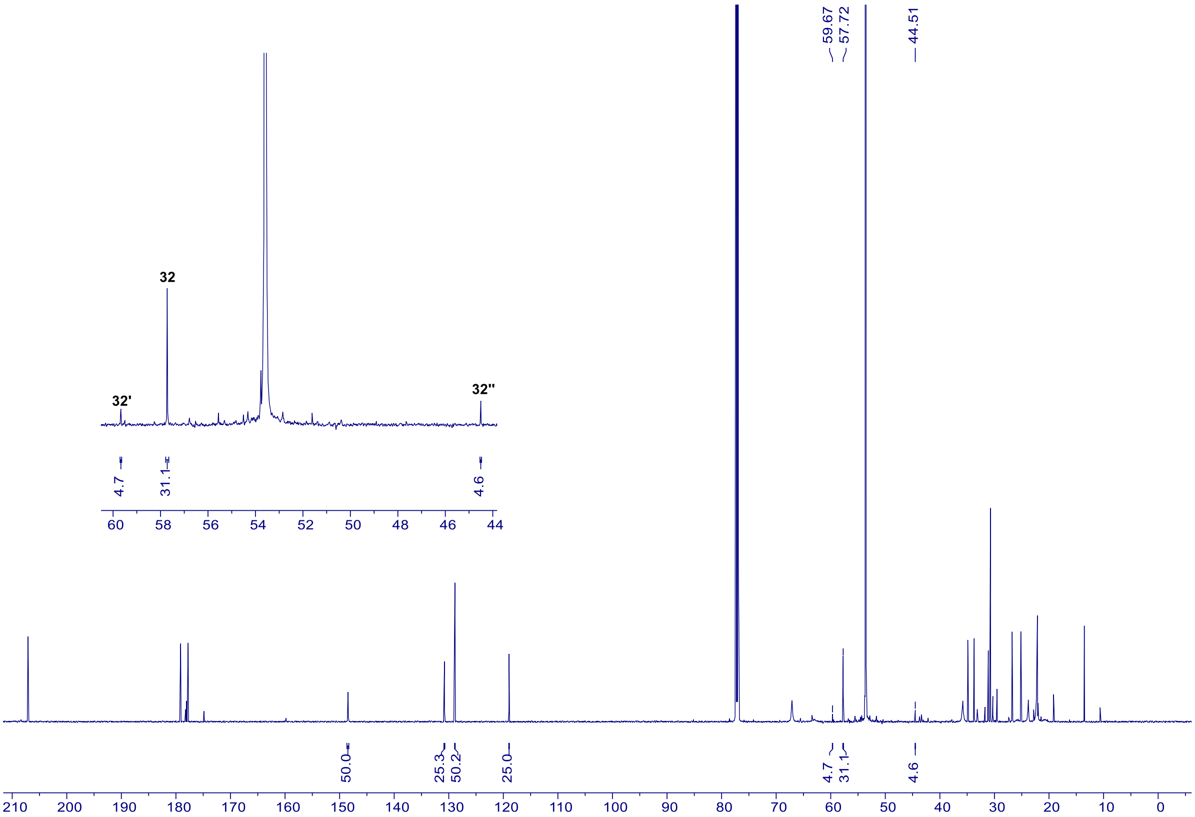

12.31 Chlorination of 1-Bromobutane to Give 33

1-Bromo-3-chlorobutane (33)

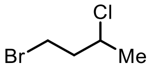

Starting Material	Observed Reaction Products
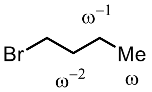	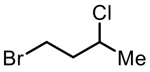 **33** 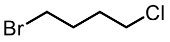 **33'** 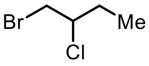 **33"**
**ω–1:ω:ω–2**	**ω–1 HAT Selectivity (%)**
9.2:1.3:1.0	80

Following **GP3** using **A3** as the amine and CH_2Cl_2 as the solvent, 1-bromobutane (21 μL, 0.2 mmol) gave **33**, **33'**and **33''** in 53% yield. GC-MS m/z (EI): 170.0 (M+).

33, **33'**: Structural assignment done by analogy with similar compounds and distinctive $^{35/37}$Cl carbon peak shoulder.
33'': Data in accordance with the literature.[38]

^{1}H NMR (600 MHz, $CDCl_3$)

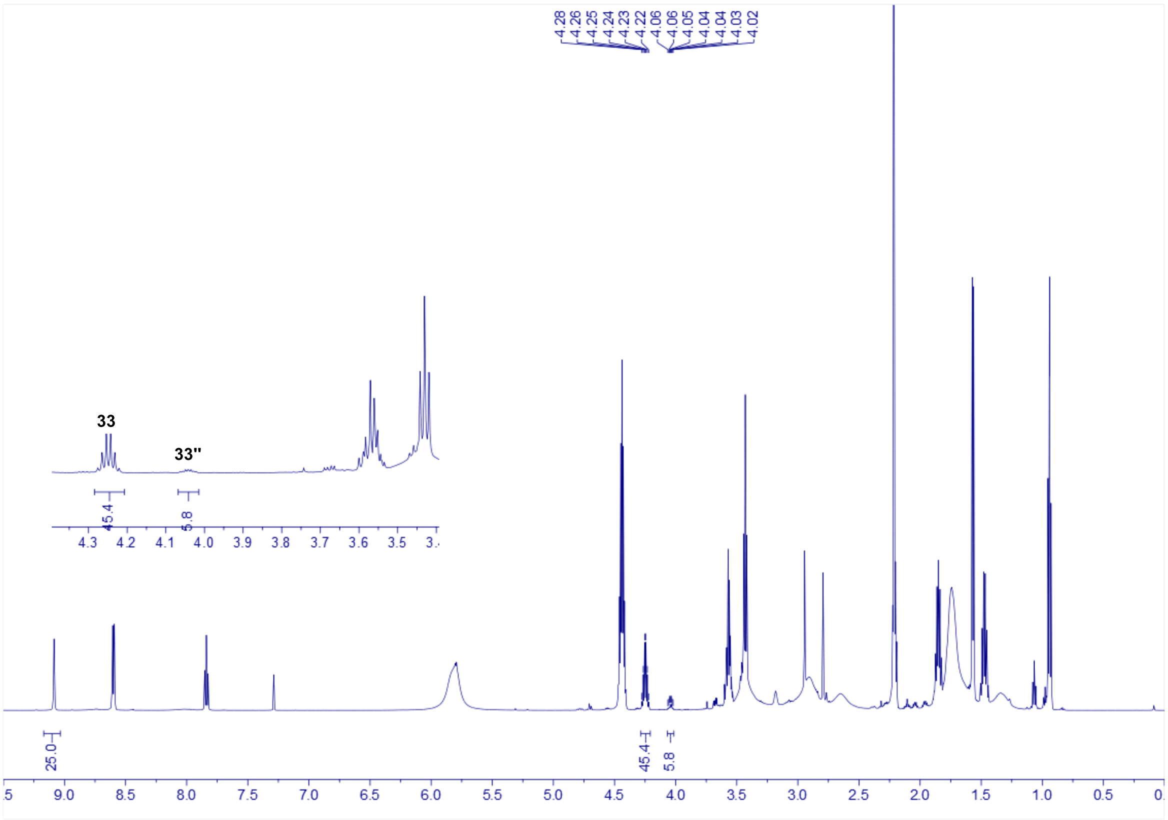

^{13}C NMR (151 MHz, $CDCl_3$)

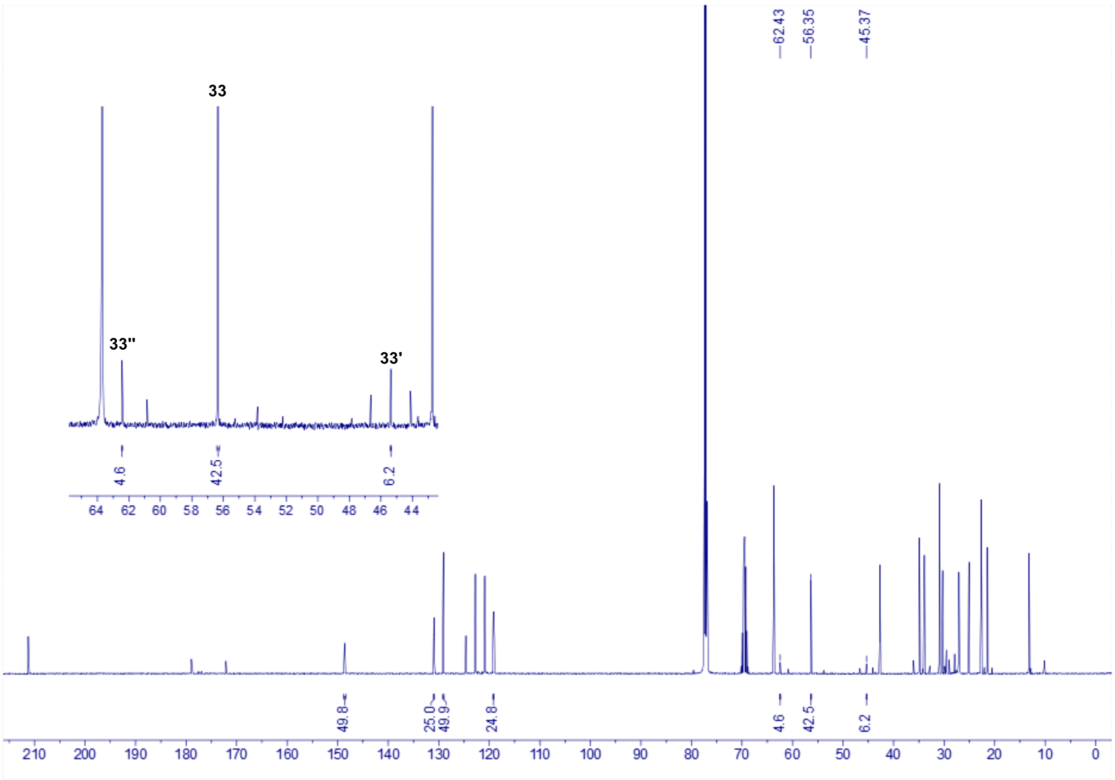

12.32 Chlorination of Butyl Acetate to Give 34

3-Chlorobutyl Acetate (34)

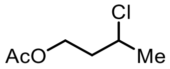

Starting Material	Observed Reaction Products		
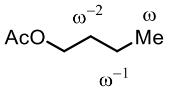	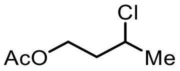 **34**	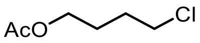 **34'**	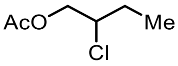 **34''**
**ω–1:ω:ω–2**	**ω–1 HAT Selectivity (%)**		
14:1.7:1.0	84		

Following **GP3** using **A1** as the amine and HFIP as the solvent, butyl acetate (26 μL, 0.2 mmol) gave **34**, **34’** and **34’’** in 19 % yield.

34: Data in accordance with the literature.[39]

34’: Data in accordance with the literature.[40]

34’’: Structural assignment done by analogy with similar compounds and distinctive $^{35/37}$Cl carbon peak shoulder.

^{1}H NMR (400 MHz, $CDCl_3$)

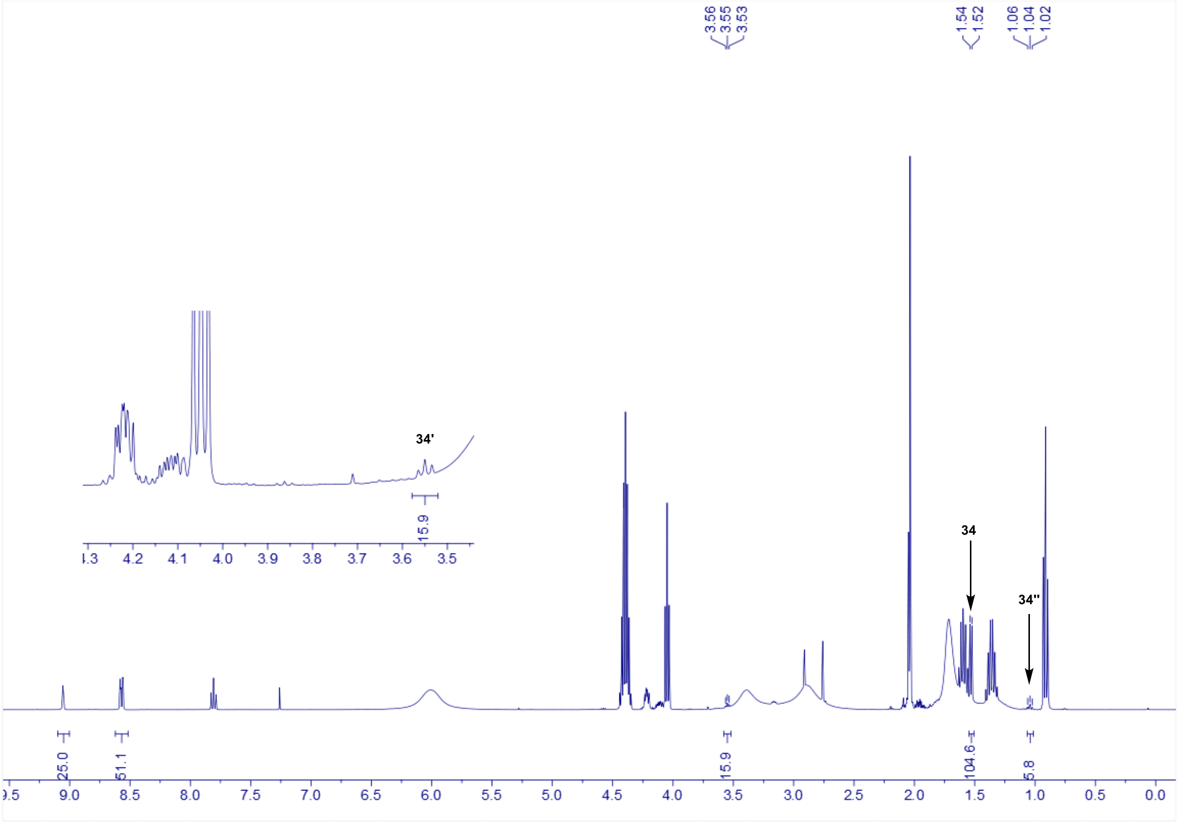

13C NMR (151 MHz, CDCl3)

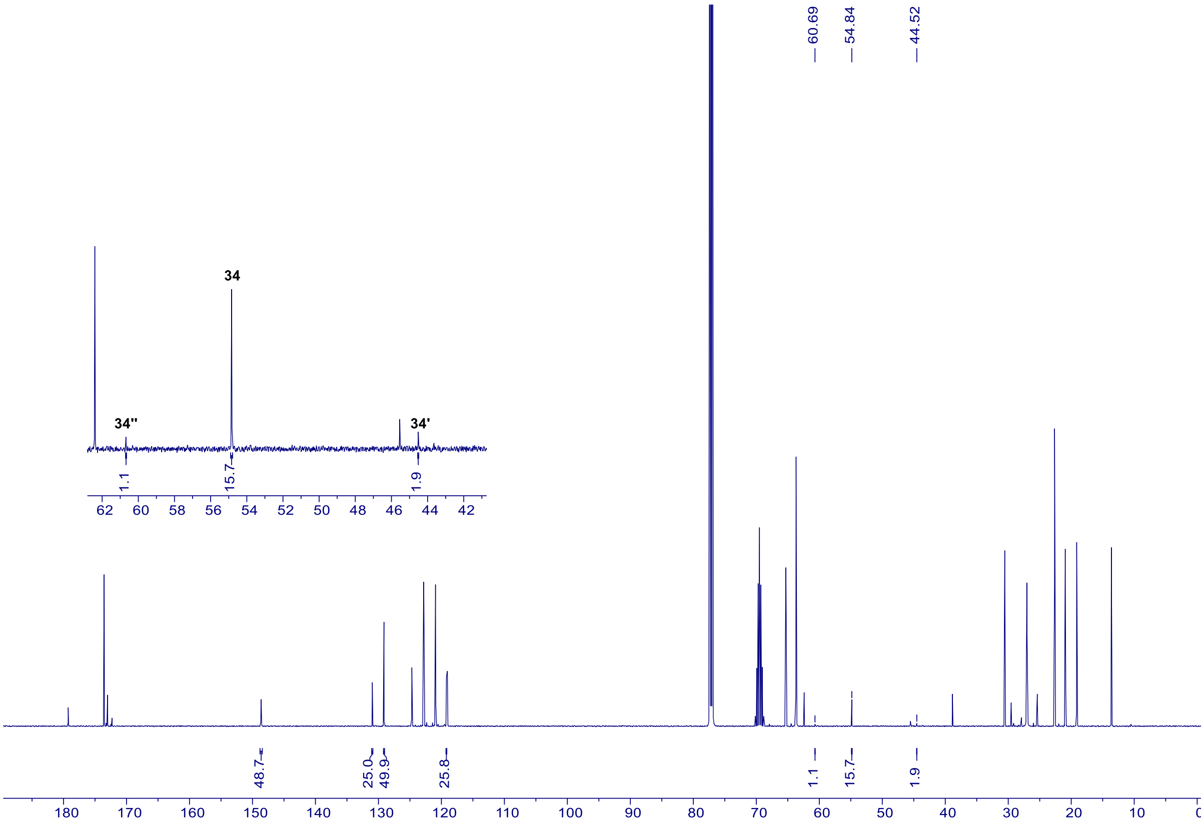

12.33 Chlorination of Methyl Pentanoate to Give 35

Methyl 4-Chloropentanoate (35)

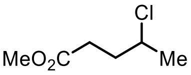

Starting Material	Observed Reaction Products
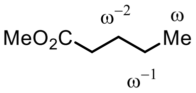	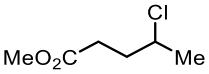 **35** 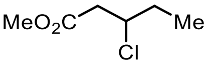 **35'** 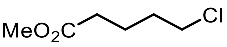 **35''**
**ω–1:ω–2:ω**	**ω–1 HAT Selectivity (%)**
8.1:1.7:1.0	75

Following **GP3** using **A3** as the amine and CH_2Cl_2 as the solvent, methyl pentanoate (29 μL, 0.2 mmol) gave **35**, **35’** and **35’’** in 35% yield. GC-MS m/z (EI): 150.1 (M+).

35’, **35’’**: Structural assignment done by analogy with similar compounds and distinctive $^{35/37}$Cl carbon peak shoulder

35: Data in accordance with the literature.[21]

^{1}H NMR (400 MHz, $CDCl_3$)

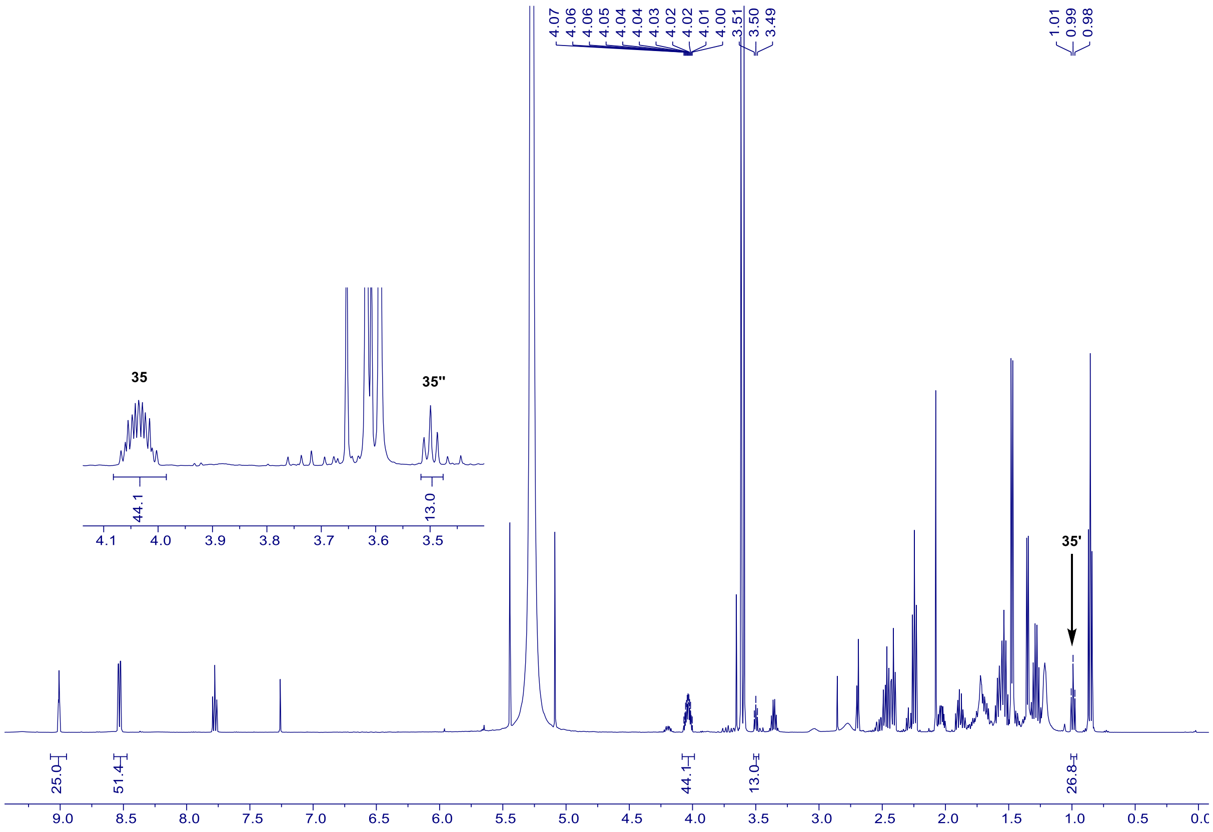

13C NMR (151 MHz, CDCl3)

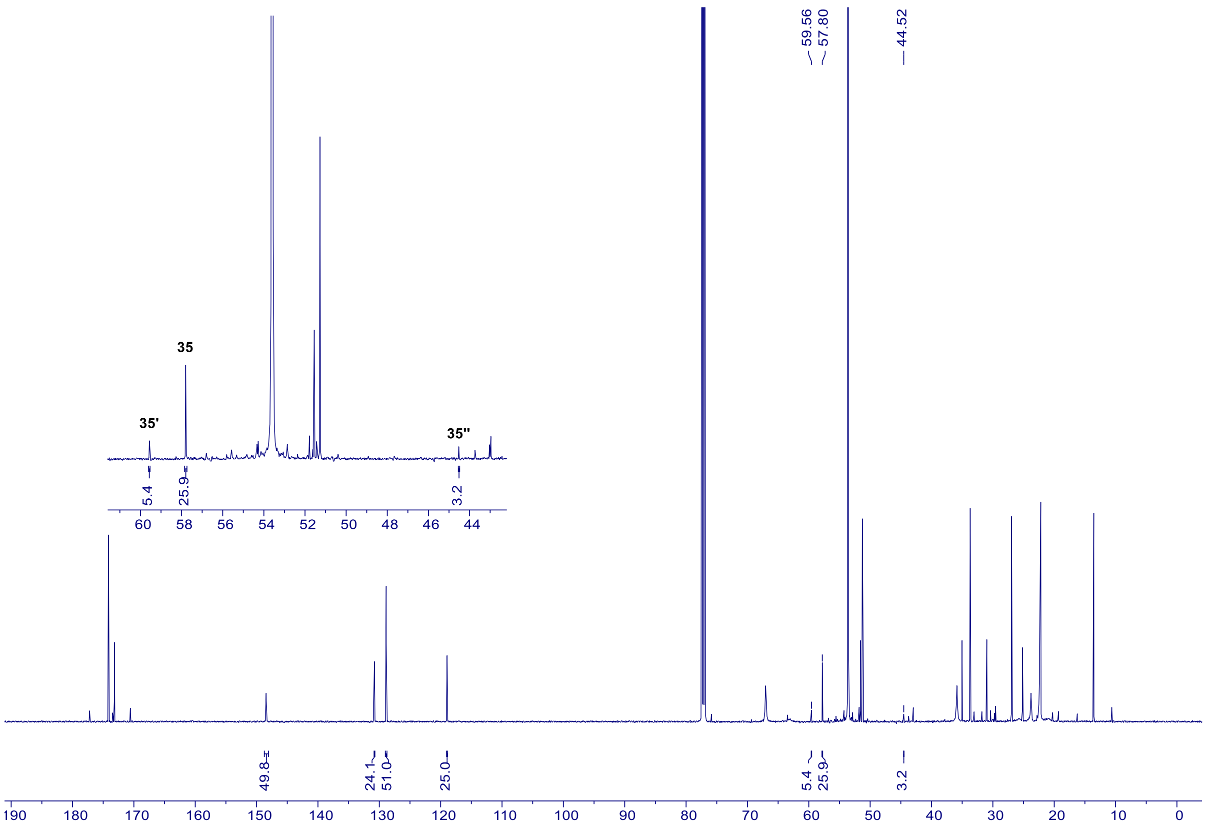

12.34 Chlorination of γ-Undelactone to Give 36

5-(6-Chloroheptyl)dihydrofuran-2(3H)-one (36)

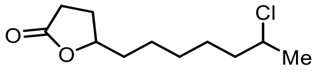

Starting Material	Observed Reaction Products
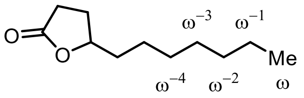	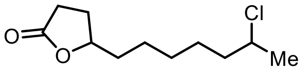 36 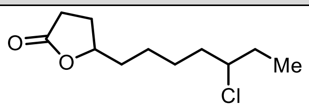 36' 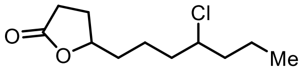 36'' 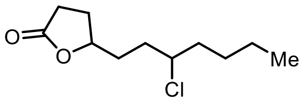 36''' 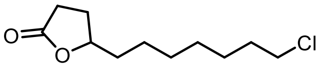 36''''
**ω–1:ω–2:ω–3:ω–4:ω**	**ω–1 HAT Selectivity (%)**
25:7.8:1.7:1.4:1.0	68

Following **GP3** using **A3** as the amine and HFIP as the solvent, γ-undeclactone (39 μL, 0.2 mmol) gave **36**, **36'**, **36''**, **36'''** and **36''''** in 62% yield. GC-MS m/z (EI): 218.1 (M+).

36, **36'**, **36''**: Structural assignment done based on J couplings and by analogy with similar compounds and distinctive $^{35/37}$Cl carbon peak shoulder.
36''', **36''''**: Structural assignment done by HSQC analysis.

^{1}H NMR (600 MHz, $CDCl_3$)

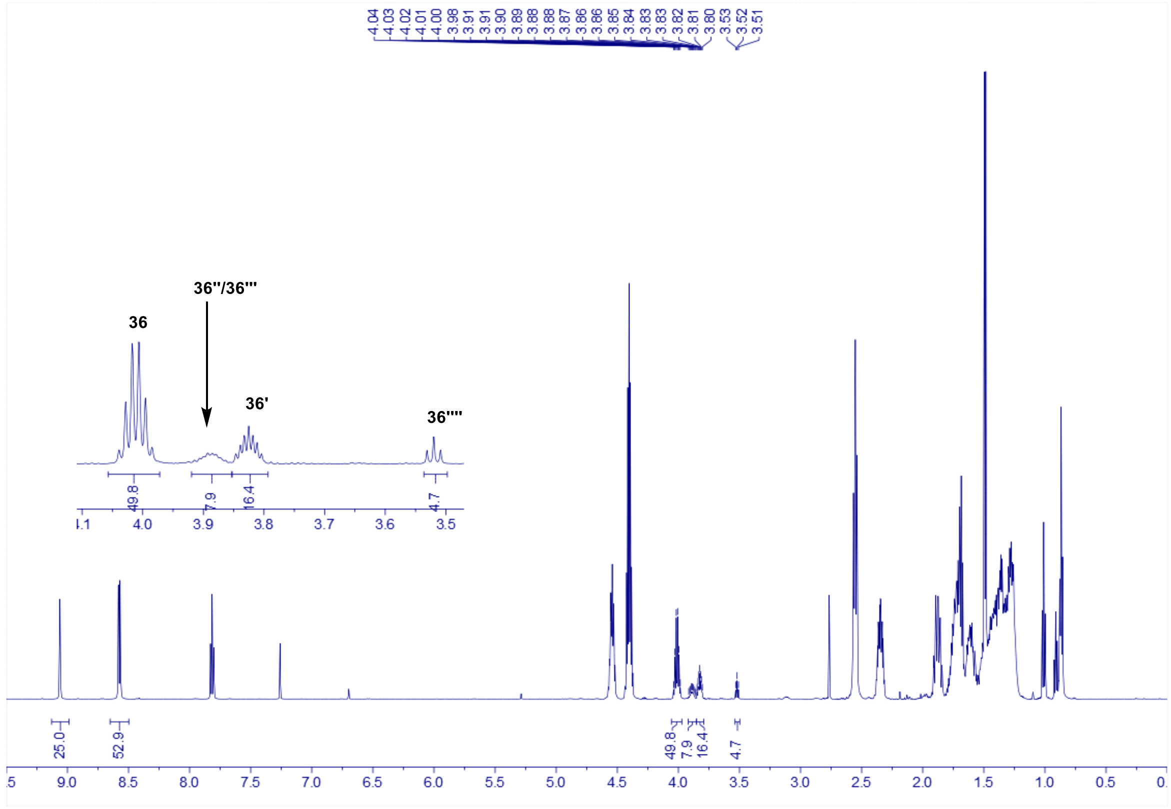

^{13}C Quantitative NMR (151 MHz, $CDCl_3$)

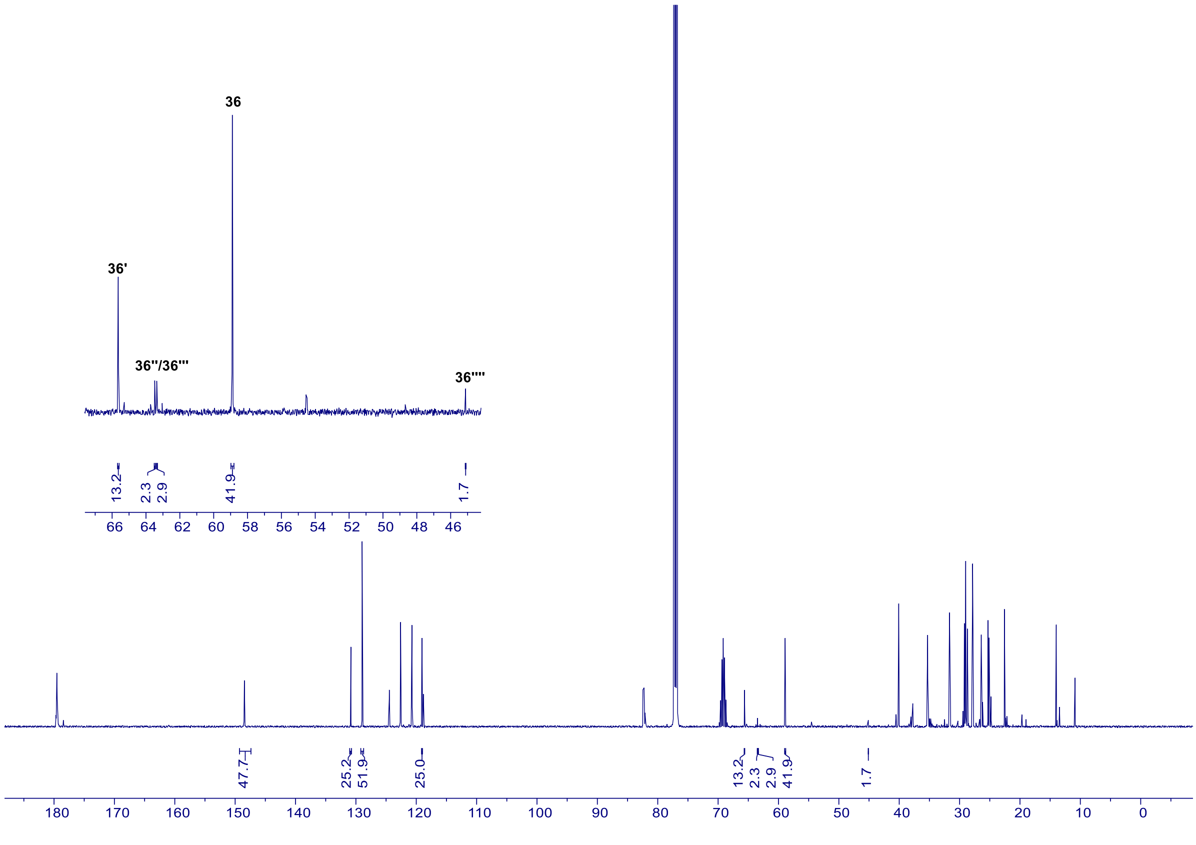

HSQC (400 MHz, $CDCl_3$)

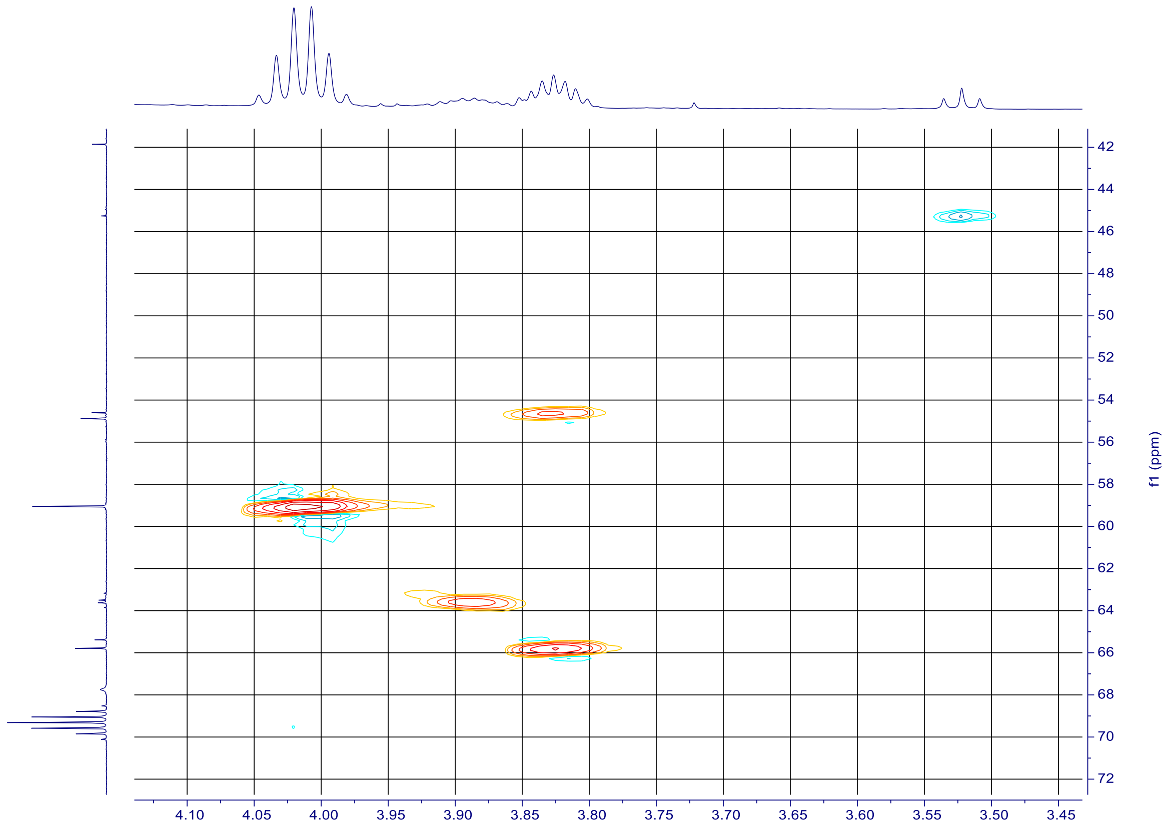

12.35 Chlorination of NCS60591 to give 37

10-Chloro-7-ethyl-2-methylundecan-4-ol (37)

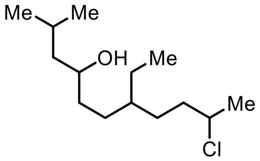

Starting Material	Observed Reaction Products
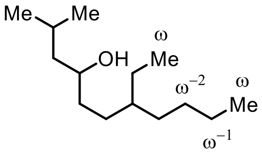	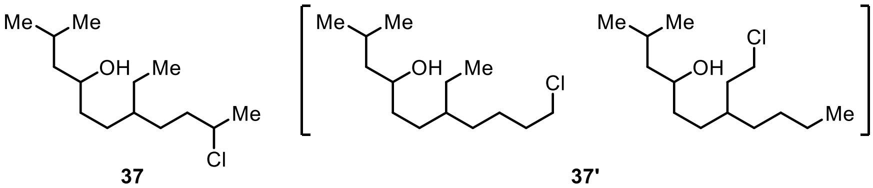
**ω–1:ω**	**ω–1 HAT Selectivity (%)**
12:1.0	92

This reaction was run utilising a modified procedure employing preformed TMPCl (**S7**) with HFIP as the solvent.

A tube equipped with a stirring bar was capped with a Supelco aluminium crimp seal with septum (PTFE/butyl), evacuated and refilled with N_2 (x 3). NCS60591 (51 μL, 0.2 mmol, 1.0 equiv.), HFIP (0.5 mL, 0.4M) (degassed by bubbling through with N_2 for 20 min) and TMPCl (36 μL, 0.2 mmol, 1.0 equiv) were added and the reaction mixture was cooled to 0 °C in an ice bath for 10 minutes. $HClO_4$ (52 μL, 0.6 mmol, 3.0 equiv., 70 % in H_2O) was charged immediately after which the tube was stirred under light irradiation for 2 h at 0 °C. Water (4 mL) was charged followed by 1,3-dinitrobenzene (8.4 mg, 0.25 equiv., 0.1 M solution in $CDCl_3$). The layers were separated and the organic phase was passed through a short pad of $MgSO_4$ (anhydrous) directly into an NMR tube for analysis to give **37** and **37'** 32% yield. GC-MS m/z (EI): 248.2 (M+).

37, **37'**: Structural assignment done using J constants, analogy with similar compounds and distinctive $^{35/37}$Cl carbon peak shoulder.

^{1}H NMR (600 MHz, $CDCl_3$)

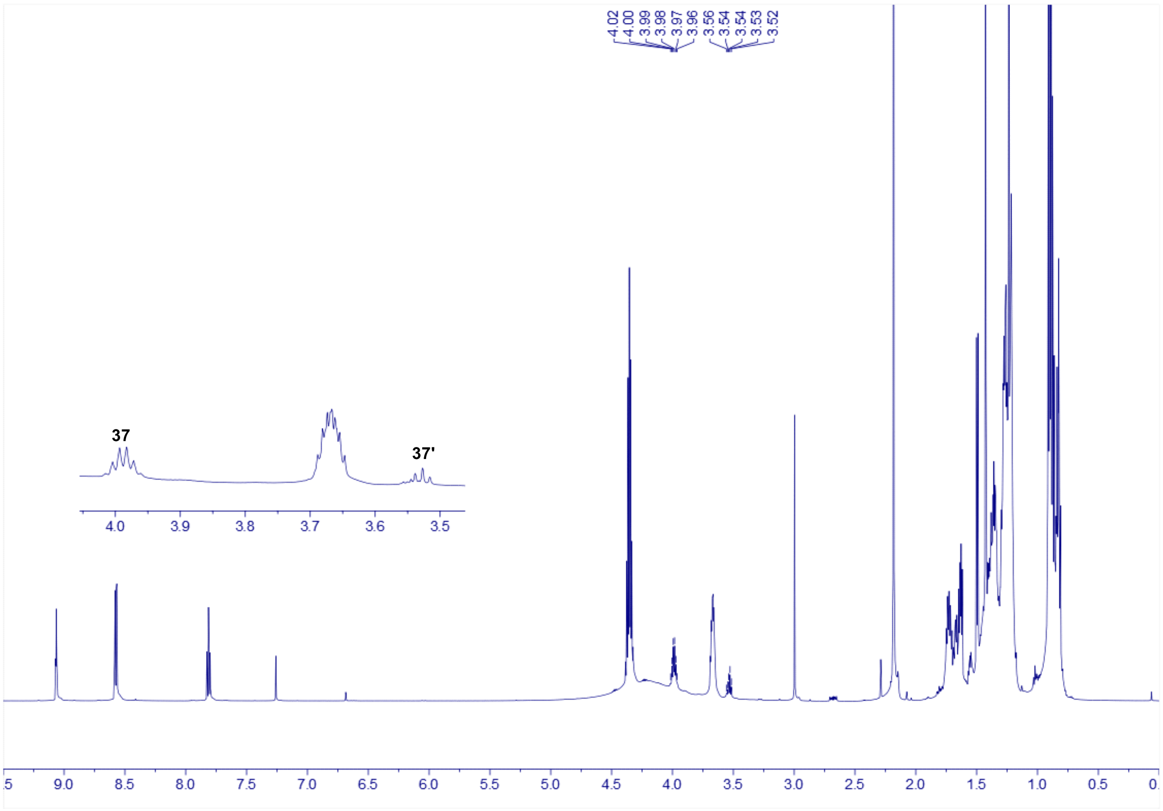

^{13}C Quantitative NMR (151 MHz, $CDCl_3$)

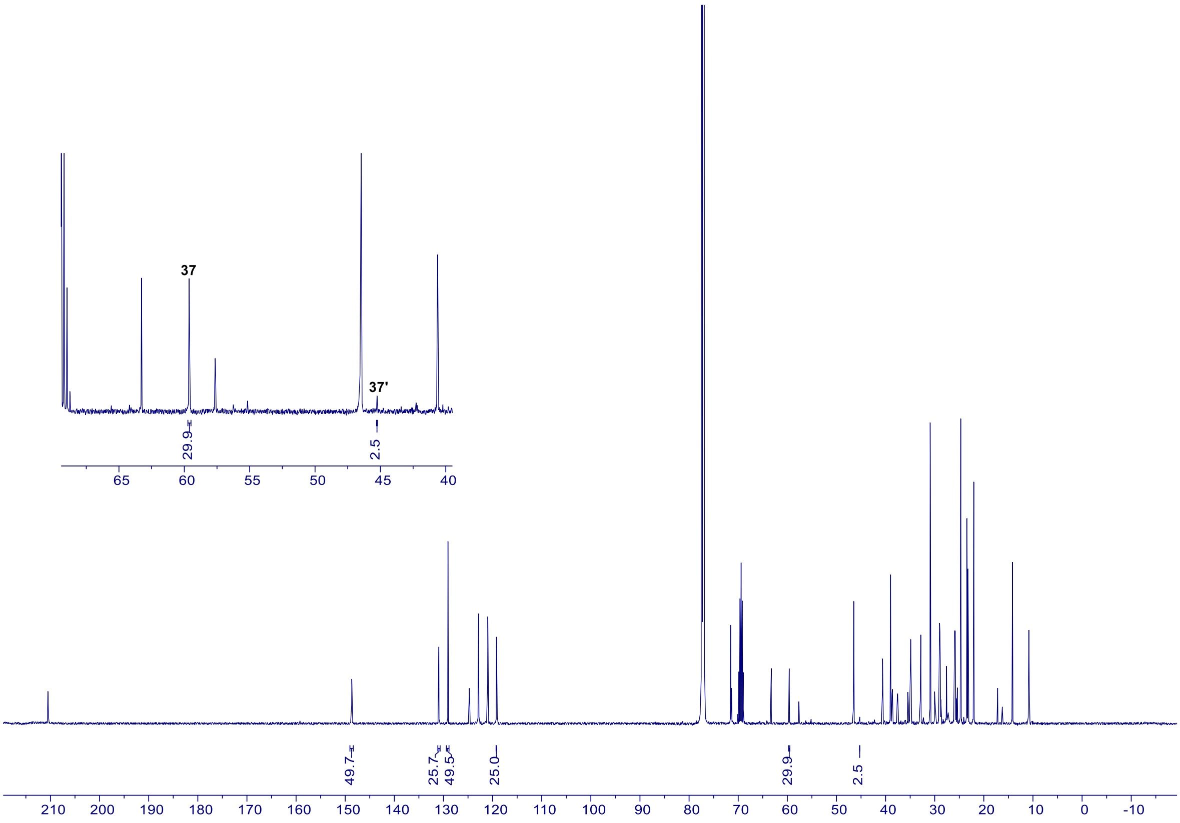

HSQC (400 MHz, $CDCl_3$)

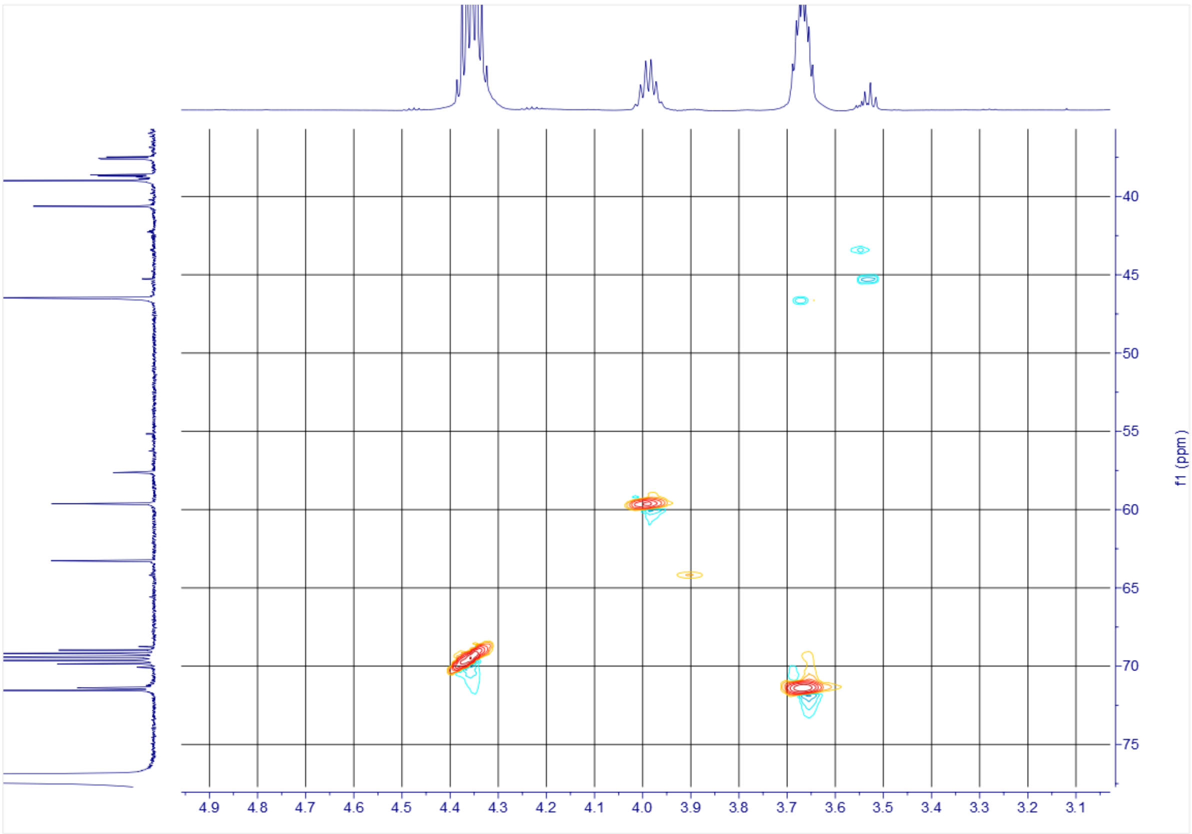

12.36 Chlorination of 5α-Cholestane to Give 38

(5*S*,8*R*,9*S*,10*S*,13*R*,14*S*,17*R*)-3-Chloro-10,13-dimethyl-17-((*R*)-6-methylheptan-2-yl)hexadecahydro-1H-cyclopenta[a]phenanthrene (38)

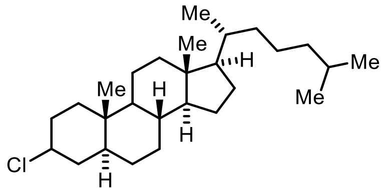

Starting Material	Observed Reaction Products
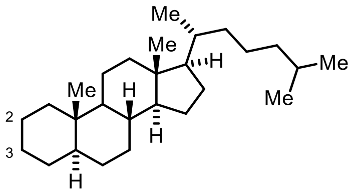	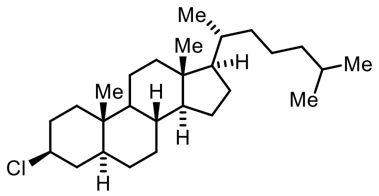 38a 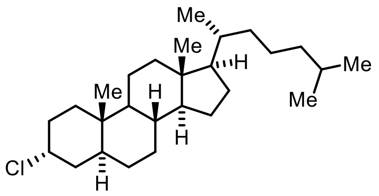 38b 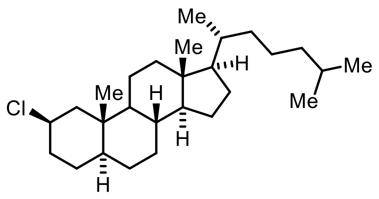 38'a 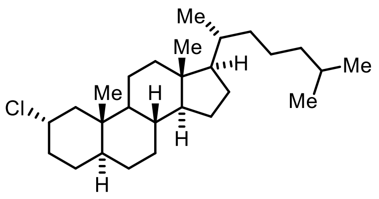 38'b
**C-3:C-2 (38a:38b:38’a:38’b)**	**C-3 HAT Selectivity (%)**
1.1:1.0 (1.4:1.5:1.0:1.6)	53

Following **GP4** using **A4** as amine and CH_2Cl_2 as the solvent, 5α-cholestane (74 mg, 0.2 mmol) gave **38** and **38’** in 58% yield. GC-MS m/z (EI): 406.3 (M+).

38a, **38b**, **38’a**, **38’b**: Data in accordance with the literature.[41]

^{1}H NMR (600 MHz, $CDCl_3$)

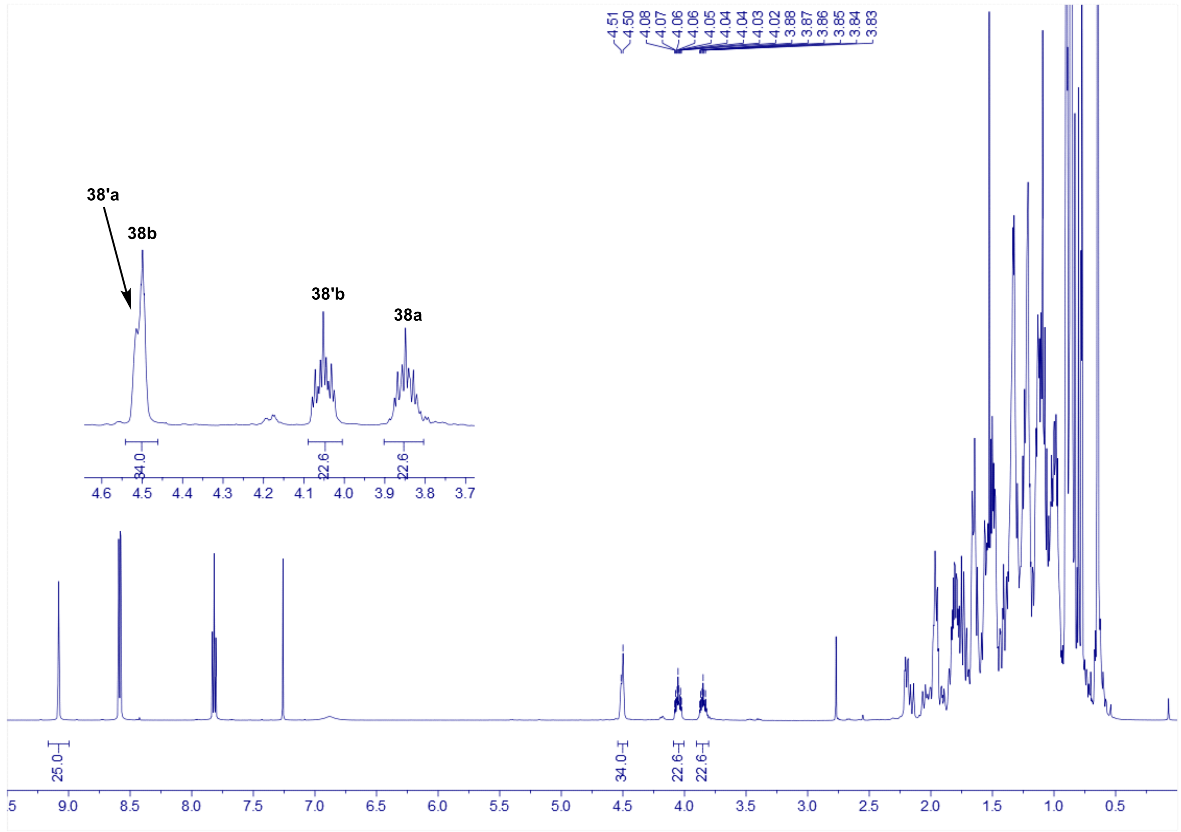

^{13}C NMR (151 MHz, $CDCl_3$)

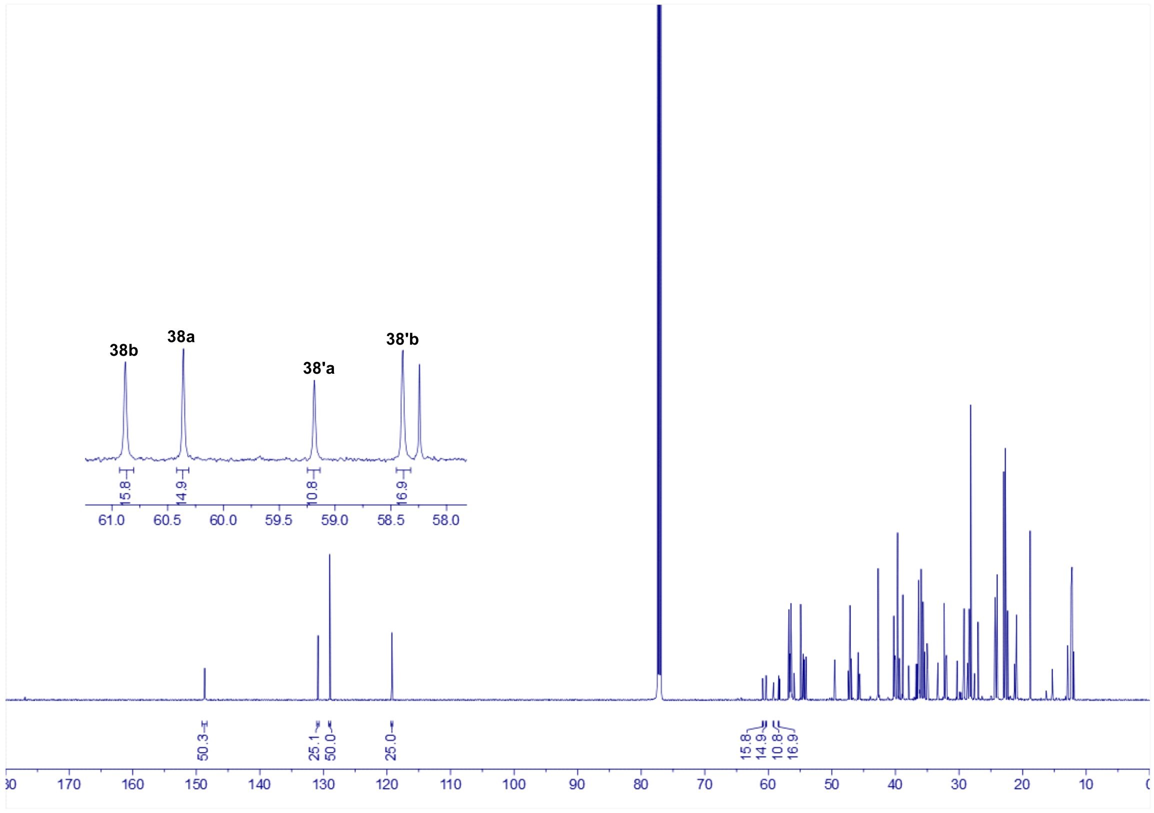

HSQC (400 MHz, $CDCl_3$)

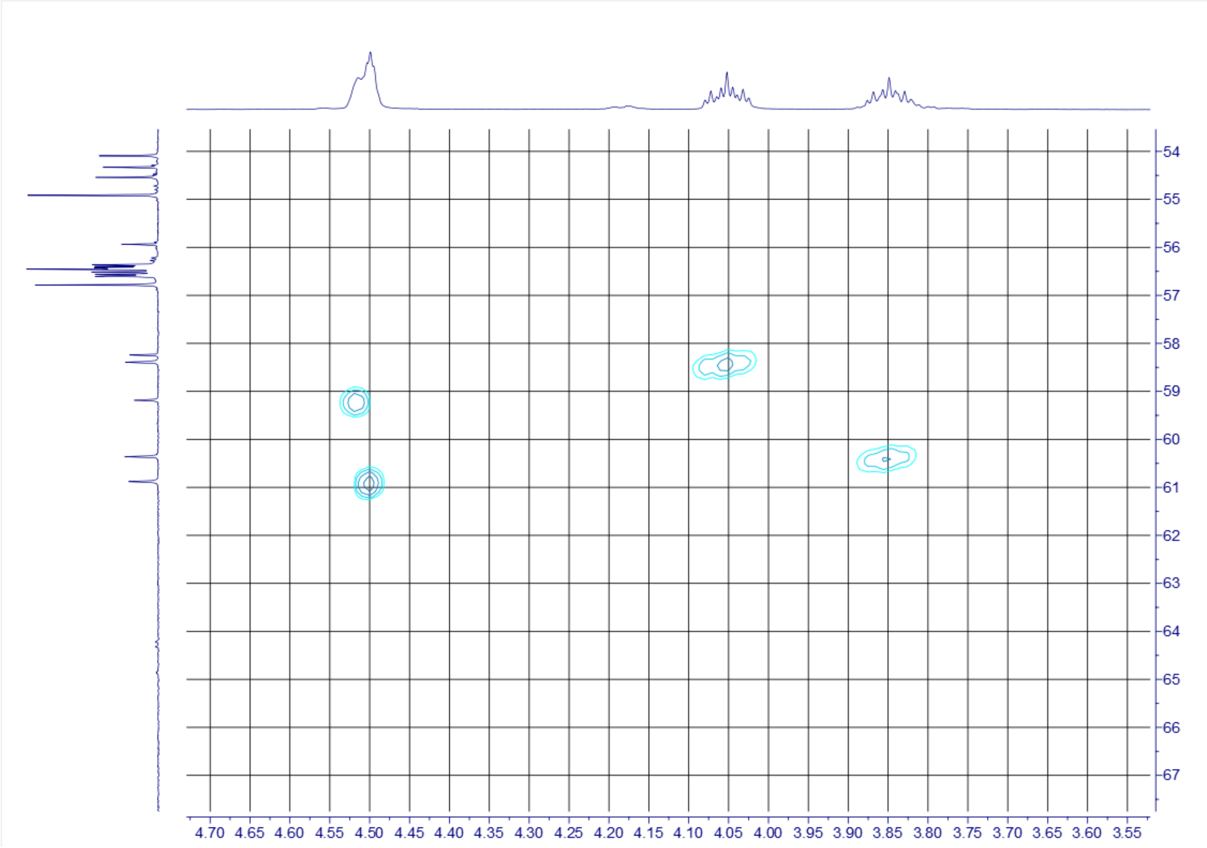

12.37 Chlorination of (+)-Sclareolide to Give 40

(3a*R*,5a*S*,9a*S*,9b*R*)-8-Chloro-3a,6,6,9a-tetramethyldecahydronaphtho[2,1-b]furan-2(1H)-one (40)

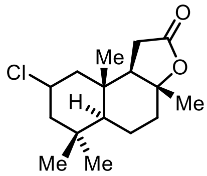

Starting Material	Observed Reaction Products	
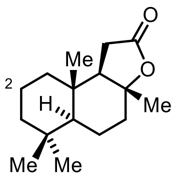	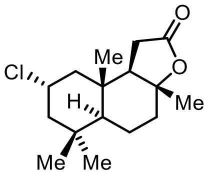 **40a**	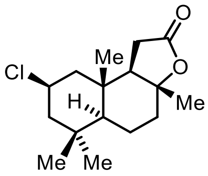 **40b**
**C-2 HAT Selectivity (%)**		
100		

Following a modified **GP3** using **A3** as the amine, CH_2Cl_2 as the solvent and adding Bu_4NCl (5.5 mg, 0.05 mmol, 0.1 equiv.) before the addition of (+)-sclareolide.

(+)-sclareolide (50 mg, 0.2 mmol) gave **40** in 60% yield.

^{1}H NMR (600 MHz, $CDCl_3$)

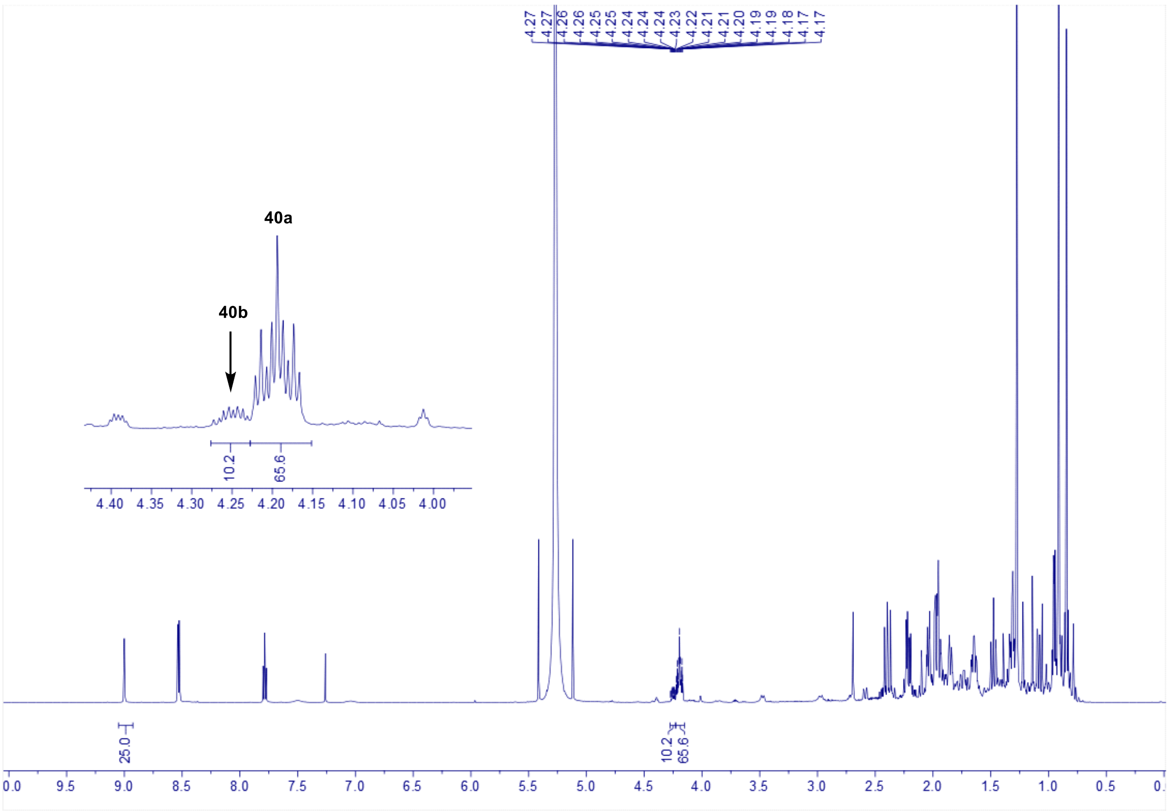

^{13}C NMR (151 MHz, $CDCl_3$)

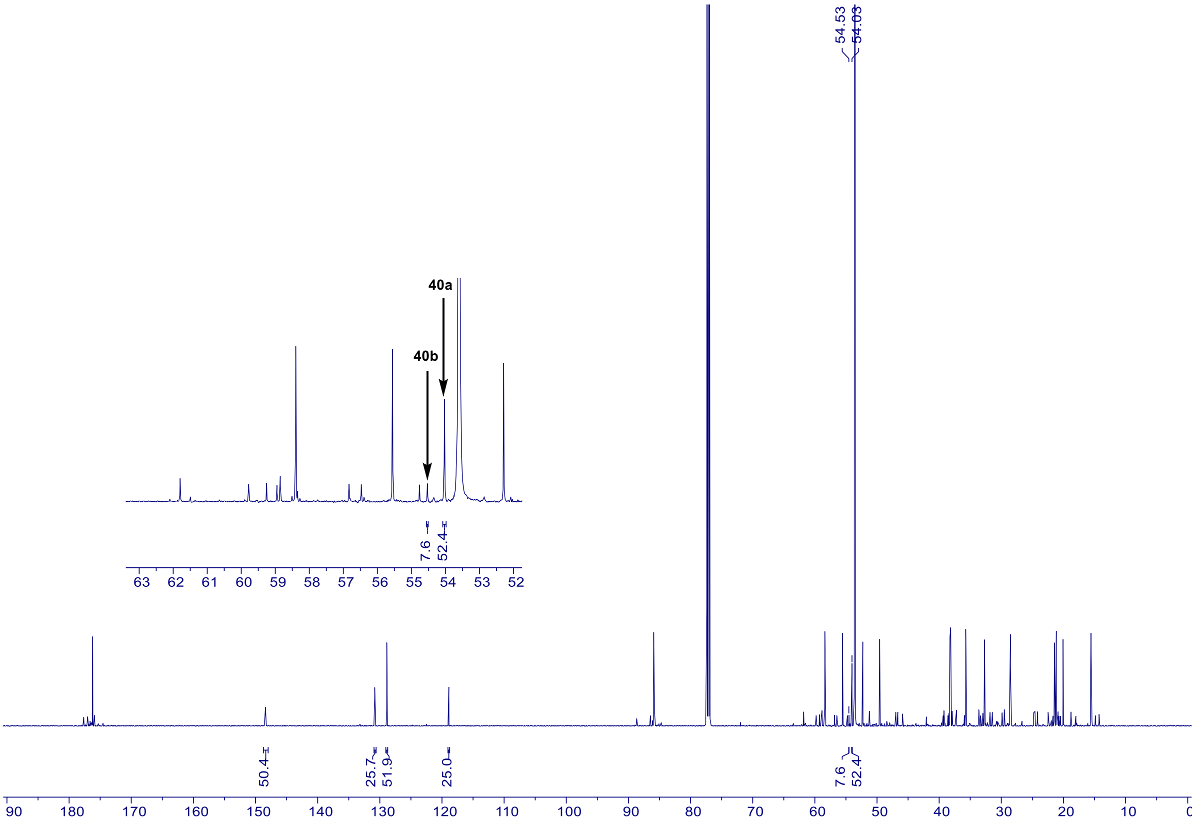

Purification performed by flash chromatography on silica gel eluting hexane-EtOAc (20:1 → 5:1) gave **40** as a mixture of two diastereoisomers. dr 6.9:1. ^{1}H NMR (400 MHz, $CDCl_3$) δ 4.28^{m} (0.1H, dtd, *J* = 10.1, 7.3, 4.2 Hz), 4.22^{M} (1H, tt, *J* = 12.2, 4.2 Hz), 2.46^{m} (0.1H, dd, *J* = 16.2, 14.8 Hz), 2.43^{M} (1H, dd, *J* = 16.2, 14.7 Hz), 2.27^{M} (1H, dd, *J* = 16.2, 6.5 Hz), 2.26^{m} (0.1H, dd, *J* = 16.2, 6.4 Hz), 2.09^{M} (1H, dt, *J* = 12.0, 3.4 Hz), 2.09–1.92^{m} (0.3H, m) 2.05–1.96^{M} (3H, m), 1.94–1.87^{m} (0.1H, m), 1.90^{M} (1H, dq, *J* = 14.1, 3.0 Hz), 1.83^{m} (0.1H, dq, *J* = 14.4, 3.4 Hz), 1.83–1.75^{m} (0.2H, m), 1.70^{M} (1H, td, *J* = 12.3, 4.1 Hz), 1.70–1.63^{m} (0.1H, m), 1.52^{M} (1H, t, *J* = 12.6 Hz), 1.50–1.43^{m} (0.1H, m), 1.41–1.33^{M} (2H, m), 1.33^{m} (0.3H, s), 1.32^{M} (3H, s), 1.19^{m} (0.3H, s), 1.15^{m} (0.1H, dd, *J* = 12.1, 3.2 Hz), 1.12^{M} (1H, dd, *J* = 12.6, 2.7 Hz), 1.01^{m} (0.3 H, s), 0.99^{m} (0.3H, s), 0.96^{M} (6H, s), 0.89 (3H, s); ^{13}C NMR (101 MHz, $CDCl_3$) δ 176.3^{m}, 176.2^{M}, 85.9^{M}, 85.8^{m}, 59.2^{m}, 58.7^{M}, 55.9^{M}, 54.5^{m}, 54.0^{M}, 52.5^{M}, 51.6^{m}, 49.8^{M}, 47.2^{m}, 46.9^{m}, 38.5^{M}, 38.2^{m}, 38.3^{M}, 37.4^{m}, 36.0^{M}, 33.9^{m}, 33.0^{M}, 31.8^{m}, 28.7^{m}, 28.7^{M}, 24.4^{m}, 21.8^{M}, 21.5^{M}, 21.3^{m}, 21.1^{m}, 20.3^{M}, 19.0^{m}, 15.9^{M}; GC-MS m/z (EI): 284.2 (M+). Data in accordance with the literature.[2,41]

12.38 Chlorination of (+)-Sclareolide to Give 41

(3a*S*,5a*S*,9a*S*,9b*R*)-4-Chloro-3a,6,6,9a-tetramethyldecahydronaphtho[2,1-b]furan-2(1H)-one (41)

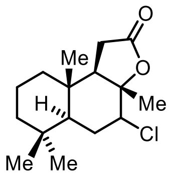

Starting Material	Observed Reaction Products
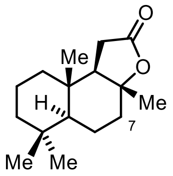	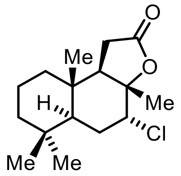 **41a** 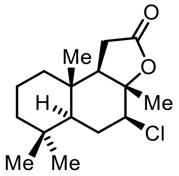 **41b**
**C-7 HAT Selectivity (%)**	
100	

This reaction was run utilising a modified procedure employing preformed TMPCl (**S7**) with CH_2Cl_2 as the solvent.

A tube equipped with a stirring bar was charged with (+)-sclareolide (50 mg, 0.2 mmol, 1.0 equiv.). The tube was capped with a Supelco aluminium crimp seal with septum (PTFE/butyl), evacuated and refilled with N_2 (x 3). CH_2Cl_2 (0.5 mL, 0.4M) (degassed by bubbling through with N_2 for 20 min) and TMPCl (72 μL, 0.4 mmol, 2.0 equiv.) were added followed by $HClO_4$ (105 μL, 1.2 mmol, 6.0 equiv., 70 % in H_2O) was charged immediately after which the tube was stirred under light irradiation for 2 h at r.t. (no fan was utilised so the actual temperature of the reaction will be higher). Water (4 mL) was charged followed by 1,3-dinitrobenzene (8.4 mg, 0.25 equiv., 0.1 M solution in $CDCl_3$). The layers were separated and the organic phase was passed through a short pad of $MgSO_4$ (anhydrous) directly into an NMR tube for analysis to give **41** in 29% yield.

^{1}H NMR (600 MHz, $CDCl_3$)

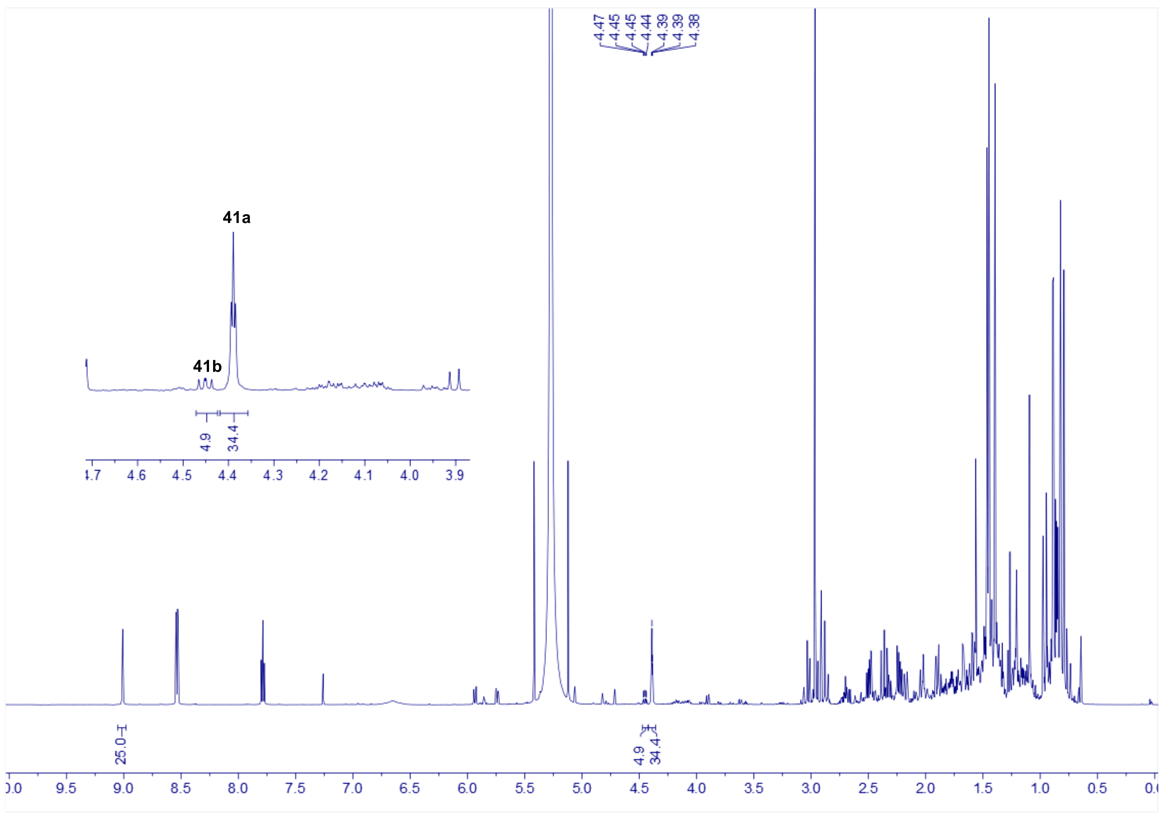

^{13}C NMR (151 MHz, $CDCl_3$)

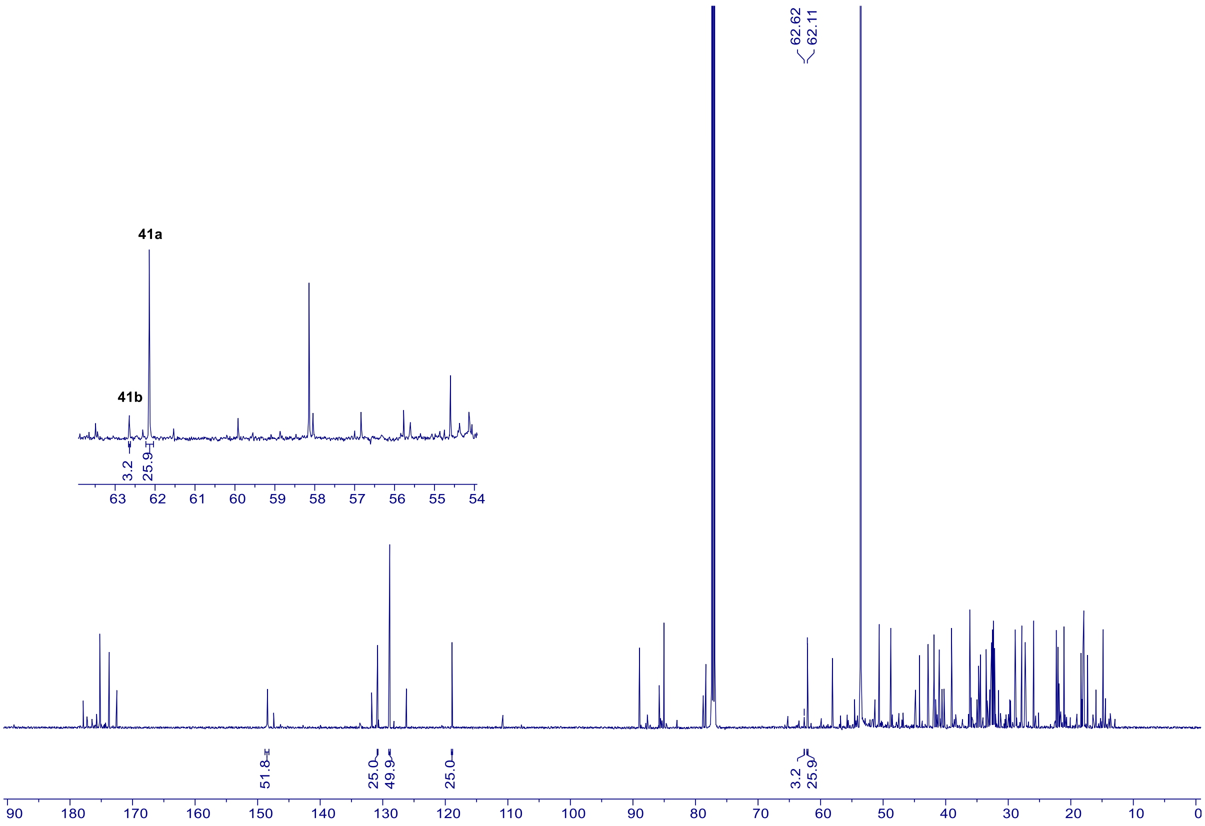

Purification by flash chromatography on silica gel eluting hexane-EtOAc (9:1 → 5:1) gave **41** as a mixture of diastereomers. dr 8.1:1. ^{1}H NMR (400 MHz, $CDCl_3$, 400 MHz) δ 4.42 (1H, t, *J* = 3.1 Hz), 2.56 (1H, dd, *J* = 14.2, 7.1 Hz), 2.40 (1H, dd, J = 16.1, 14.2 Hz), 2.29 (1H, dd, *J* = 16.1, 7.1 Hz), 2.09 (1H, dt, *J* = 15.3, 3.0 Hz), 1.92 (1H, ddd, *J* = 15.4, 12.7, 3.0 Hz), 1.73–1.62 (2H, m), 1.52–1.38 (3H, m), 1.44 (3H, s), 1.27 (1H, td, *J* = 13.6, 4.4 Hz), 1.14 (1H, td, *J* = 13.2, 4.0 Hz), 0.94 (3H, s), 0.87 (3H, s), 0.84 (3H, s); ^{13}C NMR (101 MHz, $CDCl_3$) δ 175.3, 85.2, 62.1, 50.8, 49.0, 42.1, 39.3, 36.4, 32.8, 32.7, 29.1, 28.1, 22.7, 21.5, 18.1, 15.2. HRMS (APCI POS): Found MH+ 285.1611, $C_{16}H_{26}ClO_2$ requires 285.1616.

Structural analysis confirmed by diagnostic HMBC couplings from α-chloro hydrogen.

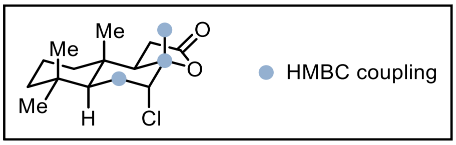

From the analysis of the crude ^{1}H-NMR of compound **41** is clear the presence of an olefin-containing by-product **41’** (**41**:**41’** = 4:1). We propose this product arises from the E2 elimination of **41**. Although we could not isolate **41’** as it co-eluted with another unknown impurity, we have been able to obtain a sample pure enough to enable structure determination by NMR spectroscopy, 2D-NMR analysis and HRMS.

Purification by two preparative TLC eluting with n-hexane-EtOAc (9:1) and then benzene-EtOAc (20:1). Diagnostic signals: ^{1}H NMR (400 MHz, $CDCl_3$, 400 MHz) δ 5.97 (1H, dd, *J* = 10.4, 1.7 Hz), 5.79 (1H, dd, *J* = 10.4, 3.1 Hz), 3.09 (1H, d, *J* = 18.2 Hz), 3.04 (1H, d, *J* = 18.2 Hz), 1.62 (3H, s), 1.04 (3H, s), 1.00 (3H, s), 0.91 (3H, s); ^{13}C NMR (101 MHz, $CDCl_3$) δ 172.76, 131.9, 126.5, 45.0, 40.9, 33.3, 27.5, 22.2, 16.3; HRMS (APCI POS): Found MH+ 248.1821, $C_{16}H_{24}O_2$ requires 248.1828

Structural analysis confirmed by diagnostic NOESY, COSY and HMBC couplings.

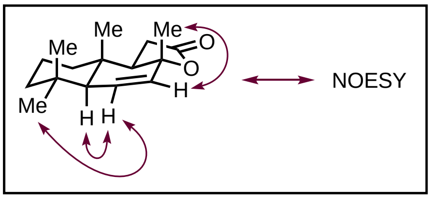

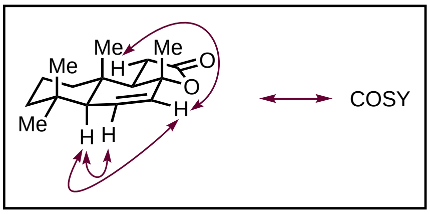

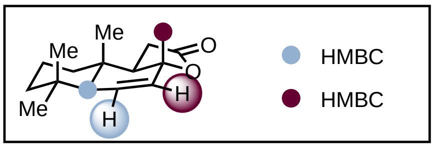
Me
Me
Me
O
O
H
H
HMBC
HMBC

13 Gram-scale Reactions

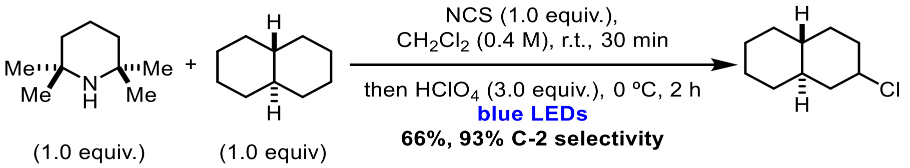

This reaction was performed on a 7.2 mmol scale (1.0 g) according to **GP4** to give **3** in 66% yield and 93% C-2 selectivity according to crude ^{1}H NMR spectroscopy. Due to the low boiling point of **3** an isolated yield was not obtained.

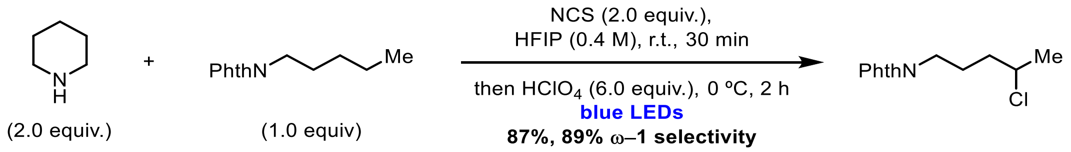

This reaction was performed on a 4.6 mmol scale (1.0 g) according to **GP3** making use of a modified workup in which, after blue LEDs irradiation, the reaction mixture was diluted with CH_2Cl_2 (20 mL) and washed with water (3 x 20 mL). The layers were separated and the organic layer was dried ($MgSO_4$), filtered and evaporated. Purification by flash column chromatography on silica gel eluting with *n*-hexane-EtOAc (4:1→2:1) gave **20**, **20'** and **20''** (1.01 g, 87%, 89% ω-1 selectivity) as an oil.

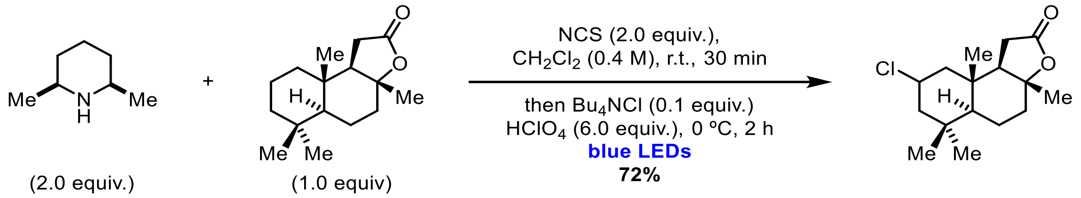

This reaction was performed on a 4 mmol scale (1.0 g) according to **GP3** making use of a modified workup in which, after blue LEDs irradiation, the reaction mixture washed with water (3 x 20 mL). The layers were separated and the organic layer was dried ($MgSO_4$), filtered and evaporated. Purification by flash column chromatography on silica gel eluting *n*-hexane-EtOAc (9:1→4:1) gave **40a** and **40b** (0.82 g, 72%) as a solid.

14 X-Ray Analysis of Compound 41

PLATON version of 05/12/2020; check.def file version of 05/12/2020

Datablock s5712a - ellipsoid plot

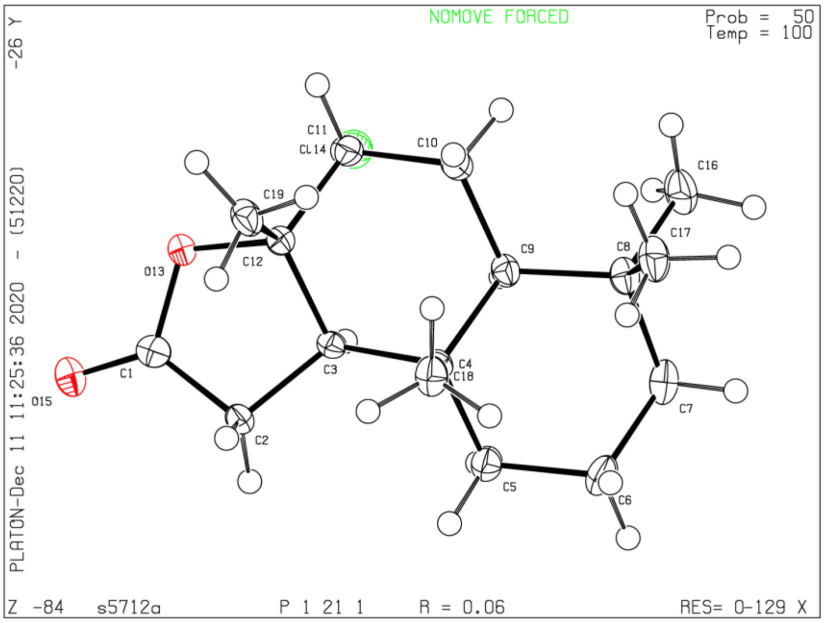

checkCIF/PLATON report

Structure factors have been supplied for datablock(s) s5712a

THIS REPORT IS FOR GUIDANCE ONLY. IF USED AS PART OF A REVIEW PROCEDURE FOR PUBLICATION, IT SHOULD NOT REPLACE THE EXPERTISE OF AN EXPERIENCED CRYSTALLOGRAPHIC REFEREE.

No syntax errors found. CIF dictionary Interpreting this report

Datablock: s5712a

```
Bond precision:   C-C = 0.0060 A          Wavelength=0.71073

Cell:             a=7.6920(6)     b=7.8889(5)        c=12.3729(8)
                  alpha=90        beta=91.021(6)     gamma=90
Temperature:      100 K

                 Calculated                  Reported
Volume           750.69(9)                   750.69(9)
Space group      P 21                        P 1 21 1
Hall group       P 2yb                       P 2yb
Moiety formula   C16 H25 Cl O2               C16 H25 Cl O2
Sum formula      C16 H25 Cl O2               C16 H25 Cl O2
Mr               284.81                      284.81
Dx,g cm-3        1.260                       1.260
Z                2                           2
Mu (mm-1)        0.251                       0.251
F000             308.0                       308.0
F000'            308.42
h,k,lmax         10,10,16                    10,10,16
Nref             4017[ 2148]                 3414
Tmin,Tmax        0.949,0.961                 0.468,1.000
Tmin'            0.949

Correction method= # Reported T Limits: Tmin=0.468 Tmax=1.000
AbsCorr = MULTI-SCAN

Data completeness= 1.59/0.85       Theta(max)= 29.085

R(reflections)= 0.0579( 2842)      wR2(reflections)= 0.1320( 3414)

S = 1.045                  Npar= 176
```

The following ALERTS were generated. Each ALERT has the format
test-name_ALERT_alert-type_alert-level.
Click on the hyperlinks for more details of the test.

15 Computational Studies

15.1 General Details

Density-Functional Theory calculations were carried out with the Gaussian 09 package at the UB3LYP/6-311+g(d,p) level.[42] All geometry optimizations were followed by vibrational frequency calculations to verify that the obtained geometries were true minima on the potential energy surface.

After gas-phase optimisation with density-functional theory molecular conformations were generated from the gas phase DFT-optimised structure using the genetic search algorithm in Open Babel, selecting to optimise for RMSD diversity.[43] This search algorithm considers only the rotation of single bonds with non-hydrogen substituents and so accounts for the rotation of aminium radical substituents in solution, but does not account for ring inversions, vibrations or rotation of small methyl substituents. For molecules with rotable substituents we report the average, standard deviation, minimum and maximum percentage visible solid angle (%VSA) over all conformers. The maximum %VSA is reported in the figures.

The AtomAccess code is basd on a ZCW angular grid centred on the atom of interest (N atom), where we have used density of 11, which corresponds to 4181 angular points.[44] Rays were traced out from the central atom in 0.02 Å increments until reaching the maximum distance between the atom of interest and any peripheral atom (atoms other than the central atom) plus the peripheral atoms's radius. At each radial step, the point was checked as to whether it lay within the van der Waals non-bonded radius[45] of any peripheral atom and if this was the case the ray was considered to be blocked. The %VSA was calculated as the fraction of unblocked rays over the total number of angular points.

15.2 %VSA Values

Table S5

Amine	Number of Conformations	%VSA (Optimised Structure)	Average %VSA (All Conformations)	Standard Deviation (All Conformations)	Min %VSA (All Conformations)	Max %VSA (All Conformations)
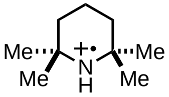	1	14.30	14.30	0.00	14.30	14.30
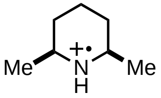	1	19.13	19.13	0.00	19.13	19.13
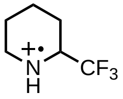	2	19.18	19.35	0.17	19.18	19.52
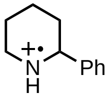	6	19.85	20.03	0.17	19.40	20.55
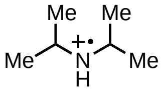	9	17.08	18.20	0.13	17.75	18.90
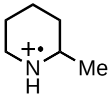	1	20.52	20.52	0.00	20.52	20.52
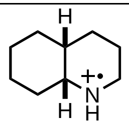	1	20.83	20.83	0.00	20.83	20.83

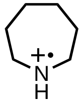	1	19.23	19.23	0.00	19.23	19.23
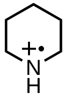	1	21.79	21.79	0.00	21.79	21.79
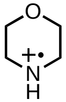	1	21.96	21.96	0.00	21.96	21.96
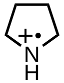	1	24.71	24.71	0.00	24.71	24.71
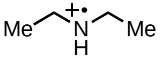	9	19.37	20.57	0.05	20.40	20.86
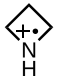	1	29.01	29.01	0.00	29.01	29.01

15.3 Correlation Between %VSA and Site-Selectivity for the Chlorination of *trans*-Decalin

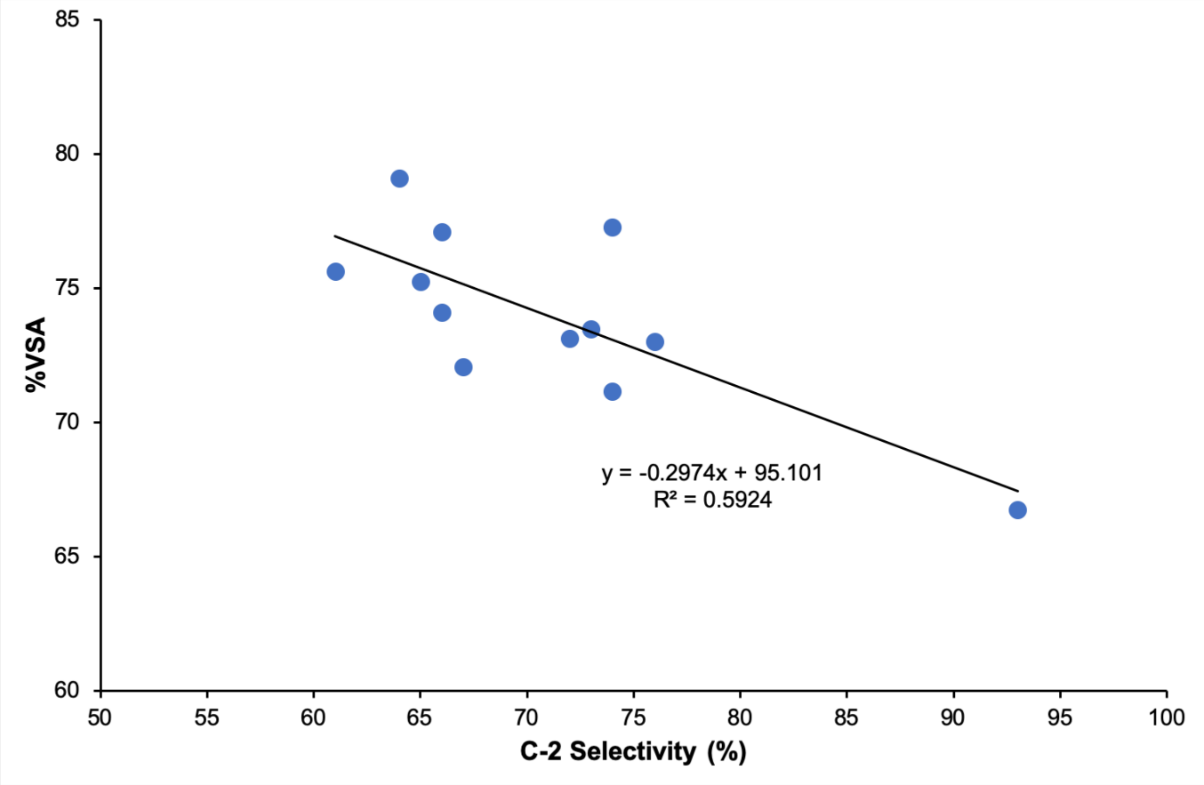

Figure S5.

15.4 Optimised geometries for aminium radicals

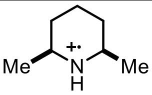

C	-0.74557	0.88028	1.26570
C	0.46984	-0.13760	1.25764
C	-0.74812	1.73200	0.00000
C	-0.74557	0.88028	-1.26570
C	0.46984	-0.13760	-1.25764
N	0.39001	-0.83352	0.00000
H	-0.62964	1.48323	2.16925
H	1.38080	0.47152	1.22976
H	-1.64498	2.36173	0.00000
H	-1.67174	0.30611	-1.36341
H	1.38080	0.47152	-1.22976
H	-1.67174	0.30611	1.36341
C	0.46984	-1.08944	2.44661
H	0.10909	2.41307	0.00000
H	-0.62964	1.48323	-2.16925
C	0.46984	-1.08944	-2.44661
H	-0.01485	-1.77088	0.00000
H	-0.44124	-1.69386	-2.47522
H	1.33680	-1.75272	-2.42304
H	0.51511	-0.51435	-3.37228
H	-0.44124	-1.69386	2.47522
H	0.51511	-0.51435	3.37228
H	1.33680	-1.75272	2.42304

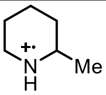

C	-1.89543	-0.05022	-0.29706
C	-1.09887	-1.30645	0.22257

C	-1.22256	1.22785	0.19771
C	0.25509	1.28284	-0.17877
C	1.02859	-0.00569	0.34452
N	0.27803	-1.13082	-0.14103
H	-2.91784	-0.15365	0.07276
H	-1.17428	-1.33886	1.31343
H	-1.73059	2.09051	-0.24824
H	0.39135	1.34343	-1.26245
H	0.92458	0.00255	1.43616
H	-1.93059	-0.08517	-1.38924
H	-1.48180	-2.23054	-0.20779
H	-1.34136	1.32307	1.28192
H	0.76509	2.13743	0.27173
C	2.48874	-0.04937	-0.08282
H	0.63101	-1.62061	-0.96311
H	2.58719	-0.05177	-1.17207
H	2.99323	-0.92873	0.32267
H	3.00447	0.83441	0.29463

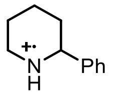

C	1.28124	0.80488	-1.01846
C	0.55578	0.39941	0.35451
C	2.79229	0.91000	-0.83986
C	3.38956	-0.37389	-0.27155
C	2.68096	-0.73723	1.06829
N	1.25066	-0.73818	0.88460
H	0.83045	1.74985	-1.32370
H	3.23748	1.11489	-1.81997
H	3.27244	-1.20568	-0.97201
H	2.92816	0.03311	1.80977
H	1.01844	0.04992	-1.76271

H	3.04336	1.76232	-0.19945
H	4.45530	-0.26860	-0.05892
H	2.99125	-1.70913	1.44878
H	0.73275	-1.60811	0.98627
C	-0.91360	0.16667	0.16018
C	-1.37588	-0.95591	-0.54499
H	-0.67627	-1.65603	-0.99323
C	-2.74058	-1.16878	-0.70621
H	-3.09166	-2.03657	-1.25115
C	-3.65403	-0.25176	-0.18464
H	-4.71700	-0.41039	-0.32139
C	-3.20020	0.87734	0.49983
H	-3.90975	1.58918	0.90379
C	-1.83601	1.08840	0.66967
H	-1.48950	1.96312	1.20983
H	0.74262	1.24448	1.03130

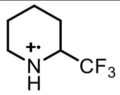

C	-2.75681	0.08398	0.29916
C	-1.94061	1.32749	-0.24935
C	-2.13216	-1.21070	-0.21258
C	-0.64676	-1.31548	0.12842
N	-0.56618	1.11341	0.08168
H	-3.78351	0.22055	-0.04710
H	-2.04731	1.35231	-1.33801
H	-2.65229	-2.05777	0.24929
H	-2.75957	0.12708	1.39110
H	-2.28834	2.26002	0.19153
H	-2.28236	-1.30604	-1.29274
C	1.59825	-0.00139	-0.02398
F	2.21789	1.01371	-0.64084
F	2.20123	-1.14218	-0.34470

C	0.11513	-0.04570	-0.42422
H	-0.17508	-2.18434	-0.33440
H	-0.48417	-1.37376	1.20760
H	-0.17564	1.57598	0.90509
F	1.70794	0.19659	1.30864
H	0.04575	-0.05026	-1.51676

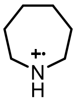

N	0.27553	-1.40070	-0.35474
C	-0.93241	-1.27029	0.40453
C	-1.76916	-0.00951	-0.04606
C	-0.97144	1.26983	-0.35768
C	0.34468	1.48880	0.41986
C	1.54088	0.72007	-0.14541
C	1.51899	-0.83820	0.08421
H	0.20636	-1.73787	-1.31481
H	-0.65762	-1.18297	1.45504
H	-1.54528	-2.16303	0.25640
H	-2.45595	0.15114	0.78941
H	-2.37191	-0.29430	-0.91015
H	-1.63727	2.11484	-0.16978
H	-0.74417	1.30626	-1.42855
H	0.21851	1.28461	1.48928
H	0.59724	2.55056	0.35155
H	2.47098	1.03717	0.33555
H	1.64368	0.91214	-1.21680
H	1.61873	-1.03583	1.15422
H	2.33872	-1.30197	-0.46495

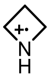

N	0.00000	-1.00818	0.00001

C	1.07087	-0.03182	-0.00000
C	0.00000	1.09285	0.00001
H	1.71592	-0.13072	0.88484
H	1.71589	-0.13072	-0.88486
C	-1.07087	-0.03182	-0.00000
H	0.00000	1.71813	0.89013
H	0.00000	1.71814	-0.89011
H	-1.71592	-0.13072	0.88484
H	-1.71589	-0.13072	-0.88486
H	0.00000	-2.03142	0.00001

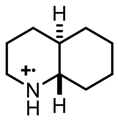

C	-2.46979	0.73200	-0.40229
C	-2.52640	-0.70029	0.14320
C	-1.23477	-1.48776	-0.12252
C	-0.02242	-0.65542	0.44170
C	0.05400	0.73938	-0.18113
C	-1.24495	1.49784	0.12259
C	1.33960	1.44584	0.29703
C	2.61182	0.63308	-0.03789
C	2.29205	-0.82530	-0.50353
N	1.21263	-1.36486	0.27475
H	-2.44064	0.70618	-1.49787
H	-2.72188	-0.68318	1.22124
H	-1.26510	-2.46880	0.35844
H	-1.19717	2.49008	-0.33348
H	1.27058	1.60349	1.37765
H	3.17942	1.09122	-0.84957
H	3.15412	-1.48721	-0.44384
H	1.39542	2.43536	-0.15950
H	3.27356	0.56939	0.82738
H	1.95807	-0.78783	-1.55120

H	-3.38420	1.26469	-0.13106
H	-3.35338	-1.24981	-0.31651
H	-1.07504	-1.63828	-1.19441
H	-0.19547	-0.59179	1.52975
H	0.11517	0.61757	-1.27181
H	-1.33166	1.65654	1.20470
H	1.33499	-2.24977	0.76384

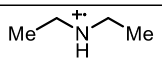

C	2.41718	-0.25980	-0.00811
C	1.14051	0.59363	0.22497
N	-0.00000	0.00003	-0.42527
C	-1.14049	-0.59363	0.22495
C	-2.41720	0.25977	-0.00809
H	3.24448	0.25525	0.48319
H	2.64900	-0.35078	-1.07044
H	2.30595	-1.25204	0.42943
H	1.29524	1.58564	-0.21806
H	0.91896	0.69628	1.28658
H	-0.91894	-0.69632	1.28655
H	-1.29519	-1.58563	-0.21813
H	-2.30599	1.25199	0.42949
H	-3.24447	-0.25533	0.48318
H	-2.64902	0.35079	-1.07042
H	-0.00001	0.00007	-1.44685

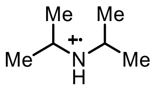

C	2.02738	1.18230	-0.60936
C	1.19972	-0.06415	-0.26123
N	-0.00443	0.32850	0.44219
C	-1.35789	-0.19758	0.33479
C	-2.34312	0.99508	0.26830

H	2.34425	1.71703	0.29045
H	1.47068	1.86421	-1.25386
H	2.92534	0.86547	-1.14252
C	1.99051	-1.04943	0.65148
H	0.89273	-0.59179	-1.16369
H	-1.52844	-0.69055	1.30982
C	-1.54233	-1.21014	-0.78717
H	-2.20378	1.68737	1.10189
H	-3.35908	0.60250	0.32485
H	-2.22769	1.53662	-0.67216
H	0.12491	1.03722	1.16725
H	2.30570	-0.56194	1.57611
H	2.88063	-1.35807	0.09937
H	1.40188	-1.93544	0.89101
H	-1.40654	-0.75212	-1.77010
H	-2.56361	-1.59059	-0.74121
H	-0.87160	-2.06586	-0.69332

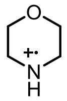

C	-0.00826	-0.81429	1.17361
C	-0.00826	0.78935	1.22079
O	-0.65628	-1.19896	0.00000
C	-0.00826	-0.81429	-1.17361
C	-0.00826	0.78935	-1.22079
N	0.57064	1.23525	0.00000
H	-0.58411	-1.18316	2.02122
H	-1.05242	1.10043	1.28694
H	1.02522	-1.17192	-1.21341
H	-1.05242	1.10043	-1.28694
H	1.02522	-1.17192	1.21341
H	0.55902	1.13892	2.08213
H	-0.58411	-1.18316	-2.02122

H	0.55902	1.13892	-2.08213
H	1.55848	1.47565	0.00000

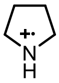

C	-0.74307	1.02044	-0.20757
C	0.74315	1.02038	0.20758
C	1.20653	-0.40970	-0.11239
C	-1.20656	-0.40962	0.11238
N	-0.00004	-1.19486	0.00000
H	-0.85247	1.21091	-1.27779
H	-1.33144	1.76339	0.32799
H	0.85256	1.21084	1.27780
H	1.33156	1.76330	-0.32799
H	1.99324	-0.83384	0.52174
H	1.56367	-0.51523	-1.15400
H	-1.99330	-0.83370	-0.52175
H	-1.56372	-0.51513	1.15399
H	-0.00007	-2.21554	0.00001

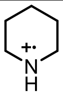

C	-1.26727	0.72339	-0.23717
C	-1.23947	-0.78483	0.23478
C	0.00002	1.43943	0.22416
C	1.26729	0.72335	-0.23717
C	1.23944	-0.78487	0.23478
N	-0.00002	-1.35252	-0.20606
H	-2.17271	1.16083	0.18902
H	-1.26601	-0.80201	1.32875
H	0.00003	2.45500	-0.18790
H	1.36555	0.74473	-1.32569
H	1.26598	-0.80205	1.32875

H	-1.36552	0.74476	-1.32569
H	-2.07644	-1.34897	-0.17296
H	0.00002	1.54521	1.31394
H	2.17274	1.16077	0.18902
H	2.07641	-1.34902	-0.17297
H	-0.00002	-1.94057	-1.03815

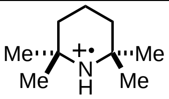			
C	-1.23720	0.49460	-1.25307
C	0.23319	0.03852	-1.32647
C	-1.96163	-0.00160	0.00000
C	-1.23720	0.49460	1.25307
C	0.23319	0.03852	1.32647
N	0.84795	0.07649	0.00000
H	-1.73868	0.15277	-2.16074
C	0.35250	-1.44118	-1.82070
H	-2.98554	0.37762	0.00000
H	-1.26385	1.58892	1.27351
C	0.35250	-1.44118	1.82070
H	-1.26385	1.58892	-1.27351
C	1.07637	0.94477	-2.24824
H	-2.04468	-1.09306	0.00000
H	-1.73868	0.15277	2.16074
C	1.07637	0.94477	2.24824
H	1.86781	0.01135	0.00000
H	1.07811	1.97728	1.89435
H	2.10815	0.59207	2.33143
H	0.63705	0.92757	3.24742
H	-0.27637	-2.11214	-1.23674
H	1.38416	-1.79701	-1.78536
H	0.02059	-1.46056	-2.86073
H	-0.27637	-2.11214	1.23674

H	0.02059	-1.46056	2.86073
H	1.38416	-1.79701	1.78536
H	1.07811	1.97728	-1.89435
H	0.63705	0.92757	-3.24742
H	2.10815	0.59207	-2.33143

15.5 Determination of Electrophilicity Indices

The electrophilicity indices for the aminium radical were calculated following a literature method.[46]

Table S6.

Entry	Aminium Radical	Local Electrophilicity Index (ω^{+}_{rc}, eV)
**1**	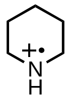	4.1
**2**	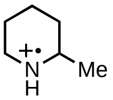	4.1
**3**	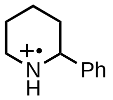	3.9
**4**	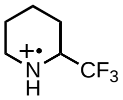	4.5
**5**	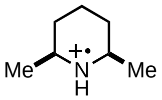	4.0
**6**	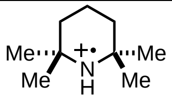	4.2
**7**	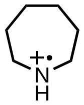	4.2
**8**	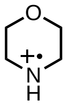	3.2
**9**	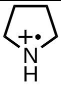	4.5
**10**	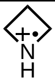	4.7
**11**	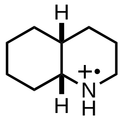	4.0
**12**	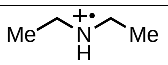	4.5
**13**	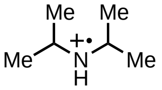	4.3

16 NMR spectra

40 – ^{1}H NMR (400 MHz, $CDCl_3$)

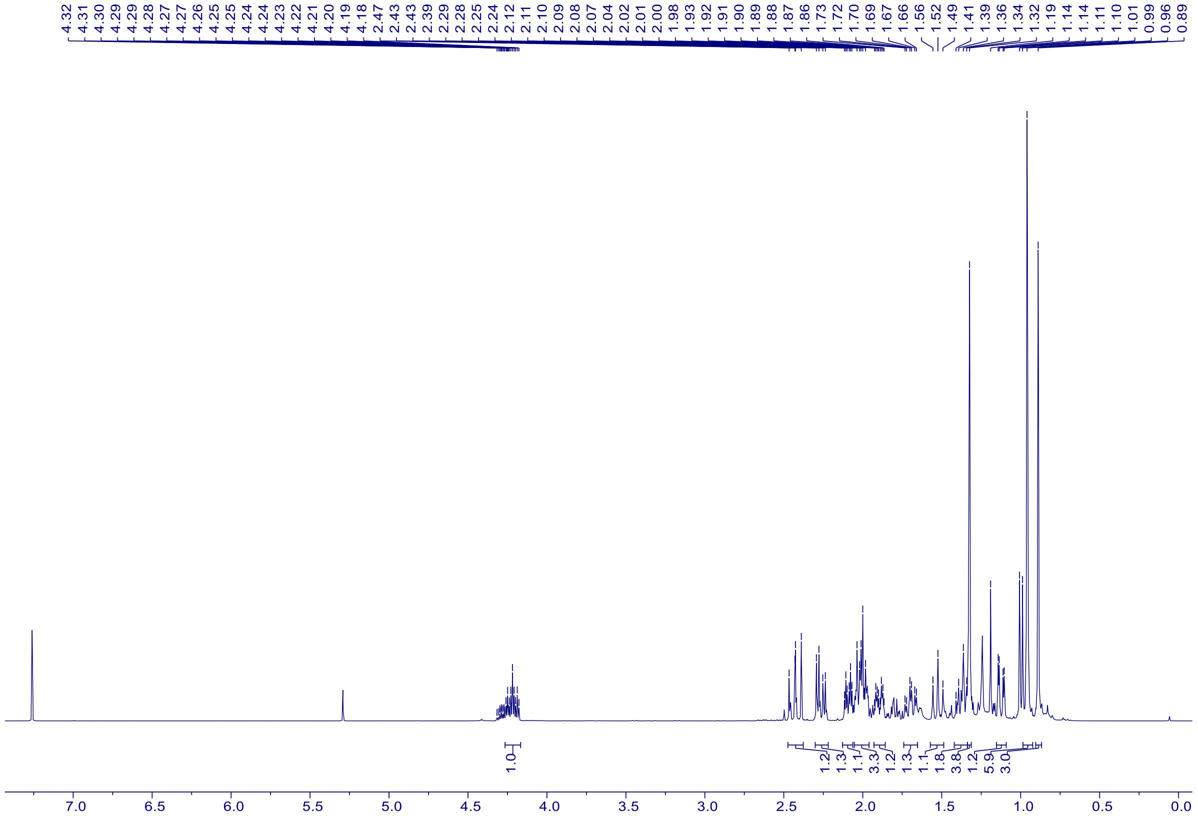

40 – ^{13}C NMR (101 MHz, $CDCl_3$)

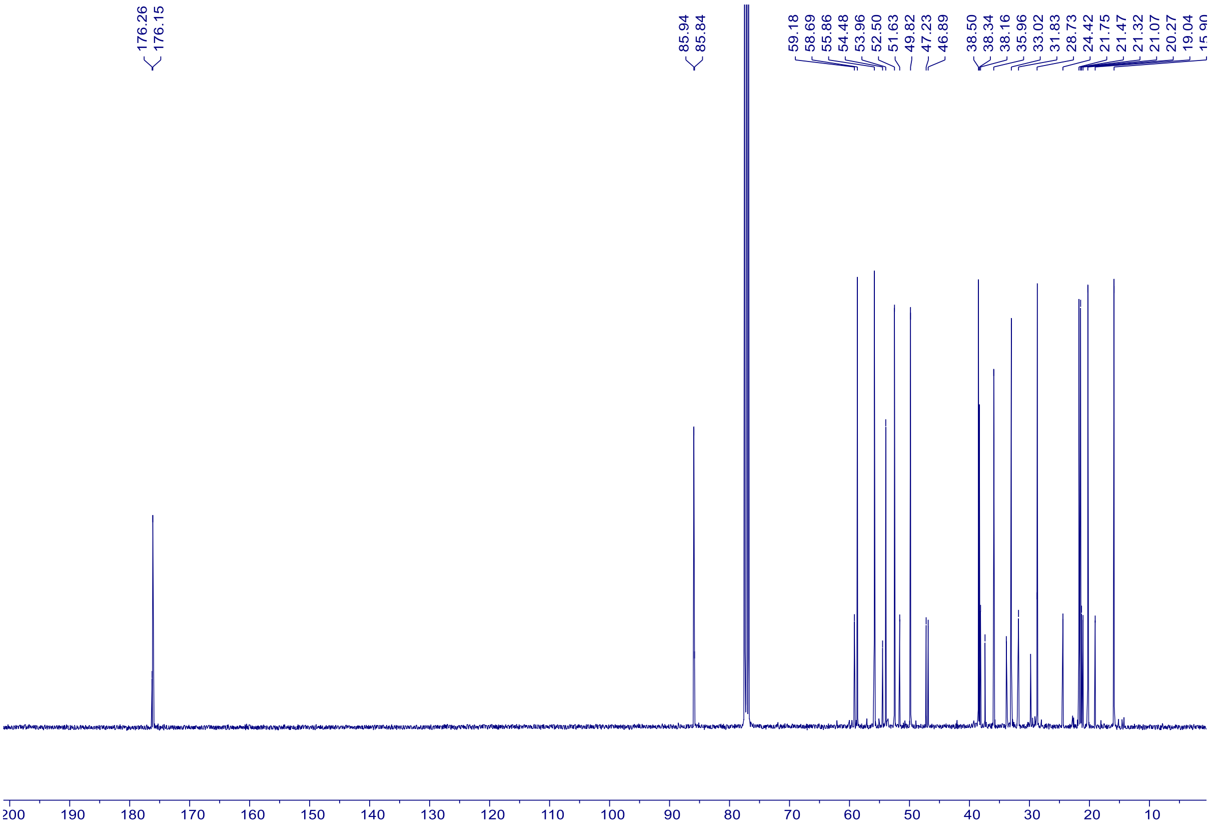

40 – HSQC (400 MHz, $CDCl_3$)

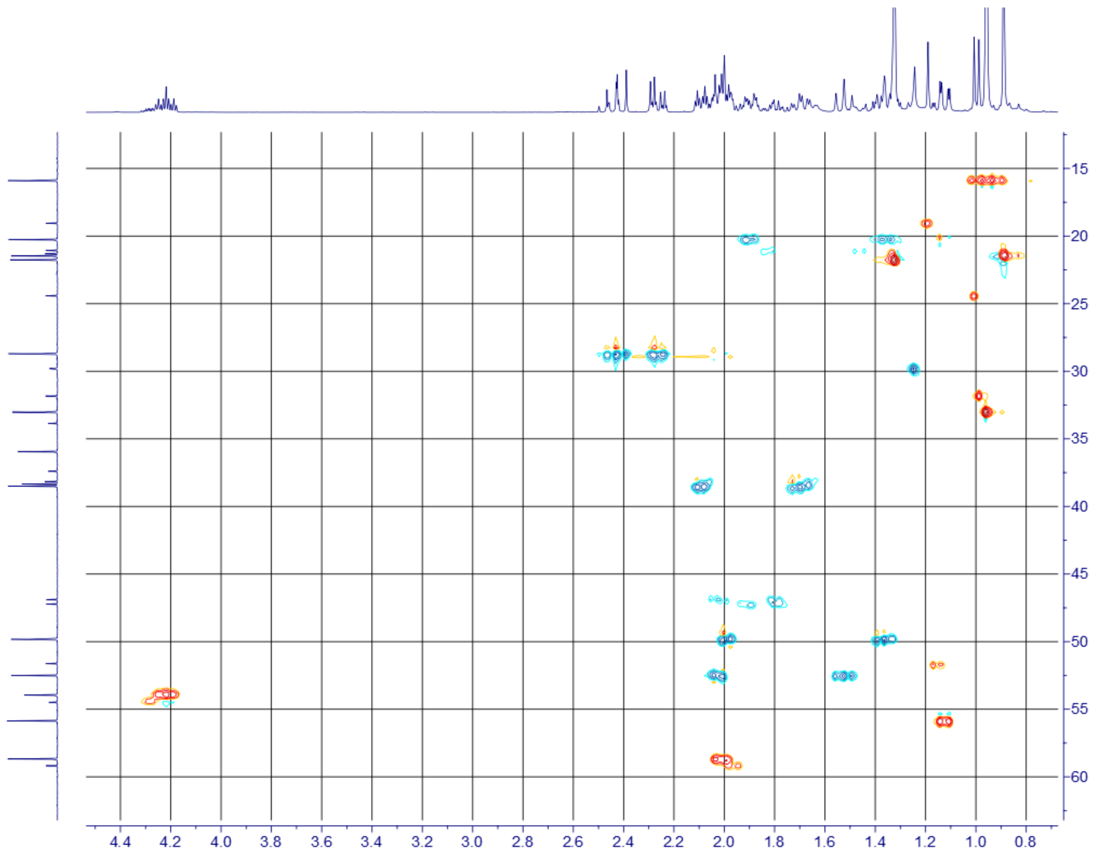

40 – HMBC (400 MHz, $CDCl_3$)

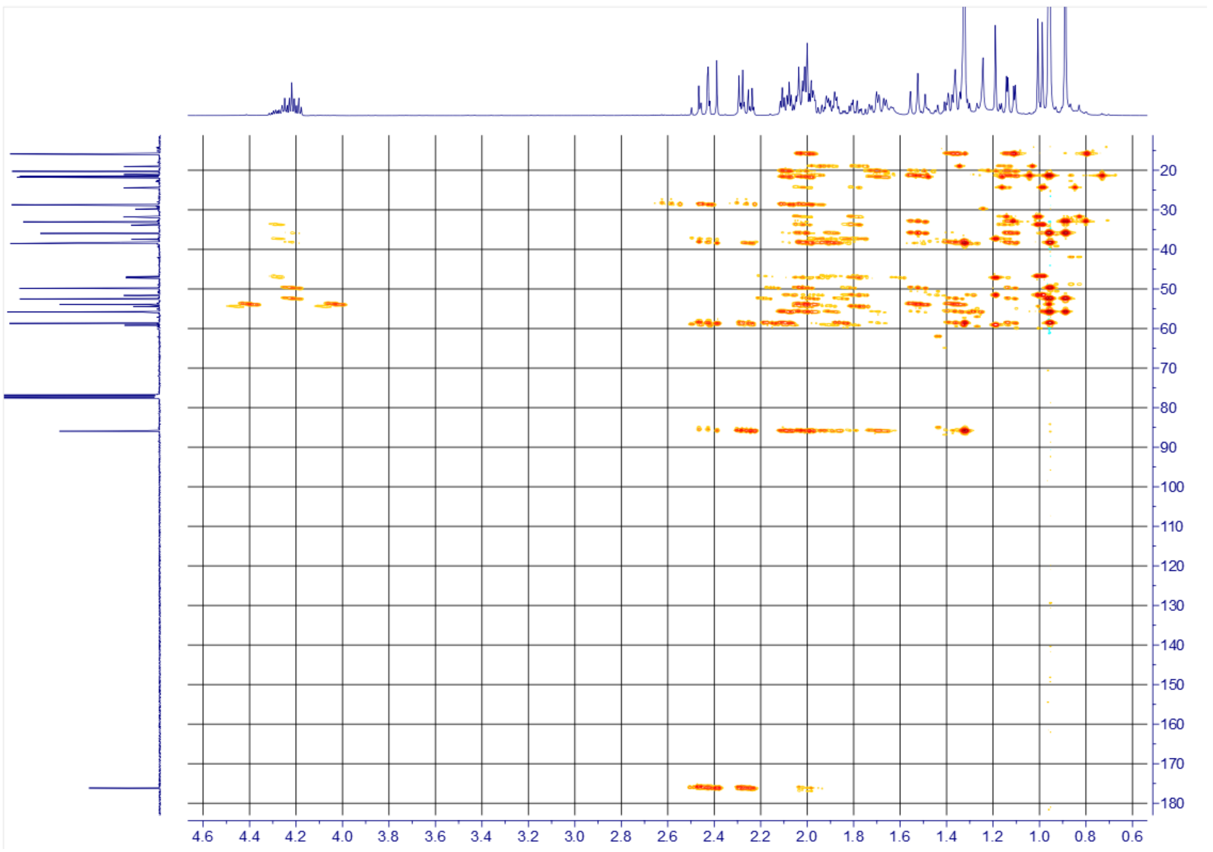

41 – ^{1}H NMR (400 MHz, $CDCl_3$)

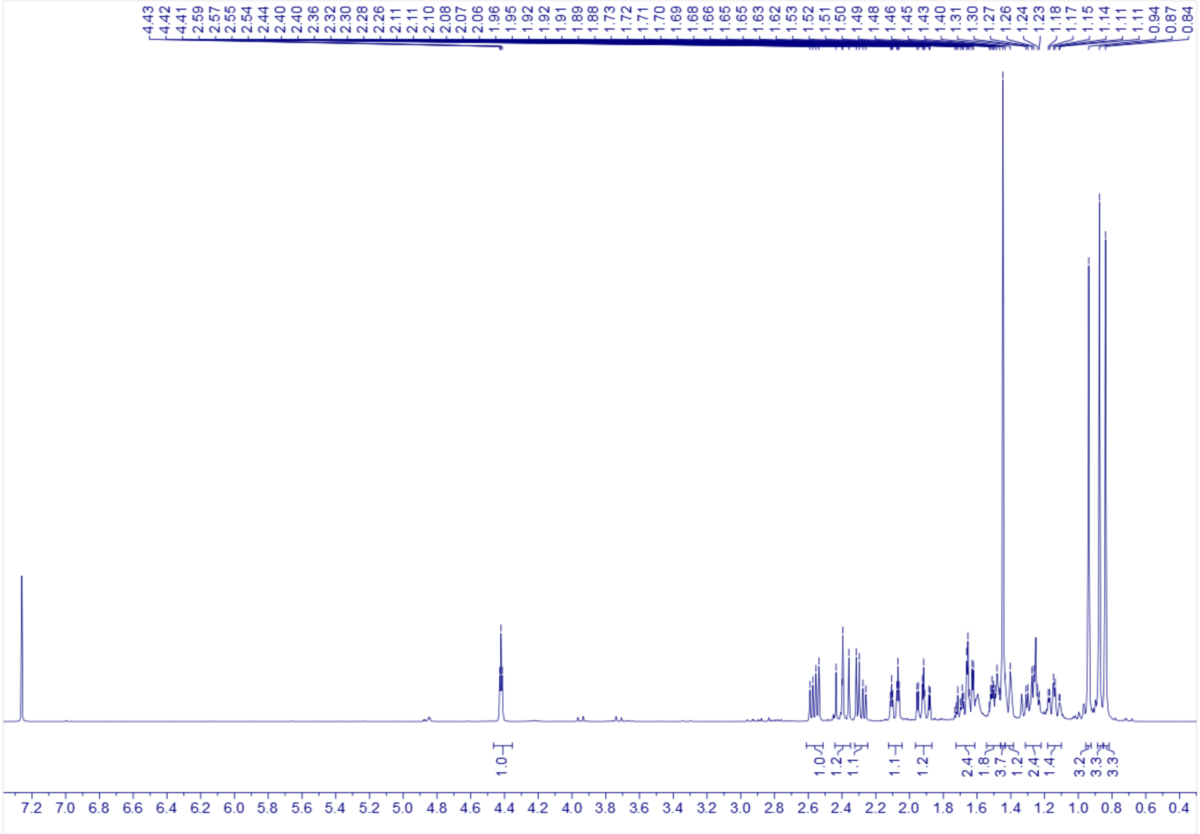

41 – ^{13}C NMR (101 MHz, $CDCl_3$)

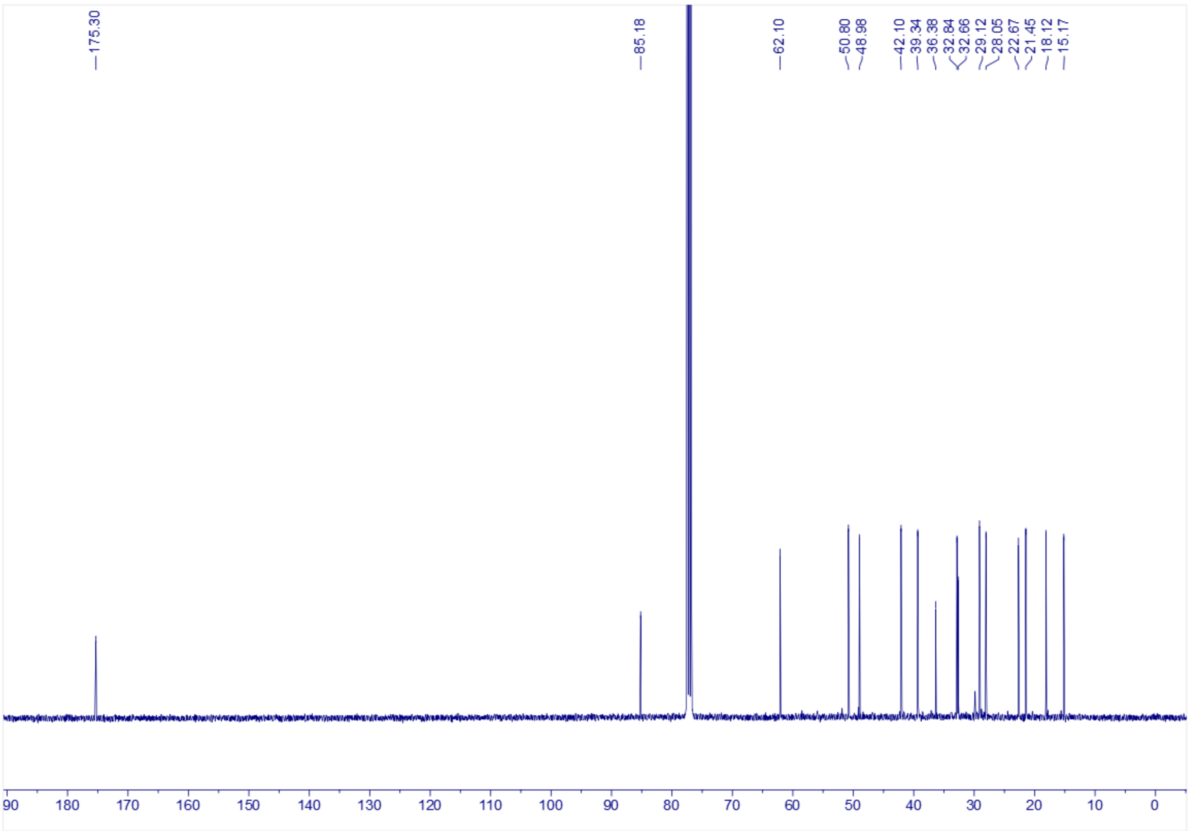

41 – HSQC (400 MHz, $CDCl_3$)

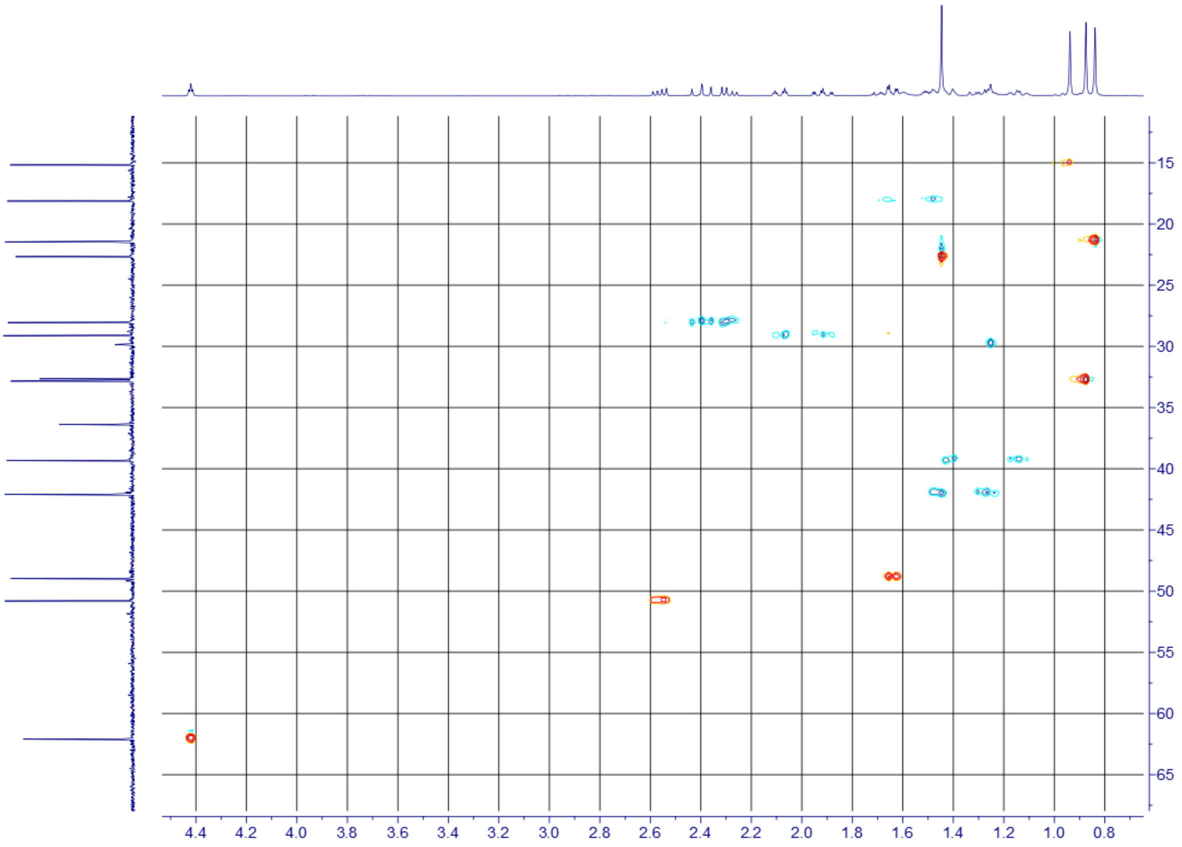

41 – HMBC (400 MHz, $CDCl_3$)

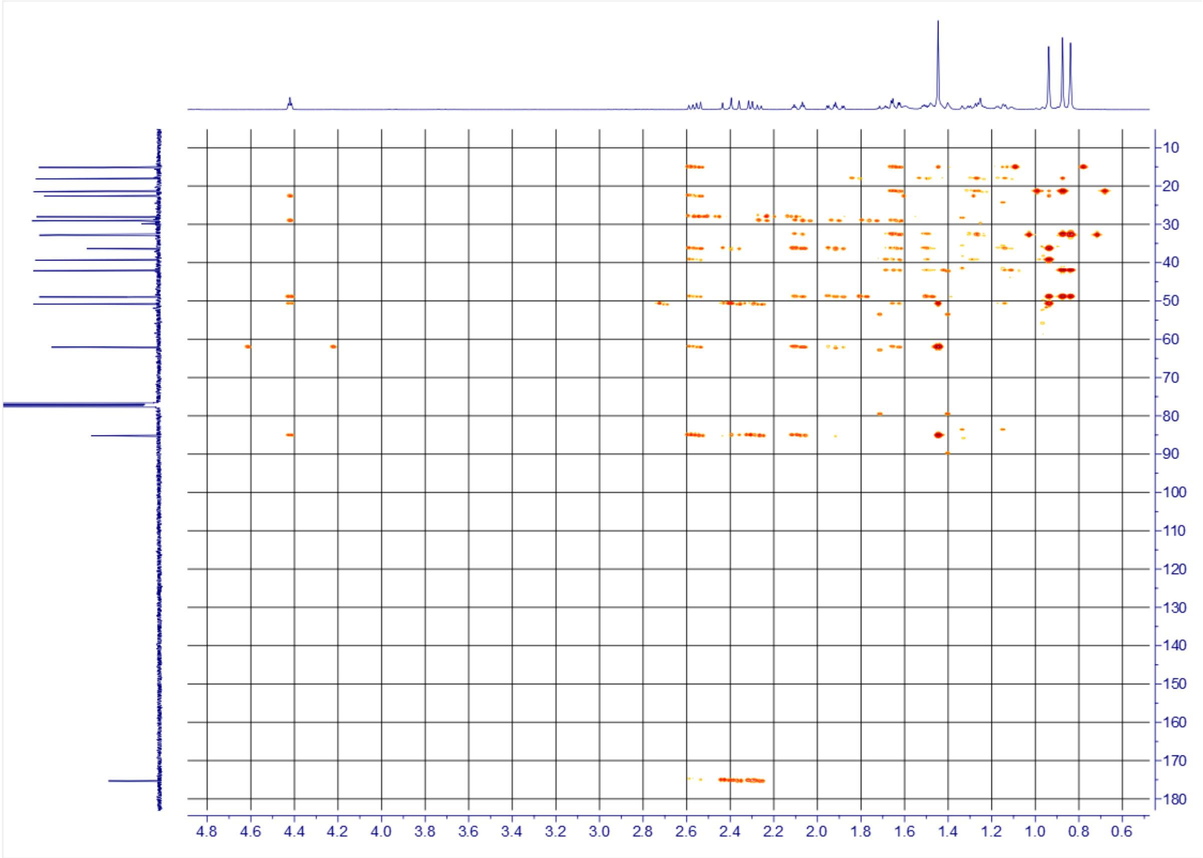

41’ – ^{1}H NMR (500 MHz, CDCl$_3$)

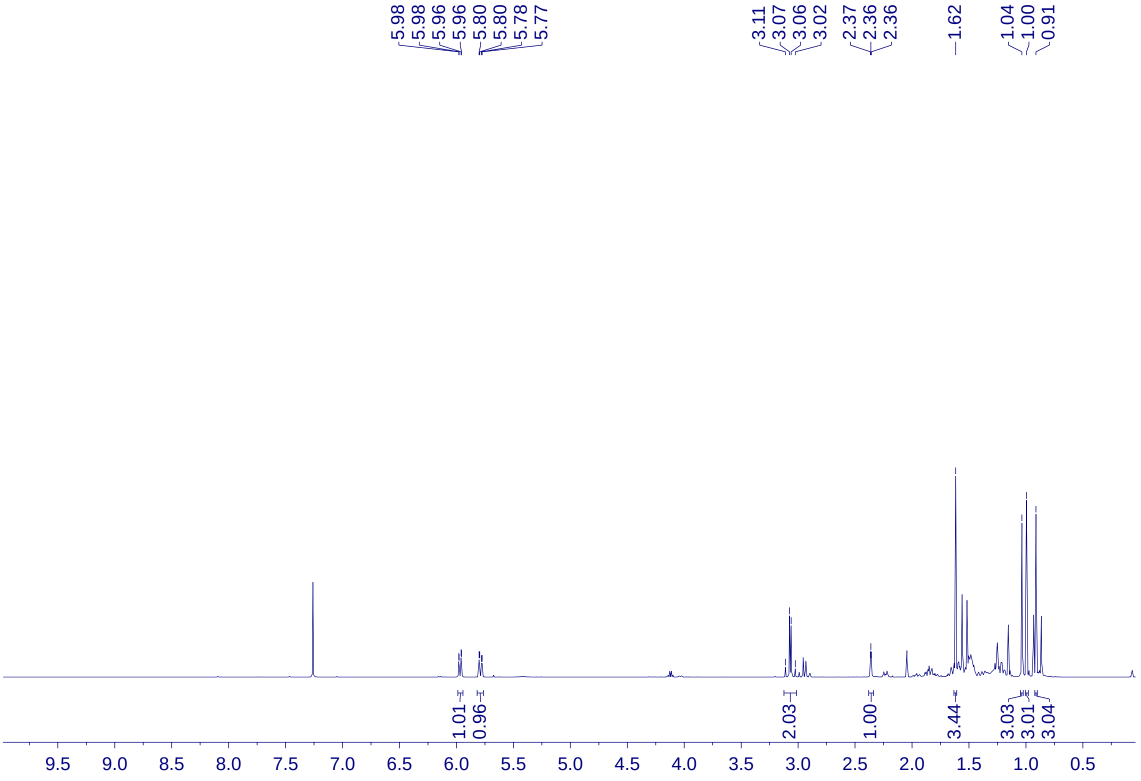

41’ – ^{13}C NMR (125 MHz, CDCl$_3$)

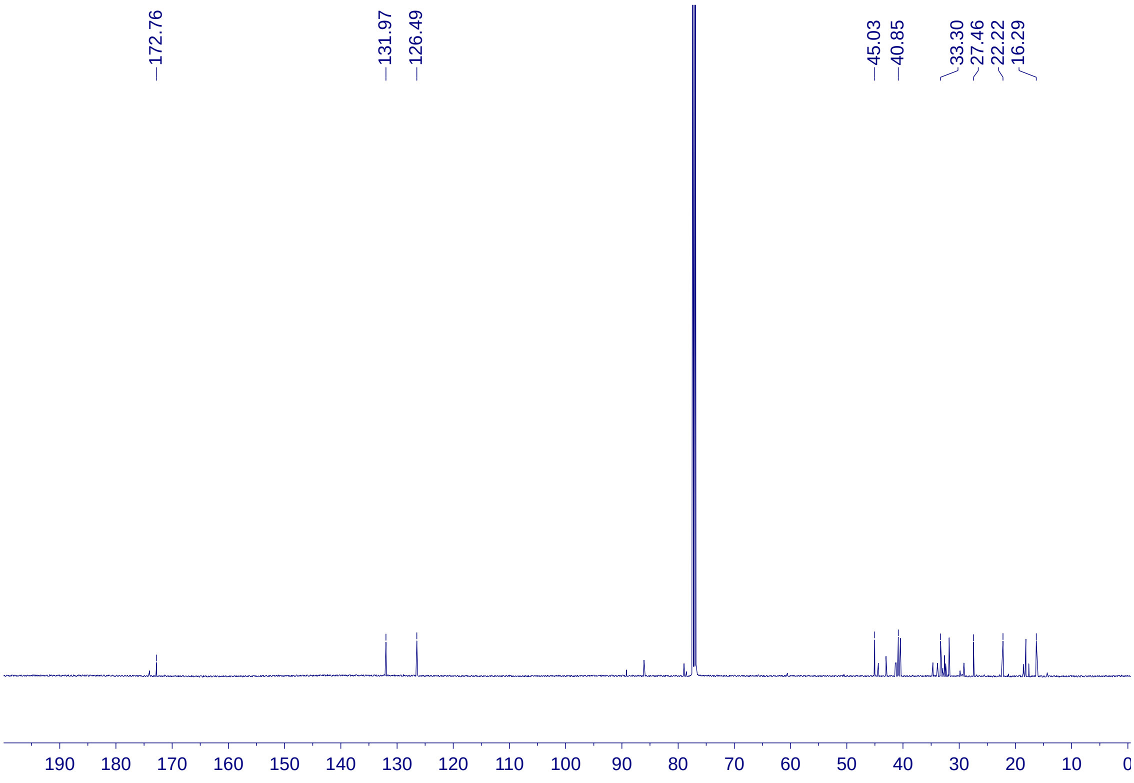

41' – NOESY (500MHz, $CDCl_3$)

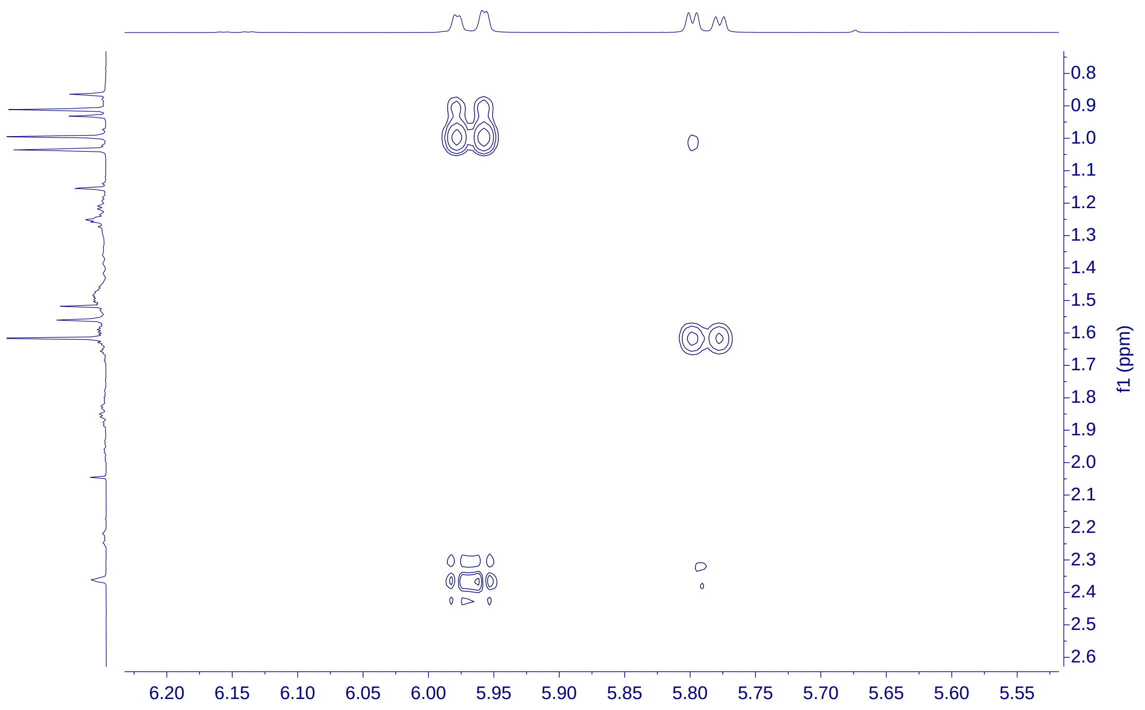

41' – COSY (500MHz, $CDCl_3$)

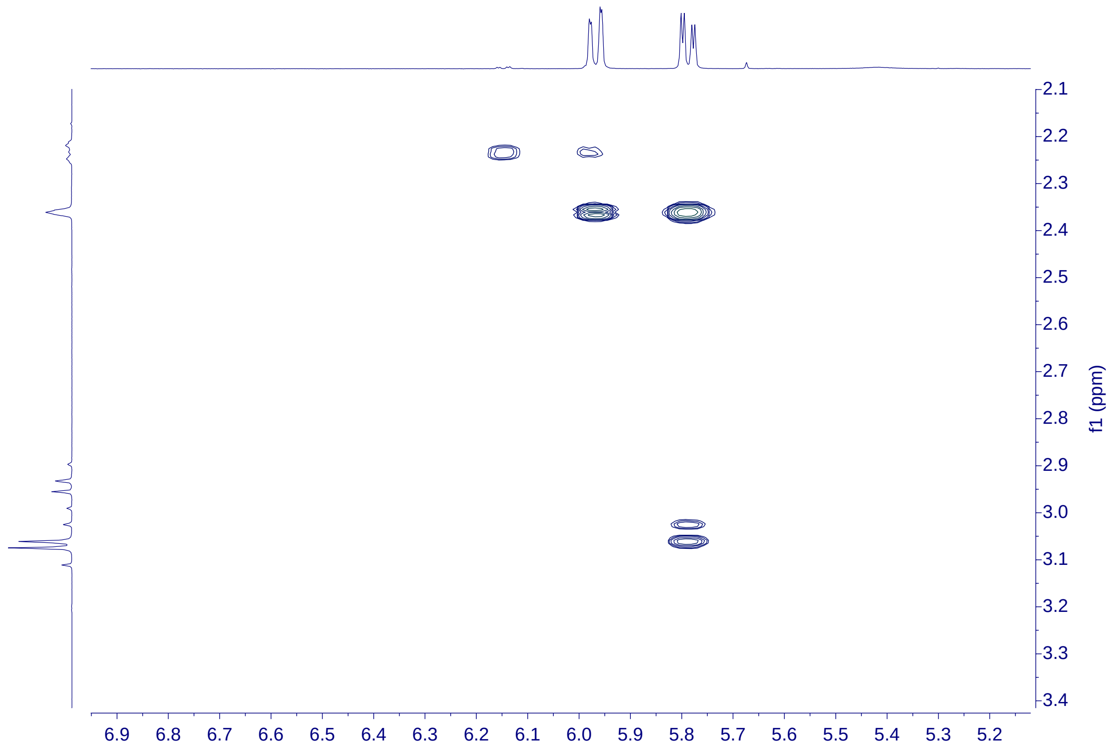

41’ – HSQC (500MHz, $CDCl_3$)

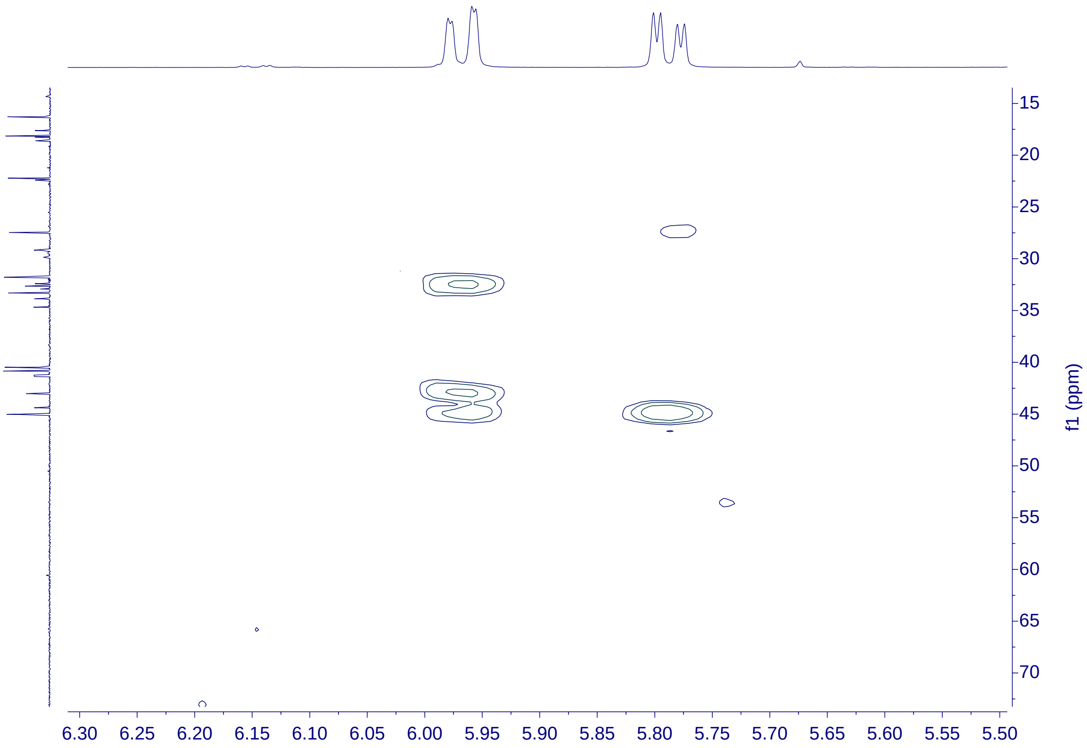

17 References

(1) Aliev, A. E.; Harris, K. D. M. *Magn. Reson. Chem.* **1993**, *31*, 54–57.

(2) Quinn, R. K.; Könst, Z. A.; Michalak, S. E.; Schmidt, Y.; Szklarski, A. R.; Flores, A. R.; Nam, S.; Horne, D. A.; Vanderwal, C. D.; Alexanian, E. J. *J. Am. Chem. Soc.* **2016**, *138*, 696–702.

(3) Tanner, D. D.; Arhart, R.; Meintzer, C. P. *Tetrahedron* **1985**, *41*, 4261–4277.

(4) Wypych, J. C.; Nguyen, T. M.; Bénéchie, M.; Marazano, C. *J. Org. Chem.* **2008**, *73*, 1169–1172.

(5) Orru, R. V. A.; Ruijter, E.; Vande Velde, C. M. L.; de Wit, M. J. M.; Mouarrawis, V.; van Schaik, T. B.; der Heijden, G. *Eur. J. Org. Chem.* **2019**, 5313–5325.

(6) Begtrup, M. *J. Chem. Soc. Perkin Trans. 2* **1983**, No. 9, 1609–1618.

(7) Saper, N. I.; Snider, B. B. *J. Org. Chem.* **2014**, *79*, 809–813.

(8) Otte, D. A. L.; Borchmann, D. E.; Lin, C.; Weck, M.; Woerpel, K. A. *Org. Lett.* **2014**, *16*, 1566–1569.

(9) Nacsa, E. D.; MacMillan, D. W. C. *J. Am. Chem. Soc.* **2018**, *140*, 3322–3330.

(10) Kuhn, H. J.; Braslavsky, S. E.; Schmidt, R. *Pure Appl. Chem.* **2004**, *76*, 2105–2146.

(11) Hubinger, S.; Nee, J. B. *J. Photochem. Biol.* **1995**, *86*, 1–7.

(12) Busch, G. E.; Mahoney, R. T.; Morse, R. I.; Wilson, K. R. *J. Chem. Phys.* **1969**, *51*, 449–450.

(13) Kraus, G. A.; Taschner, M. J. *J. Org. Chem.* **1980**, *45*, 1175–1176.

(14) Taylor, N. W.; Hildebrand, J. H.; Hildebrand, J. H. **1923**, *45*, 682–694.

(15) Carestia, A. M.; Ravelli, D.; Alexanian, E. J. *Chem. Sci.* **2018**, *9*, 5360–5365.

(16) Dauben, W. G.; Bridon, D. P.; Kowalczyk, B. A. *J. Org. Chem.* **1989**, *54*, 6101–6106.

(17) Manabe, Y.; Kitawaki, Y.; Nagasaki, M.; Fukase, K.; Matsubara, H.; Hino, Y.; Fukuyama, T.; Ryu, I. *Chem. Eur. J.* **2014**, *20*, 12750–12753.

(18) Longwitz, L.; Jopp, S.; Werner, T. *J. Org. Chem.* **2019**, *84*, 7863–7870.

(19) Namavari, M.; Satyamurthy, N.; Barrio, J. R. *J. Fluor. Chem.* **1995**, *72*, 89–93.

(20) Nouguier, R.; Surzur, J.-M.; Virgili, A. *Org. Magn. Reson.* **1981**, *15*, 155–157.

(21) Pitkänen, M. T.; Korhonen, I. O. O.; Korvola, J. N. J. *Tetrahedron* **1981**, *37*, 529–533.

(22) Aurell, M. J.; Ceita, L.; Mestres, R.; Tortajada, A. *Tetrahedron* **1997**, *53*, 10883–10898.

(23) Kim, M. J.; Mun, J.; Kim, J. *Tet. Lett.* **2017**, *58*, 4695–4698.

(24) Barry, C. N.; Evans, S. A. *J. Org. Chem.* **1981**, *46*, 3361–3364.

(25) Windhorst, A. D.; Bechger, L.; Visser, G. W. M.; Menge, W. P. M. B.; Leurs, R.;

Timmerman, H.; Herscheid, J. D. M. *J. Fluor. Chem.* **1996**, *80*, 35–40.

(26) Bodduri, V. D. V.; Choi, K. M.; Vaidya, R. R.; Patil, K.; Chirumarry, S.; Jang, K.; Yoon, Y. J.; Falck, J. R.; Shin, D. S. *Tet. Lett.* **2015**, *56*, 7089–7093.

(27) US5376673 (A), 1994.

(28) Short, M. A.; Blackburn, J. M.; Roizen, J. L. *Angew. Chemie Int. Ed.* **2018**, *57*, 296–299.

(29) Af Gennäs, G. B.; Talman, V.; Aitio, O.; Ekokoski, E.; Finel, M.; Tuominen, R. K.; Yli-Kauhaluoma, J. *J. Med. Chem.* **2009**, *52*, 3969–3981.

(30) De Buyck, L.; De Pooter, H. *Bull. Soc. Chim. Belges* **1992**, *101*, 807–815.

(31) Meng, Q. Y.; Wang, S.; König, B. *Angew. Chemie Int. Ed.* **2017**, *56*, 13426–13430.

(32) Shing, K.-P.; Liu, Y.; Cao, B.; Chang, X.-Y.; You, T.; Che, C.-M. *Angew. Chemie Int. Ed.* **2018**, *57*, 11947–11951.

(33) Jansen, A.; Pitter, S. *Monatshefte fur Chemie* **1999**, *130*, 783–794.

(34) Van Vliet, K. M.; Van Leeuwen, N. S.; Brouwer, A. M.; De Bruin, B. *Beilstein J. Org. Chem.* **2020**, *16*, 398–408.

(35) Eng, W. T.; Chan, B.; Blackman, A. G. *J. Am. Chem. Soc.* **2002**, *124*, 2078–2079.

(36) Schmidt, A. C.; Stark, C. B. W. *Org. Lett.* **2011**, *13*, 4164–4167.

(37) Li, C.; Zhao, P.; Li, R.; Zhang, B.; Zhao, W. *Angew. Chemie Int. Ed.* **2020**, *59*, 10913–10917.

(38) Negoro, T.; Ikeda, Y. *Bulletin of the Chemical Society of Japan*. 1986, pp 2547–2551.

(39) Méndez, J. J.; Eras, J.; Balcells, M.; Canela, R. *Synth. Commun.* **2006**, *36*, 1167–1175.

(40) Fotie, J.; Adolph, B. R.; Bhatt, S. V.; Grimm, C. C. *Tet. Lett.* **2017**, *58*, 4648–4651.

(41) Liu, W.; Groves, J. T. *J. Am. Chem. Soc.* **2010**, *132*, 12847–12849.

(42) Frisch, M. J.; Trucks, G. W.; Schlegel, H. B.; Scuseria, G. E.; Robb, M. A.; Cheeseman, J. R.; Scalmani, G.; Barone, V.; Peterson, G. A.; Nakatsuji, H.; Li, X.; Caricato, M.; Marenich, A.; Bloino, J.; Janesko, B. G.; Gomperts, R.; Mennucci, B.; Hratchian, H. P.; Ortiz, J. V.; Izmaylov, A. F.; Sonnenberg, J. L.; Williams-Young, D.; Ding, F.; Lipparini, F.; Egidi, F.; Goings, J.; Peng, B.; Petrone, A.; Henderson, T.; Ranasinghe, D.; Zakrzewski, V. G.; Gao, J.; Rega, N.; Zheng, G.; Liang, W.; Hada, M.; Ehara, M.; Toyota, K.; Fukuda, R.; Hasegawa, J.; Ishida, M.; Nakajima, M.; Honda, Y.; Kitao, O.; Nakai, H.; Vreven, T.; Throssell, K.; Montgomery, J. A.; Peralta, J. E.; Ogliaro, F.; Bearpark, M.; Heyd, J. J.; Brothers, E.; Kudin, N.; Staroverov, V. N.; Keith, T.; Kobayashi, R.; Normand, J.; Raghavachari, K.; Rendell, A.; Burant, J. C.; Iyengar, S. S.; Tomasi, J.; Cossi, M.; Millam, J. M.; Klene, M.;

Adam, C.; Cammi, R.; Ochterski, J. W.; Martin, R. L.; Morokuma, K.; Farkas, O.; Foresman, J. B.; Fox, D. . J. Wallingford CT 2016.

(43) Boyle, N. M. O.; Banck, M.; James, C. A.; Morley, C.; Vandermeersch, T.; Hutchison, G. R. *J. Cheminform.* **2011**, *3*, 1–14.

(44) Ede, M.; Levitt, M. H. *J. Magn. Reson.* **1998**, *132*, 220–239.

(45) Royal Society of Chemistry (2020) Periodic Table https://www.rsc.org/periodic-table/ (accessed Oct 2, 2020).

(46) De Vleeschouwer, F.; Van Speybroeck, V.; Waroquier, M.; Geerlings, P.; De Proft, F. *Org. Lett.* **2007**, *9*, 2720–2724.
